# A three-antigen *Plasmodium falciparum* DNA prime—Adenovirus boost malaria vaccine regimen is superior to a two-antigen regimen and protects against controlled human malaria infection in healthy malaria-naïve adults

**DOI:** 10.1371/journal.pone.0256980

**Published:** 2021-09-08

**Authors:** Marvin J. Sklar, Santina Maiolatesi, Noelle Patterson, Martha Sedegah, Keith Limbach, Nimfa Teneza-Mora, Ilin Chuang, K. Monique Hollis-Perry, Jo Glenna Banania, Ivelese Guzman, Harini Ganeshan, Sharina Reyes, Michael R. Hollingdale, Mimi Wong, Ashley Lindstrom, Anatalio Reyes, Yolanda Alcorta, Lindsey Garver, Kelli Bankard, Arnel Belmonte, Maria Belmonte, Jun Huang, Kalpana Gowda, Sandra Inoue, Rachel Velasco, Elke Bergmann-Leitner, Jack Hutter, Tida Lee, Nehkonti Adams, Sidhartha Chaudhury, Devin Hunt, Cindy Tamminga, Eleanor Berrie, Duncan Bellamy, Mustapha Bittaye, Katie Ewer, Carter Diggs, Lorraine A. Soisson, Alison Lawrie, Adrian Hill, Thomas L. Richie, Eileen Villasante, Judith E. Epstein, Christopher A. Duplessis

**Affiliations:** 1 Naval Medical Research Center, Silver Spring, Maryland, United States of America; 2 Henry M. Jackson Foundation, Bethesda, Maryland, United States of America; 3 Walter Reed Army Institute of Research, Silver Spring, Maryland, United States of America; 4 University of Oxford, Oxford, United Kingdom; 5 United States Agency for International Development (USAID), Washington, DC, United States of America; 6 Naval Medical Research Center, Malaria Department, Silver Spring, Maryland, United States of America; Beth Israel Deaconess Medical Center, UNITED STATES

## Abstract

**Background:**

A DNA-prime/human adenovirus serotype 5 (HuAd5) boost vaccine encoding *Plasmodium falciparum* (Pf) circumsporozoite protein (PfCSP) and Pf apical membrane antigen-1 (PfAMA1), elicited protection in 4/15 (27%) of subjects against controlled human malaria infection (CHMI) that was statistically associated with CD8+ T cell responses. Subjects with high level pre-existing immunity to HuAd5 were not protected, suggesting an adverse effect on vaccine efficacy (VE). We replaced HuAd5 with chimpanzee adenovirus 63 (ChAd63), and repeated the study, assessing both the two-antigen (CSP, AMA1 = CA) vaccine, and a novel three-antigen (CSP, AMA1, ME-TRAP = CAT) vaccine that included a third pre-erythrocytic stage antigen [malaria multiple epitopes (ME) fused to the Pf thrombospondin-related adhesive protein (TRAP)] to potentially enhance protection.

**Methodology:**

This was an open label, randomized Phase 1 trial, assessing safety, tolerability, and VE against CHMI in healthy, malaria naïve adults. Forty subjects (20 each group) were to receive three monthly CA or CAT DNA priming immunizations, followed by corresponding ChAd63 boost four months later. Four weeks after the boost, immunized subjects and 12 infectivity controls underwent CHMI by mosquito bite using the Pf3D7 strain. VE was assessed by determining the differences in time to parasitemia as detected by thick blood smears up to 28-days post CHMI and utilizing the log rank test, and by calculating the risk ratio of each treatment group and subtracting from 1, with significance calculated by the Cochran-Mantel-Haenszel method.

**Results:**

In both groups, systemic adverse events (AEs) were significantly higher after the ChAd63 boost than DNA immunizations. Eleven of 12 infectivity controls developed parasitemia (mean 11.7 days). In the CA group, 15 of 16 (93.8%) immunized subjects developed parasitemia (mean 12.0 days). In the CAT group, 11 of 16 (63.8%) immunized subjects developed parasitemia (mean 13.0 days), indicating significant protection by log rank test compared to infectivity controls (p = 0.0406) and the CA group (p = 0.0229). VE (1 minus the risk ratio) in the CAT group was 25% compared to -2% in the CA group. The CA and CAT vaccines induced robust humoral (ELISA antibodies against CSP, AMA1 and TRAP, and IFA responses against sporozoites and Pf3D7 blood stages), and cellular responses (IFN-γ FluoroSpot responses to CSP, AMA1 and TRAP) that were not associated with protection.

**Conclusions:**

This study demonstrated that the ChAd63 CAT vaccine exhibited significant protective efficacy, and confirmed protection was afforded by adding a third antigen (T) to a two-antigen (CA) formulation to achieve increased VE. Although the ChAd63-CAT vaccine was associated with increased frequencies of systemic AEs compared to the CA vaccine and, historically, compared to the HuAd5 vectored malaria vaccine encoding CSP and AMA1, they were transient and associated with increased vector dosing.

## Introduction

In August 2019, the World Health Organization issued a report [1] from its Strategic Advisory Group on Malaria Eradication calling for “transformative tools” in the fight against malaria, encouraging research and development on vector control, chemotherapy and vaccines. Gene-based vaccines are a promising approach for inducing the CD8+ T cell responses thought to mediate protection against liver stage malaria in humans [2] and could provide such a transformative tool. Although malaria vaccines based on DNA or adenoviruses alone have been immunogenic, eliciting robust CD8+ T cell responses, they have demonstrated sub-optimal protection in CHMI studies [3–5], while heterologous prime-boost strategies have proven more immunogenic and protective [6–9].

In a prior clinical trial, a recombinant DNA plasmid-prime/recombinant human adenovirus serotype 5 (HuAd5) boost vaccine encoding two pre-erythrocytic antigens, *Plasmodium falciparum* (Pf) circumsporozoite protein (PfCSP) and Pf apical membrane antigen-1 (PfAMA1) sterilely protected 4/15 (27%) subjects against controlled human malaria infection (CHMI) [6]. Protection was significantly associated with CD8+ T cell responses [6], with effector memory CD8+ T cells targeting class I-restricted CSP and AMA1 epitopes identified as the likely effector mechanism [10].

Although the vaccine efficacy (VE) generated by the DNA-prime/HuAd5 prime-boost regimen using PfCSP and PfAMA1 appeared promising. However, concerns regarding the safety of HuAd5 [11], and effects of naturally-acquired neutralizing antibodies (Nabs) to HuAd5 on HuAd5 vaccine immunogenicity [6, 12], led to development of an alternative adenovirus vector, chimpanzee adenovirus 63 (ChAd63), at the University of Oxford. Simian adenoviruses including ChAd63 are not known to cause pathology or illness in humans and the ChAd63 vectors are replication-deficient [13]. Nabs to ChAd63 are low in human populations [7], do not neutralize ChAd63 and are unlikely to affect ChAd63-induced immune responses [14]. The higher levels of protection seen with whole organism-based malaria vaccines [15, 16] as compared with single-antigen subunit vaccines [17], suggest that multiple antigens will be required if there is to be an effective subunit vaccine. We hypothesized that the addition of PfME-TRAP to the PfCSP+PfAMA1 formulation would induce additive or synergistic T-cell responses that would improve efficacy. A ChAd63 prime/modified vaccinia virus Ankara (MVA) boost vaccine expressing the PfME-TRAP antigen (malaria multiple epitopes, ME, fused to the Pf thrombospondin-related adhesion protein, TRAP) elicited monofunctional CD8+ IFN-γ T cell responses that correlated with sterile protection in 3/14 (21%) subjects [17].

We established a collaboration with the University of Oxford in which we replaced HuAd5 with ChAd63 to avoid neutralizing antibodies (Nabs) and repeated our study using the DNA/ChAd63 two antigen (CSP, AMA1: CA) vaccine and a novel three antigen (CSP, AMA1, ME-TRAP: CAT) vaccine. A DNA vector encoding the native sequence TRAP lacking the ME component, similar to the codon-optimized DNA-TRAP vector used in this study, has been studied in humans in the United States [5, 18, 19]. Two of the three DNA plasmids had been previously assessed in the United States in the earlier HuAd5 trial (CSP and AMA1) [6], and a TRAP plasmid (lacking the ME) was assessed in a multiple DNA plasmid trial [5]. The three boosting vectors, ChAd63-CSP, ChAd63-AMA1, and ChAd63-ME-TRAP, have been studied in the United Kingdom (UK) [7, 20–23]. The DNA priming immunizations were administered via the Biojector^®^2000 needle-free injection device (Inovio Pharmaceuticals, Inc., Plymouth Meeting, PA), while the adenovirus-vectored vaccine was administered vis direct intramuscular (IM) injection via needle and syringe.

## Methods

The Study Event Schedule and Procedures for this trial and supporting CONSORT checklist are available as S1 File and S1 Checklist.

### Objectives

The primary objectives were to assess (1) the safety and tolerability of a DNA vaccine prime with ChAd63 vaccine boost encoding the CA antigens (DNA/ChAd63-CA), and (2) the safety and tolerability of a DNA vaccine prime with ChAd63 vaccine boost encoding the CAT antigens (DNA/ChAd63-CAT) in healthy malaria-naïve adults.

The secondary objectives were to assess: (1) VE of DNA/ChAd63-CA, and DNA/ChAd63-CAT in healthy malaria-naïve adults subjected to CHMI with Pf3D7 sporozoites administered by mosquito bites; (2) cellular immunogenicity by FluoroSpot assay; (3) humoral immunogenicity by enzyme-linked immunosorbent assay (ELISA) to CSP, AMA1 and TRAP antigens; (4) humoral immunogenicity by immunofluorescence assay (IFA) against sporozoite and erythrocytic stage parasites; (5) any association of Nab to HuAd5 with VE and humoral and cellular immunogenicity; and (6) any degree of seroconversion to HuAd5 among subjects after immunization with ChAd63.

### Ethics

The study was conducted at the Naval Medical Research Center (NMRC) Clinical Trials Center from 2018–2019; CHMIs were conducted at the Walter Reed Army Institute of Research (WRAIR) secure insectary. The study protocol was reviewed and approved by the NMRC Institutional Review Board (IRB) in compliance with all federal regulations governing the protection of human subjects. WRAIR and NMRC hold a Federalwide Assurance from the Office of Human Research Protections (OHRP) under the Department of Health and Human Services. NMRC also holds a Department of Defense/Department of the Navy Assurance for human subject protections. All key personnel were certified as having completed mandatory human subjects’ protection curricula and training under the direction of the WRAIR IRB and Human Subjects Protections Branch (HSPB) or the NMRC IRB and Office of Research Administration (ORA). All potential study subjects provided written, informed consent before screening and enrollment and had to pass an assessment of understanding. This study was conducted according to the Declaration of Helsinki as well as principles of Good Clinical Practices under the United States Food and Drug Administration (FDA) Investigational New Drug (IND) application BB-IND 17572. This trial was performed under an IND allowance by the FDA and was registered on ClinicalTrials.gov (NCT03341754).

### Study design

This study was an open label, randomized controlled Phase 1 trial, assessing safety, tolerability, and VE against CHMI in healthy, malaria naïve adults. It is an open label design as subjects were aware if they were receiving a vaccine despite being randomized to either the CA or CAT groups. The infectivity controls received the CHMI without prior immunizations and were enrolled sequentially. Immunized subjects were randomly assigned (block randomization) to one of the two vaccine groups and were blinded to their immunization group. The clinical study team were aware of the subject assignments, but all laboratory support and microscopists were blinded. The design of this study is summarized in S1 Fig in S2 File.

Forty subjects were to receive three DNA priming immunizations at weeks 0, 4, and 8 followed by ChAd63 boosting immunizations on week 24. Four weeks after the boost, immunized subjects and 12 infectivity controls were to receive CHMI by mosquito bite using the Pf3D7 (a clone of PfNF54) strain used in previous NMRC clinical trials [24]. All subjects were monitored for adverse signs and symptoms, laboratory abnormalities, and humoral and cellular immune responses. Giemsa-stained malaria blood smears were read by certified microscopists on days 6 through 21 post-challenge, then every other day through day 28 in subjects remaining smear negative. Subjects exhibiting positive results for malaria were promptly treated as described below.

### Study subjects and eligibility

Enrollment was limited to healthy malaria-naïve adults aged 18–50 years who passed screening by medical history, physical examination, electrocardiogram, and laboratory testing. Cardiac risk screening was conducted to identify and exclude individuals at moderate or high risk of developing symptomatic coronary artery disease during the next 5 years [25].

### Vaccines

#### DNA vaccines

3D7 PfCSP and PfAMA1 genes were identical to those used previously [5, 6] and the 3D7 PfTRAP gene was a codon-optimized version of that used previously [7] (S1 Table in S2 File, S2 Fig in S2 File). The expressed Pf proteins for the DNA prime and ChAd63 boost are similar (S2 Fig in S2 File). DNA priming immunizations were three separate doses 4 weeks apart in each deltoid muscle by Biojector^®^ 2000 needle-free injection device (Inovio Pharmaceuticals, Inc., Plymouth Meeting, PA). The D-CA component was administered at 2 mg (1 mg D-CA/mL), split into two 1 mL intramuscular injections each of 1 mg of D-C and 1 mg D-A plasmid DNA (pDNA) constructs. Each construct was vialed separately at 3 mg/mL, 0.5 mL/vial; 0.4 mL D-C and 0.4 mL D-A were combined then diluted with 1.6 mL of PBS and mixed to formulate the D-CA vaccine in 2.4 mL, sufficient for 2 injections of 1 mg/mL of D-CA (0.5 mg D-C and D-A per mL). The D-CAT component was administered at 3 mg (1.5 mg D-CAT/mL), split into two 1 mL intramuscular injections of 1 mg of D-C, 1 mg D-A, and 1 mg D-T pDNA products. Each construct was vialed separately at 3 mg/ml, 0.5 mL/vial, and 0.4 mL D-C, 0.4 mL D-A, 0.4 mL D-T were combined then diluted with 1.2 mL of PBS and mixed to formulate the D-CAT vaccine in 2.4 mL, sufficient for 2 injections of 1.5 mg/mL of D-CAT (0.5 mg each D-C, D-A, and D-T per mL).

#### ChAd63 vaccines

ChAd63-C, ChAd63-A, and ChAd63-ME-TRAP, were developed by The University of Oxford and Okairos AG (Basel, Switzerland), and manufactured by the Clinical BioManufacturing Facility (Oxford, UK) under UK Medicines and Healthcare products Regulatory Agency GMP guidelines [26]. The CSP construct is based on Pf3D7, but unlike DNA-CSP, has a longer deletion of the repeat region and an 11 amino acid (aa) deletion at the C-terminus; the AMA1 construct is based on two divergent alleles of AMA1, Pf3D7 (ectodomain) and PfFVO (full length, but lacking the signal sequence), whereas DNA-AMA1 is based only on Pf3D7; the ME-TRAP construct is based on the PfT9/96 strain and a string of 20 CD4+ and CD8+ epitopes (ME) [27], whereas DNA-TRAP is based on Pf3D7 and has a slightly shorter deletion approximately 200 aa from the C-terminus (S2 Fig in S2 File). The dose of the ChAd63-CA vaccine component was 1.0 x 10^11^ viral particles (vp)/dose (5 x 10^10^ vp/construct). Appropriate volumes of each of the products were combined and provided as 1 dose containing 5 x 10^10^ vp of ChAd63-C and 5 x 10^10^ vp of ChAd63-A. The dose of ChAd63-CAT vaccine regimen was 1.5 x 10^11^ vp/dose (5 x 10^10^ vp/construct). Appropriate amounts of each product were combined and provided as 1 dose containing 5 x 10^10^ vp of each of the constructs, ChAd63-C, ChAd63-A, and ChAd63-ME-TRAP. These vaccines were administered by intramuscular injection (IM) with needle and syringe into the deltoid muscle of the non-dominant arm.

### Dose justification

DNA dose selection was based upon the safety, immunogenicity, and protection data from the DNA/HuAd5 Trial [6]. Although the use of a codon-optimized version of DNA-T was first in humans, the native sequence DNA-T plasmid was safely administered at a dose of 0.5 mg administered 3 times in humans in the United States as part of the MuStDO5 trial [5], and in the UK at doses up to 2 mg [27, 28]. Based upon safety and immunogenicity data derived from hundreds of healthy subjects across varied populations, the consensus optimal dosing for the ChAd63-vectored components doses (5 x 10^10^ vp) [29, 30].

### Sample size

The sample size was designed to demonstrate that the frequency of serious or severe vaccine-related adverse events (AEs) was sufficiently low to allow continued testing in a larger number of subjects in the future. The predicted rates of serious or severe vaccine-related AEs in the general population were determined using the exact binomial method (1-p) n = 1-c where p is the probability that a subject has an event, n is the total number of subjects and c is the level of confidence [4]. In addition, the sample size was powered to detect a two day mean delay in patency in the immunized group compared to the infectivity controls (80% power, α = 0.05, one-sided) [31, 32], as this delay indicates a reduction in liver parasite burden.

### Controlled human malaria infection (CHMI)

CHMI was administered by five bites of *P*. *falciparum* 3D7-infected mosquitoes as previously described [15, 16]. The presence of two parasites was required for a positive diagnosis, leading to immediate antimalarial treatment. Four tablets of Malarone^®^ (250 mg atovaquone/100 mg proguanil per tablet) administered orally once per day for 3 days was given as first line therapy. Subjects diagnosed with parasitemia by thick blood smear (TBS) were monitored daily by symptom checks and blood smears until two consecutive daily negative smears were documented. Subjects who remained negative for parasitemia were similarly monitored daily until day 18 post CHMI, then approximately every other day until day 28.

### Safety and tolerability

Women underwent pregnancy testing prior to each immunization. Subjects underwent follow-up evaluations (S2 Table in S2 File) after each immunization: 30 minutes post-immunization monitoring, telephone call on Day 1, and clinical follow-ups on day 2, day 7 and day 14 after immunization, day 28 and day 70 following the third immunization, and day 2, day 7 and day 27 following the boosting immunization. All evaluations included AE monitoring, vital signs, physical examination, blood procurement for safety laboratory tests, and at pre-defined days for immunologic assays. AEs after each immunization evaluated safety, tolerability and reactogenicity. Solicited AEs were recorded on days 0, 1, 2 and 7, unsolicited AEs on days 0, 1, 2, 7, 14 and 28 and laboratory tests (complete blood count, aspartate, aminotransferase [AST], alanine aminotransferase [ALT], creatinine, and total bilirubin) on days 0, 2, 7 and 28 following each immunization. Abnormal laboratory values were assessed until 28 days post immunizations. Solicited AEs, consistent with previously reported systemic reactions [6], included headache, fever (objective or subjective), chills, rigors, myalgia, arthralgia, nausea, vomiting, diarrhea, abdominal pain, fatigue, malaise, headache, dizziness, cough, and a ’flu-like syndrome’. Unsolicited adverse events were collected by direct subject interview clarifying any symptoms experienced beyond the solicited AEs at each encounter. Monitoring for serious AEs (SAEs) was performed until the week 40 termination of the study.

### Immunologic endpoints

Samples for measuring cell-mediated immunity (FluoroSpot assay) were collected pre-immunization, 28 days after the third DNA (post-DNA) immunization, 112 days after the third DNA immunization and within seven days prior to the ChAd63 boosting immunization (pre-ChAd63), 27–28 days post-ChAd63 boosting immunization (1–2 days pre-CHMI), 35 days post-CHMI and 90 days post-CHMI (final). Antibody levels (ELISA, IFA) were measured at similar time points and at 14 and 28 days after each DNA immunization (S1 Fig in S2 File). Nab to HuAd5 were measured [33] on the day of the ChAd63 boosting immunization, and 27 days post-boost (prior to CHMI).

### Enzyme-linked immunosorbent assay (ELISA) assay

The ELISAs using CSP and AMA1 were performed in the WRAIR Serology Laboratory [34, 35]: CSP repeat region (CSPrp), CS(NANP)_6_C, 0.25 μg/mL; CSP Full Length (CSPFL) 0.25 μg/mL and CSP C-terminal region peptide (CSPf16) 2 μg/mL [36], AMA1 recombinant ectodomain 100 μg/mL [37, 38] plate antigens. The ELISA using plate antigen recombinant Pf3D7 TRAP ectodomain produced in HEK293 cells 1 μg/mL was performed at the University of Oxford [39]. Antibody data are reported as end-point dilution at an optical density of 0.5 (CSP, AMA1) or IgG ELISA Unit (EU) titers (TRAP).

### Immunofluorescence antibody assay (IFA)

IFAs used air dried PfNF54 sporozoites and Pf3D7 blood stages from the WRAIR Entomology Branch. End-point titers were determined as the last dilution above the background that fluorescent parasites were observed [40].

### Neutralizing antibodies to HuAd5 (Nab)

Nabs to HuAd5 were measured as previously described [33].

### Interferon-gamma FluoroSpot assay

Antigen-specific circulating peripheral blood mononuclear cells (PBMCs) secreting gamma-interferon (INF-γ) were evaluated as previously described [6, 16]. Full length PfCSP and PfAMA1 were covered by a series of 65 (PfCSP) and 153 (PfAMA1) 15mer amino acid (aa) sequences overlapping by11 aa. PfCSP 15mers were combined into 9 individual peptide pools (Cp1-Cp9), and PfAMA1 15mers were combined into 12 individual peptide pools (Ap1-Ap12). Full length PfTRAP strain T9/96 (TT) and PfTRAP strain 3D7 (TD) were covered by 20mer peptides overlapping by 11 aa; 56 PfTRAP TT and TD peptides were combined into 6 individual peptide pools (TT1-TT6; TD1-TD6). PBMCs were stimulated with each individual peptide pool in capture plates coated with both anti-IFN-γ and anti-Granzyme B, (whereas ELISpot used plates coated with anti-IFN-γ alone), and activities expressed as spot forming cells (sfc) secreting IFN-γ/10^6^ PBMC. A positive response to each individual peptide pool was defined as previously described [6]. A subject was considered positive to a particular antigen if there was a positive response to one or more of the peptide pools for that antigen.

### Statistical analyses

The ages in each group were analyzed using ANOVA followed by Tukey testing. Differences in gender and race between groups was analyzed via Fishers exact test. Descriptive statistics (percentage of study subjects, rate/immunization) were used to characterize the occurrence of local and systemic solicited and unsolicited adverse events in immunized subjects. Measurements with normal distributions expressed as means of continuous data (e.g., magnitude of responses) were assessed using the Student’s t test (2-tailed), paired if pre-immunization values were compared with post-immunization values, and unpaired if comparisons were made between groups. For comparisons of the local and systemic AEs between each DNA vaccination and the ChAd63 boost, we utilized the exact McNemar’s test. The Mann-Whitney test was used to compare times to parasitemia in the three subject groups.

Kaplan-Meier survival curves for each group showing the proportion of subjects remaining free of parasitemia over time were tested for differences using the log-rank test. VE was assessed by the log rank test, and by calculating the risk ratio of each treatment group and subtracting from 1, with significance calculated by the Cochran-Mantel-Haenszel method. Of note, the proportional hazards assumptions were not satisfied excluding a hazards ratio calculation. A Mann-Whitney test was utilized to assess differences in the time to parasitemia in non-protected subjects.

A mixed-effects linear model was used to compare geometric means for ELISA, IFA, and FluoroSpot between different time points, adjusting for comparisons between the pre-immune, pre-ChAd63 dose, and pre-challenge time points using Dunnette’s method. All antibody responses were log10 transformed. Univariate analysis was carried out to determine if any of the immune measures were significantly different between protected and non-protected subjects using a Shapiro-Wilk test for normality followed by a student’s t-test or Mann-Whitney test. The Accelerated Failure Time model was used to determine the relationship between immune measures and time to parasitemia (delay in onset of parasitemia indicating partial protection), censoring the fully protected volunteers on day 28. Two-sided p = <0.05 was considered significant in all tests. Rank Spearman correlations [33] were used to examine the relationship of fold-changes of Nab titers and immune responses before and after ChAd63 immunization. A precise comparison between cellular immune responses in this trial and those recorded in the prior HuAd5 trial could not be conducted because different assays were used (FluoroSpot vs. ELISpot).

## Results

### Study flow

**Fig 1:**
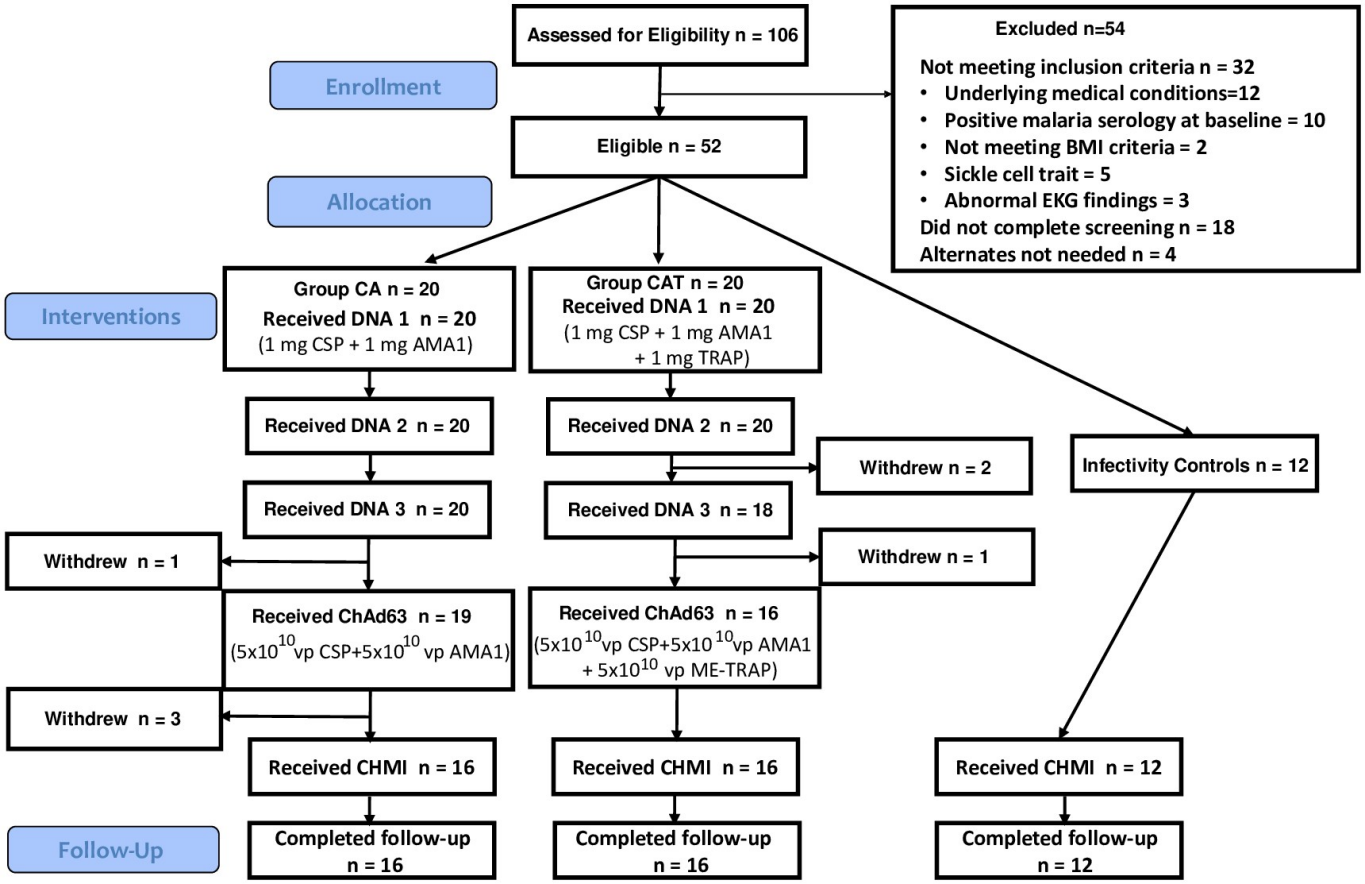
Flow diagram of immunized and control subjects. Fifty-two subjects met all eligibility criteria and were randomly allocated to the CA group (n = 20), CAT group (n = 20) and infectivity controls (n = 12). CA group: Prior to the ChAd63 immunization, 1 subject withdrew for personal reasons; prior to CHMI, 3 subjects were withdrawn: 1 due to a laboratory abnormality thought possibly related to the study interventions, 1 due to relocation, and 1 due to suicidal ideation requiring inpatient admission (unrelated to the study intervention). CAT group: Prior to the third DNA immunizations, 2 subjects withdrew: 1 subject due to an unrelated SAE and 1 subject due to a medical issue (commencing latent tuberculosis treatment); prior to ChAd63 immunizations, 2 subjects withdrew, 1 subject due to pregnancy, and 1 subject due to a SAE grade 4 laboratory abnormality (neutropenia) likely attributed to benign ethnic neutropenia but possibly to study intervention.

Participant flow is shown in Fig 1. Recruitment of vaccine recipients took place at the NMRC Clinical Trials Center, Bethesda, MD between February–May 2018, and CHMI commenced for vaccine recipients and for infectivity controls in October–November 2018. One hundred and six healthy, malaria-naïve, civilian, and military adult men and women aged 18–50 years were assessed for eligibility and 54 were excluded. The remaining 52 subjects who met all screening criteria were randomly assigned to the CA vaccine group (n = 20), the CAT vaccine group (n = 20) or the infectivity control group (n = 12). The demographics of each group were approximately balanced in gender, age, and ethnic background, except age in the CA group was significantly higher than in the CAT groups and infectivity controls (ANOVA followed by Tukey testing) (p = 0.0123) (Table 1). Sixteen subjects each in the CA and CAT groups received all scheduled immunizations and, with the 12 infectivity controls, were subject to CHMI.

**Table 1:** Forty volunteers were enrolled into the immunization groups (20 group CA and 20 group CAT). 16 in each group remained through CHMI. Infectivity controls (12) were enrolled later, in time for CHMI on week 28.

	Immunized Group CA n = 20	Immunized Group CAT n = 20	Infectivity Controls n = 12
**Male**	11 (55%)	14 (70%)	8 (66.7%)
**Female**	9 (45%)	6 (30%)	4 (33.3%)
**Mean Age ± standard deviation**	33.1 ± 9.50	27.0 ± 6.47	26.0 ± 3.95
**White or Caucasian**	9 (45%)	12 (60%)	8 (66.7%)
**Black or African American**	6 (30%)	5 (25%)	2 (16.7%)
**Asian**	3 (15%)	0	1 (8.3%)
**Native Hawaiian or Other Pacific Islander**	1 (5%)	0	0
**American Indian or Alaska Native**	0	0	1 (8.3%)
**Unknown/Other**	1 (5%)	3 (15%)	0

There were no significant differences between groups, except the mean age of the CA group was significantly (ANOVA followed by Tukey testing) higher than the infectivity controls.

### Safety and tolerability

#### Solicited adverse events (AEs)

**Fig 2:**
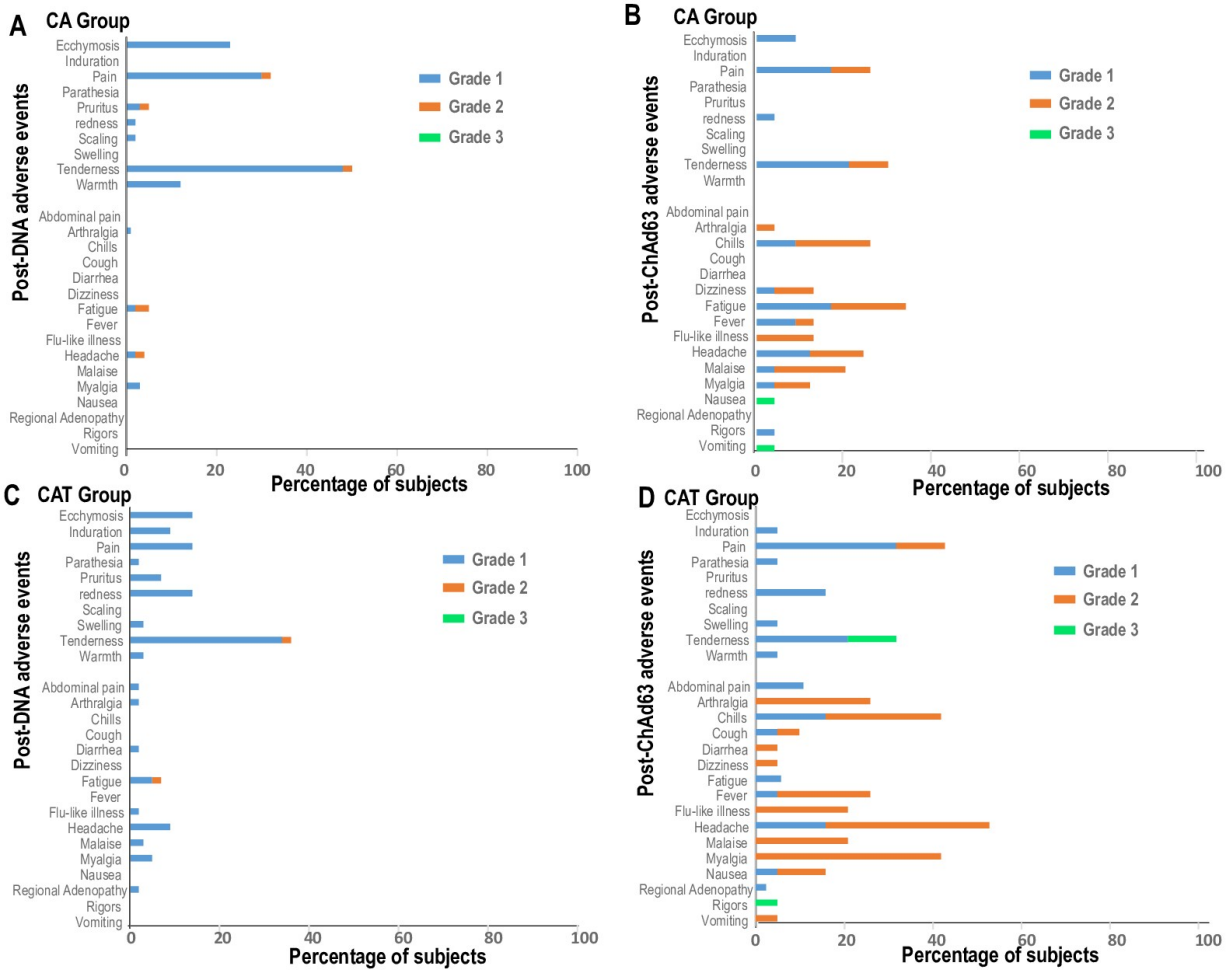
CA and CAT groups: Post-DNA and post-ChAd63 local and systemic adverse events. Horizontal bars represent percentage of subjects affected by Grade 1, 2 or 3 AEs after DNA and ChAd63 immunizations. This was calculated by dividing the total occurrences of each AE after all 3 DNA immunizations, or after the ChAd63 immunization by the total numbers of subjects that received each immunization: CA group: 60 subjects received three DNA immunizations, and 19 subjects received one ChAd63 immunization; CAT group: 58 subjects received 3 DNA immunizations, and 16 subjects received one ChAd63 immunization. Most adverse events (AEs) were mild to moderate and were similar after each DNA immunization but were more frequent after ChAd63 immunization, especially in the CAT group. Grade 3 AEs in the CA group after ChAd63 were one each of fatigue, nausea, and vomiting. However, in the CAT group Grade 3 AEs were more frequent.

Subjects in the CA and CAT groups received a total of 118 DNA and 35 ChAd63 immunizations and were all included in the safety analysis (Fig 2, and Tables 2 and 3). During the 7 days after each immunization, the total of solicited local and systemic adverse events (AEs) designated as definitely, probably, or possibly related to immunization were 185 in the CA group and 204 in the CAT group. In both groups, total numbers of local and systemic AEs were similar after the first two DNA immunizations and lower after the third DNA immunizations but were higher and affected more subjects after ChAd63 immunizations, especially after the ChAd63 CAT immunization, largely due to increased systemic AEs. All solicited AEs resolved during the 7 days follow up period.

**Table 2:** Immunized cohort CA (solicited AEs): Numbers of volunteers experiencing local, and systemic adverse events (days 0–7 post each immunization)^1,2^.

Sign or Symptom	DNA 1 (n = 20) (% of vol’s)		DNA 2 (n = 20) (% of vol’s)		DNA 3 (n = 20) (% of vol’s)		ChAd (n = 19) (% of vol’s)		Total AE’s (% of all AE’s)
	Gr1	Gr2/3	Gr1	Gr2/3	Gr1	Gr2/3	Gr1	Gr2/3	
**LOCAL**									
Ecchymosis	3(15%)	0	4(20%)	0	4(20%)	0	2(11%)^3^	0	14(14%)
Induration	0	0	0	0	0	0	0	0	0
Pain	4(20%)	0	5(25%)	1(5%)	7(35%)	0	5(26%)	2(11%)	29(28%)
Parasthesia	0	0	0	0	0	0	0	0	0
Pruritus	1(5%)	0	2(10%)	1(5%)	2(10%)	0	0	0	6(6%)
Redness	0	0	0	0	0	0	0	0	0
Scaling	0	0	0	0	1(5%)	0	0	0	1(1%)
Swelling	0	0	0	0	0	0	0	0	0
Tenderness	9(45%)	0	11(55%)	1(5%)	7(35%)	0	9(47%)	2(11%)^3^	45(44%)
Warmth	2(10%)	0	3(15%)	0	1(5%)	0	0	0	7(7%)
**Total Local AEs**	**24**	**0**	**33**	**3**	**22**	**0**	**16**	**4**	**102 (55%)**
**SYSTEMIC**									
Abdominal Pain	0	0	0	0	0	0	1(5%)	1(5%)	2(2%)
Arthralgia	1(5%)	0	0	0	0	0	1(5%)	1(5%)^3^	3(4%)
Chills	0	0	0	0	0	0	1(5%)	6(32%)^3^	8(10%)
Cough	2(10%)	0	0	1(5%)	2(10%)	0	1(5%)	0	7(8%)
Diarrhea	0	0	0	0	0	0	0	0	0
Dizziness	0	0	0	0	0	0	2(11%)	2(11%)^3^	4(5%)
Fatigue	0	1(5%)	2(10%)	1(5%)	0	0	4(21%)	6(32%)^5^	15(18%)
Fever	0	0	0	0	0	0	2(11%)	1(5%)^3^	3(4%)
Flu-like illness	0	0	0	1(5%)	1(5%)	1(5%)	1(5%)	3(16%)^3^	7(8%)
Headache	2(10%)	0	2(10%)	0	0	1(5%)	3(15%)	5(26%)^3^	14(17%)
Malaise	0	0	0	0	0	0	2(11%)	4(21%)^3^	6(7%)
Myalgia	2(10%)	1(5%)	0	0	0	0	1(5%)	3(16%)^3^	9(11%)
Nausea	1(5%)	0	0	0	0	1(5%)	0	1(5%)^4^	3(4%)
Regional adenopathy	0	0	0	0	0	0	0	0	0
Rigors	0	0	0	0	0	0	1(5%)	0	1(1%)
Vomiting	0	0	0	0	0	0	0	1(5%)^4^	1(1%)
**Total Systemic AEs**	**11**	**4**	**4**	**3**	**3**	**3**	**21**	**34**	**83(45%)**
**Total All AEs**	**35**	**4**	**37**	**6**	**25**	**3**	**37**	**38**	**185(100%)**

**Table 3:** Immunized cohort CAT (solicited AEs): Numbers of volunteers experiencing local, and systemic adverse events (days 0–7 post each immunization)^1^^,^^2^.

Sign or Symptom	DNA 1 (n = 20) (% of vol’s)		DNA 2 (n = 20) (% of vol’s)		DNA 3 (n = 18) (% of vol’s)		ChAd (n = 16) (% of vol’s)		Total AE’s (% of all AE’s)
	Gr1	Gr2/3	Gr1	Gr2/3	Gr1	Gr2/3	Gr1	Gr2/3	
**LOCAL**									
Ecchymosis	2(10%)	0	3(15%)	0	1(6%)	0	1(6%)	0	7(9%)
Induration	0	0	1(5%)	0	1(6%)	0	0	0	2(3%)
Pain	5(25%)	0	2(10%)	0	3(17%)	0	4(24%)	4(24%)^4^	20(25%)
Parasthesia	0	0	1(5%)	0	0	0	1(6%)	0	2(3%)
Pruritus	1(5%)	0	3(15%)	0	0	0	0	0	4(5%)
Redness	0	0	0	0	1(6%)	0	3(18%)	0	4(5%)
Scaling	0	0	0	0	0	0	0	0	0
Swelling	0	0	0	0	0	0	2(12%)	0	2(3%)
Tenderness	10(50%)	1(5%)	4(20%)	0	7(39%)	0	5(29%)	3(18%)^4^	34(43%)
Warmth	1(5%)	0	1(5%)	0	0	0	2(12%)	0	4(5%)
**Total Local AEs**	**21**	**1**	**18**	**0**	**13**	**0**	**19**	**7**	**79(39%)**
**SYSTEMIC**									
Abdominal Pain	1(5%)	0	0	0	0	0	2(12%)	0	3(2%)
Arthralgia	0	0	1(5%)	0	0	0	0	6(35%)^2^	8(6%)
Chills	0	0	1(5%)	0	0	0	3(18%)	7(41%)^2^^,^^6^	13(10%)
Cough	0	0	1(5%)	0	1(6%)	0	1(6%)	2(29)^3^	5(4%)
Diarrhea	1(5%)	0	0	0	0	0	0	1(6%)^3^	2(2%)
Dizziness	0	0	0	0	0	0	0	1(6%)^3^	1(1%)
Fatigue	2(10%)	0	2(10%)	1(5%)	2(11%)	0	0	6(35%)^5^	15(12%)
Fever	0	0	1(5%)	0	0	0	1(6%)	5(29%)^5^	8(6%)
Flu-like illness	1(5%)	0	0	0	0	0	0	5(29%)^5^	7(6%)
Headache	4(20%)	0	1(5%)	0	2(11%)	0	3(18%)	8(47%)^5^	21(17%)
Malaise	1(5%)	0	1(5%)	0	2(11%)	0	0	6(35%)^5^	11(9%)
Myalgia	2(10%)	0	2(10%)	0	0	0	0	10(59%)^5^	15(12%)
Nausea	0	0	1(5%)	0	0	0	1(6%)	2(12%)^3^	5(4%)
Regional adenopathy	1(5%)	0	1(5%)	0	0	0	1(6%)	0	3(2%)
Rigors	0	0	0	0	0	0	1(6%)	2(12%)^4^	4(3%)
Vomiting	0	0	0	0	0	1(6%)	1(6%)	1(6%)^3^	4(3%)
**Total Systemic AEs**	**13**	**0**	**14**	**4**	**7**	**1**	**15**	**71**	**125 (61%)**
**Total All AEs**	**34**	**1**	**32**	**4**	**20**	**1**	**34**	**78**	**204 (100%)**

Solicited local and systemic adverse events were assessed starting on day of immunization (following receipt of vaccine) through 7 days post-immunization (Day 7). **AEs: Adverse Events; vols (volunteers); Gr (Grade)** Severity classification for signs and symptoms: Gr1 = adverse event does not interfere with daily activities; Gr2 = interferes with but does not prevent daily activities; Gr3 = prevents daily activities. All adverse events in the table are Gr1 (mild) unless noted otherwise.

^1^The local and systemic AEs are tabulated by subjects experiencing the AE according to their highest grade recorded in days 0–7 For those that experienced multiple events for a specific AE at a specific dose, the most severe event was used.

^2^The total AE’s is the total number of all adverse events (i.e., subjects may have exhibited the same AE at a specific dose more than once).

^3^ = Gr2 (moderate);

^4^ = Gr3 (severe);

^5^ = both Gr2 and Gr3.

All local adverse events occurred in the arm ipsilateral to the injection site.

^6^ Includes an isolated grade 4 event (resolved).

*Post-DNA immunizations*. Local Grade 1 and Grade 2/3 AEs in the CA group (total 82, 1.37/subject) were similar (not statistically different) to those in the CAT group (total 53, 0.91/subject), due to increased frequency of tenderness and pain at the vaccination site. Most AEs were mild (Grade 1) with fewer moderate (Grade 2); none were above Grade 2. The most frequent local AEs were tenderness, pain, and ecchymosis at the injection site. Systemic Grade 1 and Grade 2 AEs in the CA group (total 28, 0.47/subject) were similar to the CAT group (total 39, 0.70/subject). In both groups, the most frequent systemic AEs were fatigue and headache. Local and systemic AEs were similar to those post-DNA AEs in the DNA/HuAd5 trial [6].

*Post-ChAd63 immunizations*. The incidence of local and systemic AEs was similar in both groups: local AEs (CA 11%; CAT; 13%), systemic AEs (CA 30%; CAT 42%). The most frequent local AEs were tenderness and pain at the injection site. The most frequent systemic AEs were fatigue, fever, chills, and malaise. These frequencies were similar or lower to those reported using ChAd63-ME-TRAP as the priming dose followed by MVA -ME-TRAP boost [7]. The number of severe systemic (Grade 3) AEs in the CAT group (21, 13% all AEs) appeared more frequent compared to the CA group (3, 2%) (Fig 2). Severe AEs in the CA group were one each of fatigue, nausea and vomiting; in the CAT group these were myalgia (5), chills (4), arthralgia (2), fatigue (2), flu-like illness (2), headache (2), malaise (2), rigors (1), fever (1).

The exact McNemar’s test showed that the rate of local AEs was significantly higher after the second DNA vaccination than that after the ChAd63 boost vaccination in the CA group (p = 0.0129), with a non-significant difference between the first and third DNA vaccination and ChAd63 boosting vaccination (p = 0.5811 and 0.0963, respectively). There were no significance differences in the rates of local AEs between each DNA vaccination and the ChAd63 boost vaccination in the CAT group (p = 0.5488, 0.6072, and 0.5811, respectively). The rates of systemic AEs were significantly higher after the ChAd63 boost vaccination compared to each of the three DNA vaccinations in both the CA group (p = 0.0074, 0.0034, and 0.001, respectively), and the CAT group (p = 0.0018, 0.0018, and 0.0001, respectively). There were no significant differences of local or systemic AEs, or both, between the CA and CAT groups after the DNA or ChAd63 immunizations.

#### Solicited AE’s post-CHMI

Finally, although not an *a priori* objective, in a post hoc analyses we assessed for differences in the rates of malaria associated AEs between the vaccines and infectivity controls to assess for any disease modifying effects of vaccination. In all significance testing that follows we employed the unpaired two-tailed t-test with normality assumptions satisfied. We observed similar rates of AEs (mean/standard deviation) [5.9 (4.4) in vaccinated and 7.1 (4.3) in infectivity controls (p = 0.45)]. Using limiting analysis to those who were parasitemic, the rates of AEs were 6.9 (4.1) and 6.7 (4.3) respectively in vaccinated and controls (p = 0.9). This is consistent with the non-significant difference in time to parasitemia in subjects who exhibited parasitemia and suggests that the vaccine did not alter the clinical expression of blood stage infection.

#### Laboratory AEs

There were few laboratory AEs related to immunization, as in the DNA/HuAd5 trial (17). The numbers of laboratory AEs after ChAd63 immunizations were not significantly different in both the CA (15) and CAT (11) groups compared to those after DNA immunizations (S5 Table in S2 File).

#### Unsolicited AEs

Three possibly vaccine-related Grade 1 AEs were reported in the CA group, one after the third DNA immunization (ecchymosis not at injection site) and two systemic AEs post-ChAd63 (S6 Table in S2 File). Two possibly vaccine-related Grade 2 systemic AEs post-ChAd63 were reported in the CAT group (S6 Table in S2 File). All resolved rapidly without sequelae.

### Vaccine efficacy (VE)

**Fig 3:**
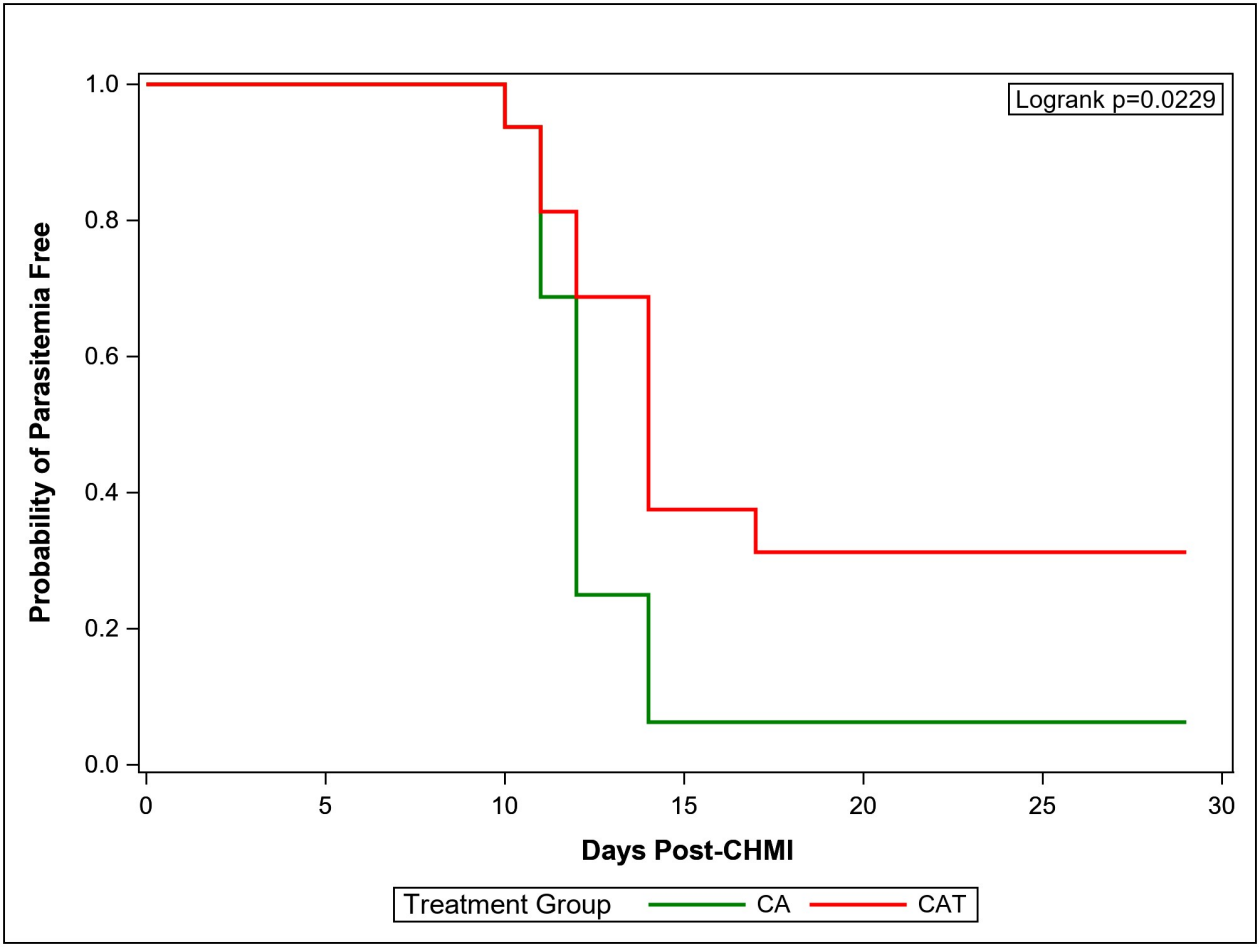
Kaplan-Meier curve (CAT and CA). K-M curves depicting the CAT and CA groups.

**Fig 4:**
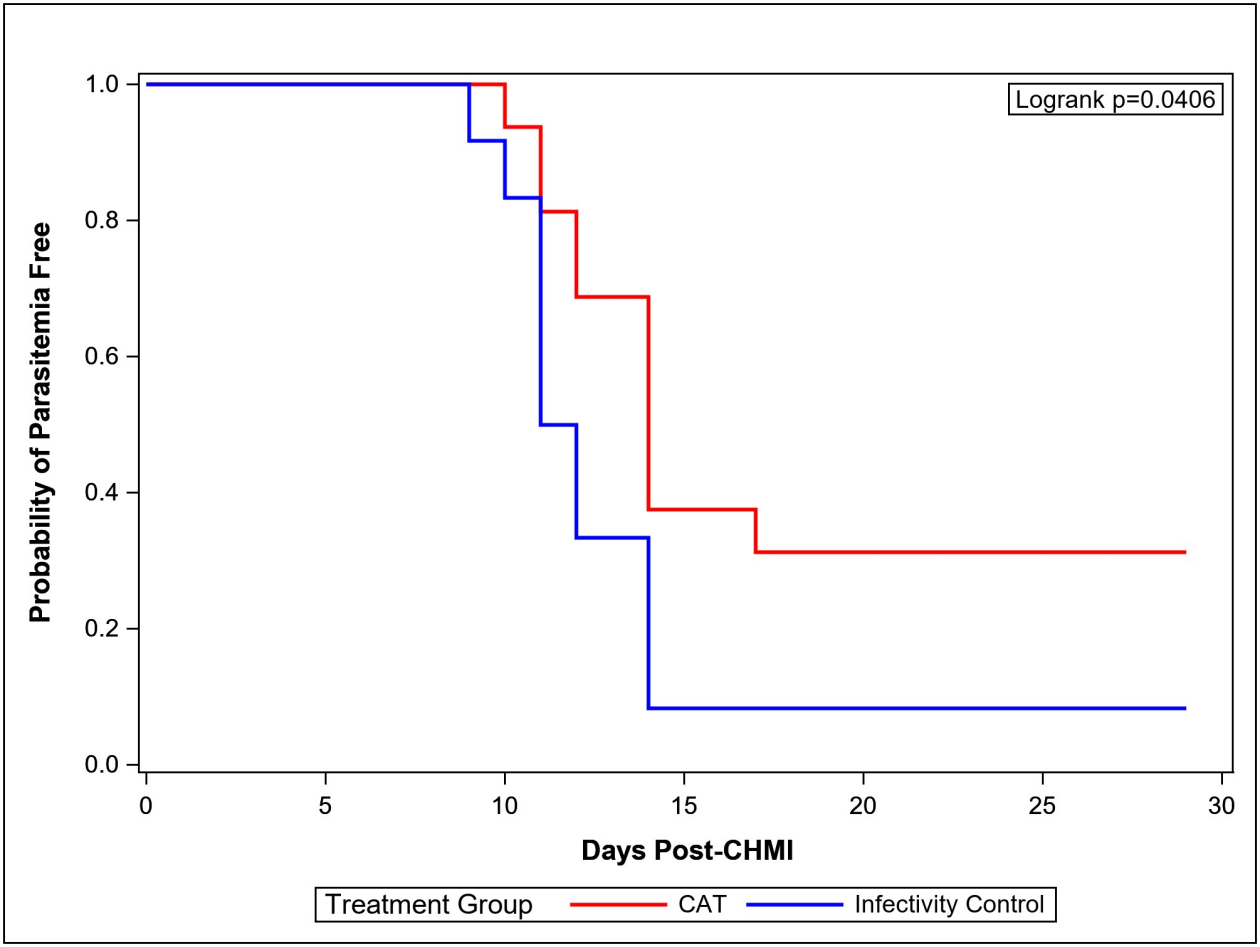
Kaplan-Meier curve [CAT and infectivity controls (IC)]. K-M curves depicting the CAT and infectivity controls (IC) group. In both K-M curves data was censored at day 28. Parasitemia was based on microscopic examination of peripheral blood smears.

Eleven of 12 infectivity controls developed parasitemia detected by blood smear between days 9 and 14 (mean 11.7 days, standard deviation 1.7 days), comparable to previous trials [4, 6, 38]; the twelfth control remained parasite-free and was treated presumptively on day 29 post CHMI. In the CA group, one of 16 (6%) immunized subjects did not develop parasitemia during 28 days of follow-up (Table 4; Fig 3). The remaining 15 subjects became parasitemic between days 10 to 17 (mean 12.0 days), a similar prepatent period to that of the DNA/HuAd5 CA trial [6]. In the CAT group, 5 of 16 (31%) immunized subjects did not develop parasitemia, and the remaining 11 subjects became parasitemic between days 11 to 17 (mean 12.9 days), an interval similar to that seen in the parasitemic subjects in the CA and infectivity control groups (Table 4, Fig 4). There was statistically significant protection in the CAT group compared to the infectivity controls (p = 0.0406) (Fig 4) and to the CA group (p = 0.0229) (log rank test) (Fig 3), whereas the CA group was not different from the controls. There were no significant differences in the three groups in time to parasitemia in those subjects that developed parasitemia (Mann-Whitney test).

**Table 4:** Microscopic smear results through day 28 post-CHMI.

	CA (N = 16)	CAT (N = 16)	Infectivity Control (N = 12)
Positive Blood Smears, n (%)	15 (94%)	11 (69%)	11 (92%)
Negative Blood Smears, n (%)	1 (6%)	5 (31%)	1 (8%)
Time to 1st Positive Smear (Days)	10.1, 10.9, 10.9, 10.9, 11.2, 11.8, 11.9, 11.9, 11.9, 12.0, 12.0, 12.5, 13.8, 13.9, 14.0	10.0, 10.9, 11.1, 11.8, 12.5, 13.4, 13.8, 13.9, 14.0, 14.5, 17.0	8.8, 9.9, 10.9, 10.9, 10.9, 11.0, 11.9, 11.9, 13.9. 13.9, 13.8
Mean (SD)	11.98 (1.16)	12.98 (1.98)	11.62 (1.68)
Median	12.99	13.42	11
Vaccine Efficacy (VE), % (95% CI)	-2% (-26.5–17.3)	25% (-8.8–48.3)	NA
	p-value: 0.8352	p-value: 0.0746	NA

VE = (1—Relative Risk).

P-values were calculated by Cochran-Mantel-Haenszel method.

CI: Confidence Interval.

VEs for each vaccine group calculated as 1 minus the risk ratio are shown in Table 4. VE in the CAT group was 25% (95% CIs: -8.8 to 48.3, p-value: 0.0746] and in the CA group was -2% (95% CIs: -26.5 to 17.3, p = 0.835).

### Immunogenicity

#### Enzyme-linked immunosorbent assay (ELISA)

**Fig 5:**
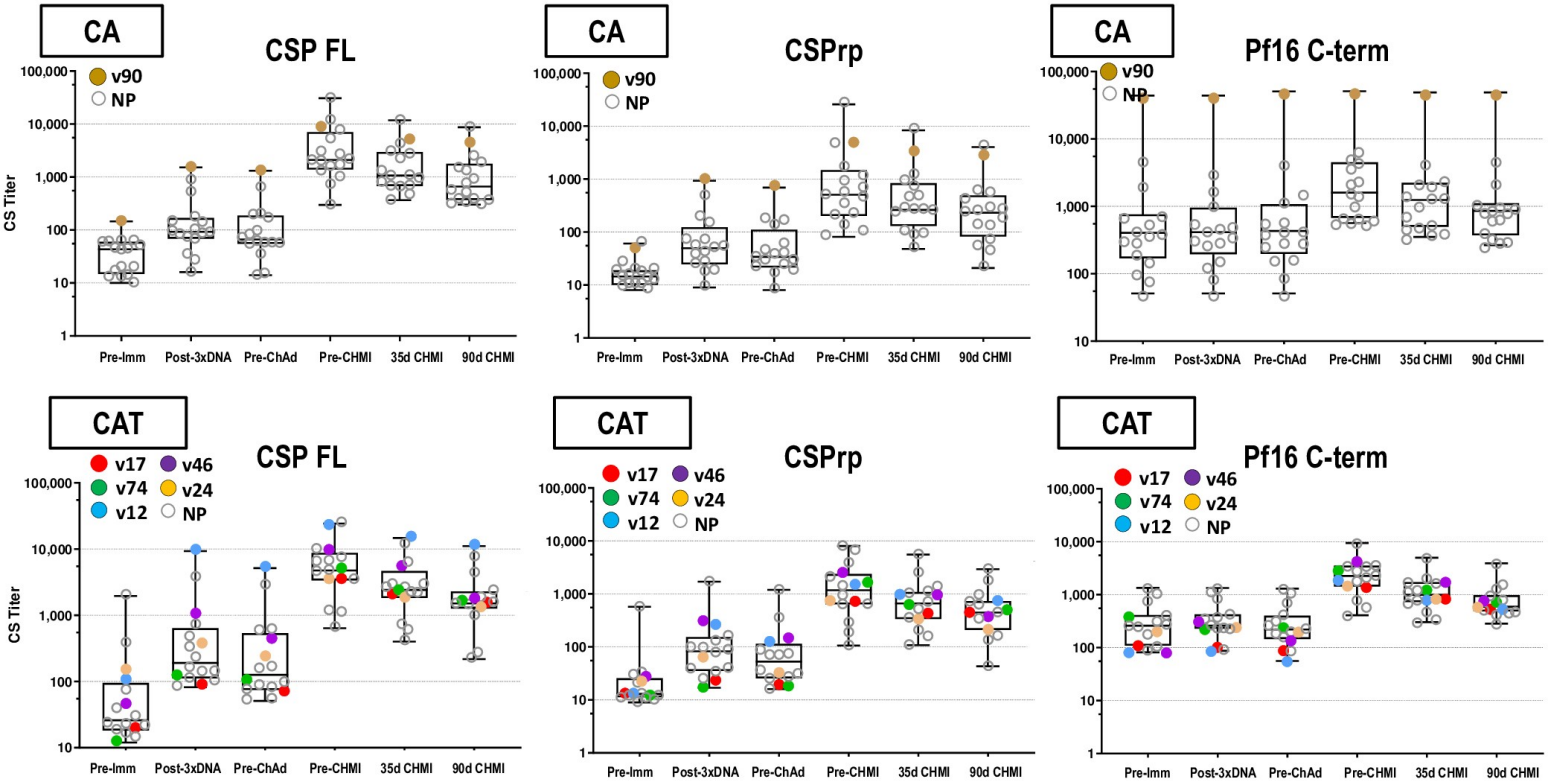
CA and CAT cohorts: ELISA antibody responses to CSP. The box plots (see Statistical Analysis section for description) represent anti-CSP by ELISA for all challenged subjects. The time points on the x-axis are described in Methods. Protected subjects are shown as larger, color-coded dots.

**Fig 6:**
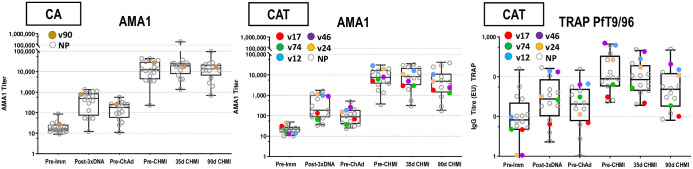
CA and CAT cohorts: ELISA antibody responses to AMA1 and TRAP. The box plots (see Statistical Analysis section for description) represent anti-AMA1 titers and anti-TRAP activities by ELISA for all challenged subjects. The time points on the x-axis are described in Methods. Protected subjects are shown as larger, color-coded dots.

CSP: Post-DNA geometric mean CA and CAT responses to full length CSP (CSPFL) and CSP repeat region (CSPrp) were low (Fig 5, S7-S9 Tables in S2 File), significantly rose post-ChAd63, were similar in both groups; and significantly dropped by 90 days post-CHMI. One protected subject in the CA group had high pre-immunization activities to the CSP terminal region (CSPf16), but not CSPFL or CSPrp, that were unchanged post-DNA and post-ChAd63, and its significance is unknown. AMA1: post-DNA geometric mean CA and CAT responses were low (Fig 6; S10 Table in S2 File), significantly rose post-ChAd63 and dropped non-significantly by 90 days post-CHMI. TRAP: post-DNA geometric mean CAT IgG EU titers were low (Fig 4, S11 Table in S2 File), significantly rose post-ChAd63 and significantly dropped by 90 days post-CHMI (Fig 6; S11 Table in S2 File). Post-ChAd63 responses of the CA and CAT groups to CSP and AMA1 were statistically similar (Mann-Whitney U Test), suggesting that the addition of TRAP to the CAT group did not cause immune interference with these responses.

#### Immunofluorescence antibody assay (IFA)

**Fig 7:**
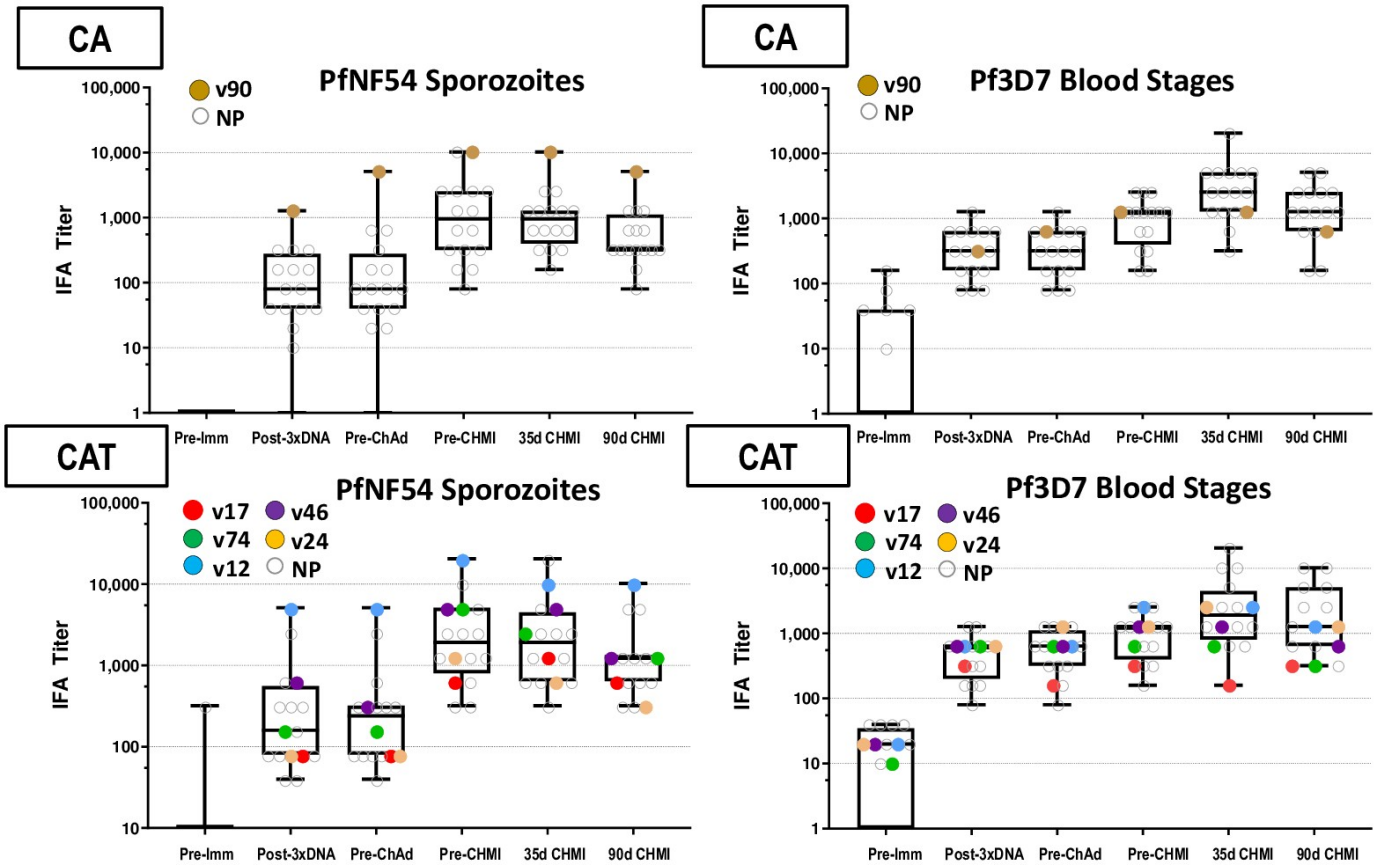
Antibody responses by IFA to PfNF54 sporozoites and Pf3D7 blood stages. The box plots represent responses to PfNF54 sporozoites and Pf3D7 blood stages. Responses of protected subjects are color-coded; not protected (NP) subjects are open circles.

*Sporozoites*. Post-DNA geometric mean CA and CAT responses to PfNF54 sporozoites were low (Fig 7; S12 Table in S2 File), significantly rose post-ChAd63, and were higher (but not significantly) in the CAT group than the CA group pre-CHMI. CA responses significantly and CAT responses non-significantly dropped by 90 days post-CHMI.

*Blood stages*. Post-DNA geometric mean CA and CAT responses to Pf3D7 blood stages were low (Fig 7; S10 Table in S2 File) and non-significantly rose post-ChAd63; responses significantly rose by 35 days post-CHMI due to increases only in non-protected subjects likely derived from transient blood stages expressing AMA1 (Fig 7; S13 Table in S2 File).

Using the Accelerated Failure Time model, there were no statistical associations (p = >0.05) between ELISA (CSPFL, CSPrp, CSPF16, AMA1, and TRAP) or IFA (sporozoite and blood stage) antibody levels and VE, similar to the lack of association between antibody levels and VE for CSP and AMA1 in the DNA/HuAd5 CSP/AMA1 trial [6]. Two protected subjects in the CAT group (v12, v24) exhibited the highest post-ChAd63/pre-CHMI ELISA responses to AMA1, and v12 exhibited the highest responses in IFA to both sporozoites and blood stages.

### Cellular *ex vivo* FluoroSpot IFN-γ activities to CSP, AMA1 and TRAP

#### CA group

**Fig 8:**
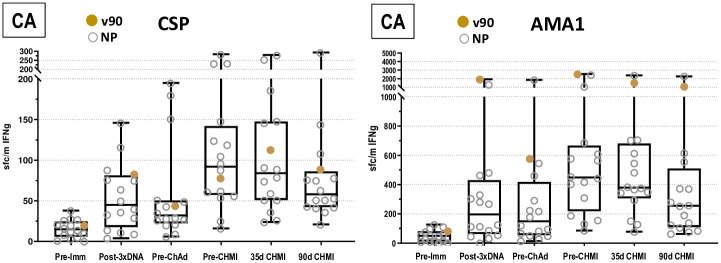
CA cohort: IFN-γ FluoroSpot responses to CSP and AMA1. *Ex-vivo* T-cell activities by FluoroSpot Assay for CSP and AMA1. The box plots represent CSP and AMA1. Summed IFN-γ T-cell responses against peptide pools are spot forming cells per million PBMCs for all challenged subjects.

*CSP pooled peptides*. Post-DNA geometric mean responses (Fig 8; S14 Table in S2 File) were similar in both CA and CAT groups, non-significantly rose and dropped pre-ChAd63, and significantly rose post-ChAd63. Responses post-ChAd63 were similar in both groups but more subjects were positive in the CAT group. Responses significantly dropped by 90 days post CHMI. Numbers of positive subjects rose post-DNA prime (3 doses), declined pre-ChAd63, rose post-ChAd63 (10/16, 63%), and declined 90 days post-CHMI.

*AMA1 pooled peptides*. Post-DNA prime geometric mean responses (Fig 8; S15 Table in S2 File) significantly rose, were higher than CSP, were similar in both CA and CAT groups, dropped pre-ChAd63, significantly rose post-ChAd63 and dropped by 90 days post CHMI. The numbers of subjects with positive responses were higher than CSP at all time points, especially post-ChAd63 (16/16, 100%).

All subjects had post-ChAd63/pre-CHMI responses to one or both antigens, but these were low (<100 sfc/m) in 6/16 (38%) subjects. The single protected subject (v90) had the highest responses to AMA1 at all time points, particularly post-ChAd63 (2549 sfc/m).

#### CAT group

**Fig 9:**
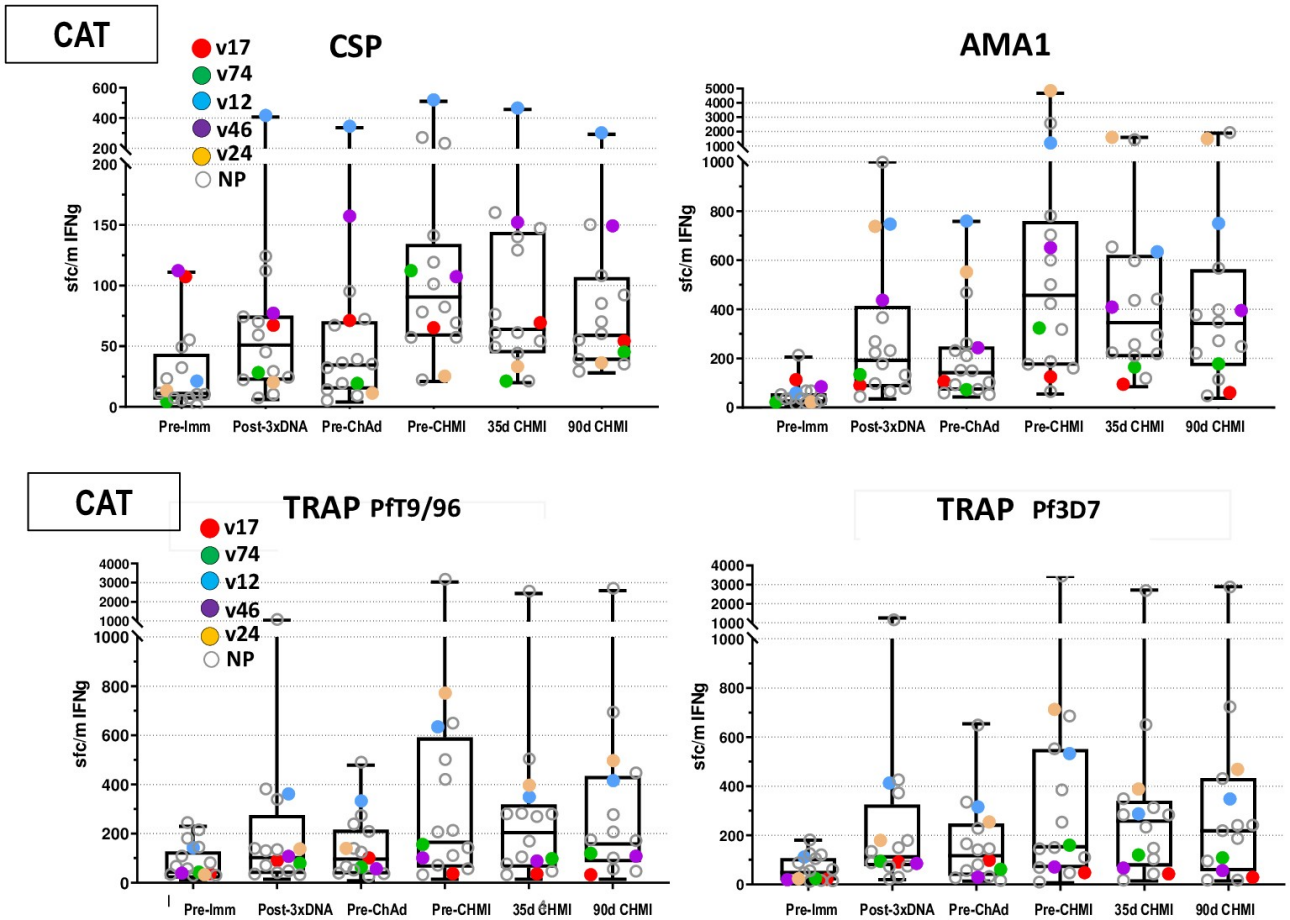
CAT cohort: IFN-γ FluoroSpot responses to CSP, AMA1 and TRAP. *Ex-vivo* T-cell activities by FluoroSpot Assay for CSP, AMA1 and TRAP. The box plots represent CSP, AMA1 and TRAP (Pf9/96 and Pf3D7). Summed IFN-γ T-cell responses against peptide pools are spot forming cells per million PBMCs for all challenged subjects.

*CSP pooled peptides*. Geometric mean responses were like the CA group; post-DNA prime geometric mean responses were low (Fig 9; S14 Table in S2 File), but more subjects had positive responses than the CA group; responses significantly rose post-ChAd63 and fell by 90 days post-CHMI. Numbers of positive post-ChAd63 (12/16, 75%) subjects were slightly higher than the CA group.

*AMA1 pooled peptides*. Geometric mean responses were also like the CA group; post-DNA prime geometric mean responses (Fig 9; S15 Table in S2 File) were low, but more subjects had positive responses in the CAT group, significantly rose post-ChAd63; and declined non-significantly by 90 days post-CHMI. As in the CA group, numbers of subjects with positive responses to AMA1 were higher than CSP, post-ChAd63 (15/16, 94%).

*TRAP pooled peptides*. The DNA-T vaccine contained the Pf3D7 sequence, whereas the ChAd63-T vaccine contained the PfT9/96 sequence. Responses to 3D7 TRAP and T9/96 TRAP were measured after the DNA and ChAd63 immunizations. Geometric mean responses and numbers of subjects with positive responses to 3D7 TRAP (Fig 6B; S16 Table in S2 File) were like T9/96 TRAP at all time points.

*Responses to T9/96*. Post-DNA prime geometric mean responses (Fig 9; S16 Table in S2 File) remained low, rose significantly post-ChAd63, and were almost unchanged post-CHMI. Numbers of subjects with positive responses rose post-DNA, rose further post-ChAd63 (12/16, 75%), and were almost unchanged post-CHMI.

*Responses to 3D7*. Post-DNA prime geometric mean responses (Fig 9; S16 Table in S2 File) remained low, rose significantly post-ChAd63 as observed with T9/96, and were almost unchanged post-CHMI. Numbers of positive subjects were also like Pf T9/96, including post-ChAd63 (13/16, 81%).

One protected subject in the CAT group, v12, had the highest responses to CSP at all time points including pre-CHMI. A second protected subject, v24, had the highest responses to AMA1 at pre-CHMI. Protected subject v24 also had high responses to T9/96 and 3D7 TRAP, but one non-protected subject had much higher responses.

There was no statistical association between FluoroSpot responses to CSP, AMA1 and TRAP and VE (Accelerated Failure Time model, p = >0.05), mirroring the lack of association of antibody levels, and unlike our previous DNA/HuAd5 CA trial [6] where there was an association between ELISpot responses to AMA1, but not CSP, and protection. The accelerated time to failure model did not identify significant differences in cellular and antibody responses controlling for the age variations shown in Table 1.

There were no significant differences in responses to CSP and AMA1 at any time point in the CA and CAT group (using Mann-Whitney), and we conclude there was no evidence of immune interference when TRAP was added to CSP and AMA1.

### Effect of Nab responses to HuAd5 on immunogenicity

Geometric mean Nab titers to HuAd5 (S17 Table in S2 File) before and after ChAd63 were statistically similar (p = 0.36), confirming that cross reaction between HuAd5 and ChAd63 is low or absent [14]. Nabs to HuAd5 did not show significant correlations with any immune measure (S18 Table in S2 File). In addition, there was no association between Nab titers prior to ChAd63 boosts and protection (S3 Fig in S2 File), unlike the DNA/HuAd5 trial where all protected subjects had low (<1:500) Nab responses to HuAd5 [6].

## Discussion

Our hypothesis for this trial was that adding a third antigen, ME-TRAP (CAT), to our original two antigen (CA) formulation would increase efficacy, and this was achieved. The CAT formulation provided significant protection against CHMI (p = 0.0406) compared to controls, with a VE of 25%. The CAT group was significantly protective relative to the CA group as well (p = 0.0229). Surprisingly, the CA formulation did not provide protection relative to the controls, with a VE of -2%. In addition, there were no significant differences in time to parasitemia in the parasitemic subjects in the three groups, indicating that vaccination had not affected parasite multiplication rates in the blood. Others have suggested [41] that a delay to parasitemia is related to the per cent reduction in liver stage parasites and is therefore another indicator of vaccine effectiveness. This suggests that protection in the CAT group appeared to be based entirely on successfully eliminating the pre-erythrocytic (sporozoite and liver) stages of the parasite. This is consistent with the proposed mechanism of protection, CD8+ T cells recognizing parasite antigens expressed on the surface of infected hepatocytes.

VE at the group level was not significantly associated with FluoroSpot IFN-γ responses to CSP, AMA1 or TRAP, unlike our DNA/HuAd5 CA trial where we found a significant association with ELISpot IFN-γ responses to AMA1, though not CSP. In the earlier ChAd63/MVA ME-TRAP trial conducted in the UK, VE was associated with CD8+ T cells secreting IFN-γ, though not ELISpot IFN-γ [17]. In the current trial, however, we generally observed comparable overall geometric mean immune responses (ELISA, IFA, FluoroSpot) in the CA and CAT groups, despite differences in VE.

Detailed examination of individual subjects may prove more revealing. In the prior DNA/HuAd5 CA trial [10], three of the four protected subjects had the highest post-HuAd5/pre-CHMI ELISpot and CD8+ T cell IFN-γ activities to either CSP or AMA1, or both antigens, and one protected subject had the highest anti-AMA1 antibody concentration. In the current DNA/ChAd63 trial, we found that the single protected subject in the CA group had the highest post-ChAd63/pre-CHMI responses to AMA1, and two protected subjects in the CAT group had the highest responses to CSP or AMA1, with one also showing high responses to TRAP. However, three of the five protected subjects in the CAT group did not have noticeably higher FluoroSpot IFN-γ responses to any antigen. Therefore, we are planning to determine the genetic restriction of responses to these antigens that may reveal differences between protected and non-protected subjects. It is also possible that protective mechanisms in these subjects may not be measured by FluoroSpot. In the DNA/HuAd5 trial, we identified CD4+ responses to CSP in one protected subject that did not have elevated ELISpot responses. We plan to use other methods, including flow cytometry/intracellular cytokine staining, to better investigate their responses.

Therefore, based upon our prior clinical evidence with the DNA/HuAd5 vaccine that that protection involved HLA-restricted class-I epitopes, we will investigate whether recognition of Class I-restricted epitopes in CSP, AMA1 or TRAP are associated with protection, via prediction of the binding affinities of CSP, AMA-1 and TRAP epitopes using the NetMHCpan algorithm followed by confirmation of activity using multi-parameter FluoroSpot to analyze cellular IFN-γ responses [10].

The absence of protection in the CA group was surprising, as the two-antigen formulation had been protective in the prior trial. Replacing HuAd5 with ChAd63 may have adversely affected immunogenicity, possibly reflecting differences in the level of innate immunity triggered by the two different adenovirus vectors, with resulting effects on acquired, antigen-specific T cell responses [42–44]. We also considered differences in the antigenic inserts in the recombinant DNA plasmids and ChAd63 vectors, which were not identical, unlike the DNA/HuAd5 trial. We used the same DNA plasmids as before; however, the ChAd63 vectors were provided by the University of Oxford and had minor differences compared to the DNA plasmids affecting the size of amino acid deletions in the repeat and C-terminal regions and the addition of an FVO allele in the ChAd63 AMA1 insert. However, post-DNA and post-ChAd63 antibody responses were similarly low using the 3D7 allele as the ELISA capture antigen.

There were differences in the kinetics of FluoroSpot responses in the DNA/ChAd63 trial and the kinetics of ELISpot responses in the DNA/HuAd5 trial. The FluoroSpot assay was adapted from the ELISpot assay, but batches of peptides were manufactured separately, and used slightly different capture plates (see Methods). We generally observed that overall geometric mean FluoroSpot responses in the CA and CAT groups were low after DNA immunizations but were significantly boosted by the ChAd63 immunizations. In the DNA/HuAd5 trial, post-DNA and post-HuAd5 ELISpot responses were similar and not significantly different than pre-immunization responses. However, pre-immunization ELISpot responses measured in the DNA/HuAd5 trial [6] were higher than pre-immunization FluoroSpot responses measured in the DNA/ChAd63 trial and may have biased the interpretation of post-immunization boosting responses. Geometric mean post-DNA and post-ChAd63 FluoroSpot responses and post-HuAd5 ELISpot responses were similar, but more subjects in the DNA/ChAd63 trial had positive FluoroSpot responses to CSP and AMA1 in the CA, and CAT groups; this was particularly the case of post-DNA responses, suggesting that re-manufacturing the DNA plasmids did not adversely affect immunogenicity. In addition, the range of FluoroSpot responses to TRAP (3D7 and T9/96) were like those reported previously [17]. We also found that there was no antigenic competition when TRAP was added to the two-antigen CA vaccine. Therefore, we suggest that differences in VE between the DNA/HuAd5 CA trial (27%) and the DNA/ChAd63 CA trial are probably not related to potency of the vaccines, but rather differences in HLA alleles expressed by subjects in both trials. This subunit vaccine approach requires optimization of HLA-restricted CD8+ T cell responses. Therefore, the optimal approach to these vaccines requires integrating genetically HLA restricted epitopes in different antigens to ensure sufficient coverage in a genetically-diverse population.

Local and systemic AEs after DNA immunizations were similar between groups and those in the DNA/HuAd5 trial [6]. However, after the ChAd63 boost, there was an increase in both the number and severity of AEs in the CAT group as compared to the CA group and as compared to AEs after the HuAd5 boost [6]. Prior dosing regimens used the ChAd63 vector at ranges between 1x10^8^ to 2x10^11^ vp and the median numbers of AEs increased with dose [7]. The dose used in the CAT group (1.5x10^11^ vp) was higher than the CA group (1.0x10^11^ vp) and is the most likely associated with the observed increase in AEs. Vaccine-induced immune thrombotic thrombocytopenia (VITT) has been observed after widespread use of the COVID19 ChAd0x1 vaccine [45], but this study used ChAd63 and there have been no reports of VITT in clinical trials using this vector, although their future use may require scrutiny for VITT.

The levels of pre-existing Nabs to HuAd5 were low (<1:500) in most subjects in the CA and CAT groups and were not significantly boosted by the ChAd63 vaccine, confirming an absence of cross-reactivity between HuAd5 and ChAd63. There was no association between Nabs and effects on pre- and post-ChAd63 immune responses or with VE, demonstrating that replacing the HuAd5 vector with ChAd63 effectively eliminated the concerns of potential deleterious effects of pre-existing Nabs observed in the DNA/HuAd5 trial [6].

How could the CAT vaccine be further improved? Since HLA-restricted responses are crucial in determining protection using gene-based vaccines, we could rapidly screen sequences of other antigens for possible protective epitopes and identify other antigens that could be added to the CAT vaccine. We have previously used this approach of predicting T cell epitopes that were confirmed in T cell assays for malaria epitopes [46]. It is also being used to successfully identify SARS-CoV-2 virus T cell specific peptides that were tested using PBMCs from COVID-19 convalescent or acute respiratory distress syndrome (ARDS) patients [47] or SARS [48, 49].

Prime-boost vaccines based on DNA and chimpanzee adenovirus constructs designed to elicit protective T cell responses are being actively developed for a variety of cancers [50] and infectious diseases including COVID-19 [51], Ebola [52–55], influenza [56], rift valley fever [57], hepatitis C [58], Chikungunya [59], MERS [60] and HIV-1 [61]. It should be possible to do the same for malaria, albeit the parasite has a much larger proteome and complexity of life cycle, and a higher number of antigens may need to be included. We suggest that subunit malaria vaccine approaches allow targeted immunization by confirming protective HLA-restricted Class I epitopes that elicit host protective immunity.

### Limitations

This study is limited by the small sample size. Future studies should aim to replicate these findings with a large sample size and well-balanced baseline characteristics. T cell IFN-γ responses were low or negative in many non-protected subjects probably due to genetic-restriction of responses to these three antigens, indicating that the addition of more antigens to broaden HLA-coverage may increase protection. Identification of suitable antigens may require screening of matched HLA-restricted epitopes to increase vaccine coverage of diverse HLA alleles.

## Supporting information

S1 Checklist CONSORT checklist. (DOCX)

S1 File (DOCX)

S2 File (DOCX)
