# Supplementary material for: A three-antigen Plasmodium falciparum DNA prime—Adenovirus boost malaria vaccine regimen is superior to a two-antigen regimen and protects against controlled human malaria infection in healthy malaria-naïve adults
Source: PLoS One. 2021 Sep 8;16(9):e0256980. doi: 10.1371/journal.pone.0256980 (PMC8425539; doi:10.1371/journal.pone.0256980)
Supplement: S1 File — (DOCX) [file pone.0256980.s002.docx]

Title Page

**A Phase 1 Trial with Controlled Human Malaria Infection to Evaluate Safety, Immunogenicity, and Protective Efficacy of Two-Antigen and Three-Antigen *Plasmodium falciparum* DNA Prime- Adenovirus Boost Malaria Vaccine Regimens in Healthy Malaria Naïve Adults**

| Sponsor | The Surgeon General, Department of the Army | |
| --- | --- | --- |
| **Sponsor’s Representative** | Mark S. Paxton, JD, MS  US Army Medical Research and Development Command (USAMRDC) Office of Regulated Activities (ORA)  1430 Veterans Drive  Fort Detrick, Maryland 21702-5009  Telephone: 301-619-0317; DSN: 343-0317  Fax: 301-619-0197  Email: usarmy.detrick.medcom-usamrmc.mbx.regulatory-affairs@mail.mil | |
| **Primary Research Monitor** | Charmagne G. Beckett, MD, MPH, FACP, CAPT, MC, USN  Naval Medical Research Center  Infectious Diseases Directorate  503 Robert Grant Ave  Silver Spring, Maryland 20910  Office: 301-319-7662  Cell: 301-919-2350  Email: [charmagne.g.beckett.mil@mail.mil](mailto:charmagne.g.beckett.mil@mail.mil) | |
| **Secondary Research Monitor** | Alexandra Singer, MD, LCDR, MC, USN  Defense Health Agency  J3/Public Health  7700 Arlington Blvd, Suite 3M348A  Falls Church, VA 22042-5143  Office: (703) 681-6866  Cell: (803) 553-2447  Email: alexandra.l.singer.mil@mail.mil | |
| **Principal Investigator (Qualified Physician Responsible for All Trial-Site-Related Medical Decisions)** | Christopher Duplessis, MD, MPH, CDR, MC, USN  Naval Medical Research Center  503 Robert Grant Avenue  Silver Spring, Maryland 20910-7500  Telephone: 301-295-0021  Cell: 240-778-7268  Email: christopher.a.duplessis.mil@mail.mil | |
| **Clinical Investigators** | Judith Epstein, MD  National Institutes of Health (NIH)  9000 Rockville Pike, Bethesda, Maryland 20892  Telephone: 240-281-6331  Email: judithepsteinmd@gmail.com | |
|  |  | |
|  | Ilin Chuang, MD, CAPT, MC, USN  United States Military Malaria Vaccine Program  Naval Medical Research Center  503 Robert Grant Avenue  Silver Spring, Maryland 20910-7500  Telephone: 301-319-9786  Fax: 301-319-7545  Email: Ilin.chuang.mil@mail.mil | |
|  |  | |
|  | Karine M. Hollis-Perry, MD, CDR, MC, USN  Naval Medical Research Center  Director, NMRC Clinical Trials Center  503 Robert Grant Avenue  Silver Spring, Maryland 20910-7500  Telephone: 301-295-0007  Email: karine.m.hollisperry.mil@mail.mil | |
|  | Marvin J. Sklar, MD, CDR, MC, USN  Head of Clinical Studies  U.S. Naval Medical Research Unit-2  #2 Samdach Penn Nouth Street, Khan Tuol Kork,  Phnom Penh, Cambodia  Telephone: +855-23-728-000  Email: marvin.sklar.mil@namru2.org.kh | |
| **Research Investigators** | Martha Sedegah, PhD  United States Military Malaria Vaccine Program  Naval Medical Research Center  503 Robert Grant Avenue  Silver Spring, Maryland 20910-7500  Telephone: 301-319-7586  Fax: 301-319-7545  Email: Martha.sedegah.civ@mail.mil | |
|  | Eileen D. Franke Villasante, PhD  United States Military Malaria Vaccine Program  Naval Medical Research Center  503 Robert Grant Avenue  Silver Spring, Maryland 20910-7500  Telephone: 301-319-2076  Fax: 301-319-7545  Email: Eileen.d.villasante.civ@mail.mil | |
|  | Keith Limbach, PhD  United States Military Malaria Vaccine Program  Naval Medical Research Center  503 Robert Grant Avenue  Silver Spring, Maryland 20910-7500  Telephone: 301-319-7657  Fax: 301-319-7545  Email: Keith.j.limbach.ctr@mail.mil | |
|  | Noelle Patterson  United States Military Malaria Vaccine Program  Naval Medical Research Center  503 Robert Grant Avenue  Silver Spring, Maryland 20910-7500  Telephone: 301-319-7362  Fax: 301-319-7545  Email: noelle.b.patterson.ctr@mail.mil | |
| **Consulting Cardiologist** | Todd C. Villines, MD, FACC, FAHA, FSCCT, FACP  Walter Reed National Military Medical Center, Department of Cardiology  Telephone: 301.295.7839  Cell: 202.386.1160  todd.c.villines.mil@mail.mil | |
| **Clinical Pharmacist** | Kelli Bankard, PharmD. BCPS  Walter Reed National Military Medical Center  Inpatient Internal Medicine Pharmacy  4494 North Palmer Road  Bethesda, MD 20889  Telephone: 301-400-0919  Kelli.j.bankard.civ@mail.mil | |
| **Clinical Trial Sites** | Naval Medical Research Center Clinical Trials Center  8901 Wisconsin Ave.  Building 17, Suite 2B  Bethesda, MD 20889  Telephone: 301-295-4298  Cell: 301-233-9640 | |
|  | Controlled Human Malaria Infection will take place at:  Walter Reed Army Institute of Research (WRAIR)  503 Robert Grant Avenue, Insectary (3rd Floor)  Silver Spring, Maryland 20910-7500 | |
|  | Site for post-challenge overnight stays:  Hotel in close proximity to NMRC CTC (TBD) | |
| **Clinical Trials Center Staff** | Yolanda Alcorta, MS, MAT  Telephone 301-295-5650  Yolanda.alcorta.ctr@med.navy.mil  Santina Maiolatesi, BA Telephone 301-295-0331 Santina.e.maiolatesi.ctr@mail.mil  Anatalio Reyes, BS, MSQSM, CCRP Telephone 301-295-1095 Anatalio.e.reyes.ctr@mail.mil  Mimi Wong Telephone 301-319-4349 Mimi.wong.ctr@mail.mil | |
| **Clinical Laboratories and Other Departments/Institutions Involved in the Trial** | | |
| **Site Investigational Product Accountability** | Santina Maiolatesi  Project Manager  United States Military Malaria Vaccine Program  Naval Medical Research Center  503 Robert Grant Avenue  Silver Spring, Maryland 20910-7500  Telephone: 301-295-0331  Fax: 301-295-8025  Email: Santina.e.maiolatesi.ctr@mail.mil | |
| **Clinical Laboratory** | Quest Diagnostics  1901 Sulphur Spring Road  Baltimore, MD 21227  Telephone: 866-697-8378 | |
| **Research Laboratories** | US Military Malaria Vaccine Program (USMMVP)  NMRC Component  Malaria Department, Infectious Diseases Directorate  503 Robert Grant Avenue  Silver Spring, Maryland 20910-7500 | |
|  | Clinical Immunology Laboratory  Contact: Martha Sedegah, PhD  Telephone: 301-391-7586  Email: Martha.sedegah.civ@mail.mil | |
|  | Virology Laboratory  Contact: Keith Limbach, PhD  Telephone: 301-319-7657  Email: Keith.j.limbach.ctr@mail.mil | |
|  | USMMVP  U.S. Army Component  Malaria Vaccine Branch  503 Robert Grant Avenue  Silver Spring, Maryland 20910 | |
|  | Entomology Branch  Contact: Andrezza Campos Chagas, PhD  Telephone: 301-319-9824  Email: andrezza.camposchagas.ctr@mail.mil | |
|  | Flow Cytometry Center & Serology Laboratory  Contact: Elke Bergmann-Leitner, PhD  Telephone: 301-319-9278  Email: elke.s.bergmannleitner.civ@mail.mil | |
| **Other Research Laboratories** | The Jenner Institute Laboratories, University of Oxford  Old Road Campus Research Building  Roosevelt Drive, Oxford, UK, OX3 7DQ  Contact: Katie Ewer, Senior Immunologist  Telephone: +44 (0)1865 617622  Email: katie.ewer@ndm.ox.ac.uk | |
|  | Biotechnology High-Performance Computing Software Applications Institute  2405 Whittier Drive  Frederick, MD 21702  Contact: Jaques Reifman  Email: jaques.reifman.civ@mail.mil  Contact: Sidhartha Chaudhury  Email: sidhartha.chaudhury.civ@mail.mil | |
| **Quality Management Systems** | Clinical Trials Center  Anatalio Reyes, MS, MSQSM, CCRP  Quality Assurance Manager  Telephone: 301-295-1095  Fax: 301-295-8025  E-mail: Anatalio.e.reyes.ctr@mail.mil | |
| **Statistician(s)** | Devin Hunt  Walter Reed Army Institute of Research  503 Robert Grant Avenue  Silver Spring, Maryland 20910  Telephone: 301-319-9571  Email: [devin.j.hunt.ctr@mail.mil](mailto:devin.j.hunt.ctr@mail.mil)  Sidhartha Chaudhury, PhD, CPT, MSC, USA  Microbiologist, Center for Enabling Capabilities  Walter Reed Army Institute of Research  503 Robert Grant Avenue  Silver Spring, Maryland 20910  Phone: 301-319-9389  Mobile: 410-952-0461  Email: sidhartha.chaudhury.mil@mail.mil | |
| **Data Management** | Naval Medical Research Center Clinical Trials Center  8901 Wisconsin Ave. Building 17, Suite 2B  Bethesda, Maryland 20889  Telephone: 301-295-4298 | |
| **Institutional Review Boards** | Naval Medical Research Center  Office of Research Administration  503 Robert Grant Avenue  Silver Spring, Maryland 20910  Office: 301-319-7276  Fax: 301-319-7277  FWA00000152 IRB00001008 | |
|  | Walter Reed Army Institute of Research  Human Subjects Protection Branch  503 Robert Grant Avenue, Room 1W36  Silver Spring, Maryland 20910  Main: 301-319-9940  Fax: 301-319-9961  FWA00000015 IRB00000794 | |
| **USAMRMC Office of Research Protections** | HQ USAMRMC IRB  US Army Medical Research and Materiel Command  ATTN: MRMC-RPI  504 Scott Street  Fort Detrick, Maryland 21702-5012  Fax: 301-619-4165  Telephone: 301-619-6240  Email: usarmy.detrick.medcom-usamrmc.other.irb-office@mail.mil | |
| **Collaborating Institutions** | University of Oxford of Wellington Square  Oxford, UK, OX1 2JD | |
|  | Walter Reed Army Institute of Research  503 Robert Grant Avenue  Silver Spring, MD 20910 | |
|  | United States Agency for International Development (USAID)  Global Health Bureau  Office of Health, Infectious Disease and Nutrition  Malaria Vaccine Development Program  1300 Pennsylvania Avenue NW  Washington, DC 20523-3700 | |
| FOR OFFICIAL USE ONLY  Information and data included in this document contain privileged and/or proprietary information, which is the property of the United States Army. No person is authorized to make it public without express written permission of the United States Army. These restrictions on disclosure will apply equally to all future information, which is indicated as privileged or proprietary. | |  |

Investigator’s Agreement

**A Phase 1 Trial with Controlled Human Malaria Infection to Evaluate the Safety, Immunogenicity, and Protective Efficacy of Two-Antigen and Three-Antigen *Plasmodium falciparum* DNA Prime- Adenovirus Boost Malaria Vaccine Regimens in Healthy Malaria Naïve Adults**

“I have read this protocol and agree to conduct the study as outlined herein in accordance with International Conference on Harmonization Good Clinical Practice Guideline and FDA, DoD, and United States Army Regulations.”

|  |  |  |
| --- | --- | --- |
| Christopher Duplessis, MD, MPH, CDR, MC, USN Principal Investigator NMRC, Clinical Trials Center |  | Date |

Emergency Contact Information

| Role in Study | Name | Address and Telephone Number |
| --- | --- | --- |
| Principal Investigator | Christopher Duplessis, MD, MPH, CDR, MC, USN | Naval Medical Research Center Infectious Diseases Directorate US Military Malaria Vaccine Program  503 Robert Grant Avenue Silver Spring, MD 20910 Telephone: 301-295-0021 Cell: 240-778-7268 |
| Clinical Study Team | Yolanda Alcorta, MS, MAT  Santina Maiolatesi, BA  Anatalio Reyes, BS, MSQSM, CCRP  Mimi Wong, BS | Naval Medical Research Center Clinical Trials Center 8901 Wisconsin Avenue Building 17b, Suite 2b  Bethesda, MD 20889 Telephone: 301-295-4298 Cell: 301-233-9640 |
| Research Monitors | Charmagne G Beckett, MD, MPH, FACP, CAPT, MC, USN | Naval Medical Research Center  Infectious Diseases Directorate  503 Robert Grant Ave  Silver Spring, Maryland 20910  Desk 301-319-7662  Email: charmagne.g.beckett.mil@mail.mil |
|  | Alexandra Singer, MD, LCDR, MC, USN | Defense Health Agency  J3/Public Health  7700 Arlington Blvd, Suite 3M348A  Falls Church, VA 22042-5143  Office: (703) 681-6866  Cell: (803) 553-2447  Email: alexandra.l.singer.mil@mail.mil |
| Office of Regulated Activities (ORA) | Director, ORA | USAMRMC 1430 Veterans Drive Fort Detrick, MD 21702-5009 Telephone: 301-619-1106 Fax: 301-619-7790 |
| Sponsor’s Representative | Mark S. Paxton, JD, MS | USAMRMC 1430 Veterans Drive Fort Detrick, MD 21702-5009 Telephone: 301-619-0317 Email: usarmy.detrick.medcom-usamrmc.mbx.regulatory-affairs@mail.mil |
| Institutional Review Board | Naval Medical Research Center  Office of Research Administration | 503 Robert Grant Avenue Silver Spring, MD 20910 Telephone: 301-319-7276 Fax: 301-319-7277 |
|  | Human Research Protections Office  US Army Medical Research and Materiel Command | ATTN: MCMR-RPI 504 Scott Street Fort Detrick, MD 20702-5012 Telephone: (301) 619-6240/ DSN 343-6240 Fax: (301) 619-4165 Email: usarmy.detrick.medcom-usamrmc.other.irb-office@mail.mil |

1. Synopsis

| Name of Sponsor:  The Surgeon General, Department of the Army | |
| --- | --- |
| **Name of Investigational Products:**  Vaccine Names:  (1) NMRC-M3V-D-PfCA/ChAd63-PfCA (referred to herein as D/ChAd63-CA)  (2) NMRC-M3V-D-PfCAT/ChAd63-PfCAT (referred to herein as D/ChAd63-CAT)  Vaccine Priming Components (DNA):  (1) NMRC-M3V-D-PfCA (referred to herein as D-CA)  (2) NMRC-M3V-D-PfCAT (referred to herein as D-CAT)  Vaccine Boosting Components (adenovector):   1. ChAd63 CA 2. ChAd63 CAT   Vaccine Priming Products (DNA):  (1) NMRC-MV-D-PfC (VCL-2571-Ald) (referred to herein as D-C)  (2) NMRC-MV-D-PfA (VCL-2577-Ald) (referred to herein as D-A)  (3) NMRC-MV-D-PfT (VCL-2576-Ald) (referred to herein as D-T)  Vaccine Boosting Products (adenovector):  (1) ChAd63 CSP (referred to herein as ChAd63-C)  (2) ChAd63 AMA1 (referred to herein as ChAd63-A)  (3) ChAd63 ME-TRAP (referred to herein as ChAd63-T) | |
| **Name of Active Ingredient:**   1. Three replication-deficient chimpanzee-adenovirus 63 (ChAd63) vectors expressing *Plasmodium falciparum* (Pf) circumsporozoite protein (CSP or C), Pf apical membrane antigen-1 (AMA1 or A) or Pf thrombospondin-related adhesion protein/sporozoite surface protein-2 (TRAP/SSP2 or T) fused to multiple epitopes (ME) from several other Pf proteins (ME-TRAP) 2. Three DNA plasmids each expressing PfCSP, PfAMA1, or PfTRAP/SSP2 (lacking the multiple-epitope string) | |
| **Title of Study:**  A Phase 1 Trial with Controlled Human Malaria Infection to Evaluate the Safety, Immunogenicity, and Protective Efficacy of Two-Antigen and Three-Antigen *Plasmodium falciparum* DNA Prime-Adenovirus Boost Malaria Vaccine Regimens in Healthy Malaria Naïve Adults | |
| **Study Sites(s):**  Naval Medical Research Center (NMRC) Clinical Trials Center ;  Walter Reed Army Institute of Research (WRAIR) Insectary for administration of controlled human malaria infection (CHMI or malaria challenge);  Local hotel in close proximity to CTC (to be determined) for post CHMI follow-up | |
| **Principal Investigator:**  Christopher Duplessis, MD, MPH, CDR, MC, USN | |
| **Study Period (years):**  Estimated date first subject enrolled: May 2018  Estimated date last study visit completed: Feb 2019 | **Phase of Development:**  Phase 1 (with Controlled Human Malaria Infection [CHMI]) |
| **Objectives:**  Primary:   - Assess the safety and tolerability of a heterologous prime-boost regimen expressing CSP (C) and AMA1 (A) antigens through a DNA vaccine prime (D-CA) with chimpanzee adenovirus 63 vaccine boost (ChAd63-CA) in healthy malaria-naïve adults. - Assess the safety and tolerability of a heterologous prime-boost regimen expressing CSP (C), AMA1 (A), and TRAP (T) antigens through a DNA vaccine prime (D-CAT) with chimpanzee adenovirus 63 vaccine boost (ChAd63-CAT) in healthy malaria-naïve adults.   Secondary:   - Assess the protective efficacy of 2 heterologous prime-boost regimens (D /ChAd63-CA, and D/ChAd63-CAT) in healthy malaria-naïve adults against a CHMI with Pf sporozoites administered by mosquito bites. - Assess the cellular immunogenicity of these prime-boost regimens to CSP, AMA1 and TRAP antigens by cytokine FluoroSpot and flow cytometry assays - Assess the humoral immunogenicity of these prime-boost regimens by: Enzyme linked immunosorbent assay (ELISA) to PfCSP NANP repeat peptide, N- and C-terminal peptides, and full length recombinant protein; to PfAMA1 recombinant protein; and to Pf TRAP recombinant protein - Analysis of immunofluorescence assay (IFA) titers against sporozoite and erythrocyte stage parasites - Assess the association between the subjects’ pre-ChAd63 immunization neutralizing antibody titers to human adenovirus serotype 5 (HuAd5) and the protective efficacies against CHMI, humoral and cellular immunogenicity of these prime-boost regimens to CSP, AMA1 and TRAP as measured by ELISAs and FluoroSpot assays as previously described. - Assess the rate of seroconversion to HuAd5 among subjects who are immunized with the ChAd63 boost vaccine. Compare the safety, tolerability, immunogenicity, and protective efficacy of. D-CA + ChAd63-CA vs. D-CAT + ChAd63-CAT   Exploratory:   - Collect continuous physiological status monitoring data using non-invasive wearable devices before, during, and after malaria infection in infectivity control subjects | |

| **Methodology:**  This is a study designed to assess the safety, tolerability, immunogenicity, and protective efficacy of 2 heterologous prime-boost vaccine regimens in healthy, malaria naïve adults. The study will include 2 vaccine groups and an infectivity control (IC) group consisting of non-immunized subjects. Subjects to be immunized will be randomly assigned to one of two vaccine groups as specified in the table below. Subjects will be blinded to their treatment groups. Each vaccine group will be immunized according to the schedule shown in the following figure:     \| Group \| Week \| Event \| \| --- \| --- \| --- \| \| 1 \| 0 \| Prime with D-CA \| \|  \| 4 \| Prime with D-CA \| \|  \| 8 \| Prime with D-CA \| \|  \| 24 \| Boost with ChAd63-CA \| \|  \| 28 \| CHMI \| \| 2 \| 0 \| Prime with D-CAT \| \|  \| 4 \| Prime with D-CAT \| \|  \| 8 \| Prime with D-CAT \| \|  \| 24 \| Boost with ChAd63-CAT \| \|  \| 28 \| CHMI \| \| Infectivity Control \| 28 \| CHMI \|   Approximately 4 weeks after administration of the boosting immunization the vaccinated groups and the IC group will participate in CHMI wherein subjects will be exposed to the bites of 5 *Anopheles stephensi* mosquitoes carrying infectious Pf sporozoites within a controlled clinical environment. Protection will be determined by the examination of thick blood smears through 28 days post-CHMI and by retrospective PCR analysis for the presence of blood stage parasites. All groups will be enrolled and evaluated in one cohort. Due to a limit in the number of subjects who can undergo malaria challenge at the facility in one day, CHMI will be conducted in approximately 2 days. |
| --- | --- | --- | --- | --- | --- | --- | --- | --- | --- | --- | --- | --- | --- | --- | --- | --- | --- | --- | --- | --- | --- | --- | --- | --- | --- | --- | --- | --- | --- | --- | --- | --- | --- | --- | --- | --- |
| **Estimated Number of Subjects Screened:**  236 (4 screened:1 enrolled) |
| **Maximum Number of Subjects Enrolled:**  Enrollment of a subject occurs when an individual who has been screened meets all inclusion and exclusion criteria, and has undergone the first post-screening study-specific procedure. The first post-screening study-specific procedure for immunized subjects of Groups 1, and 2 will be the first immunization. The first post-screening study-specific procedure for the IC Group will be CHMI.  Total number of subjects: 52  20 in Group 1  20 in Group 2  12 in IC group  4 Alternates for Groups 1 and 2  3 Alternates for the Infectivity Control Group |
| **Main Criteria for Inclusion/Exclusion:**  The study population will consist of healthy, malaria naïve men and women ages 18 through 50, inclusive, recruited from the Washington, DC/Baltimore metropolitan area. |
| **Investigational Product Dosage, Schedule, and Mode of Administration:**  Group 1 (2-antigen): 3 doses at 4 week intervals (Week 0, 4, and 8) of DNA prime with D-C + D-A at 2 mg total (1 mg per construct) per dose as two 1 mL IM injections of the blended D-CA, one in each arm, via Biojector 2000 needle-free injection device or an equivalent disposable syringe needle-free injection device. This will be followed after 16 weeks (Week 24) by 1 dose of ChAd63-C + ChAd63-A boost, at a total dose of 1 x 10^11^ virus particles (vp) (5 x 10^10^ vp/construct) as a single IM injection of 0.65mL, using a needle and syringe.  Group 2 (3-antigen): 3 doses at 4 week intervals (Week 0, 4, and 8) of DNA prime with D-C + D-A + D-T at 3 mg total (1 mg per construct) per dose as two 1 mL intramuscular (IM) injections of the blended D-CAT, one in each arm, via Biojector 2000 needle-free injection device or an equivalent disposable syringe needle-free injection device. This will be followed after 16 weeks (Week 24) by 1 dose of ChAd63-C + ChAd63-A + ChAd63-T boost, at a total dose of 1.5 x 10^11^ vp (5 x 10^10^ vp/construct) as a single IM injection of 1.0mL, using a needle and syringe. |
| **Duration of Participation:**  Each immunized subject will actively participate up to approximately 50 weeks (screening, immunization, CHMI, and follow-up). Infectivity control subjects will participate up to approximately 24 weeks including screening, CHMI, treatment, and follow-up. |
| **Criteria for Evaluation:**  Primary:   - Occurrence, severity, and duration of solicited adverse events following immunization through Day 7 after each immunization - Occurrence, severity, and duration of unsolicited adverse events, abnormal physical findings, and abnormal laboratory values following immunization through Day 28 after each immunization - Occurrence of any serious adverse events, as defined in 21 CFR 312.32 throughout the study period   Secondary:   - Vaccine efficacy as determined by protection from the development of parasitemia and time to development of parasitemia, as measured by microscopic examination of thick smears and by retrospective PCR after CHMI - Analysis of IFN-γ, interleukin (IL)-2, IFN-γ + IL-2, Granzyme B and IFN-γ + Granzyme B cytokine secretion in response to stimulation with synthetic peptides derived from PfCSP, PfAMA1, and Pf-TRAP by FluoroSpot assay using peripheral blood mononuclear cells (PBMC) - Analysis of IFN-γ, TNF-α, and IL-2 cytokine secretion by intracellular cytokine staining (ICS) using multi-parameter flow cytometry in response to stimulation with synthetic peptides derived from PfCSP, PfAMA, Pf-TRAP using PBMCs - Enzyme linked immunosorbent assay (ELISA) to PfCSP NANP repeat peptide, N- and C-terminal peptides, and full length recombinant protein; to PfAMA1 recombinant protein; and to Pf-TRAP recombinant protein - Measures of correlation between pre-ChAd63 immunization HuAd5 neutralizing antibody titers and protective efficacies against CHMI, humoral and cellular immune responses of the 2 prime-boost regimens - Measurement of antibody titers against sporozoite and erythrocyte stage parasites by immunofluorescence assay (IFA) using sera/plasma - Comparison of safety, immunogenicity, and vaccine efficacy of D-CA/ChAd63-CA and D-CAT/ ChAd63-CAT |
| **Statistical Methods:**  Descriptive statistics (percentage of study subjects, rate/immunization) will be used to characterize the occurrence of local and systemic solicited and unsolicited adverse events in immunized subjects. Measurements with normal distributions expressed as means of continuous data (e.g., magnitude of responses) will be assessed using the Student’s t test (2-tailed), paired if pre-immunization values are compared with post immunization values, and unpaired if comparisons are made between groups. For discrete variables with normal distributions (e.g. number of responders, the number of positive assays, the number of individuals protected against CHMI), the chi-squared test or Fisher’s exact test will be used (2-tailed, uncorrected for chi-squared), except when the cell value is 5 or less, in which case only Fisher’s exact test will be used (2-tailed).  For days to parasitemia, subjects will be rank-ordered and a non-parametric test suitable for unpaired groups (e.g., Mann Whitney) will be employed (1-tailed if comparing a vaccine group to controls, 2-tailed if comparing one vaccinated group to another vaccinated group). Days to parasitemia will be assessed using a Cox Proportional Hazards model and will be displayed using Kaplan-Meier plots. The association between an immune response and protection (time to parasitemia) will be evaluated using the Accelerated Failure Time model, with delay in onset of parasitemia indicating partial protection and with censoring any fully protected subjects on Day 28; test results for this model will be corrected for the number of comparisons performed (Bonferroni correction). |

Table of Contents, List of Tables, and List of Figures

Table of Contents

[1. Title Page 1](#_Toc9328278)

[Investigator’s Agreement 9](#_Toc9328279)

[Emergency Contact Information 10](#_Toc9328280)

[2. Synopsis 12](#_Toc9328281)

[Table of Contents, List of Tables, and List of Figures 17](#_Toc9328282)

[3. List of Abbreviations and Definitions of Terms 23](#_Toc9328283)

[4. Introduction 29](#_Toc9328284)

[4.1. Need for a Malaria Vaccine 29](#_Toc9328285)

[4.2. Military Relevance 29](#_Toc9328286)

[4.3. Rationale for Study 29](#_Toc9328287)

[4.3.1. Previous Studies at NMRC and Oxford University 31](#_Toc9328288)

[4.3.2. Proposed Study 32](#_Toc9328289)

[4.4. Name and Description of the Investigational Product 33](#_Toc9328290)

[4.5. Summary of Nonclinical and Clinical Trials 35](#_Toc9328292)

[4.5.1. Nonclinical Studies 35](#_Toc9328293)

[4.5.1.1. DNA Vaccine 35](#_Toc9328294)

[4.5.1.2. ChAd63 Vaccine 35](#_Toc9328295)

[4.5.2. Clinical Studies 36](#_Toc9328296)

[4.6. Known and Potential Risks and Benefits to Human Subjects 37](#_Toc9328297)

[4.6.1. Risks/Discomfort to Subjects and Precautions to Minimize Risk 37](#_Toc9328298)

[4.6.1.1. Local Reactions 37](#_Toc9328299)

[4.6.1.2. Systemic Reactions 37](#_Toc9328300)

[4.6.1.3. Risks Associated with Use of the Needle-free Injection Device 38](#_Toc9328301)

[4.6.1.4. Risks Associated with Controlled Human Malaria Infection (CHMI) 38](#_Toc9328302)

[4.6.1.5. Risks Associated with Anti-malarial Medications 41](#_Toc9328303)

[4.6.1.6. Pregnancy and Lactation 43](#_Toc9328304)

[4.6.1.7. Risks Associated with Venipuncture/Blood Drawing 43](#_Toc9328305)

[4.6.1.8. Allergic Reaction 44](#_Toc9328306)

[4.6.1.9. The Risk of HIV Infection 44](#_Toc9328307)

[4.6.1.10. Risks associated with Physiological Status Monitoring 45](#_Toc9328308)

[4.6.1.11. Unknown Risks 46](#_Toc9328309)

[4.6.1.12. Alternatives to this IND Product or Study 46](#_Toc9328310)

[4.6.2. Intended Benefit for Subjects 46](#_Toc9328311)

[4.6.3. Risks to the Study Personnel and the Environment 46](#_Toc9328312)

[4.7. Investigational Product Preparation, Route of Administration, Dosage Regimen, Treatment Period, and Justification 47](#_Toc9328313)

[4.7.1. Investigational Product Preparation and Route of Administration 47](#_Toc9328314)

[4.7.1.1. DNA Vaccine 47](#_Toc9328315)

[4.7.1.2. Chimpanzee Adenovirus-Vectored Vaccine 48](#_Toc9328316)

[4.7.2. Dosage 48](#_Toc9328317)

[4.7.3. Justification 49](#_Toc9328319)

[4.8. Compliance Statement 50](#_Toc9328320)

[4.9. Study Population 50](#_Toc9328321)

[4.10. Study Site 50](#_Toc9328322)

[5. Trial Objectives and Purpose 51](#_Toc9328323)

[5.1. Primary Objectives 51](#_Toc9328324)

[5.2. Secondary Objectives 51](#_Toc9328325)

[5.3. Exploratory Objectives 52](#_Toc9328326)

[6. Trial Design 52](#_Toc9328327)

[6.1. Study Endpoints 52](#_Toc9328328)

[6.1.1. Primary Endpoints 52](#_Toc9328329)

[6.1.2. Secondary Endpoints 52](#_Toc9328330)

[6.2. Overall Study Design 53](#_Toc9328331)

[6.3. Measures Taken to Minimize/Avoid Bias 64](#_Toc9328332)

[6.4. Investigational Products 64](#_Toc9328333)

[6.4.1. Investigational Product Packaging and Labeling 66](#_Toc9328335)

[6.4.2. Investigational Product Storage 68](#_Toc9328336)

[6.4.3. Investigational Product Preparation and Administration 69](#_Toc9328337)

[6.4.3.1. DNA Priming Vaccines 69](#_Toc9328338)

[6.4.3.2. Adenovector Boosting Vaccine (ChAd63) 69](#_Toc9328339)

[6.4.4. Investigational Product Accountability 70](#_Toc9328340)

[6.5. Duration of Subject Participation 70](#_Toc9328341)

[6.6. Dose-adjustment Criteria 70](#_Toc9328342)

[6.6.1. Stopping Rules 70](#_Toc9328343)

[6.6.2. Study Termination Criteria 71](#_Toc9328344)

[6.7. Trial Treatment Randomization Codes 71](#_Toc9328345)

[6.8. Identification of Data to be Recorded on the Case Report Forms 71](#_Toc9328346)

[7. Selection and Withdrawal of Subjects 72](#_Toc9328347)

[7.1. Recruitment of Subjects 72](#_Toc9328348)

[7.2. Referral Fee 72](#_Toc9328349)

[7.3. Informed Consent Process 72](#_Toc9328350)

[7.4. Eligibility Screening 73](#_Toc9328351)

[7.5. Subject Inclusion Criteria 73](#_Toc9328352)

[7.6. Subject Exclusion Criteria 74](#_Toc9328353)

[7.7. Subject Withdrawal Criteria 76](#_Toc9328354)

[7.7.1. When and How to Withdraw Subjects 76](#_Toc9328355)

[7.7.2. Data Collected for Withdrawn Subjects 77](#_Toc9328356)

[7.7.3. Replacement of Subjects 77](#_Toc9328357)

[7.7.4. Follow-up for Withdrawn Subjects 77](#_Toc9328358)

[8. Treatment of Subjects 78](#_Toc9328359)

[8.1. Study Visit Schedule and Follow-up Periods 78](#_Toc9328360)

[8.1.1. Screening Visit 78](#_Toc9328361)

[8.1.2. Pre-Immunization Visit / Pre-CHMI Visit 80](#_Toc9328362)

[8.1.3. Immunization Visits 80](#_Toc9328363)

[8.1.4. Follow-Up Visits Post-Immunization 81](#_Toc9328364)

[8.1.5. Challenge (CHMI) 83](#_Toc9328365)

[8.1.6. Management of Subjects Post-Challenge 84](#_Toc9328366)

[8.1.7. Days 7 through 18 Post Challenge (Overnight Hotel Stays) 85](#_Toc9328367)

[8.1.8. Days 20, 22, 25, and 28 Post Challenge Visits 87](#_Toc9328368)

[8.1.9. Day 35 Post Challenge Visit 88](#_Toc9328369)

[8.1.10. Day 90 Post Challenge (Final Visit) 88](#_Toc9328370)

[8.1.11. Post-Challenge Telephone Follow-up 89](#_Toc9328371)

[8.1.12. Unscheduled Visits 89](#_Toc9328372)

[8.2. Treatment of Malaria for Parasitemic Subjects 89](#_Toc9328373)

[8.3. Photographs 90](#_Toc9328374)

[8.4. Specimen Collection/Processing/Storage 90](#_Toc9328375)

[8.5. Concomitant Medications 90](#_Toc9328376)

[8.6. Procedures for Monitoring Subject Compliance 91](#_Toc9328377)

[9. Immunogenicity Assays 91](#_Toc9328378)

[9.1. Cellular Assays 91](#_Toc9328379)

[9.2. Antibody Assays 92](#_Toc9328380)

[10. Physiological Status Monitoring 95](#_Toc9328381)

[10.1. Wearable Devices 95](#_Toc9328382)

[10.2. Data Collection 96](#_Toc9328383)

[10.3. Data Analysis Plan 96](#_Toc9328384)

[11. Safety Assessment 96](#_Toc9328385)

[11.1. Specification of Safety Endpoints 97](#_Toc9328386)

[11.2. IND Safety Reporting 97](#_Toc9328387)

[11.2.1. Adverse Event or Suspected Adverse Reaction 98](#_Toc9328388)

[11.2.2. Solicited Adverse Event 98](#_Toc9328389)

[11.2.3. Serious Adverse Event or Serious Suspected Adverse Reaction 98](#_Toc9328390)

[11.2.4. Unexpected Adverse Event or Unexpected Suspected Adverse Reaction 99](#_Toc9328391)

[11.2.5. Unanticipated Problems Involving Risks To Subjects Or Others 99](#_Toc9328392)

[11.2.6. Relationship to Investigational Product 100](#_Toc9328393)

[11.2.7. Severity Assessment 100](#_Toc9328394)

[11.3. Recording Adverse Events 101](#_Toc9328395)

[11.3.1. Methods/Timing for Assessing, Recording, and Analyzing Safety Endpoints 101](#_Toc9328396)

[11.3.1.1. Post Immunization 102](#_Toc9328397)

[11.3.1.2. Following CHMI 102](#_Toc9328398)

[11.3.2. Duration of Follow-Up of Subjects after Adverse Events 102](#_Toc9328399)

[11.4. Reporting Adverse Events 103](#_Toc9328400)

[11.4.1. Reporting Serious and Unexpected Adverse Events 103](#_Toc9328401)

[11.4.1.1. Reporting to the Sponsor 104](#_Toc9328403)

[11.4.1.2. Reporting to NMRC IRB 105](#_Toc9328404)

[11.4.2. Reporting Additional Immediately Reportable Events to the Sponsor's Safety Office and Local IRB and the USAMRMC ORP 106](#_Toc9328405)

[11.4.2.1. Pregnancy 106](#_Toc9328406)

[11.4.2.2. AE-related Withdrawal of Consent 106](#_Toc9328407)

[11.4.2.3. Pending Inspections/Issuance of Reports 106](#_Toc9328408)

[11.4.3. IND Annual Reports and Final Clinical Study Reports 106](#_Toc9328409)

[11.4.3.1. IND Annual Report to the FDA 106](#_Toc9328410)

[11.4.3.2. Final Clinical Study Report 107](#_Toc9328411)

[12. Statistics 107](#_Toc9328412)

[12.1. Description of Statistical Methods 107](#_Toc9328413)

[12.1.1. Statistical Tests 107](#_Toc9328414)

[12.1.2. Safety Analyses 108](#_Toc9328415)

[12.1.3. Efficacy Analyses 108](#_Toc9328416)

[12.1.4. Clinical Laboratory Data Analyses 109](#_Toc9328417)

[12.1.5. Analysis of Immune Responses 109](#_Toc9328418)

[12.1.6. Assessment of PSM Data Collection 109](#_Toc9328419)

[12.2. Sample Size and Power Calculations 109](#_Toc9328420)

[12.3. Statistical Criteria for the Termination of the Trial 111](#_Toc9328421)

[12.4. Accounting for Missing, Unused, and Spurious Data 111](#_Toc9328422)

[12.5. Procedures for Reporting Deviations from the Original Statistical Plan 111](#_Toc9328423)

[12.6. Selection of Subjects to be Included in Analyses 111](#_Toc9328424)

[13. Direct Access to Source Data/Documents 111](#_Toc9328425)

[13.1. Study Monitoring 111](#_Toc9328426)

[13.2. Audits and Inspections 112](#_Toc9328427)

[13.3. Institutional Review Board 112](#_Toc9328428)

[14. Quality Management Systems 112](#_Toc9328429)

[14.1. Policies, Processes, Procedures, and Forms 112](#_Toc9328430)

[14.2. Document Control 113](#_Toc9328431)

[14.3. Personnel and Training 113](#_Toc9328432)

[14.4. Quality Assurance and Quality Control 113](#_Toc9328433)

[14.5. Equipment and Facility 113](#_Toc9328434)

[14.6. Computer System Validation 113](#_Toc9328435)

[15. Ethics 114](#_Toc9328436)

[15.1. Ethics Review 114](#_Toc9328437)

[15.1.1. Review/Approval of Study Protocol 114](#_Toc9328438)

[15.1.2. Protocol Modifications 114](#_Toc9328439)

[15.1.3. Protocol Deviation Procedures 115](#_Toc9328440)

[15.2. Ethical Conduct of the Study 115](#_Toc9328441)

[15.2.1. Confidentiality 115](#_Toc9328442)

[15.2.2. Compensation for Participation 116](#_Toc9328443)

[15.2.3. Medical Care for Research-Related Injury 117](#_Toc9328444)

[15.3. Written Informed Consent 117](#_Toc9328445)

[16. Data Handling and Recordkeeping 118](#_Toc9328446)

[16.1. Inspection of Records 118](#_Toc9328447)

[16.2. Retention of Records 119](#_Toc9328448)

[17. Publication Policy 120](#_Toc9328449)

[18. List of References 121](#_Toc9328450)

[Appendix A. Informed Consent Document (Immunized and Infectivity Controls) 126](#_Toc9328451)

[Appendix B. Human Immunodeficiency Virus (HIV) Testing Consent 127](#_Toc9328452)

[Appendix C. Assessment of Understanding (Immunized and Infectivity Controls) 128](#_Toc9328453)

[Appendix D. Gaziano Cardiovascular Disease Risk Assessment Chart for Men and Women 129](#_Toc9328454)

[Appendix E. Informed Consent Document for Infectivity Controls, Additional Consent for Physiological Status Monitoring 131](#_Toc9328455)

[Appendix F. Toxicity Grading Scales 132](#_Toc9328456)

List of Tables

[Table 1: Vaccine Products 34](#_Toc9328457)

[Table 2: Summary of Previous Study Protocols: HuAd5-CA and D/HuAd5-CA 36](#_Toc9328458)

[Table 3: Vaccine Doses 49](#_Toc9328459)

[Table 4: Study Design by Group 53](#_Toc9328460)

[Table 5: Study Event Schedule and Procedures from Day -90 through Day 195 for Immunized Subjects 54](#_Toc9328461)

[Table 6: Study Event Schedule and Procedures from Day 196 Through Day 286 for Immunized Subjects 58](#_Toc9328462)

[Table 7: Study Event Schedule and Procedure for Infectivity Control Subjects 60](#_Toc9328463)

[Table 8: Investigational Vaccines 64](#_Toc9328464)

[Table 9: Blood Volumes Required for Cellular Immunogenicity Assays 91](#_Toc9328465)

[Table 10: Blood Volume Requirements for Antibody Assays 93](#_Toc9328466)

[Table 11: Immunogenicity Assays 94](#_Toc9328467)

[Table 12: Local and Systemic Solicited Adverse Events 98](#_Toc9328468)

[Table 13: Categories for Adverse Event Relationship to Investigational Product 100](#_Toc9328469)

[Table 14: Adverse Event Severity Categories 101](#_Toc9328470)

[Table 15: Study Contacts for Reporting Serious Adverse Events and Unanticipated Problems Involving Risk to Patients or Others 104](#_Toc9328471)

[Table 16: SAE Information to be Reported to the Sponsor's Safety Office 105](#_Toc9328472)

[Table 17: Power for Comparing Vaccine Efficacies between Groups 1 and 2 110](#_Toc9328473)

[Table 18: Grading Scale for Clinical Abnormalities (Local Reaction to Injectable Product) 133](#_Toc9328474)

[Table 19: Grading Scale for Clinical Abnormalities (Vital Signs) 134](#_Toc9328475)

[Table 20: Grading Scale for Clinical Abnormalities (Symptoms) 135](#_Toc9328476)

[Table 21: Grading Scale for Laboratory Abnormalities 136](#_Toc9328477)

List of Figures

[Figure 1: Vaccine 1 Components and Products 33](#_Toc9328478)

[Figure 2: Vaccine 2 Components and Products 34](#_Toc9328479)

[Figure 3: Study Design 53](#_Toc9328480)

[Figure 4: Label Information for the DNA Priming Products 66](#_Toc9328481)

[Figure 5: Label Information for the ChAd63 Boosting Products 68](#_Toc9328482)

1. List of Abbreviations and Definitions of Terms

| Abbreviation | Explanation |
| --- | --- |
| Ad | Adenovirus |
| AdCh63 | ChAd63, Chimpanzee adenovirus serotype 63 |
| AdCh63 AMA1 | ChAd63 AMA1; Chimpanzee Adenovector Serotype 63 expressing *P falciparum* Apical Membrane Antigen 1 |
| AdCh63 ME-TRAP | ChAd63 TRAP; Chimpanzee Adenovector Serotype 63 expressing a string of malaria multiple epitopes and *P falciparum* Thrombospondin-Related Adhesion Protein |
| AE | Adverse event, adverse experience |
| Ag | Antigen |
| AI | Associate Investigator |
| ALT | Alanine aminotransferase |
| AMA1 | Apical Membrane Antigen 1 |
| AR | Army Regulation |
| AST | Aspartate aminotransferase |
| β-HCG | Beta-Human chorionic gonadotropin |
| BUMED | Navy Bureau of Medicine and Surgery |
| C | *P* *falciparum* Circumsporozoite Protein |
| CBC | Complete Blood Count |
| CBER | Center for Biologics Evaluation and Research |
| CBF | Clinical BioManufacturing Facility, Oxford, UK |
| CD8 | Cluster of differentiation 8 |
| CFR | Code of Federal Regulation |
| ChAd63 | AdCh63; Chimpanzee adenovirus serotype 63 |
| ChAd63-A | AdCh63 AMA1; ChAd63 AMA1; Chimpanzee Adenovector Serotype 63 expressing *P falciparum* Apical Membrane Antigen 1 |
| ChAd63 AMA1 | Chimpanzee Adenovector Serotype 63 expressing *P falciparum* Apical Membrane Antigen 1 |
| ChAd63-C | ChAd63 CS; ChAd63 CSP; Chimpanzee Adenovector Serotype 63 expressing *P falciparum* Circumsporozoite Protein |
| ChAd63 CS | Chimpanzee Adenovector Serotype 63 expressing *P falciparum* Circumsporozoite Protein |
| ChAd63 CSP | Chimpanzee Adenovector Serotype 63 expressing *P falciparum* Circumsporozoite Protein |
| ChAd63-PfCA | Chimpanzee Adenovector Serotype 63 expressing *P falciparum* Circumsporozoite Protein and Apical Membrane Antigen 1 |
| ChAd63-PfCAT | Chimpanzee Adenovector Serotype 63 expressing *P falciparum* Circumsporozoite Protein, Apical Membrane Antigen 1 and Thrombospondin-Related Adhesion Protein/ Sporozoite Surface Protein 2 |
| ChAd63-T | AdCh63 ME-TRAP; ChAd63 TRAP; Chimpanzee Adenovector Serotype 63 expressing a string of malaria multiple epitopes and *P falciparum* Thrombospondin-Related Adhesion Protein |
| ChAd63 TRAP | Chimpanzee Adenovector Serotype 63 expressing a string of malaria multiple epitopes and *P falciparum* Thrombospondin-Related Adhesion Protein |
| CHMI | Controlled Human Malaria Infection |
| CRO | Contract Research Organization |
| CRF | Case Report Form |
| CSP | Circumsporozoite protein |
| CTC | Clinical Trials Center |
| C-terminus | Carboxyl-terminus |
| D-A | NMRC-MV-D-PfA (VCL-2577-Ald) |
| D-C | NMRC-MV-D-PfC (VCL-2571-Ald) |
| D-CA | NMRC-M3V-D-PfCA |
| D-CAT | NMRC-M3V-D-PfCAT |
| D-T | NMRC-MV-D-PfT (VCL-2576-Ald) |
| DNA, D | Deoxyribonucleic acid |
| D/ChAd63-CA | NMRC-M3V-D/ChAd63-PfCA |
| D/ChAd63-CAT | NMRC-M3V-D/ChAd63-PfCAT |
| D/HuAd5-CA | NMRC-M3V-D/Ad5-PfCA |
| DoD | Department of Defense |
| ECG, EKG | Electrocardiogram |
| EDC | Electronic data capture |
| ELISA | Enzyme linked immunosorbent assay |
| ELISpot | Enzyme linked immunospot assay |
| EMS | Emergency Medical Services |
| FDA | US Food and Drug Administration |
| FFB | Final Formulation Buffer |
| F/U | Follow-up |
| GCP | Good Clinical Practice |
| GLP | Good Laboratory Practices |
| GMP | Good Manufacturing Practices |
| HbsAg | Hepatitis B surface antigen |
| HCV | Hepatitis C virus |
| HIPAA | Health Insurance Portability and Accountability Act |
| HIV | Human Immunodeficiency Virus |
| HLA | Human leukocyte antigen |
| HRPO | Human Research Protection Office |
| HSPB | Human Subjects Protection Branch |
| HuAd5 | Human Adenovirus Serotype 5 |
| HuAd5-A | NMRC-MV-Ad-PfA |
| HuAd5-C | NMRC-MV-Ad-PfC |
| HuAd5-CA | NMRC-M3V-HuAd5-PfCA |
| IB | Investigator’s Brochure |
| IC | Infectivity Control |
| ICD | Informed Consent Document |
| ICH | International Conference on Harmonization |
| ICS | Intracellular Cytokine Staining |
| ID | Infectious Dose |
| IFA(T) | Indirect fluorescent antibody (test) |
| IFN-γ, IFN-gamma, IFN-g | Interferon gamma |
| IM | Intramuscular |
| Imm | Immunization |
| IMPD | Investigational Medicinal Product Dossiers |
| IND | Investigational New Drug |
| INR | International normalized ratio |
| IRB(s) | Institutional Review Board(s) |
| Irr-spz | Irradiated Sporozoite |
| LN | Lot Number |
| LSA1 | Liver Stage Antigen 1 |
| M3V | Multi-Stage, Multi-Antigen, Malaria Vaccine |
| M | Molar |
| ME | Multiple epitopes or multi-epitope |
| ME-TRAP | Malaria multiple epitopes and *P falciparum* Thrombospondin-Related Adhesion Protein |
| MF | Master File |
| MHRA | UK Medicines and Healthcare Products Regulatory Agency |
| μg | Microgram |
| mg | Milligram |
| MgC_l2_ | Magnesium Chloride |
| MIDRP | Military Infectious Diseases Research Program |
| mL | Milliliter |
| mM | Millimoles |
| MSP1_42_ | Merozoite Surface Protein 1 42 kDa C terminus |
| MV | Malaria Vaccine |
| MVA | Modified Vaccinia Ankara |
| Nab | Neutralizing Antibodies |
| NaCl | Sodium Chloride |
| NHP | Non-Human Primate |
| NIH | National Institutes of Health |
| NMRC | Naval Medical Research Center |
| NMRC-M3V-D-PfCA | NMRC – Multi-Stage, Multi-Antigen, Malaria Vaccine – DNA – expressing *P falciparum* Circumsporozoite Protein and Apical Membrane Antigen 1 |
| NMRC-M3V-D-PfCAT | NMRC – Multi-Stage, Multi-Antigen, Malaria Vaccine – DNA – expressing *P falciparum* Circumsporozoite Protein, Apical Membrane Antigen 1, and Thrombospondin-Related Adhesion Protein |
| NMRC-MV-D-PfA (VCL-2577-Ald) | NMRC – Malaria Vaccine – DNA – expressing *P falciparum* Apical Membrane Antigen 1-Vical designation-Aldevron |
| NMRC-MV-D-PfC (VCL-2571-Ald) | NMRC – Malaria Vaccine – DNA – expressing *P falciparum* Circumsporozoite Protein –Vical designation-Aldevron |
| NMRC-MV-D-PfT (VCL-2576-Ald) | NMRC – Malaria Vaccine – DNA – expressing *P falciparum* Thrombospondin-Related Adhesion Protein –Vical designation-Aldevron |
| N-terminus | Amino terminus |
| OD | Optical Density |
| OHRP | Office for Human Research Protections, Department of Health and Human Services |
| ORA | Office of Regulated Activities |
| ORF | Open Reading Frame |
| ORM | Office of Research Management |
| ORP HRPO | Office of Research Protections, Human Research Protection Office |
| PBMC(s) | Peripheral Blood Mononuclear Cell(s) |
| pDNA | Plasmid DNA |
| *Pf, P falciparum* | *Plasmodium falciparum* |
| PfSPZ | *Plasmodium falciparum* Sporozoite |
| PfSPZ CVac approach | *Plasmodium falciparum* Sporozoite challenge administered with chloroquine chemoprophylaxis as a vaccine approach |
| PI | Principal investigator |
| *Pk, P knowlesi* | *Plasmodium knowlesi* |
| Pre-IMM | pre-immunization |
| PSM | Physiological status monitoring |
| PT | Prothrombin time |
| PTT | Partial thromboplastin time |
| Pu | Particle Units |
| *Py, P. yoelii* | *Plasmodium yoelii* |
| PSSB | Product Safety Surveillance Branch |
| QA | Quality Assurance |
| QC | Quality Control |
| RCA | Replication competent adenovirus |
| RNA | Ribonucleic acid |
| RT-PCR | Real-Time Polymerase Chain Reaction |
| RUNMC | Radboud University Nijmegen Medical Center, Netherlands |
| SAE | Serious Adverse Event |
| SFC | Spot Forming Cell |
| SHIV | Simian Human Immunodeficiency Virus |
| SIV | Simian Immunodeficiency Virus |
| SMC | Safety Monitoring Committee |
| SOP | Standard Operating Procedure |
| Spp | Species |
| SRC | Scientific Review Committee |
| SSP | Study Specific Procedure |
| SSP2 | Sporozoite Surface Protein 2 |
| SST | Serum separating tube |
| SWFI | Sterile Water For Injection |
| TBD | To Be Determined |
| TIS1 | Transcriptionally Inert Spacer 1 |
| TRAP | Thrombospondin-Related Adhesion Protein |
| TSG | The Surgeon General |
| USAID | United States Agency for International Development |
| USAMMDA | US Army Medical Materiel Development Activity |
| USAMRAA | US Army Medical Research Acquisition Activity |
| USAMRMC | US Army Medical Research & Materiel Command |
| USMMVP | US Military Malaria Vaccine Program |
| USUHS | Uniformed Services University of the Health Sciences |
| VDP | Vaccine Drug Product |
| vp | Viral particle |
| VSS | Vector Seed Stock |
| VV | Vaccinia Virus |
| WCB | Working Cell Bank |
| WHO | World Health Organization |
| WRAIR | Walter Reed Army Institute of Research |
| WRNMMC | Walter Reed National Military Medical Center |

1. Introduction
   1. Need for a Malaria Vaccine

Malaria represents a major public health problem worldwide, causing significant morbidity and mortality in immunologically naïve individuals as well as endemic populations. According to the latest estimates, there were about 214 million new cases of malaria worldwide (range from 149 to 303 million) and an estimated 438,000 deaths in 2015. Approximately 90 percent of all malaria deaths occur in sub-Saharan Africa and the majority of these deaths occur in children under the age of 5 ([World Malaria Report-2015](#WHO_2015)). Analyses suggest that the medical impact of malaria may actually be significantly underestimated ([Breman et al-2004](#Breman_et_al_2004)) and that the enormous economic impact of malaria has never been adequately considered ([Gallup and Sachs-2001](#Gallup_and_Sachs_2001)). Both international tourists and individuals residing in malaria-free regions of malaria-endemic countries who travel to areas where malaria is transmitted are at serious risk of developing this deadly disease. Widespread and increasing drug and insecticide resistance, by the parasite and vector respectively, highlight the importance of developing an effective malaria vaccine. This remains an unmet urgent public health need ([Hill et al-2010](#Hill_et_al_2010)).

- 1. Military Relevance

Malaria has had a significant impact on US military operations throughout history. It was responsible for a greater loss of manpower than enemy fire in all conflicts occurring in tropical regions during the 20th century ([Beadle and Hoffman-1993](#Beadle_and_Hoffman_1993)). Malaria continues to present a major challenge to force health protection during operations in any environment where malaria is endemic. This includes 108 countries spanning the tropical and subtropical regions of the world, including most of sub Saharan Africa and large regions of South Asia, Southeast Asia, Oceania, Central Asia, the Middle East, Central and South America, and the Caribbean. The US military is either currently deployed or has the potential to deploy on short notice to any of these regions, making malaria a leading infectious threat to mission success. In our malaria-naïve military population, an infection with malaria can severely degrade performance, result in missed duty, and may lead to prolonged hospitalization and, in some cases, death. The measures used to avoid malaria frequently compromise military performance and, given the difficulties of implementing control measures and chemoprophylaxis in combat, cannot be completely relied upon to prevent infection. Deployment of troops in a number of endemic locations including Liberia in 2003, 2009-2010, and 2014; Benin in 2009; and Haiti in 2010 underscore the Department of Defense’s (DoD) critical need for a malaria vaccine for deployed military personnel ([Whitman et al-2010](#Whitman_et_al_2010); [Armed Forces Health Surveillance-2010](#AFHSC_2010); [Centers for Disease Control-2010](#DCD_2010)). Of the 5 malaria species infecting humans, *Plasmodium falciparum* has been prioritized by the US military for vaccine development, because of its greater severity, with vaccines against a *Plasmodium vivax* a close second in priority.

- 1. Rationale for Study

A highly effective vaccine preventing malaria infection remains a top priority of the US Department of Defense (DoD). Animal models indicate that CD8+ T cells targeting liver stage malaria parasites may provide an effective immunological mechanism for such a vaccine. The purpose of this Phase 1 clinical trial is to evaluate the safety and efficacy of 2 *Plasmodium falciparum* (Pf) DNA prime-adenovector (Ad) boost vaccines. This study builds on earlier work by NMRC/Walter Reed Army Institute of Research (WRAIR) US Military Malaria Vaccine Program that demonstrated that a heterologous DNA prime-HuAd5 boost regimen encoding the Pf circumsporozoite protein (CSP) and Pf apical membrane-antigen-1 (AMA1) elicited sterile protection against parasitemia in 4 of 15 research subjects who underwent malaria challenge (27% sterile protection) ([Chuang et al-2013](#Chuang_et_al_2013)). This favorable result provides the rationale for this study with the following hypotheses:

1. A boost regimen with a chimpanzee-derived adenovector (ChAd63) using the same 2 antigens, CSP and AMA1, has an acceptable safety profile and provides similar protection as the HuAd5 boost vaccine.
2. The addition of a third antigen, ME-TRAP, to the DNA prime-ChAd63 boost regimen has an acceptable safety profile and can improve the vaccine regimen’s protective efficacy.

This clinical trial is part of a larger collaborative effort between the Navy and the University of Oxford (“NavOx collaboration”), which includes the proposed clinical trial and 2 trials already conducted in the United Kingdom (UK1 and UK2). The UK trials involved the same 3 antigens (CSP, AMA1, ME-TRAP) but a different prime-boost regimen, ChAd63 prime-MVA boost (Ad-MVA). The UK trials were conducted at the University of Oxford with Medicines and Healthcare products Regulatory Agency (MHRA) oversight.

The overall goal of the NavOx collaboration is to select the more protective of the prime-boost approaches, DNA-Ad or Ad-MVA, for further development, assuming that each regimen is safe and well-tolerated and achieves sufficient efficacy to justify advancement. The 3 ChAd63 constructs to be evaluated in this clinical study are the same as those studied in trials UK1 and UK2.

The proposed study is a proof-of-concept clinical trial to determine whether ChAd63 can replace HuAd5 in the protective regimen used in the earlier clinical trial conducted at NMRC ([Chuang et al-2013](#Chuang_et_al_2013), BB-IND 13977). In our previous studies we showed that DNA-priming was essential for protection with the HuAd5-based vaccine and there is evidence to suggest that the DNA altered the Ad immunologic responses to malaria antigens ([Sedegah et al-2014](#Sedegah_et_al_2014)). Cell-mediated immunity was implicated as important for protection for the DNA/HuAd5 (NMRC) regimen. Data from the study revealed IFN-γ ELISpot responses to AMA1 that were CD8-dependent in depletion studies showed the strongest association with protection (p = 0.019). Thus preliminary data indicated that protection was likely CD8+ T cell-dependent for each regimen.

In the previous DNA/HuAd5 clinical trial, all 4 of the protected subjects had neutralizing antibody titers for adenovirus serotype 5 of less than 500 prior to immunization, and none of the 5 vaccine recipients with neutralizing antibodies greater than 500 was protected. Although it is not known whether neutralizing antibodies acquired as a result of wild-type adenovirus infection inhibit the protective effect of adenovirus vectored vaccines in humans, data from the NMRC DNA/HuAd5 trial suggest that this may be the case. Another important limitation of the HuAd5 vector vaccine regimen is the possible increase in the relative risk of HIV infection through undetermined mechanisms recently observed in clinical trials of a HuAd5-based candidate HIV vaccine. In an effort to circumvent the limitations of the HuAd5 vaccine, vectors from nonhuman sources thought to have lower seroprevalence in human populations have been developed and evaluated in preclinical and clinical studies. The proposed study aims to determine if the ChAd63 utilized as a boost vaccine can provide a similar protection as the HuAd5 boost using the same antigens, CSP and AMA1. It is anticipated that the combination of DNA prime and ChAd63 boost regimen encoding the same antigens, CSP and AMA1, would lead to a high rate of sterile protection against controlled human malaria challenge (CHMI).

ME-TRAP was selected as the third antigen because of sterile protection induced in humans by this antigen following administration of a gene-based regimen (ChAd63 prime-modified vaccinia Ankara [MVA] boost) in several studies conducted at the University of Oxford ([Dunachie et al-2006](#Dunachie_et_al_2006), [Ewer et al-2013](#Ewer_et_al_2013), [Ogwang et al-2015](#Ogwang_et_al_2015)). A clinical trial (VAC034) conducted by the University of Oxford with the ChAd63 prime/MVA boost regimen encoding TRAP as the sole antigen showed sterile efficacy against CHMI in 21% (3/14) of subjects with an additional 36% (5/14) showing a delayed patency ([Ewer et al-2013](#Ewer_et_al_2013)). In another trial (VAC052), the ChAd63 prime/MVA boost vaccine regimen encoding 2 antigens (ME-TRAP and CSP) sterilely protected 23% (3/13) of subjects against CHMI and delayed patency in an additional 23% (3/13), but the same prime/boost regimen encoding ME-TRAP, CSP and AMA1, sterilely protected 15% (2/13) and delayed patency in 31% (4/13) (Hill et al-unpublished) indicating that there was no antigenic interference with this multicomponent vaccine. In a recently published clinical trial, 121 healthy adult male research subjects in Kenya received the ChAd63-ME-TRAP prime vaccine followed by the MVA-ME-TRAP boost vaccine and efficacy was measured under conditions of natural transmission in malaria-endemic field sites. The frequency of malaria detected by qPCR was reduced by 67% over a 2-week period during malaria transmission ([Ogwang et al-2015](#Ogwang_et_al_2015)). These data provide further rationale for including TRAP in the 3-antigen combination in Group 2, as we hypothesize that inclusion of TRAP will increase efficacy of the 2-antigen group either additively or synergistically, by inducing T cell responses that recognize liver stages that express each of these antigens. The much higher levels of protection seen with the radiation-attenuated sporozoite vaccines ([Hoffman et al-2002](#Hoffman_et_al_2002), [Seder et al-2013](#Seder_et_al_2013)) compared with single-antigen subunit vaccines, highly suggest that multiple antigens will be needed to provide high levels of protection in a sub-unit vaccine and this trial will provide a proof-of-concept for this approach using a DNA prime-viral vector boost regimen.

- - 1. Previous Studies at NMRC and Oxford University

In a previous study at NMRC (IND 13977, S-13-03, A-15350, WRAIR#1550), subjects received 3 doses of a mixture of DNA plasmids encoding the pre-erythrocytic stage antigen Pf circumsporozoite protein (CSP) and the pre-erythrocytic stage/blood stage antigen Pf apical membrane-antigen-1 (AMA1). This was followed 16 weeks later by one dose of a mixture of 2 human adenovectors (HuAd5) encoding CSP and AMA1. This heterologous prime boost regimen sterilely protected 27% (4 of 15) of subjects; all 4 subjects had neutralizing antibody titers for serotype 5 adenovirus of less than 500 ([Chuang et al-2013](#Chuang_et_al_2013)).

The clinical studies at Oxford employed a chimpanzee adenovector (ChAd63) encoding the pre-erythrocytic stage antigen Pf thrombospondin-related adhesion protein (TRAP, also called sporozoite surface protein-2, or SSP2) fused to a string of multiple malaria epitopes (ME) derived from other malaria antigens. Subjects received 1 dose of ChAd63-ME-TRAP followed 8 weeks later by 1 dose of Modified Vaccinia Ankara (MVA)-ME-TRAP. This regimen sterilely protected 21% (3 of 14) of subjects against CHMI (2 of 8 subjects in 1 study, 1 of 6 in a second study) with a further 5 of the remaining 11 subjects showing a significant delay in time to patency. In a recently published clinical trial evaluating the efficacy of the ChAd63-ME-TRAP prime vaccine followed by the MVA-ME-TRAP boost vaccine in 121 healthy adult male subjects in Kenya, the vaccination was demonstrated to reduce the risk of infection by 67% at 8 weeks post CHMI ([Ogwang et al-2015](#Ogwang_et_al_2015)).

Cell-mediated immunity was implicated as important for protection for both the DNA/Ad (NMRC) and Ad/MVA (Oxford) regimens. At NMRC, ex vivo enzyme-linked immunosorbent spot assay (ELISpot) and CD8+T cell interferon-γ (IFN-γ) responses to CSP were higher in 2 of 4 protected subjects and to AMA1 were higher in 3 of 4 protected subjects. At Oxford, IFN- γ -secreting CD8+ T cells also showed the strongest association with protection (P = 0.005). Thus preliminary data indicated that protection was likely CD8+ T cell-dependent for each regimen.

- - 1. Proposed Study

The proposed trial is designed to determine whether the sterile protection achieved at NMRC with the DNA prime and HuAd5 boost can be improved.

Concerns have been raised about the safety of HuAd5 vectors based on data from clinical trials in which the vector was used as the carrier of synthetic HIV genes. In 2013, the National Institute of Allergy and Infectious Diseases (NIAID), part of the National Institutes of Health (NIH), held a scientific meeting to examine why certain investigational HIV vaccines may have increased susceptibility to HIV infection. In a perspectives article appearing in the journal Science, HIV research leaders from NIAID and collaborators summarized the findings and considerations for future HIV vaccine research ([Fauci et al-2014](#Fauci_et_al_2014)).

Between 2005 and 2013, investigational HIV vaccines based on a HuAd5 vector were tested in 3 clinical trials. Two of those studies, known as Step and Phambili, involved the same experimental vaccine with an adenovirus-5 vector manufactured by Merck Pharmaceuticals. Both the Step and Phambili studies showed no efficacy against acquisition of HIV infection; however, they suggested an increased risk of HIV acquisition among vaccinated male study participants ([Buchbinder et al-2008](#Buchbinder_et_al_2008), [Gray et al-2011)](#Gray_et_al_2011). Based on subsequent analyses, the authors hypothesized that the HuAd5-based vaccines tested may have heightened susceptibility to HIV infection by activating CD4+ T-cells, the key target for HIV, for sustained periods while producing ineffective or limited protective effects against HIV.

The initial plan was to include a study group in which subjects would receive DNA prime-HuAd5 PfCA boost to serve as a positive control. However, after a careful re-evaluation of the study aims and objectives to ensure subject safety, it was determined that the risks exceeded the benefits of including the DNA prime-HuAd5 boost group. Hence, the first study group will be administered a heterologous prime boost regimen, in which a rare-serotype vector, ChAd63, will be substituted for HuAd5 in the DNA/Ad regimen to investigate whether equivalent (or improved) protection can be achieved. In addition, the study will address whether protection by the DNA prime ChAd63 boost regimen can be enhanced by adding a third antigen, TRAP. Subjects in the second study group will thus receive a prime-boost vaccine regimen that will express CSP, AMA1, and TRAP.

- 1. Name and Description of the Investigational Product

The vaccine regimens to be studied under this protocol are NMRC-M3V-D/ChAd63-PfCA (referred to as D/ChAd63-CA) and NMRC-M3V-D/ChAd63-PfCAT (referred to as D/ChAd63-CAT). The composition of the vaccine regimens are illustrated in Figure 1 and Figure 2. The vaccines will consist of 3 doses of the DNA priming components followed by 1 dose of the adenovirus vectored boosting components. The DNA prime and the Ad (adenovirus) boost components of each vaccine consist of mixtures of individually manufactured and vialed products (constructs) encoding Pf circumsporozoite protein (CSP), Pf apical membrane antigen-1 (AMA1), and Pf thrombospondin related adhesion protein/sporozoite surface protein-2 (TRAP/SSP2). A full description of each of these components is provided in Table 1.

The DNA priming products, D-C, D-A, and D-T are vialed separately and will be blended for each of the DNA priming regimens in a separate mixing vial prior to administration. The priming vaccines are administered at a total dose of 2 mg for the D-CA priming regimens (1 mg per construct) or 3 mg for the D-CAT priming regimens (1 mg per construct), per administration intramuscularly using the Biojector 2000^®^ needle-free jet injection device or an equivalent disposable syringe needle-free injection device to the deltoid muscle of each arm. The concentration of the mixed plasmid D-CA vaccine is 1 mg/mL, and the concentration of the mixed plasmid D-CAT vaccine is 1.5 mg/mL.

ChAd63 boosting products, ChAd63-C, ChAd63-A, and ChAd63-T, are each vialed separately and mixed just prior to administration. ChAd63 products are not diluted prior to use in the clinic. The ChAd63 boost will be delivered intramuscularly by needle and syringe as a single injection to the deltoid (0.65 mL injectate for ChAd63-CA and 1 mL injectate for ChAd63-CAT).

For detailed dosage information please refer to Section 4.7.

Figure 1: Vaccine 1 Components and Products

Figure 2: Vaccine 2 Components and Products

Table 1: Vaccine Products

| Name | Description |
| --- | --- |
| **DNA Priming Products** | |
| D-C | DNA plasmid encoding the *Plasmodium falciparum* pre-erythrocytic stage antigen circumsporozoite protein (CSP, PfCSP or “C”). The DNA sequence for the CSP antigen is derived from the 3D7 strain of Pf containing a synthetic, codon optimized gene sequence. |
| D-A | DNA plasmid encoding the *Plasmodium falciparum* pre-erythrocytic/ erythrocytic antigen apical membrane antigen-1 (AMA1, PfAMA1 or “A”). The DNA sequence for the AMA1 antigen is derived from the 3D7 strain of Pf containing a synthetic, codon optimized gene sequence. |
| D-T | DNA plasmid encoding the *Plasmodium falciparum* pre-erythrocytic stage antigen thrombospondin-related adhesion protein / sporozoite surface protein-2 (TRAP/SSP2, PfTRAP/SSP2, TRAP, or “T”). The DNA sequence for the TRAP antigen is derived from the 3D7 strain of Pf containing a synthetic, codon optimized gene sequence. |
| Phosphate Buffered Saline (PBS)  for DNA | 0.01M sodium phosphate (NaH_2_PO_4_) [pH 7.2], 0.9% NaCl |
| **Adenovector Boosting Products** | |
| ChAd63-C | Recombinant attenuated simian adenovirus, chimpanzee adenovirus serotype 63, encoding *P falciparum* CSP cloned from the Pf3D7 strain. The CSP insert is codon-optimized encoding a C-terminally truncated PfCSP protein. |
| ChAd63-A | Recombinant attenuated simian adenovirus, chimpanzee adenovirus serotype 63, encoding a mixture of 2 divergent AMA 1 alleles from 3D7 and FVO strains of Pf. The AMA1 insert in this vaccine is codon-optimized. |
| ChAd63-T | Recombinant simian adenovirus, chimpanzee adenovirus serotype 63, encoding a string of malaria multiple epitopes, ME, fused to a complete Pf TRAP of strain T9/96. |
| Formulation Buffer (FB)  for ChAd63 | 10 mM Histidine, 7.5% sucrose, 35mM NaCl, 1mM MgCl_2_, 0.1% Polysorbate 80, 0.1mM EDTA, 0.5% ethanol, pH 6.6 (this is used for formulation only, not vialed) |
| **Priming Regimens**  NMRC-M3V-D-PfCA  NMRC-M3V-D-PfCAT | NMRC-MV-D-PfC and NMRC-MV-D-PfA DNA plasmid prime  NMRC-MV-D-PfC, NMRC-MV-D-PfA, and NMRC-MV-D-PfT DNA plasmid prime |
| **Boosting Regimens**  ChAd63 CA  ChAd63 CAT | ChAd63 CSP, and ChAd63 AMA1 adenovector boost  ChAd63 CSP, ChAd63 AMA1, and ChAd63 TRAP/SSP2 adenovector boost |
| **Prime-Boost Regimens**  NMRC-M3V-D/ChAd63-PfCA (Vaccine 1)  NMRC-M3V-D/ChAd63-PfCAT (Vaccine 2) | NMRC-MV-D-PfC and NMRC-MV-D-PfA DNA plasmid prime; ChAd63 CSP and ChAd63 AMA1 adenovector boost  NMRC-MV-D-PfC, NMRC-MV-D-PfA, and NMRC-MV-D-PfT DNA plasmid prime; ChAd63 CSP, ChAd63 AMA1, and ChAd63 TRAP adenovector boost |

- 1. Summary of Nonclinical and Clinical Trials
     1. Nonclinical Studies
        1. DNA Vaccine

The 3 DNA plasmids encoding CSP, AMA1, and TRAP/SSP2 have been studied in a non-GLP 6-week intramuscular administration tissue distribution study in mice and in a GLP 12-week intramuscular administration, repeat-dose toxicity study in rabbits as part of the MuStDO9 vaccine nonclinical study portfolio (MF 12983). The first 2 DNA plasmids encoding CSP and AMA1 (but not the plasmid encoding TRAP) have also been evaluated in a non-GLP biodistribution study in mice and a GLP 67-day, repeat intramuscular administration toxicity study in rabbits (IND 13977, [Patterson et al-2006](#Patterson_2006), [Patterson et al-2007](#Patterson_2007)). In aggregate, these studies show that these vaccine products and mixtures of them are safe in animals (highest administered dose of 5 mg total pDNA per injection, 3 administrations).

- - - 1. ChAd63 Vaccine

The 3 ChAd63 adenoviral vectors encoding CSP, AMA1, and ME-TRAP/SSP2 have been studied in individual 28-day intramuscular administration ChAd63/MVA single dose each prime-boost and, for ME-TRAP/SSP2, 28-day intradermal repeat-dose toxicity studies in mice performed in the United Kingdom under UK Good Laboratory Practice Regulations and Organisation for Economic Co-operation and Development Principles of Good Laboratory Practice. The 3 ChAd63 adenovectors have also been studied as a mixture in a 28‑day intramuscular administration ChAd63/MVA single dose each prime- boost toxicity study in mice. In aggregate, these studies show that these vaccine products and mixtures of them are safe in mice.

Refer to the Investigator’s Brochure for additional information including evaluation of potential antigenic interference with administration with a multivalent vaccine and other relevant background research.

- - 1. Clinical Studies

Each of the vaccine products, in various combinations, planned for evaluation in the proposed study was found to be safe in previous clinical trials.

The DNA vaccine products D-C and D-A, as a part of the DNA prime HuAd5 boost vaccine regimen expressing the circumsporozoite protein and apical membrane antigen 1(D/HuAd5-CA), were previously studied in humans in the United States under IND 13977 ([Chuang et al-2013](#Chuang_et_al_2013)). A summary of previous studies is provided in Table 2. These studies have shown that the vaccines are safe; moreover, the D/HuAd5-CA vaccine elicited sterile protection against parasitemia in 4 of 15 challenged research subjects (27% sterile protection) under IND 13977.

Table 2: Summary of Previous Study Protocols: HuAd5-CA and D/HuAd5-CA

| Study No | Test Article (s) | Screening Ad5 serostatus | Dose | No of doses | No Subjects immunized | Challenge  yes/no | No of subjects protected |
| --- | --- | --- | --- | --- | --- | --- | --- |
| Protocol 1 (IND 13003) | | | | | | | |
| Study 1 dose escalation (2007) | HuAd5-CA  (low dose) | Negative or low titer | 2x10^10^pu | 1 | 6 | No | NA |
|  | HuAd5-CA (high dose) |  | 1x10^11^pu | 1 | 6 | No | NA |
| Study 2 regimen comparison (2008) | HuAd5-C | Negative, low titer or positive | 2x10^10^pu | 2 | 15 | Yes | 0/12 |
| Study 3  (2010) | HuAd5-CA | Negative or low titer | 2x10^10^pu | 1 | 20 | Yes | 0/18 |
| Protocol 2 (IND 13977) | | | | | | | |
| DNA/Ad-CA  (2010) | DNA prime | Negative, low titer or Positive | 1 mg per construct | 3 | 20 | Yes | 4/15 |
|  | HuAd5-CA boost |  | 2x10^10^ pu | 1 |  |  |  |

a Included 6 controls per challenge

b Ad5 low titer defined as neutralizing antibody titer ≤ 500

c 15 subjects received first dose; 14 subjects received second dose

d 20 subjects received first DNA dose, 19 subjects received second DNA dose, 19 subjects received third DNA dose, 16 subjects received all 3 DNA doses + 1 HuAd5-CA dose (complete regimen).

The D/ChAd63-CA and D/ChAd63-CAT vaccines have not been previously studied in the United States; however, the D-C and D-A products used for the prime have been studied in humans in the United States (IND 13977, [Chuang et al-2013](#Chuang_et_al_2013)), and a DNA vector encoding native sequence TRAP that expresses a protein very similar to the D-T proposed for this study has also previously been studied in the United States (IND 8687, [Hedstrom et al-1998](#Hedstrom_et_al_1998), [Richie et al-2012](#Richie_et_al_2012), [Wang et al-2005](#Wnag_et_al_2005)).

The ChAd63 products, ChAd63-C, ChAd63-A, ChAd63-T, have been studied in the United Kingdom, with oversight from the Medicines and Healthcare Products Regulatory Agency (MHRA). The ChAd63 CSP and AMA1 components have been studies in humans as individual vaccines ([Ogwang et al-2013](#Ogwang_et_al_2013), [O’Hara et al-2012](#OHara_et_al_2012), [Sheehy et al-2012](#Sheehy_et_al_2012)a).

Refer to the Investigator’s Brochure for additional information.

- 1. Known and Potential Risks and Benefits to Human Subjects
     1. Risks/Discomfort to Subjects and Precautions to Minimize Risk

Outlined below are anticipated adverse reactions, and a brief description of procedures to ameliorate risks and symptoms. All known risks and precautions described here are explained in detail in the informed consent document (Appendix A).

- - - 1. Local Reactions

Common local reactions previously reported following these investigational vaccines include self-limited redness, warmth, induration, swelling, pain, tenderness, ecchymosis, scaling, pruritus, and paresthesia at the injection site.

- - - 1. Systemic Reactions

Previously reported systemic reactions include headache, fever (objective or subjective), chills, rigors, myalgia, arthralgia, nausea, vomiting, diarrhea, abdominal pain, fatigue, malaise, headache, dizziness, cough, and a 'flu-like syndrome'. There is no risk of adenoviral infection in humans resulting from the administration of the ChAd63 vaccine. Simian adenoviruses are not known to cause pathology or illness in humans ([Tatsis-2007](#Tatsis_et_al_2007)). Additionally, the ChAd63 vectors are replication-deficient as the essential E1 gene region has been deleted so the virus can only propagate in cells expressing E1 functions ([Tatsis-2006](#Tatsis_et_al_2006)).

Subjects will be asked to remain at the Clinical Trials Center (CTC) for an observation period of at least 30 minutes to allow the study team to confirm that there are no immediate reactions following receipt of the vaccine. If any reactions do occur, appropriate supportive care will be provided according to standard operating procedures (SOPs), by certified study physicians and nurses using emergency supplies and equipment maintained at the sites of all study procedures including the NMRC CTC, WRAIR Insectary and CHMI follow-up hotel.

Subjects will be contacted by telephone within 24 hours after each immunization to assess symptoms. Subjects will be seen at the CTC on Day 2 post-immunization and approximately 7 days after each immunization and their memory aids will be reviewed and solicited AEs documented on the progress note. If memory aids are not completed or returned, an investigator will review the AEs that occur during that timeframe to the best of the subject’s recollection. At each of these visits, signs and symptoms will be monitored and safety testing may be performed. We will ensure that all subjects have a cell phone or access to a land line so that they may call study staff immediately, if needed. Subjects will be able to reach a CTC staff member by telephone at all times during the study.

- - - 1. Risks Associated with Use of the Needle-free Injection Device

The DNA priming vaccines will be administered by the IM route using the Biojector 2000^®^ needle-free injection device or an equivalent disposable syringe needle-free injection device. The Biojector 2000^®^ device was used in the D/HuAd5 vaccine clinical trial and demonstrated superior immunogenicity in previous NMRC clinical studies ([Wang et al-2001](#Wang_et_al_2001)), as well as good tolerability (Epstein et al-2002, [Richie et al-2012](#Richie_et_al_2012)). NMRC findings were concordant with those of the Vaccine Research Center at the NIH, which administered DNA priming immunizations for its HIV vaccines via IM Biojector ([Graham et al-2006](#Graham_et_al_2006), [Catanzaro et al-2007](#Catanzaro_et_al_2007)). Jet injection spreads the vaccine more widely through the tissue plains of the muscle, and this appears to increase cellular uptake and transgene expression. It may enhance the inflammatory response associated with injection in comparison with the small, relatively atraumatic depot of injectate that results from IM needle administration. This was consistent with the moderate intensity adverse events (AEs) recorded at the site of immunization when jet injection was used (although tolerability was still very good).

In the previous D/HuAd5-CA clinical study ([Chuang et al-2013](#Chuang_et_al_2013)), the most commonly noted AEs associated with the DNA priming vaccine administered with the Biojector 2000^®^ device were erythema (36% of all AEs), induration (28%), and pain (21%) at the injection site. Unsolicited localized AEs recorded during the 28 days after each immunization resolved rapidly without sequelae. In a clinical study in which the Biojector 2000^®^ was used to deliver DNA priming vaccine in a heterologous prime-boost HIV vaccine regimen, a small skin lesion, described as a papule or scab, at the immunization site, was commonly observed from Day 2 to Day 4 post-immunization and resolved without treatment ([Graham et al-2013](#Graham_et_al_2013)). The local AEs associated with the use of the Biojector 2000^®^ were pain/tenderness, swelling, and redness, most of which were mild in severity and all resolved without sequelae ([Graham et al-2013](#Graham_et_al_2013)). Mild bleeding or bruising has also been noted to occur.

- - - 1. Risks Associated with Controlled Human Malaria Infection (CHMI)

Some subjects are expected to develop malaria, and thus experience signs and symptoms of malaria which include fever, chills, rigors, headache, malaise, fatigue, dizziness, myalgia, arthralgia, nausea, vomiting, stomach cramps, diarrhea, tachycardia, moderate decrease in leukocytes and platelets, mild anemia, and, rarely, enlarged liver or spleen. The clinical outcomes of subjects post-challenge are discussed in detail in the published literature ([Epstein et al-2007](#Epstein_et_al_2007); [Roestenberg et al-2012](#Roestenberg_et_al_2012)).

There is also the possibility of complications of malaria, which are seen during naturally acquired malaria when diagnosis and treatment are delayed and high levels of parasitemia develop. In uncontrolled circumstances, malaria infections can lead to kidney, liver, or brain injury (seizures, coma) and death. Under the carefully controlled conditions and implementation of early diagnosis and antimalarial treatment in this study, the chance of such complications is unlikely and the risk of death from malaria infection is very small. There have been no cases with complications resulting in severe disability or death in study subjects undergoing CHMI. Subjects are monitored closely and treatment is initiated immediately upon identification of parasitemia.

Of note, cardiac events have been reported following CHMI in clinical trials in the Netherlands at Radboud University Nijmegen Medical Center (RUNMC). It remains unclear whether any of these events are related to CHMI or treatment of CHMI, but the details are provided below.

In 2008, a 20-year-old healthy female subject developed retrosternal chest pain 2 days after treatment with artemether/lumefantrine for parasitemia. A diagnosis of acute coronary syndrome with limited myocardial necrosis of the inferior wall was based upon the pain, < 1 mm ST segment elevation ECG findings and cardiac enzyme analysis. A cardiac MRI was negative for evidence of atherosclerotic disease. The subject recovered quickly and her follow-up was uneventful. It is unclear if this subject’s AE was the result of the experimental vaccine, malaria infection, anti-malarial medication, or an unknown cause ([Nieman et al-2009](#Niemanetal2009)).

The AE resolved following treatment and did not re-occur in the 1-year follow-up period. A definite relationship between the cardiac event and the experimental infection or its treatment was not established.

A second cardiac event occurred at this same site in the Netherlands in a subject who participated in a trial in which the PfSPZ challenge was administered with chloroquine prophylaxis as a vaccine approach (the PfSPZ-CVac approach). Here, 1 subject experienced an episode of myocarditis (serious adverse event) following treatment for malaria. The subject was hospitalized due to increasing troponin levels without any symptoms, and had a single short-lived episode of chest pain that was treated with nitroglycerin; his magnetic resonance imaging (MRI) showed evidence of myocarditis. Specifically, the SAE occurred in the subject who underwent CHMI on Day 124 using 5 Pf NF54-infected mosquitoes. The CHMI occurred 60 days after he had received the last injection of PfSPZ Challenge for immunization. On Day 9 post-CHMI the subject had a sore throat. On Day 11 post-CHMI, the subject’s thick film was positive for malaria, and treatment with atovaquone-proguanil was initiated. The subject was asymptomatic, but due to elevated troponin T levels (maximum: 299 ng/L), which are routinely assessed at RUNMC on a daily basis, the subject was hospitalized on Day 13 after CHMI. On Day 14 after CHMI, the subject experienced chest pain for 10 minutes and received sublingual nitroglycerin spray. He was diagnosed with myocarditis based on a magnetic resonance imaging (MRI) and minor repolarization disturbances on electrocardiogram. The subject had no further symptoms on follow-up, and the troponin T levels returned to normal by Day 28 post CHMI. The subject was discharged on Day 17, and the electrocardiogram was normal on Day 31 post CHMI. A follow-up cardiac MRI, performed approximately 5 months after the start of the SAE, demonstrated good left ventricular function with mild hypokinesia in a few segments and some remaining mid-wall delayed enhancement. An etiology was not established, but the subject’s throat swab was positive for rhinovirus 9 days after CHMI. Furthermore, 14 days after the third immunization with PfSPZ Challenge, the subject received immunizations for diphtheria, tetanus, polio, typhoid, hepatitis A and hepatitis B in preparation for international travel. The troponin levels returned to normal after 8 days and there was normal cardiac function. The SAE occurred 73 days after the last administration of PfSPZ Challenge, 13 days after CHMI with Pf-NF54 infected mosquitoes, and 2 days after the start of treatment for malaria with Malarone (atovaquone/proguanil). Whether or not these cardiac events are related to CHMI itself remains unclear ([Van Meer et al-2014](#Vanmeeretal2014)).

Most recently, a third (unpublished) cardiac event took place at RUNMC. On November 21, 2014, a 23 year old male subject had an asymptomatic high sensitive (hs)-troponin-T elevation 10 days after the first immunization with bites from 15 NF54 infected mosquitoes under chloroquine prophylaxis. The patient was admitted to the hospital for cardiac monitoring during 2 days, after which he was discharged in good clinical condition. During the 48 hour observation period, the patient remained asymptomatic, hs-troponin-T was maximally 168 ng/l (normal value < 14 ng/l), and ECG and cardiac monitoring showed no abnormalities. Urine toxicology was positive for cannabis and a cardiac MRI showed findings that could be consistent with a myocarditis, but may represent artifact due to movement. In conclusion, elevated hs-troponin-T concentrations and cardiac MRI findings likely represent a mild myocarditis in an apparently healthy male subject 10 days after a first CPS-immunization per protocol. After thorough study of the event, the Data Safety and Monitoring Board (DSMB) for this trial and the Central Committee on Research Involving Human Subjects (CCMO) in the Netherlands approved the continuation of the clinical trial on December 8, 2014.

While there are several confounding factors associated with each of these cardiac events, nevertheless a causal relationship with CHMI cannot be excluded and therefore the design of the trial will incorporate safeguards to minimize to the extent possible the risk of cardiovascular events in study participants. Enrolled participants will be at low risk of cardiovascular events due to their age, absence of known cardiovascular disease, absence of known diabetes mellitus, and documented normal ECG, systolic and diastolic blood pressure at screening. During the study, participants will be closely followed for the occurrence of cardiovascular-related signs or symptoms and signs or symptoms of an event suggestive of a cardiac etiology will prompt a cardiology work-up.

The WHO Consensus Document for the Standardization of Design and Conduct of *P falciparum* Sporozoite Challenge Trials ([Laurens et al-2012](#Laurensetal2012)) will be utilized to help ensure the safety of all subjects from the day of challenge until completion of all post-challenge follow-up. The safe use of malaria challenge via mosquito bite in 118 subjects by NMRC through 1992 has been summarized ([Church et al-1997](#Churchetal1997)). A more recent review corroborates the same level of safety with further challenges by mosquito bite conducted through 2007 ([Epstein et al-2007](#Epstein2007)).

Relapsing malaria is not an issue since Pf has no hypnozoite stage and therefore does not relapse after appropriate treatment. The mosquitoes used for CHMI are raised in a closed insectary colony at NMRC/WRAIR in Silver Spring, Maryland to minimize the risk of infection with any vector or blood-borne disease. In the insectary, the mosquitoes for challenge are infected with malaria by feeding on malaria-infected human blood. To minimize the risk of disease transmission the following precautions are taken:

- The blood is obtained from commercial sources and has been tested for syphilis, hepatitis B virus, hepatitis C virus, and human immunodeficiency (HIV) virus
- The length of time from when the mosquitoes feed on the infected blood until they feed on the subject is 17-23 days, decreasing any chances of transmitting an infectious agent

There has been no reported transmission of human viruses, including HIV or hepatitis, using this system. Additionally, mosquitoes are known to digest hepatitis B surface virions within 2 to 3 days of ingestion.

The clinical study team will review each subject's adherence to the schedule and safety follow up to date. This review will be done in order to identify any likelihood that the subject may be unreliable or non-compliant with study visits post-challenge. A subject who has been non-compliant with prior study visits may be excluded from the challenge phase and followed for safety.

Study subjects will be counseled at the time of challenge about the signs and symptoms of malaria and given 24 hour per day contact information should any signs or symptoms develop. Prior to the challenge, at least 2 emergency contact numbers will be confirmed for each subject. Subjects will be monitored closely, especially following the challenge with viable sporozoites. As soon as malaria infection is diagnosed in immunized subjects or infectivity controls, the subject will be treated as described in Section 8.2. Prompt treatment minimizes the risk of developing a serious complication due to malaria infection.

Subjects will receive instructions not to travel outside of the Washington, DC, metro area from the day of challenge to 4 weeks after challenge. If a subject must travel and is assessed to be at risk of developing malaria, he or she will be clinically evaluated by the principal investigator and he/she may be presumptively treated for malaria infection and proper arrangements will be made to ensure adequate follow-up. To ensure appropriate and safe follow-up, subjects may be asked to make or change travel arrangements; study funding will provide compensation and/or cover associated costs.

For any subject that must travel at a critical point, especially a travel decision shortly before CHMI, the PI or his/her designee will perform risk assessment for the subject, and the possibility of proceeding to CHMI and presumptive treatment may be considered. A subject needing travel during the critical period would be counseled, and the PI or designee, Research Monitor, Sponsor, and the IRB Chair or Vice Chair would discuss and decide whether to proceed to CHMI and treatment.

- - - 1. Risks Associated with Anti-malarial Medications

The primary anti-malarial medication will be Malarone (atovaquone/proguanil), as the 3D7 clone of the NF54 strain is highly sensitive to Malarone. Malarone is a generally well tolerated and highly effective treatment regimen. It has only been rarely associated with severe adverse reactions. The following adverse reactions have been reported in patients being treated with Malarone for malaria:

- Abdominal pain
- Nausea
- Vomiting
- Headache
- Transient elevations of liver-associated enzymes

Subjects who cannot tolerate Malarone will be given Coartem. Coartem is a fixed dose combination of 2 drugs: artemether and lumefantrine. Reported adverse reactions include the following:

- Headache
- Dizziness
- Loss of appetite
- Fever
- Chills
- Abdominal pain
- Weakness
- Myalgias
- Arthralgias
- Nausea
- Vomiting
- Sleeping disturbances
- Cough

Chloroquine will serve as the third line anti-malarial treatment, to be used if there are reported or known adverse effects from the other agents. Reported side effects of chloroquine are as follows:

- Abdominal cramps
- Anorexia
- Diarrhea
- Nausea
- Vomiting
- Headache
- Insomnia
- Dizziness
- Blurred vision
- Pruritus

Maculopathy and macular degeneration have been reported in persons receiving long term or high doses of chloroquine; however this serious side effect seems unlikely during its use as routine treatment for malaria infection. Chloroquine has been reported to exacerbate psoriasis and has been associated with tinnitus or reduced hearing acuity in persons with pre-existing auditory damage. Chloroquine should be used with caution in subjects with known glucose‑6 phosphate dehydrogenase (G6PD) deficiency.

The study team will discuss these medications and their possible side effects with study subjects both as part of the informed consent process and prior to initiation of treatment for subjects who are infected with malaria. Intolerable side effects will be managed by switching to an alternative regimen.

- - - 1. Pregnancy and Lactation

The risk of study participation to the fetus or a breastfeeding child is unknown; therefore pregnant and lactating females will be excluded from this study. Study subjects, both male and female of childbearing potential, must agree to consistently use effective means of birth control throughout the duration of the study.

- Sexually active females, unless surgically sterile or post-menopausal, must use an effective method to avoid pregnancy (including oral or implanted contraceptives, intrauterine device, female condom, diaphragm with spermicide, cervical cap, abstinence, use of a condom by the sexual partner or surgical sterilization of sexual partner) from 14 days prior to the first immunization throughout the study and at least 6 months after the last study visit.
- If female subjects are unable to bear children due to menopause or have had a procedure performed (tubal ligation or hysterectomy), a medical note from her physician is required. If post-menopausal, female subjects must have experienced at least 1 year of amenorrhea.
- Sexually active men must agree to use effective means of birth control (including condom use or vasectomy) from the day of the first immunization through the end of the study (the last study visit which occurs at 3 months after CHMI).

If a female subject becomes pregnant, either during the study or within 30 days of the study completion, the subject will be instructed to notify a clinical investigator. A subject who becomes pregnant during the study may be asked to complete study procedures such as medical history, physical examination, and laboratory testing for safety and immunogenicity at the discretion of the Principal Investigator. The research monitor will be notified about the pregnancy and may recommend additional safety evaluations, as appropriate (see Section 11.4.2.1 for reporting requirements related to the occurrence of pregnancy in a subject).

- - - 1. Risks Associated with Venipuncture/Blood Drawing

At specified points throughout the study, blood samples will be collected from the subject for safety and immunologic testing. (See Section 6.2 Overall Study Design, Table 5, Table 6, and Table 7) There are risks associated with blood drawing that include discomfort, swelling, bruising around the vein, lightheadedness/fainting, anemia following repeated blood draws, and, rarely, infection at the blood-drawing site or clinically significant hematoma.

Venipuncture will be done by trained staff using aseptic technique in a designated area, to reduce the risks of complications. Subjects will be counseled to return to the enrollment site if infection or any other unexpected outcome is suspected.

The amount of blood collected will be monitored closely. Throughout this study, the amount of blood collected will be no more than 5 mL/kg (250 mL for a 50 kg subject) during a 24‑hour period and 10.5 mL/kg (525 mL for a 50 kg subject) in any 8-week period following the guidelines of the American Association of Blood Banks. Complete blood counts will be performed periodically to monitor hemoglobin levels.

- - - 1. Allergic Reaction

As with any investigational new drug (IND) product administration and no matter what precautions are taken, there is always the risk of a serious, or even life-threatening, allergic reaction. Subjects with allergies to any component of the vaccine formulation or who have had serious adverse reaction to other vaccines or injectable products (such as hives, anaphylaxis, respiratory difficulty, angioedema, or abdominal pain) will be excluded from the study.

During the manufacturing process of Oxford's ChAd63 CSP, AMA1, and ME-TRAP/SSP2 vectors, a biocide named Kathon is used (active ingredients: 2 isothiazolinones, 5-chloro-2-methyl-4-isothiazolin-3-one, and 2-methyl-4-isothiazolin-3-one). Kathon is added to body washes, conditioners, liquid soaps, shampoos, and wipes as a preservative. The maximum dose is 0.1% for 'rinse off' products and 0.05% for “leave-on” products. It has been approved by regulatory authorities throughout the world as a preservative in these products. As a skin sensitizer, it is known to cause contact dermatitis. An internal study was set up to quantify the levels of Kathon that were removed during the final purification step of buffer exchange during the manufacture of the ChAd63 vaccines. The study utilized high-performance liquid chromatography and showed that trace amounts of Kathon may be left on the desalting column after carrying out the rinse and sanitization steps. However, the study confirmed greater than 99.9975% removal of Kathon to approximately 30-fold less than the limits for “leave-on” products containing Kathon. Because small residual amounts may be present, we will exclude from immunization anyone with a history of clinically significant contact dermatitis or sensitivity to Kathon.

There is also always the risk of a serious, or even life-threatening, allergic reaction to mosquito bites or anti-malarial drugs. Subjects with a history of anaphylactic response to mosquito bites or known allergy to chloroquine phosphate or atovaquone/proguanil antimalarials will be excluded from the study.

Study physicians will be available to manage emergencies which may arise at the CTC. Emergency equipment is centrally located in the NMRC CTC where subjects will be observed for a minimum of 30 minutes following receipt of immunization. Refer to CTC SOP titled "Anaphylaxis Treatment."

Medical emergency equipment will be available during challenges at the WRAIR/NMRC insectary and at the hotel post challenge. There will always be a physician on-site during these procedures and for at least 30 minutes post-challenge. Topical steroids and antihistamines will be made available to treat pruritus.

- - - 1. The Risk of HIV Infection

In 2013, the National Institute of Allergy and Infectious Diseases (NIAID), part of the National Institutes of Health, held a scientific meeting to examine why certain investigational HIV vaccines using the HuAd5 vector may have increased susceptibility to HIV infection (NIAID-2013). During this meeting, data from multiple studies were presented and discussed. The findings were summarized in a recent perspectives article in the journal Science ([Fauci et al- 2014](#Fauci_et_al_2014)), the authors use the wording “rAd5” in place of HuAd5:

“Between 2005 and 2013, two rAd5 vaccines for HIV were assessed in 3 efficacy studies ([Buchbinder et al-2008](#Buchbinder_et_al_2008), [McElrath et al-2008](#McElrath_et_al_2008), [Gray et al-2011](#Gray_et_al_2011)). The first study (Step), using 3 doses of the Merck rAd5 vaccine containing genes encoding 3 HIV-1 proteins (gag, pol, and nef), was stopped for futility. In addition, a statistically significant trend toward increased HIV infections in vaccine recipients was observed ([Buchbinder et al-2008](#Buchbinder_et_al_2008), [Duerr et al-2012](#Duerr_et_al_2012)). The group at highest risk was uncircumcised men who both had sex with men (MSM) and had high titers of preexisting antibodies against Ad5. The following Phambili trial of the same Merck rAd5 vaccine, conducted in South Africa, was closed and unblinded early during the enrollment period. Few participants received the planned 3 doses of vaccine. Analysis of the data showed no increased risk of HIV infection ([Gray et al-2011](#Gray_et_al_2011)). However, data from the long-term unblinded follow-up of Phambili participants suggested an increased risk of infection in vaccinated men relative to unvaccinated controls ([Gray et al-2014](#Gray_et_al_2014)). In 2009, a different rAd5 vaccine was tested in the HIV Vaccine Trial Network (HVTN) 505 trial. It contained 3 doses of a DNA prime (a plasmid of non-HIV DNA and certain HIV genes) followed by a single boost of rAd5 expressing HIV envelope and viral structural antigens. The study restricted enrollment to circumcised MSM who lacked preexisting antibodies to Ad5, because no level of increased risk had been seen in this group in the Step trial ([Duerr et al-2012](#Duerr_et_al_2012)). The HVTN 505 trial was halted prematurely because it met futility criteria; however, there was no evidence of increased risk of HIV infection in the vaccinated subjects ([Hammer et al-2013](#Hammer_et_al_2013a)).

Results of a Meta-Analysis: A meta-analysis of the Step, Phambili, and HVTN 505 trials was performed by statisticians from the HVTN Statistics and Data Management Center and from NIAID. Combining data from the 3 studies, there was an overall hazard ratio of 1.33 (P < 0.01) associated with vaccination. However, almost all of the increased risk of HIV acquisition was driven by the Merck vaccine (Step and Phambili: hazard ratio = 1.41, P = 0.005) with the Step trial contributing most infection endpoints. HVTN 505 considered alone did not show any trend toward infection risk ([NIAID-2013](#NIAID_2013), [Hammer et al-2013](#Hammer_et_al_2013a)). It could not be determined whether the lack of increased susceptibility in the latter trial was due to population or regimen (inclusion of env, DNA prime, and single rAd5 boost with differences in vector backbone).”

Pre-existing immunity to recombinant HuAd5 vector has been shown to decrease antigen expression that leads to suboptimal conditions for the induction of CD8+ T cell responses critical for inducing protection against malaria. To address the potential limitations of adenoviral vaccine candidates based on HuAd5, including the potential increased risk for the acquisition of HIV infection, a recombinant adenoviral vector from lower seroprevalence nonhuman sources, such as the Chimpanzee Adenovector Serotype 63 or ChAd63 will be evaluated in this clinical study. Currently, there is no data to suggest any significant cross-reactivity between antibodies to HuAd5 and ChAd63. Additionally, the low seroprevalence of the chimpanzee-derived ChAd63 in human cohorts from diverse geographic regions has been demonstrated ([Quinn et al-2013](#Quinn_et_al_2013)).

- - - 1. Risks associated with Physiological Status Monitoring

Subjects will be asked to wear a commercial off-the-shelf smartwatch (Samsung Gear S3 or equivalent) for at least 12 hours a day for approximately 6 weeks. The smartwatch is largely indistinguishable from a standard wristwatch in size and use and some people may experience discomfort or skin irritation when wearing the smartwatch for long periods of time, particularly those who wear the watch too tightly or have sensitive skin. Subjects who experience prolonged discomfort are asked to contact the Clinical Trial Center to identify potential solutions, such as loosening the strap or switching the hand the watch is worn. For subjects whose discomfort continues even after attempting recommended remedies will be asked to discontinue use of the device for the remainder of the study.

There is also a risk of erroneous or inaccurate heart rate readings, particularly in situations of improper wear or moisture or in certain changes in activity, such as intensive exercise. These high or low rates could potentially cause concern to subjects, who might be concerned about this reflecting a disease state or heart problem. Description of the function of the device and this sort of reading will be explained early in the training process, but it is a well-known occurrence in a number of photoplethysmography based heart rate measuring devices. Subjects with concerns about the readings are asked to call the CTC or the PI with concerns about the readings.

The heart rate analyses will only be available retrospectively, at times when subjects deliver the phone for data retrieval. Adverse event recording in terms of abnormal PSM values, with heart rates specifically, will be based on subject visits or subject reported events as specified elsewhere in the protocol, for example a subject stating persistent tachycardia at rest or demonstrating it at a visit, except where this may be expected during CHMI. Subjects are instructed in these limitations during the consent process.

- - - 1. Unknown Risks

Furthermore, there is the remote possibility of unknown risks that cannot be foreseen based on current information. This would include late effects that have been seen with some vaccines.

- - - 1. Alternatives to this IND Product or Study

An alternative is not to participate in this study.

- - 1. Intended Benefit for Subjects

There is no direct benefit to subjects in this study. There is the potential indirect benefit of gaining increased personal knowledge about their health status from their medical history, physical examination and laboratory testing.

- - 1. Risks to the Study Personnel and the Environment

The principal risk in the clinical setting is in the handling of needles that may be contaminated and the attendant risks including hepatitis, HIV, and other human pathogens. Adherence to SOP for working with infectious agents and universal precautions will reduce the risk of exposure. Subjects will be screened for HIV, hepatitis B and hepatitis C for both assessment of suitability of study participation as well as for protection of laboratory and health care personnel. Standard procedures will be followed for handling blood and body fluid specimens.

There are no known risks to the environment other than those associated with the generation of biohazardous waste attendant to venipuncture and vaccination of humans. All biohazardous waste will be disposed of as stipulated by local, state, and Federal regulations and in accordance with study site SOPs.

Since CHMI involves the bites of mosquitoes, there is the risk of release of mosquitoes into the environment that carry fully infectious malaria. However, all mosquitoes that will be used will be kept in secure containers inside the insectary. Since the institution's initiation of mosquito husbandry, no such release of mosquitoes has ever been detected. This storage of mosquitoes also limits the risk of study personnel being bitten by malaria-infected mosquitoes during challenge. If an exposure were to occur, standard SOPs would be followed, and the exposed person would be treated with Malarone if a significant risk had been incurred.

The risk of accidentally transmitting malaria to a person in the community will be negligible; infected mosquitoes will be restricted to the insectary with only temporary excursions to rooms outside of the insectary (eg, for radiation) in which case the mosquito transport containers will not be opened. All infections in subjects will be treated promptly before gametocytes can develop, thus eliminating the potential for human to mosquito transmission.

- 1. Investigational Product Preparation, Route of Administration, Dosage Regimen, Treatment Period, and Justification
     1. Investigational Product Preparation and Route of Administration

On the day of immunization, the vaccines will be prepared/mixed by appropriately trained personnel in a sterile biosafety cabinet. A summary of the vaccine preparation for each regimen to be used is provided here. More specific details are provided in study specific procedures (SSPs) for the preparation of the investigational products. The DNA vaccine components must be administered within 8 hours of thawing. The ChAd63 vaccine components must be administered within 1 hour of removal from the freezer.

- - - 1. DNA Vaccine

The DNA constructs and study diluents will be allowed to equilibrate to room temperature prior to mixing for injection. Any vaccine not used within 8 hours of thawing will be discarded according to the sponsor’s instructions and will be recorded as such for monitoring and accounting purposes. The products will be thawed, combined, and mixed with study diluent in a properly labeled mixing vial.

- - - - 1. D-CA

The D-CA component will be administered at 2 mg (1 mg D-CA/mL), split into two 1mL intramuscular injections to each deltoid muscle by Biojector^®^ 2000 needle-free injection device or an equivalent disposable syringe needle-free jet injection device at each priming immunization. Each subject receives 1 mg of D-C and 1 mg D-A pDNA constructs (2 mg D-CA total) per immunization. Briefly, each construct is vialed separately at 3 mg/mL, 0.5 mL/vial. Equal volumes (and mg) of each pDNA product will be placed into a vial and mixed to formulate the D-CA vaccine. An appropriate amount of study diluent will be added for a final total D-CA volume of 2.4 mL, sufficient for 2 injections of 1 mg/mL of D-CA (0.5 mg each D-C and D-A per mL into each deltoid).

- - - - 1. D-CAT

The D-CAT component will be administered at 3 mg (1.5 mg D-CAT/mL), split into two 1mL intramuscular injections to each deltoid muscle by Biojector^®^ 2000 needle-free device or an equivalent disposable syringe needle-free jet injection device at each priming immunization. Each subject receives 1 mg of D-C, 1 mg D-A, and 1 mg D-T pDNA products (3 mg D-CAT total) per immunization. Briefly, each construct is vialed separately at 3 mg/ml, 0.5mL/vial. Equal volumes (and mg) of each pDNA product will be placed into a mixing vial and mixed to formulate the D-CAT. An appropriate amount of study diluent will be added for a final total D-CAT volume of 2.4 mL, sufficient for 2 injections of 1.5 mg/mL of D-CAT (0.5 mg each D-C, D-A, and D-T per mL into each deltoid).

- - - 1. Chimpanzee Adenovirus-Vectored Vaccine
         1. ChAd63-CA and ChAd63-CAT

The ChAd63 adenovectored vaccine components must be administered within 1 hour of removal of the constructs from the freezer. Any vaccine not used within the 1 hour period will be discarded and recorded as such for monitoring and accounting purposes.

- - - - 1. ChAd63-CA

The dose of ChAd63-CA vaccine component to be administered is 1.0 x 10^11^ vp/dose (5 x 10^10^ vp/construct). Once the product vials are thawed, appropriate volumes of each of the products will be combined in a mixing vial. This vaccine mixture prepared per SSP will provide 1 dose containing 5 x 10^10^ vp of ChAd63-C and 5 x 10^10^ vp of ChAd63-A and will be administered by standard IM injection with a needle and syringe into the deltoid muscle of the non-dominant arm unless there is a compelling reason to use the dominant arm, at which point the decision will be deferred to PI discretion.

- - - - 1. ChAd63-CAT

The dose of ChAd63-CAT vaccine regimen to be administered is 1.5 x 10^11^ vp/dose (5 x 10^10^ vp/construct). Once the vials are thawed, appropriate amounts of each product will be mixed in a mixing vial. This vaccine mixture prepared per SSP will provide 1 dose containing 5 x 10^10^ vp of each of the constructs, ChAd63-C, ChAd63-A, and ChAd63-T. The vaccine will be administered by standard IM injection with a needle and syringe into the deltoid muscle of the non-dominant arm unless there is a compelling reason to use the dominant arm, at which point the decision will be deferred to PI discretion.

- - 1. Dosage

The vaccine doses to be used in this study are summarized in Table 3.

Table 3: Vaccine Doses

| Group | Vaccine (Components) | No of Doses | No of Injections per Dose | Total Dose |
| --- | --- | --- | --- | --- |
| 1 | D-C + D-A (prime) | 3 | 2 | 1 mg per construct,  2 mg total per dose, 1 mL injectate into each deltoid |
|  | ChAd63-C + ChAd63-A (boost) | 1 | 1 | 5 x 10^10^ vp per construct,  1.0 x 10^11^ vp total dose, 0.65 mL injectate into 1 deltoid |
| 2 | D-C + D-A + D-T (prime) | 3 | 2 | 1 mg per construct,  3 mg total per dose, 1 mL injectate into each deltoid |
|  | ChAd63-C + ChAd63-A + ChAd63-T (boost) | 1 | 1 | 5 x 10^10^ vp per construct,  1.5 x 10^11^ vp total dose, 1 mL injectate into 1 deltoid |

- - 1. Justification

Dose selection for each of the products is based upon safety, immunogenicity, and efficacy data from previous clinical trials (Section 4.5).

DNA dose selection for the priming vaccines D-C and D-A is based upon the safety, immunogenicity and protection data generated from the D/HuAd5 Trial ([Chuang et al-2013](#Chuang_et_al_2013)). The 2 plasmid DNA products were administered at a dose of 2 mg per administration (1 mg each formulation) intramuscularly by needle-free jet injection as two 1 mL injections, one to each arm. The concentration of the mixed plasmid DNA was 1 mg/mL.

Although the use of a codon optimized version of D-T will be first in humans, the native sequence D-T plasmid has been safely administered in humans in the USA at a dose of 0.5 mg administered 3 times as part of the MuStDO5 trial ([Richie et al-2012](#Richie_et_al_2012)). The native sequence D-T has also been safely administered to humans in the UK at doses up to 2 mg (administered 3 times). The TRAP products encoded by the codon optimized and native sequence D-T plasmids are the same.

Dose selection for the ChAd63 boost products is based upon the experience in the UK. Two doses of ChAd63-C that have been studied (5 x 10^9^ vp and 5 x 10^10^ vp) were selected based on safety and immunogenicity data generated from 54 healthy individuals who safely received ChAd63-vectored vaccines encoding other malaria antigens (ie, TRAP, AMA1, and MSP1) ([deBarra et al-2014](#deBarra_et_al_2014); [Hodgson et al-2014](#Hodgson_et_al_2014)). Similarly, ChAd63-A had been administered to 83 malaria-naïve healthy subjects in 4 clinical trials in Oxford ([Sheehy et al-2012a](#Sheehy_et_al_2012); [Sheehy et al-2012b](#Sheehy_et_al_2012b); [Hodgson et al-2014](#Hodgson_et_al_2014)). Two doses of ChAd63-A were clinically assessed: 5 x 10^9^ vp and 5 x 10^10^ vp. Last, ChAd63-T had been administered to 464 healthy children and infants in The Gambia and Burkina Faso, at doses of 1 x 10^10^ vp and 5 x 10^10^ vp IM, and has been well tolerated ([Ogwang et al-2013](#Ogwang_et_al_2013)). The 5 x 10^10^ vp dose per construct has been shown to be the optimal dose for ChAd63-vectored vaccines, inducing potent immunogenicity and showing a good a reactogenicity profile. All vaccinations with ChAd63 and MVA vectors had been administered IM given the proven favorable safety and immunogenicity profiles of this route of administration with these vectors.

- 1. Compliance Statement

The study will be conducted according to the protocol and in compliance with International Conference on Harmonization (ICH) Good Clinical Practice (GCP), Belmont Principles, and other applicable regulatory and Department of Defense (DoD) requirements. All identified study personnel will be trained to perform their roles and will carry out their responsibilities in accordance with ICH GCP guideline and clinic site SOPs.

- 1. Study Population

The study population will consist of healthy, malaria naive, men and women ages 18 through 50, inclusive, at the time of enrollment, and will be recruited from the Baltimore-Washington, DC metropolitan area. Up to 59 study subjects may be enrolled. Refer to Section 12.1.6 for the sample size justification. Enrollment will be open to both civilian and military personnel (active duty, reservists, military dependents, and non-military civilians).

In order to enroll the required number of eligible subjects (40 subjects will be immunized; 12 subjects will not be immunized and serve as infectivity controls; 4 subjects will be alternates for Groups 1, and 2; 3 subjects will be alternates for the infectivity control group on day of CHMI), approximately 236 subjects will be screened. The screened to eligible subject ratio of 4:1 is based on our previous experience in recruiting for malaria vaccine clinical trials in this study population. In this study, a subject is defined as being enrolled after he or she has met all of the inclusion criteria, does not have any of the exclusion criteria, and undergoes the first post-screening study specific procedure (first dose of DNA vaccination for the immunized group; CHMI for the infectivity controls).

- 1. Study Site

The study will be conducted at the NMRC CTC, building 17B at the Naval Support Activity Bethesda, on the same campus as but not directly affiliated with WRNMMC in Bethesda, Maryland. The NMRC CTC is equipped with vaccine storage and mixing capabilities and clinical rooms for subject assessment/evaluation, phlebotomy, and vaccine administration. All visits other than the challenge visits and hotel stay will take place at the NMRC CTC. Challenge procedures will be conducted at the WRAIR Insectary, 503 Robert Grant Avenue, Silver Spring, Maryland. The post-challenge overnight stays will occur in a pre-determined hotel in close proximity to the NMRC CTC. The hotel is to be determined. Subjects will be eligible for emergency medical care at the WRNMMC, and EMS transport is available from all locations.

Subjects can access the NMRC CTC either by car or by Metro. The nearest Metro station is conveniently located directly across the street from the WRNMMC Campus. A NMRC staff member is designated as the dedicated driver for any subjects who require transportation from the front gate of the WRNMMC base to the NMRC CTC.

Trial Objectives and Purpose

The design of the proposed study is summarized in Figure 3 and Table 4. A total of 40 subjects will receive 3 priming immunizations at Weeks 0, 4, and 8 followed by a boosting immunization on Week 24 (approximately 16 weeks after the third priming immunization dose).

At Week 28, the immunized subjects from the 2 immunized groups and the 12 infectivity controls will undergo CHMI by mosquito bite using either the 3D7 (a clone of NF54) or NF54 Pf strains. The 3D7 strain will be preferably used to facilitate comparability of the findings from this study with those from previous NMRC clinical trials in which the same Pf strain was used.

- 1. Primary Objectives

The primary objectives are to:

- Assess the safety and tolerability of a heterologous prime-boost regimen expressing CSP (C) and AMA1 (A) antigens through a DNA vaccine prime (D-CA) with chimpanzee adenovirus 63 vaccine boost (ChAd63-CA) in healthy malaria-naïve adults.
- Assess the safety and tolerability of a heterologous prime-boost regimen expressing CSP (C), AMA1 (A), and TRAP (T) antigens through a DNA vaccine prime (D-CAT) with chimpanzee adenovirus 63 vaccine boost (ChAd63-CAT) in healthy malaria-naïve adults.
  1. Secondary Objectives

The secondary objectives are to:

- Assess the protective efficacy of 2 heterologous prime-boost regimens (D/ChAd63-CA, and D/ChAd63-CAT) in healthy malaria-naïve adults against a CHMI with Pf (3D7 or NF54 strain) sporozoites administered by mosquito bites.
- Assess the cellular immunogenicity of these prime-boost regimens to CSP, AMA1 and TRAP antigens by cytokine FluoroSpot and flow cytometry assays
- Assess the humoral immunogenicity of these prime-boost regimens by: Enzyme linked immunosorbent assay (ELISA) to PfCSP NANP repeat peptide, N- and C-terminal peptides, and full length recombinant protein; to PfAMA1 recombinant protein; and to Pf TRAP recombinant protein
- Analysis of immunofluorescence assay (IFA) titers against sporozoite and erythrocyte stage parasites
- Assess the association between the subjects’ pre-ChAd63 immunization neutralizing antibody titers to HuAd5 and the protective efficacy and humoral and cellular immunogenicity, of these prime-boost regimens to CSP, AMA1 and TRAP as measured by ELISA and FluoroSpot assays
- Assess the rate of seroconversion to HuAd5 among subjects who are immunized with the ChAd63 boost vaccine.
- Compare the safety, tolerability, immunogenicity, and protective efficacy of D/ChAd63-CA vs. D/ChAd63-CAT
  1. Exploratory Objectives
- Collect continuous vital sign data before, during, and after malaria infection using a non-invasive wearable device in infectivity control subjects.

Trial Design

- 1. Study Endpoints
     1. Primary Endpoints

The primary endpoints are:

- Occurrence, severity, and duration of solicited adverse events following immunization through Day 7 after each immunization
- Occurrence, severity, and duration of unsolicited adverse events, abnormal physical findings, and abnormal laboratory values following immunization through Day 28 after each immunization
- Occurrence of any serious adverse events, as defined in 21 CFR 312.32 throughout the study period, from enrollment through 3 months after CHMI
  - 1. Secondary Endpoints

The secondary endpoints are:

- Vaccine efficacy as determined by protection from the development of parasitemia and time to development of parasitemia, as measured by microscopic examination of thick smears and by PCR after CHMI and by PCR analysis conducted retrospectively
- Analysis of IFN-γ, interleukin (IL)-2, IFN-γ + IL-2, Granzyme B, and IFN-γ + Granzyme B cytokine secretion in response to stimulation with synthetic peptides derived from PfCSP, PfAMA1, and Pf-TRAP by FluoroSpot assay using peripheral blood mononuclear cells (PBMC)
- Analysis of IFN-γ, TNF-α, and IL-2 cytokine secretion by intracellular cytokine staining (ICS) using multi-parameter flow cytometry in response to stimulation with synthetic peptides derived from PfCSP, PfAMA, and Pf TRAP using PBMCs
- Enzyme linked immunosorbent assay (ELISA) to PfCSP NANP repeat peptide, N- and C-terminal peptides, and full length recombinant protein; to PfAMA1 recombinant protein; and to PfTRAP recombinant protein
- Measurement of antibody titers against sporozoite and erythrocyte stage parasites by immunofluorescence assay (IFA) using sera/plasma
- Measure of correlation between pre-ChAd63 immunization HuAd5 neutralizing antibody titers and the protective efficacy against CHMI and humoral and cellular immune responses of the 2 prime-boost regimens
- Comparison of safety, immunogenicity, and vaccine efficacy of the 2 heterologous prime-boost vaccine regimens (D/ChAd63-CA vs. D/ChAd63-CAT)
  1. Overall Study Design

The trial design is illustrated in Figure 3 and summarized in Table 4. Detailed study event schedules are provided in Table 5, Table 6, and Table 7.

Figure 3: Study Design

Table 4: Study Design by Group

| Group | Week | Event |
| --- | --- | --- |
| 1 (n=20) | 0 | Prime with D-CA (#1) |
|  | 4 | Prime with D-CA (#2) |
|  | 8 | Prime with D-CA (#3) |
|  | 24 | Boost with ChAd63-CA |
|  | 28 | CHMI |
| 2 (n=20) | 0 | Prime with D-CAT (#1) |
|  | 4 | Prime with D-CAT (#2) |
|  | 8 | Prime with D-CAT (#3) |
|  | 24 | Boost with ChAd63-CAT |
|  | 28 | CHMI |
| Infectivity Control (n=12) | 28 | CHMI |

Table 5: Study Event Schedule and Procedures from Day -90 through Day 195 for Immunized Subjects

| Procedure | Screening | Pre-Imm | DNA #1 | Follow-up | | | DNA #2 | Follow-up | | | DNA #3 | Follow-up | | | | | Ad | Follow-up | | |
| --- | --- | --- | --- | --- | --- | --- | --- | --- | --- | --- | --- | --- | --- | --- | --- | --- | --- | --- | --- | --- |
| Visit Number | 1 | 2 | 3 | 4 | 5 | 6 | 7 | 8 | 9 | 10 | 11 | 12 | 13 | 14 | 15 | 16 | 17 | 18 | 19 | 20 |
| Day relative to 1st immunization a | -90 to -8 | -14 to -1 | 0 | 2 | 7 | 14 | 28 | 30 | 35 | 42 | 56 | 58 | 63 | 70 | 84 | 126 | 168 | 170 | 175 | 195 |
| Window |  | ±7 |  | ±1 | ±3 | ±5 | ±7 | ±1 | ±3 | ±5 | ±7 | ±1 | ±3 | ±5 | ±5 | ±5 | ±7 | ±1 | ±3 | ±7 |
| Day relative to each immunization |  |  | 0 | 2 | 7 | 14 | 0 | 2 | 7 | 14 | 0 | 2 | 7 | 14 | 28 | 70 | 0 | 2 | 7 | 27 |
| **General Procedures** | | | | | | | | | | | | | | | | | | | | |
| Study Briefing | X |  |  |  |  |  |  |  |  |  |  |  |  |  |  |  |  |  |  |  |
| Informed Consent | X |  |  |  |  |  |  |  |  |  |  |  |  |  |  |  |  |  |  |  |
| Assessment of Understanding | X |  |  |  |  |  |  |  |  |  |  |  |  |  |  |  |  |  |  |  |
| Provision of Study Information b |  | X |  |  |  |  |  |  |  |  |  |  |  |  |  |  |  |  |  |  |
| Medical History c | X | X | X | X | X | X | X | X | X | X | X | X | X | X | X | X | X | X | X | X |
| Physical Examination d | X | X | X | X | X | X | X | X | X | X | X | X | X | X | X | X | X | X | X | X |
| Vital Signs e | X | X | X | X | X | X | X | X | X | X | X | X | X | X | X | X | X | X | X | X |
| Height and Weight | X |  |  |  |  |  |  |  |  |  |  |  |  |  |  |  |  |  |  |  |
| Memory Aid Review |  |  |  | X | X |  |  | X | X |  |  | X | X |  |  |  |  | X | X |  |
| Concomitant Medications | X |  | X | X | X | X | X | X | X | X | X | X | X | X | X | X | X | X | X | X |
| Inclusion and Exclusion Criteria | X | X | X |  |  |  | X |  |  |  | X |  |  |  |  |  | X |  |  |  |
| Sickle Cell Screen, G6PD (EDTA) | X |  |  |  |  |  |  |  |  |  |  |  |  |  |  |  |  |  |  |  |
| HIV-1/2 antigen/antibodies, 4^th^ generation screen and confirmation (if needed), HBsAg and anti-HCV (SST) | X |  |  |  |  |  |  |  |  |  |  |  |  |  |  |  |  |  |  |  |
| HuAd5 antibody testing (SST) |  |  |  |  |  |  |  |  |  |  |  |  |  |  |  |  | X |  |  | X |
| EKG | X |  |  |  |  |  |  |  |  |  |  |  |  |  |  |  |  |  |  |  |
| Urine Pregnancy Test | X |  | X |  |  |  | X |  |  |  | X |  |  |  |  |  | X |  |  |  |
| HLA Typing (Buccal swab) |  | X |  |  |  |  |  |  |  |  |  |  |  |  |  |  |  |  |  |  |
| **Product Administration** | | | | | | | | | | | | | | | | | | | | |
| Immunization f g |  |  | X |  |  |  | X |  |  |  | X |  |  |  |  |  | X |  |  |  |
| **Safety Assessments** | | | | | | | | | | | | | | | | | | | | |
| Adverse Events |  |  | X | X | X | X | X | X | X | X | X | X | X | X | X | X | X | X | X | X |
| CBC with differential (EDTA) |  |  | X | X | X |  | X | X | X |  | X | X | X |  | X | X | X | X | X |  |
| Serum Chemistry (SST) |  |  | X | X | X |  | X | X | X |  | X | X | X |  | X | X | X | X | X |  |
| Urinalysis | X |  |  |  |  |  |  |  |  |  |  |  |  |  |  |  |  |  |  |  |
| **Immunogenicity Assessments** | | | | | | | | | | | | | | | | | | | | |
| CSP/AMA1 ELISpot Baseline | X |  |  |  |  |  |  |  |  |  |  |  |  |  |  |  |  |  |  |  |
| CSP/AMA1 ELISA Baseline | X |  |  |  |  |  |  |  |  |  |  |  |  |  |  |  |  |  |  |  |
| Cellular Immunity (FluoroSpot, ICS, Heparin tube) |  | X |  |  |  |  |  |  |  |  |  |  |  |  | X |  | X |  |  | X |
| Humoral Immunity (ELISA, IFA, SST) |  | X |  |  |  | X | X |  |  | X | X |  |  | X | X |  | X |  |  | X |
| Transcriptome |  |  | X | X | X | X | X | X | X | X | X | X | X | X |  |  | X | X | X |  |
| **Blood Volumes** | | | | | | | | | | | | | | | | | | | | |
| Daily (mL) | 33^h^ | 150 | 11.5 | 11.5 | 11.5 | 12.5 | 21.5 | 11.5 | 11.5 | 12.5 | 21.5 | 11.5 | 11.5 | 12.5 | 159 k | 9 | 162 ^i^ | 11.5 | 11.5 | 150 h |
| Cumulative (mL) | 33 | 183 | 195 | 206 | 218 | 230 | 252 | 263 | 275 | 287 | 309 | 320 | 332 | 344 | 503 | 512 | 674 | 685 | 697 | 847 |

a The day relative to the first immunization will be documented per the table, even if the full window period is used.

b Study information includes a study schedule and a contact card that contains who to contact and how to contact the research site.

c Complete at screening, then noting interim changes in the medical history at other visits.

d Detailed at screening, then focused or targeted exams at other visits. Screening includes cardiovascular disease risk evaluation determined by the method of [Gaziano et al (2008)](#Gaziano_et_al_2008).

e Vital signs include temperature, heart rate, and blood pressure.

f Subjects will remain at the CTC for observation for at least 30 minutes following each immunization. After the 30-minute observation period, temperature, pulse rate, and blood pressure will be measured, and any adverse events will be recorded. Subjects will also have a telephone follow-up within 24 hours of each immunization.

g Subjects will be given a memory aid, ruler, and digital thermometer.

^h^ Screening includes ALT, AST, alkaline phosphatase, BUN, creatinine, glucose, calcium, and total bilirubin. Other visits only include ALT, AST, alkaline phosphatase, total bilirubin, and creatinine.

^i^ Blood volumes of 162 mL include 140 mL for cellular assays, 10 mL for ELISA and HuAd5, 9 mL for safety labs, and 2.5 mL for Transcriptome.

^j^ Blood volume of 150 mL include 140 mL for cellular assays, 10 mL for ELISA and HuAd5.

^k^ Blood volumes of 159 mL include 140 mL for cellular assays and 10 mL for ELISA and 9 mL for safety.

Table 6: Study Event Schedule and Procedures from Day 196 Through Day 286 for Immunized Subjects

| Procedure | CHMI | Day 1 | Post Challenge Hotel Phase | | | | | | | | | | Post Challenge  Follow-up a | | | | Final Visit b | | | |
| --- | --- | --- | --- | --- | --- | --- | --- | --- | --- | --- | --- | --- | --- | --- | --- | --- | --- | --- | --- | --- |
| Visit Number | 21 | 22 | 23 | 24 | 25 | 26 | 27 | 28 | 29 | 30 | 31 | 32 | 33 | 34 | 35 | 36 | 37 | 38 | 39 | 40 |
| Day relative to 1st immunization c | 196 | 197 | 203 | 204 | 205 | 206 | 207 | 208 | 209 | 210 | 211 | 212 | 213 | 214 | 216 | 218 | 221 | 224 | 231 | 286 |
| Window | ±7 | ±1 |  |  |  |  |  |  |  |  |  |  |  |  | ±1 | ±1 | ±2 | ±3 | ±5 | ±14 |
| Day relative to CHMI | 0 |  | 7 | 8 | 9 | 10 | 11 | 12 | 13 | 14 | 15 | 16 | 17 | 18 | 20 | 22 | 25 | 28 | 35 | 90 |
| **General Procedures** |  |  |  |  |  |  |  |  |  |  |  |  |  |  |  |  |  |  |  |  |
| Medical History d | X |  | X | X | X | X | X | X | X | X | X | X | X | X | X | X | X | X | X | X |
| Physical Examination e | X |  | X | X | X | X | X | X | X | X | X | X | X | X | X | X | X | X | X | X |
| Vital Signs f | X |  | X | X | X | X | X | X | X | X | X | X | X | X | X | X | X | X | X | X |
| Concomitant Medications | X |  | X | X | X | X | X | X | X | X | X | X | X | X | X | X | X | X | X | X |
| Inclusion and Exclusion Criteria | X |  |  |  |  |  |  |  |  |  |  |  |  |  |  |  |  |  |  |  |
| Urine Pregnancy Test | X |  |  |  |  |  |  |  |  |  |  |  |  |  |  |  |  |  |  |  |
| Adverse Events | X | X | X | X | X | X | X | X | X | X | X | X | X | X | X | X | X | X | X | X |
| CBC with differential (EDTA) g | X |  |  |  |  |  |  |  |  |  |  |  |  |  |  |  |  | X |  | X |
| Serum Chemistry (SST) g | X |  |  |  |  |  |  |  |  |  |  |  |  |  |  |  |  | X |  | X |
| Cellular Immunity (FluoroSpot, ICS; Heparine tube) |  |  |  |  |  |  |  |  |  |  |  |  |  |  |  |  |  |  | X | X |
| Humoral Immunity (ELISA, IFA, SST) |  |  |  |  |  |  |  |  |  |  |  |  |  |  |  |  |  |  | X | X |
| Transcriptome | X | X | X |  |  |  |  |  |  | X h |  |  |  |  |  |  |  |  |  |  |
| **CHMI Specific Procedures** |  |  |  |  |  |  |  |  |  |  |  |  |  |  |  |  |  |  |  |  |
| Sporozoite Challenge i | X |  |  |  |  |  |  |  |  |  |  |  |  |  |  |  |  |  |  |  |
| Malaria Smear/PCR (EDTA) |  |  | X | X | X | X | X | X | X | X | X | X | X | X | X | X | X | X |  |  |
| **Blood Volumes** | | | | | | | | | | | | | | | | | | | | |
| Daily (mL) | 11.5 | 2.5 | 6.5 | 4 j | 13 k | 4 | 4 | 13 l | 4 | 6.5 | 4 | 4 | 4 | 4 | 4 | 4 | 4 | 13 | 150 m | 159 n |
| Cumulative (mL) | 858 | 861 | 867 | 871 | 884 | 888 | 892 | 905 | 909 | 916 | 920 | 924 | 928 | 932 | 936 | 940 | 944 | 957 | 1107 | 1266 |

a The Day 20, 22, 25, and 28 visits are for subjects who remain negative for parasitemia. All subjects treated and discharged from the hotel are to come to the CTC for the Day 28 Post CHMI visit.

b There will be a telephone follow-up at 6 months and 12 months after the challenge.

c The day relative to the first immunization will be documented per the table, even if the full window period is used.

d Complete at screening, then noting interim changes in the medical history at other visits.

e Detailed at screening, then focused or targeted exams at other visits.

f Vital signs include temperature, heart rate, and blood pressure.

g Also at any time point post challenge when deemed clinically indicated by the investigator.

h Screening includes ALT, AST, alkaline phosphatase, BUN, creatinine, glucose, calcium, and total bilirubin. Other visits only include ALT, AST, alkaline phosphatase, total bilirubin, and creatinine.

i To be obtained on completion of treatment before discharge from the hotel phase. Day 14 time point is approximate.

j Subjects will be closely observed for at least 30 minutes following the challenge. After the 30-minute observation period temperature, pulse rate, and blood pressure will be measured. Subjects will be counseled to use methods that will reduce risk of exposure to mosquitoes beginning 5 days after challenge until 20 days after the challenge.

k Blood volume of 4 mL includes 2 mL for malaria smear and 2 mL for RT-PCR for *P falciparum*, the latter will be evaluated on a retrospective basis.

l Blood volume of 13 mL includes 2 mL for malaria smear and 2 mL for RT-PCR for *P falciparum* as part of daily evaluation and 9 mL for safety laboratory tests obtained at the onset of parasitemia. Parasitemia can occur at any time between Day 7 and Day 18 post-challenge.

m Blood volume of 13 mL includes 2 mL for malaria smear and 2 mL for RT-PCR for *P falciparum* as part of daily evaluation and 9 mL for safety laboratory tests obtained at 72 hours after the onset of parasitemia.

n Blood volume of 150 mL include 140 mL for cellular assays, 10 mL for ELISA.

o Blood volume of 159 mL includes 140 mL for cellular assays, 10 mL for ELISA, and 9 mL for safety laboratory tests.

Table 7: Study Event Schedule and Procedure for Infectivity Control Subjects

| Procedure | Screening | Pre-CHMI | CHMI | Day 1 | Post Challenge Hotel Phase | | | | | | | | | | | | Post Challenge Follow-up a | | | | | Final Visit b |
| --- | --- | --- | --- | --- | --- | --- | --- | --- | --- | --- | --- | --- | --- | --- | --- | --- | --- | --- | --- | --- | --- | --- |
| Visit Number | 1 | 2 | 3 | 4 | 5 | 6 | 7 | 8 | 9 | 10 | 11 | 12 | 13 | 14 | 15 | 16 | 17 | 18 | 19 | 20 | 21 | 22 |
| Day relative to CHMI c | -90 to -8 | -14 to -1 | 0 | 1 | 7 | 8 | 9 | 10 | 11 | 12 | 13 | 14 | 15 | 16 | 17 | 18 | 20 | 22 | 25 | 28 | 35 | 90 |
| Window |  | ±7 |  | +1 |  |  |  |  |  |  |  |  |  |  |  |  | ±1 | ±1 | ±2 | ±3 | ±5 | ±14 |
| **General Procedures** | | | | | | | | | | | | | | | | | | | | | | |
| Study Briefing | X |  |  |  |  |  |  |  |  |  |  |  |  |  |  |  |  |  |  |  |  |  |
| Informed Consent | X |  |  |  |  |  |  |  |  |  |  |  |  |  |  |  |  |  |  |  |  |  |
| Assessment of Understanding | X |  |  |  |  |  |  |  |  |  |  |  |  |  |  |  |  |  |  |  |  |  |
| Provision of Study Information d |  | X |  |  |  |  |  |  |  |  |  |  |  |  |  |  |  |  |  |  |  |  |
| Medical History e | X | X | X |  | X | X | X | X | X | X | X | X | X | X | X | X | X | X | X | X | X | X |
| Physical Examination f | X | X | X |  | X | X | X | X | X | X | X | X | X | X | X | X | X | X | X | X | X | X |
| Vital Signs g | X | X | X |  | X | X | X | X | X | X | X | X | X | X | X | X | X | X | X | X | X | X |
| Height and Weight | X |  |  |  |  |  |  |  |  |  |  |  |  |  |  |  |  |  |  |  |  |  |
| Concomitant Medications | X |  | X |  | X | X | X | X | X | X | X | X | X | X | X | X | X | X | X | X | X | X |
| Inclusion and Exclusion Criteria | X | X | X |  |  |  |  |  |  |  |  |  |  |  |  |  |  |  |  |  |  |  |
| Sickle Cell Screen, G6PD (EDTA) | X |  |  |  |  |  |  |  |  |  |  |  |  |  |  |  |  |  |  |  |  |  |
| HIV-1/2 antigen/antibodies 4^th^ generation screen and confirmation (if needed), HBsAg and anti-HCV (SST) | X |  |  |  |  |  |  |  |  |  |  |  |  |  |  |  |  |  |  |  |  |  |
| EKG | X |  |  |  |  |  |  |  |  |  |  |  |  |  |  |  |  |  |  |  |  |  |
| Urine Pregnancy Test | X |  | X |  |  |  |  |  |  |  |  |  |  |  |  |  |  |  |  |  |  |  |
| **Safety Assessments** | | | | | | | | | | | | | | | | | | | | | | |
| Adverse Events |  |  | X | X | X | X | X | X | X | X | X | X | X | X | X | X | X | X | X | X | X | X |
| CBC with Differential (EDTA) h | X |  | X |  |  |  |  |  |  |  |  |  |  |  |  |  |  |  |  | X |  | X |
| Serum Chemistry (SST) h i | X |  | X |  |  |  |  |  |  |  |  |  |  |  |  |  |  |  |  | X |  | X |
| Urinalysis | X |  |  |  |  |  |  |  |  |  |  |  |  |  |  |  |  |  |  |  |  |  |
| **Immunogenicity Assessments** | | | | | | | | | | | | | | | | | | | | | | |
| CSP/AMA1 ELISpot Baseline | X |  |  |  |  |  |  |  |  |  |  |  |  |  |  |  |  |  |  |  |  |  |
| CSP/AMA1 ELISA Baseline | X |  |  |  |  |  |  |  |  |  |  |  |  |  |  |  |  |  |  |  |  |  |
| Cellular Immunity (FluoroSpot, ICS; heparine tube) |  | X |  |  |  |  |  |  |  |  |  |  |  |  |  |  |  |  |  |  | X | X |
| Humoral Immunity (ELISA, IFA, SST) |  | X |  |  |  |  |  |  |  |  |  |  |  |  |  |  |  |  |  |  | X | X |
| Transcriptome |  |  | X | X | X |  |  |  |  |  |  | X^p^ |  |  |  |  |  |  |  |  |  |  |
| **CHMI Specific Procedures** | | | | | | | | | | | | | | | | | | | | | | |
| Sporozoite Challenge j |  |  | X |  |  |  |  |  |  |  |  |  |  |  |  |  |  |  |  |  |  |  |
| Malaria Smear/PCR |  |  |  |  | X | X | X | X | X | X | X | X | X | X | X | X | X | X | X | X |  |  |
| **Blood Volumes** | | | | | | | | | | | | | | | | | | | | | | |
| Daily volume (mL) | 33 | 150 | 11.5 | 2.5 | 6.5 | 4 k | 13 l | 4 | 4 | 13 m | 4 | 6.5 | 4 | 4 | 4 | 4 | 4 | 4 | 4 | 13 | 150 n | 159 o |
| Cumulative volume (mL) | 33 | 183 | 195 | 197 | 204 | 208 | 221 | 225 | 229 | 242 | 246 | 252 | 256 | 260 | 264 | 268 | 272 | 276 | 280 | 293 | 452 | 611 |
| **Physiological Status Monitoring** |  |  |  |  |  |  |  |  |  |  |  |  |  |  |  |  |  |  |  |  |  |  |
| Distribution of Devices |  | X |  |  |  |  |  |  |  |  |  |  |  |  |  |  |  |  |  |  |  |  |
| Training on use of devices |  | X |  |  |  |  |  |  |  |  |  |  |  |  |  |  |  |  |  |  |  |  |
| Collect/Download Data |  | X | X |  | X |  |  |  |  |  |  | X^p^ |  |  |  |  | X |  |  | X |  |  |
| Collect Devices |  |  |  |  |  |  |  |  |  |  |  |  |  |  |  |  |  |  |  | X |  |  |

a The Day 20, 22, 25, and 28 visits are for subjects who remain negative for parasitemia. All subjects treated and discharged from the hotel are to come to the CTC for the Day 28 Post CHMI visit.

b There will be a telephone follow-up at 6 months and 12 months after the challenge.

c The day relative to the first immunization will be documented per the table, even if the full window period is used.

d Study information includes a study schedule and a contact card that contains who to contact and how to contact the research site.

e Complete at screening, then noting any interim changes in the medical history at other visits.

f Detailed at screening, then focused or targeted exams at other visits. Screening includes cardiovascular disease risk evaluation determined by the method of [Gaziano et al-2008](#Gaziano_et_al_2008), Appendix D.

g Vital signs include temperature, heart rate, and blood pressure.

h Also at any time point post challenge when deemed clinically indicated by the investigator.

i Screening includes ALT, AST, alkaline phosphatase, BUN, creatinine, glucose, calcium and total bilirubin. Other visits only include ALT, AST, alkaline phosphatase, total bilirubin, and creatinine.

j Subjects will be closely observed for at least 30 minutes following the challenge. After the 30-minute observation period temperature, pulse rate, and blood pressure will be measured. Subjects will be counseled to use methods that will reduce risk of exposure to mosquitoes beginning 5 days after challenge until 28 days post-challenge.

k Blood volumes of 4 mL include 2 mL for malaria smear and 2 mL for RT-PCR for *P falciparum*, the latter will be analyzed on a retrospective basis.

l Blood volume of 13 mL includes 2 mL for malaria smear and 2 mL for RT-PCR for *P falciparum* as part of daily evaluation and safety laboratory tests at the onset of parasitemia.

m Blood volume of 13 mL includes 2 mL for malaria smear and 2 mL for RT-PCR for *P falciparum* as part of daily evaluation and 9 mL for safety laboratory tests at 72 hours after the onset of parasitemia.

n Blood volume of 150 mL includes 140 mL for cellular assays and 10 mL for ELISA.

o Blood volumes of 159 mL include 140 mL for cellular assays, 10 mL for ELISA, and 9 mL for safety laboratory tests.

^p^ To be obtained on completion of treatment before discharge from the hotel phase. Day 14 time point is approximate.

- 1. Measures Taken to Minimize/Avoid Bias

Recruitment will target enrollment of 44 healthy adults (20 per group plus 4 alternates) for the vaccinated cohorts. In addition 12 infectivity controls (plus 3 alternates) will be enrolled for the CHMI. Immunized subjects will be randomly assigned to 1 of 2 vaccine groups: D/ChAd63-CA vaccine (Group 1) and D/ChAd63-CAT vaccine (Group 2). Block randomization will be used to allocate subjects to the 2 vaccine groups, while subjects in the infectivity control group will be enrolled sequentially. Immunized subjects will be blinded to their vaccine group assignments. The clinical team will be aware of each subject’s vaccine group assignment, but the laboratory staff who will process the clinical samples and conduct the immunologic assays will be blinded to each subject’s group assignment. Moreover, the analysis of safety laboratory tests will be conducted by an independent laboratory whose staff will be blinded to the subjects’ vaccination status.

- 1. Investigational Products

Table 8 presents a summary description of the investigational products. The investigational vaccines are being evaluated for prevention of Pf malaria.

Table 8: Investigational Vaccines

| **D/ChAd63-CA** | |
| --- | --- |
| **Priming Products** | NMRC-MV-D-PfC (VCL-2571-Ald) referred to as D-C  NMRC-MV-D-PfA (VCL-2577-Ald) referred to as D-A |
| Dosage Form | Individually filled in single use vials:  D-C: 0.5mL/vial at 3 mg/mL  D-A: 0.5mL/vial at 3 mg/mL |
| Diluent | Phosphate Buffered Saline (0.01 M Sodium Phosphate pH 7.2, 0.9% NaCl).  The PBS diluent is used to dilute/mix the DNA products “at bedside” prior to vaccine administration. |
| Formulate | Phosphate Buffered Saline (0.01 M Sodium Phosphate pH 7.2, 0.9% NaCl).  PBS is used to formulate each DNA product before vialing |
| Unit Dose | 1 mg per construct, 2 mg total dose (2 antigens) |
| Route of Administration | Intramuscular injection via Biojector 2000^®^ device (or an equivalent needle-free jet injection device) |
| Physical Description | Clear, colorless solution |
| Manufacturer | Aldevron |
| Lot Number | To be provided by manufacturer |
| **Boosting Products** | ChAd63 CSP referred to as ChAd63-C  ChAd63 AMA1 referred to as ChAd63-A |
| Dosage Form | Individually filled in single use vials:  ChAd63-C: 0.65mL/vial at 1.41 x 10^11^vp/mL  ChAd63-A: 0.65mL/vial at 1.67 x 10^11^vp/mL |
| Diluent | None |
| Formulate | Formulation Buffer, FB (10 mM histidine, 1 mM magnesium chloride, 0.1% polysorbate 80, 0.5% ethanol, 7.5% sucrose, 35 mM sodium chloride, and 0.1 mM edetate disodium, pH 6.6)  The ChAd63 products are formulated and filled in Formulation Buffer. |
| Unit Dose | 5 x 10^10^vp per construct, 1.0 x 10^11^vp total dose (2 antigens) |
| Route of Administration | Intramuscular injection via conventional needle and syringe |
| Physical Description | Slightly opaque, particle free |
| Manufacturer | Clinical BioManufacturing Facility (CBF), Oxford, UK |
| Lot Number | ChAd63-C lot 03011-01  ChAd63-A lot 01 Fill 09-04 |
| **D/ChAd63-CAT** | |
| **Priming Products** | NMRC-MV-D-PfC (VCL-2571-Ald) referred to as D-C  NMRC-MV-D-PfA (VCL-2577-Ald) referred to as D-A  NMRC-MV-D-PfT (VCL-2576-Ald) referred to as D-T |
| Dosage Form | Individually filled in single use vials:  D-C: 0.5 mL/vial at 3 mg/mL  D-A: 0.5 mL/vial at 3 mg/mL  D-T: 0.5 mL/vial at 3 mg/mL |
| Study Diluent | Phosphate Buffered Saline (0.01 M Sodium Phosphate pH 7.2, 0.9% NaCl).  The diluent is used to dilute/mix the DNA products “at bedside” prior to vaccine administration. |
| Formulate | Phosphate Buffered Saline (0.01 M Sodium Phosphate pH 7.2, 0.9% NaCl).  PBS is used to formulate each DNA component before vialing. |
| Unit Dose | 1 mg per construct, 3 mg total dose (3 antigens) |
| Route of Administration | Intramuscular injection via Biojector 2000^®^ device (or an equivalent needle-free jet injection device) |
| Physical Description | Clear, colorless solution |
| Manufacturer | Aldevron |
| Lot Number | To be provided by manufacturer |
| **Boosting Products** | ChAd63 CSP referred to as ChAd63-C  ChAd63 AMA1 referred to as ChAd63-A  ChAd63 ME-TRAP referred to as ChAd63-T |
| Dosage Form | Individually filled in single use vials:  ChAd63-C: 0.65 mL/vial at 1.41 x 10^11^vp/mL  ChAd63-A: 0.65 mL/vial at 1.67 x 10^11^vp/mL  ChAd63-T: 0.65 mL/vial at 1.44 x 10^11^vp/mL |
| Diluent | None |
| Formulate | Formulation Buffer, FB (10 mM histidine, 1 mM magnesium chloride, 0.1% polysorbate 80, 0.5% ethanol, 7.5% sucrose, 35 mM sodium chloride, and 0.1 mM edetate disodium, pH 6.6)  FB is used to formulate the vialed product but is not needed to mix vaccines “at bedside.” |
| Unit Dose | 5 x 10^10^vp per construct, 1.5 x 10^11^vp total dose (3 antigens) |
| Route of Administration | Intramuscular injection via conventional needle and syringe |
| Physical Description | Slightly opaque, particle free |
| Manufacturer | Clinical BioManufacturing Facility (CBF), Oxford, UK |
| Lot Number | ChAd63-C lot 03011-01  ChAd63-A lot 01 Fill 09-04  ChAd63-T lot 01S11-01 |

- - 1. Investigational Product Packaging and Labeling

The DNA priming products are manufactured by Aldevron (Fargo, North Dakota). These cGMP DNA priming products are filled separately at a volume of 0.5 mL (3 mg/mL) in 2 mL glass vials with stoppers. The DNA vials are intended for single use only, and partially used vials will not be administered to other subjects. The label information for these products is shown in Figure 4.

Figure 4: Label Information for the DNA Priming Products

| NMRC-MV-D-PfC (VCL-2571-Ald)  3 mg/mL in Phosphate Buffered Saline, 0.5 mL  Aldevron Lot #: xxxxx; Manuf. Date: [add date]  Store Frozen (-80°C +/- -20°C)  Aldevron GMP Service, Fargo, ND 58104 USA  Caution: New Drug – Limited by Federal  (or United States) Law to Investigational Use. |
| --- |
| NMRC-MV-D-PfA (VCL-2577-Ald)  3 mg/mL in Phosphate Buffered Saline, 0.5 mL  Aldevron Lot #: xxxxx ; Manuf. Date: [add date]  Store Frozen (-80°C +/- -20°C)  Aldevron GMP Service, Fargo, ND 58104 USA  Caution: New Drug – Limited by Federal  (or United States) Law to Investigational Use. |
| NMRC-MV-D-PfT (VCL-2576-Ald)  3 mg/mL in Phosphate Buffered Saline, 0.5 mL/vial  Aldevron Lot #: xxxxx; Manuf. Date: [add date]  Store Frozen (-80°C +/- -20°C)  Aldevron GMP Service, Fargo, ND 58104 USA  Caution: New Drug – Limited by Federal  (or United States) Law to Investigational Use. |

The 3 replication-deficient ChAd63 vectors encoding CSP, AMA1, or ME-TRAP are manufactured by Clinical BioManufacturing Facility or CBF (Oxford, UK). The cGMP [MHRA] products are filled separately at a volume of 0.65 mL into Type 1 glass 2 mL vials with a 13 mm grey bromobutyl rubber freeze dry stopper and a 13 mm complete tear, clear lacquered aluminum seal. The label information for these products is shown in Figure 5.

Figure 5: Label Information for the ChAd63 Boosting Products

| ChAd63 CSP Vaccine  Lot #: 03O11-01; Manuf. Date: 16 June 2011  Store Frozen (-70°C to -85°C)  Strength: 1.41 x 10^11^vp/mL; 0.65mL/vial  Caution: New Drug Limited by Federal (or United States) Law to Investigational Use  Manufacturer: Clinical BioManufacturing Facility (CBF), Oxford, U.K. |
| --- |
| **ChAd63 AMA1 Vaccine**  Lot #: 01, Fill 09-04; Manuf. Date: 17 June 2009  Store Frozen (-70°C to -85°C)  Strength: 1.67 x 10^11^vp/mL; 0.65mL/vial  Caution: New Drug Limited by Federal (or United States) Law to  Investigational Use  Manufacturer: Clinical BioManufacturing Facility (CBF), Oxford, U.K. |
| **ChAd63 ME-TRAP Vaccine**  Lot #: 01S11-1; Manuf. Date: 06 January 2011  Store Frozen (-70°C to -85°C)  Strength: 1.44 x 10^11^vp/mL ; 0.65mL/vial  Caution: New Drug Limited by Federal (or United States) Law to  Investigational Use  Manufacturer: Clinical BioManufacturing Facility (CBF), Oxford, U.K. |

- - 1. Investigational Product Storage

The products and diluents will be stored according to specifications provided by the manufacturer. The vaccine supplies will be stored frozen at -80°C ± -20°C for the DNA products (D-C, D-A, and D-T) and -70°C to -85°C for the adenovirus vectored products. The study diluents for the DNA will be stored at the same conditions as the DNA vaccine products. Individual products will be stored in a secured area at NMRC's contracted storage facility. Prior to each immunization day, a sufficient supply of the product will be delivered to the NMRC Clinical Trials Center (CTC) in Building 17B, Suite 2B, on the WRNMMC campus. The Clinical Research Coordinator (CRC) or designee will record receipt of the vaccine vials and study diluent. The vaccine products and diluent will be stored in a monitored, alarmed, ultra-low freezer (approximately -80°C) used exclusively for vaccine storage. During shipment of the products, a validated/monitored temperature data recorder will be used to monitor temperature consistency.

- - 1. Investigational Product Preparation and Administration

Each DNA and adenovirus vector product is vialed individually. The products will be blended prior to each administration as a 2 antigen (CSP + AMA1 for Group 1) or 3 antigen (CSP + AMA1 + TRAP for Group 2) DNA prime/ adenovirus boost vaccine.

The vaccines will be prepared (mixed) by a pharmacist or appropriately trained personnel in a biosafety cabinet per instructions in the relevant study specific procedure (SSP) on the day of immunization. The DNA vaccine must be administered within 8 hours of thawing. The ChAd63 vaccine must be administered within 1 hour of removal from the freezer.

- - - 1. DNA Priming Vaccines

The DNA products and diluents will be allowed to equilibrate to room temperature prior to mixing for injection and according to the relevant SSP. Any component not used within 8 hours of thawing will be discarded according to the sponsor's instructions and will be recorded as such for monitoring and accounting purposes. The products will be thawed, combined, and mixed with study diluent in a properly labeled mixing vial.

- - - - 1. D-CA

D-CA is a mixture of 2 DNA plasmids, each vialed separately, that are to be blended prior to administration. The vaccine will be prepared according to the relevant SSP. The 2 plasmid DNA products will be administered at a dose of 2 mg per administration (1 mg per construct) intramuscularly as two 1‑mL injections, one to the deltoid muscle of each arm by needle-free jet injection via Biojector 2000^®^ device or an equivalent needle-free jet injection device. The concentration of the mixed plasmid DNA is 1 mg/mL.

- - - - 1. D-CAT

D-CAT is a mixture of 3 DNA plasmid products, each vialed separately, that are to be blended prior to administration. The vaccine will be prepared according to the relevant SSP. Two injections of 1 mL per injection will be administered into the deltoid muscle, one injection in each arm (by needle-free jet injection via Biojector 2000^®^ device or an equivalent needle-free jet injection device), to deliver a total of 3 mg of D-CAT (1 mg of each D-C, D-A, and D-T products). The concentration of mixed plasmid DNA is 1.5 mg/mL.

- - - 1. Adenovector Boosting Vaccine (ChAd63)

The ChAd63 vaccines must be administered within 1 hour of removal of the products from the freezer. Any product or component not used within the 1 hour period will be discarded and recorded as such for monitoring and accounting purposes.

- - - - 1. ChAd63-CA

This vaccine is a mixture of 2 recombinant adenoviruses, each vialed separately, and will be blended prior to administration, according to the relevant SSP. The vaccine is to be administered by standard IM injection using a needle and syringe at a target dose of 1.0 x 10^11^ vp of ChAd63-CA (5 x 10^10^ vp of each ChAd63-C, ChAd63-A ).

- - - - 1. ChAd63-CAT

This vaccine is a mixture of 3 recombinant adenoviruses, each vialed separately, and will be blended prior to administration, according to the relevant SSP. The vaccine is to be administered by standard IM injection using a needle and syringe at a target dose of 1.5 x 10^11^ vp of ChAd63-CAT (5 x 10^10^ vp of each ChAd63-C, ChAd63-A, and ChAd63-T).

- - 1. Investigational Product Accountability

The sponsor's representative is responsible for distributing the investigational product to the study site. The sponsor's representative has delegated drug accountability responsibility for this product to the principal investigator (PI); however, the sponsor's representative has ultimate responsibility for product accountability. After the investigational product is distributed, the PI is responsible for and will maintain logs of investigational product receipt, storage, reconstitution, accountability by subject, and investigational product remaining before final disposition. At the NMRC Clinical Trials Center, the logs will be maintained in the accountability files within the locked records room accessible only to authorized personnel. The PI may delegate, in writing, this responsibility to another individual, but the PI is ultimately responsible for the investigational product and its proper storage upon receipt at the study site until it is transferred back to the sponsor’s representative or designee or is destroyed, as directed by the sponsor's representative.

All unused or partially used investigational product and empty vials will be destroyed as directed by the sponsor's representative and as stipulated by local, state, and Federal regulations.

- 1. Duration of Subject Participation

Each immunized subject will actively participate up to approximately 50 weeks (screening, immunization, challenge, and follow-up). Control subjects will participate up to 24 weeks including screening, challenge, treatment, and follow-up.

- 1. Dose-adjustment Criteria
     1. Stopping Rules

An individual subject will be withdrawn from further vaccination if he or she develops any of the following:

- A Grade 4 vaccine-related local adverse event based on the investigator’s discretion
- Any Grade 3 systemic adverse event or laboratory adverse event lasting > 48 hours beginning within 72 hours after vaccination that is determined to be possibly, probably, or definitely related to the vaccine
- Any Grade 4 laboratory adverse event confirmed on repeat testing or Grade 4 systemic adverse event that is determined to be possibly, probably, or definitely related to the vaccine.

If it is determined that no investigational vaccine or additional investigational vaccine will be administered to a subject in the future, he or she will remain in the study for safety follow-up.

If any of the following events occur, administration of investigational product will be discontinued for all subjects in a group until a thorough review of the events is undertaken by the investigators, local Institutional Review Board (IRB) and/or the United States Army Medical Research and Materiel Command Office of Research Protections (USAMRMC ORP), research monitor, and the sponsor’s safety office [USAMRMC Office of Regulated Activities, Product Safety Surveillance Branch (PSSB)].

- Following immunization, 3 or more subjects in the same group experience the same Grade 3 laboratory abnormality, or the same Grade 3 systemic AE that is determined to be possibly, probably, or definitely related to the vaccine.
- One vaccine-related unexpected AE occurs and after evaluation by the PI, research monitor, and sponsor's representative is determined to be an unacceptable risk to the health and safety of other investigational product recipients.
- One serious adverse event (SAE) determined to be definitely, probably, or possibly related to the investigational vaccine unless it is clearly unrelated to immunization as determined by the PI, research monitor, and the sponsor’s representative.

The study may be resumed with the concurrence of the IRB, research monitor, sponsor’s representative, principal investigator, and the US Food and Drug Administration (FDA).

- - 1. Study Termination Criteria

The PI, research monitor, sponsor's representative, the United States Army Medical Research and Materiel Command (USAMRMC) Office of Research Protections, Human Research Protection Office (ORP HRPO), or the FDA may stop or suspend the use of this product at any time.

- 1. Trial Treatment Randomization Codes

When a subject is enrolled in the study, he/she will be assigned a subject identification number that will be formatted according to SSP. The allocation of subjects among the 2 immunization groups will occur prior to the first immunization. A stratification and block randomization methods will be used for the allocation of subjects to the 2 immunization groups, and this method will be further described in an SSP. Clinical staff personnel will not be blinded to the immunization group assignments. However, the subjects and immunology laboratory staff will be blinded to the immunization groups. The randomization list and randomization codes will be generated by the study statistician through computerized randomization.

- 1. Identification of Data to be Recorded on the Case Report Forms

Data will be recorded onto a hand-written form at the time of the visit, which will serve as the source documentation. Data from the source documents will be entered in the electronic data capture (EDC) system. No source data will be recorded directly in the computerized database without prior written record. The transcribed data will be consistent with the source documents or the discrepancies will be explained.

The study team will prepare the source documentation. The data management team will prepare the eCRFs, and data entry personnel will enter data. Quality Assurance will review source documentation and verify electronic data.

For more information on data handling, refer to Section 16.

Selection and Withdrawal of Subjects

- 1. Recruitment of Subjects

Adult men and women, civilian and active duty military subjects will be recruited from the general population in the Baltimore-Washington DC area, by use of advertisements in multiple media formats to include, but not limited to, informational flyers; large media formats inclusive of newspaper, Metro, bus advertisements and social media (eg, Facebook, NMRC website, etc); e-mail, radio, and word of mouth. All recruitment materials will be prepared and submitted for review and approval by the NMRC IRB (and other appropriate reviews) prior to use. The large media formats require high-definition/high-quality images in order to maintain integrity and quality. The IRB approved recruitment materials used in large media formats may be used without the IRB approval stamp in order to maintain the high resolution quality necessary but IRB approval information (including date of expiration) will be placed on those specific advertisements in some way, such as a footer, etc. The information may appear in text format and will include all of the following information: NAVY MEDICINE HRPP; HRPP # NMRC.2016.0006; Approval Date; Expiration Date; and Verification by ORA Staff.

When a subject calls the CTC and discloses an interest in the study, the recruitment staff will discuss the trial from an IRB-approved script. If the subject is still interested, contact information will be obtained and an appointment for briefing and/or screening will be arranged. Recruitment will be coordinated by civilians when done within the military to avoid any perception of coercion by rank. Military members of the study team will be available to provide study information to all prospective individuals. Active duty military subjects will require approval from their supervisor which will be documented using the Statement of Supervisor's Approval.

Refer to Section 4.9 for a description of the subject population.

- 1. Referral Fee

A referral fee of $25 will be given to any prospective or current subject or any subject who has previously screened or enrolled in a study, who successfully recruits another subject. A subject will be considered a “successful recruit” if they meet all eligibility criteria and complete the entire screening visit.

- 1. Informed Consent Process

Informed consent and Health Insurance Portability and Accountability Act (HIPAA) authorization will be obtained from each subject prior to any procedures being performed.

The informed consent process will occur at the NMRC CTC. Individuals who are interested in learning about the trial will meet with a member of the study team. They will be given a complete description of the study and view a PowerPoint presentation. The Informed Consent Document (ICD) will be reviewed by one of the study investigators and/or a designee, and subjects will be given time to review the ICD and the opportunity to ask questions. They may take the ICD home to review and/or discuss with family and friends.

When ready, the subject will sign the ICD (Appendix A) and HIV testing consent (Appendix B). A copy of the signed informed consent and HIV consent will be provided to each subject. For members of the military, we will ensure that no one supervising the work of the subject or anyone in his/her chain of command will be permitted to consent the subject.

After signing the ICD, subjects will complete an Assessment of Understanding (Appendix C). Subjects must achieve a score of at least 80% correct on the multiple-choice. If they do not score at least 80% on the initial quiz, the protocol information will be reviewed and they will have the opportunity to retest. If a subject does not pass the quiz with a score of at least 80% correct, after 2 attempts, they will be excluded from the study.

- 1. Eligibility Screening

Each subject must meet all inclusion and none of the exclusion criteria. Subjects who have signed the ICD and completed the assessment of understanding will provide a medical history and undergo a physical examination and routine laboratory screening tests (Section 8.1.1). The PI or designee will make the final decision of the eligibility. Only eligible subjects will be given the investigational product.

- 1. Subject Inclusion Criteria

Subjects must meet all of the following criteria to be included in the study:

- Adults (male or non-lactating, non-pregnant female) between 18 to 50 years of age at the time of enrollment
- Available and willing to participate for duration of study
- Able and willing to provide a written informed consent
- Able to complete an Assessment of Understanding (Appendix C) with a score of at least 80% correct
- In good general health with no clinically significant health problems as established by medical history, physical examination, and laboratory screening
- Men and women of childbearing potential must agree to consistently use effective means of birth control throughout the duration of the study
  - Sexually active females, unless surgically sterile or at least 1 year post-menopausal, must use an effective method of avoiding pregnancy (including oral or implanted contraceptives, intrauterine device, female condom, diaphragm with spermicide, cervical cap, abstinence, use of a condom by the sexual partner or sterile sexual partner) from 14 days prior to the first immunization and must agree to continue using such precautions during the study and for 6 months after the last study visit (which occurs at 90 days after CHMI).
  - If female subjects are unable to bear children due to menopause or have had a procedure performed (tubal ligation or hysterectomy), a medical note from a physician is required.
  - If post-menopausal, subjects must have experienced at least 1 year of amenorrhea and provide a medical note from her physician documenting this medical history.
  - Sexually active men must agree to use effective means of birth control such as barrier methods (use of a condom) from the day of the first immunization and for the duration of the study (through 3 months after CHMI). Vasectomy is considered an adequate means of birth control. Men who underwent sterilization or vasectomy must provide a medical note from his physician documenting such procedure.
- Agree not to travel to a malaria endemic area during the course of the study
- Agree to refrain from blood donation during the study and for 3 years following CHMI
- Must be willing to take anti-malarial treatment after CHMI, if indicated.
- Must agree to stay in a pre-determined hotel near the NMRC CTC during the designated post-CHMI follow-up period from approximately 7 days after malaria challenge until antimalarial treatment is completed, if indicated
  1. Subject Exclusion Criteria

Subjects meeting any of the following criteria will be excluded from the study:

- Weight < 110 pounds for vaccine recipients only
- Body mass index (BMI) > 35 kg/m^2^for vaccine recipients only
- Pregnant (positive urine pregnancy test) or nursing at screening or plans to become pregnant or nurse at any period from the time of enrollment through 6 months after the last study visit (which will occur at 90 days after CHMI).
- Receipt of any investigational malaria vaccine
- Any history of malaria infection
- Travel to a malaria endemic region within 6 months of enrollment or during the study (from enrollment through 3 months after CHMI)
- History of long-term residence (> 5 years) in an area known to have significant transmission of *P falciparum* (http://www.cdc.gov/malaria/map/)
- History of clinically significant contact dermatitis or sensitivity to products that contain Kathon, such as shampoos, conditioners, soaps, detergents, moisturizers, lotions, baby wipes, or cosmetics
- Positive CSP and AMA1 ELISpot assay at screening
- Positive CSP and AMA1 ELISA assay at screening
- Seropositive for the human immunodeficiency virus (HIV), hepatitis C virus (HCV), and/ or hepatitis B surface antigen (HBsAg)
- Positive sickle cell screening test, including evidence of sickle cell trait or sickle cell anemia (due to its effect on subject’s susceptibility to malaria)
- History of thalassemia or thalassemia trait (due to its effect on subject’s susceptibility to malaria)
- Participation in any clinical study involving another investigational vaccine, drug, or other products within 60 days prior to the first immunization or plan to participate in such a clinical study during or within 1 month following the active study phase of the study (from the day of the first immunization through 3 months after CHMI)
- Allergy to any component of the vaccine formulation or serious adverse reaction to other vaccines (such as hives, anaphylaxis, respiratory difficulty, angioedema, or abdominal pain)
- History of a severe and/or anaphylactic response to mosquito-bites
- Known allergy to chloroquine phosphate, atovaquone/proguanil, or artemether/lumefantrine, which will be used to treat subjects who may develop malaria after *Plasmodium falciparum* challenge
- History of psoriasis (given its interaction with chloroquine)
- History of porphyria
- History of hemolytic anemia
- Use or planned use of any drugs with significant anti-malarial activity, such as doxycycline, clindamycin, azithromycin, or trimethoprim/sulfamethoxazole among others that would coincide with periods of CHMI or post-CHMI follow-up
- Has evidence of increased cardiovascular disease risk (> 5%-10%, 5-year risk)
  - As determined by the method of [Gaziano et al-2008](#Gaziano_et_al_2008), Appendix D
  - Risk factors include sex, age (years), smoking status, body mass index (BMI, kg/m2), presence or absence of diabetes mellitus, and blood pressure.
- An abnormal EKG, defined as one showing Q waves and/or significant ST-T wave changes, left ventricular hypertrophy, any non-sinus rhythm (excluding isolated premature atrial contraction), right or left bundle branch block, or advanced (secondary or tertiary) A-V heart block
- Current or chronic use of systemic immunosuppressant pharmacotherapy or immunomodulators; however, subjects may be allowed to use inhaled steroids or topical steroids.
- History of splenectomy (given its effects on immunity to malaria)
- Receipt of immunoglobulins and/or any blood products within 90 days of scheduled immunization
- History of neurologic disorder (including seizures or migraine headache)
- History of cancer (except for basal cell carcinoma of the skin)
- Current significant medical condition (cardiovascular, pulmonary, hepatic, renal, or hematological) or evidence of any other serious underlying medical condition identified by medical history, physical examination, or laboratory screening tests
- History of any other illness or condition which, in the investigator's judgment, may substantially increase the risk associated with the subject's participation in the protocol or compromise the scientific objectives. This may include psychiatric disorders (such as personality disorders, anxiety disorders, major depressive disorder or schizophrenia) or behavioral tendencies (including active alcohol or drug abuse) discovered during the screening process that in the opinion of the investigator would make compliance with the protocol difficult
- Plan for surgery during the study (from enrollment until 3 months post-CHMI) with the exception of minor cutaneous procedures
- Females who are pregnant or nursing, or plan to become pregnant or nurse during the study period (from enrollment through 3 months post CHMI) or within 6 months after the last study visit
- Any other significant finding that in the opinion of the investigator would increase the risk of having an adverse outcome from participating in this study or compromise the scientific objectives
  1. Subject Withdrawal Criteria

Subjects may withdraw consent at any time during the study without penalty. Counseling about the subject's health will be provided if he/she decides to discontinue participation in the study. Medical advice regarding what is in the best interest of the subject will be provided.

The PI may discontinue the subject's participation without the subject's consent if any of these criteria are met:

- A subject fails to comply with study procedures
- A subject's safety or health may be compromised by further participation
- The scientific integrity of the study may be compromised by further participation
  - 1. When and How to Withdraw Subjects

If a subject withdraws, the investigator will make a reasonable effort to determine the reason for the withdrawal from the study and to complete termination procedures including safety follow-ups or visits needed to assure the study subject's health. Telephone calls, text messages, registered letters, and email correspondence are considered appropriate means of communication. For subjects leaving the study, a targeted examination may be performed, if medically indicated and if permitted by the subject. If a subject withdraws after CHMI but prior to completing the 28 day follow up he or she will be clinically evaluated by the principal investigator or his or her designee, and the subject may be presumptively treated with a curative course of anti-malarial treatments. The decision to treat subjects who withdraw will be reviewed with Research Monitor prior to treatment.

A subject may be withdrawn for an adverse event (AE) or serious adverse event (SAE) resulting in a safety concern, for noncompliance with protocol requirements or because in the PI's judgment continued participation would place the subject at increased risk or compromise scientific objectives. When a subject withdraws due to an AE or is withdrawn by the principal investigator due to an AE, the sponsor's safety office, the USAMRMC Office of Regulated Activities, Product Safety Surveillance Branch, must be notified within 24 hours by fax or email (usarmy.detrick.medcom-usamrmc.mbx.sae-reporting@mail.mil). Investigators must follow specific policy regarding the timely reporting of AEs and SAEs to the local IRB (Section 11.4.1.2). In all cases, the PI will make a reasonable effort to complete study termination procedures.

If a subject meets withdrawal conditions for a concomitant medication violation or noncompliance, this should clearly be stated in the study termination source documents and eCRF.

- - 1. Data Collected for Withdrawn Subjects

All data collected up to the time of withdrawal will be reported. The study termination source documents will be completed, with the reason for withdrawal specified.

- - 1. Replacement of Subjects

At least 4 alternates will be recruited for the immunized groups in each cohort and will undergo the pre-immunization blood collection. These alternates will be asked to be present on the day of first immunization. If an assigned subject does not present on the day of the first immunization, elects to withdraw, or is found to have met an exclusion criteria, an alternate may be enrolled, immunized and proceed with the study if he/she can be immunized within the allowable study window for first immunization. Once immunization has begun (the study window for first immunization has passed), study subjects will not be replaced.

The 3 alternates recruited for the challenge controls will be asked to be present on the day of the challenge. If an assigned subject does not present on the day of the challenge, elects to withdraw, or is found to have met an exclusion criteria, an alternate will be enrolled.

- - 1. Follow-up for Withdrawn Subjects

If a subject withdraws, the investigator will make a reasonable effort to determine the reason for the subject's withdrawal from the study and to complete termination procedures. Withdrawing from the study for any reason will not impact the subject's medical care. Subjects who withdraw or are withdrawn will be followed, as allowed by the subject, through resolution of any on-going adverse events. Women who become pregnant during the active phase of the trial (starting at enrollment up to 3 months after CHMI) will be encouraged to seek obstetric care and will be asked to provide follow-up information at the conclusion of the pregnancy. Subjects who become pregnant during the active phase of the clinical trial will be followed to term by the principal investigator (see Section 4.6.1.6).

1. Treatment of Subjects
   1. Study Visit Schedule and Follow-up Periods

Immunized subjects will actively participate for approximately 50 weeks (screening, immunization, challenge, and follow-up). Subjects who are infectivity controls will participate for approximately 24 weeks. Please refer to Section 8 for a detailed discussion on recruitment of subjects, informed consent process, and eligibility screening. Evaluation of study vaccine safety will include medical history, review of the memory aid for solicited AEs, and physical assessment by clinicians and laboratory studies following immunization. The complete schedule of study visits, permitted windows for completing the visits, and evaluations performed at each visit are provided in Table 5, Table 6, and Table 7. Although there is a defined "window" for study visits (allowable days before and after), subjects will be encouraged to be present on the target study visit day. The blood volume drawn from each subject will not exceed 5 mL/kg (250 mL for a 50 kg subject) during a 24 hour period and 525 mL per 8-week period (per guidelines of the American Association of Blood Banks). The NMRC CTC will provide light snacks and refreshments to subjects for nutritional support and safety during study visits.

- - 1. Screening Visit

Screening procedures will be conducted at the NMRC CTC and may occur over multiple visits. The initial screening visit(s) for subjects in the immunized groups will be scheduled between 90 and 8 days prior to the receipt of the first DNA prime immunization session. For the infectivity control group, the screening visit will include the same procedures as in the immunized groups and will be scheduled 90 to 8 days prior to CHMI.

The following activities and procedures will take place during the screening visits:

- A detailed informational brief about the study will be provided.
- Subjects will have the opportunity to ask research staff question(s) to assist them in making informed decision about participating in the study.
- Informed consent process will be performed (Section 7.3), and the informed consent document (ICD) and other research-related documents will be signed.
- Subjects will take a quiz to assess understanding of the study.
- Temperature, pulse rate, blood pressure, height, and weight will be measured and recorded.
- Subjects will be interviewed to obtain a complete medical history. This includes analysis of 5-year cardiovascular risk based upon the method of [Gaziano et al-2008](#Gaziano_et_al_2008).
- Physical examination will be performed.
- A baseline 12-lead EKG will be performed and results will be reviewed by a board-certified cardiologist.
- Blood samples will be collected for safety laboratory screening and baseline immunogenicity assessments:
  - - - **Hematology**
  - Complete blood count (CBC) with differential
  - Hemoglobin
  - Platelet count
  - **Serum chemistry**
    - - Glucose
      - Creatinine
      - Blood urea nitrogen (BUN)
      - Calcium
      - Alanine aminotransferase (ALT)
      - Aspartate aminotransferase (AST)
      - Alkaline phosphatase
      - Total bilirubin
  - **Screening Serology**
  - HBsAg
  - Anti-HCV
  - Anti-HIV -1/2 antigen/antibodies 4^th^ generation screen and confirmation (consent will be obtained prior to evaluation of HIV serology)
  - **Sickle cell screen**
  - **Glucose-6-phosphate dehydrogenase (G6PD) screen**
  - **CSP and AMA1 ELISpot**
  - **CSP and AMA1 ELISA**
- Urine will be collected for urinalysis, including microscopic analysis, if indicated
- A urine pregnancy test will be performed for all female subjects regardless of age or reported sterilization, unless there is written proof of sterilization or that the subject is menopausal (appropriate age with 1 year of amenorrhea)

All screening laboratories will be considered expired if performed more than 90 days prior to immunization. In the event that the screening laboratories are expired, CBC with differential, glucose, creatinine, ALT, AST, Anti-HIV-1, HBsAg, anti-HCV, and urine pregnancy test will be repeated during the screening follow-up visit and results will be reviewed prior to the first immunization for the immunized groups and prior to the challenge for the infectivity controls.

Subjects who meet all inclusion criteria and none of the exclusion criteria, sign the informed consent document, and achieve a score of at least 80% correct answers on the Assessment of Understanding will be enrolled in the study. In subjects who meet all of the inclusion criteria but have an abnormality in 1 or more screening safety laboratory tests, the PI may elect to repeat such screening laboratory test(s) to exclude spurious results due to laboratory processing error, etc. The PI or designee will identify values as abnormal and clinically significant or not clinically significant. Subjects with abnormal labs that are clinically significant, for example corresponding to Grade 1 or higher, will only be enrolled after consultation with the research monitor(s) and/or appropriate expert. Subjects excluded from this study because of significant abnormalities will be managed initially by study clinicians and will be promptly referred to the subjects’ primary care providers for evaluation as necessary. In the event of a positive HIV test, the subject will be referred for appropriate counseling and follow-up care. Notification of state and federal authorities, as required by law, will be the responsibility of the PI. For members of the military, notification of command will also be the responsibility of the PI as required by military regulations.

- - 1. Pre-Immunization Visit / Pre-CHMI Visit

This visit will occur 14 to 1 days prior to the first immunization and prior to CHMI. The following events will occur:

- Study information will be provided: study schedule, contact card (who to contact and how to contact the research site)
- Medical history will be updated, a directed physical exam will be performed, and vital signs will be collected.
- Blood samples will be collected for:
  - Cellular mediated immunity assays (FluoroSpot and ICS)
  - Humoral immunity assays (ELISA and IFA)
  - Human adenovirus serotype 5 antibody testing (for ChAd63 immunization)
- A buccal smear will be performed for HLA typing (subjects in the immunization groups only)
- For infectivity control subjects, PSM wearable devices will be distributed and training will be conducted on their appropriate use

The buccal smear for HLA typing is necessary for the conduct of cellular assays assessing antigen-specific, HLA-class restricted cellular responses. If any subjects are found to have HLA types associated with increased risk of an autoimmune disease, they will be referred to their primary care physician for further counseling but would not be excluded from the study.

- - 1. Immunization Visits

Immunization visits will occur on Day 0, Day 28, and Day 56 for DNA prime and Day 168 for the ChAd63 boost. Immunizations will be conducted at the NMRC CTC in Bethesda, MD.

Also prior to each immunization, the following evaluations and procedures will occur:

- Inclusion and exclusion criteria will be reviewed.
- Medical history will be updated, a directed physical examination will be performed, and vital signs will be collected.
- Solicited and unsolicited AEs and concomitant medications will be documented.

Blood samples will be collected for safety tests (CBC with differential and serum chemistry) and immunogenicity assays at the time points shown in Table 5 (as a pre-immunization baseline).

- Method of birth control/contraception used will be reviewed with each subject.
- Women of childbearing potential will take a urine pregnancy test.

Immunization may not proceed unless a negative pregnancy test has been obtained for women.

Following immunizations, subjects will remain at the NMRC CTC for an observation period of at least 30 minutes. This will allow the study team to confirm that there are no immediate reactions in response to immunization and to provide immediate care if any reactions do occur. After the 30-minute observation period, an assessment will be performed including the measurement of temperature, pulse rate, and blood pressure and recording of adverse events. Subjects will be instructed to contact the clinical investigator if they experience any medical problems.

Subjects will be provided with a memory aid, a ruler, and a digital thermometer on the day of immunization. Subjects will be instructed on the use of the memory aid and encouraged to fill it out as directed. Each subject will be instructed to measure the size/area of redness, swelling, or other findings at the injection site daily by using a ruler. Using the thermometer, subjects will be instructed to measure and record their body temperature twice a day (once in the morning and once in the evening) and record symptom(s) on the memory aid for 7 days after each immunization. The memory aid will serve as a tool to remind each subject about the symptom(s) that he or she may wish to further discuss with the clinical investigators during designated follow-up visits post-immunization. The memory aid will be reviewed with the subject by an investigator at follow-up visits during the 7-day post-immunization period. Solicited AEs on the memory aid and/or clarifications of other symptoms will be documented in a progress note in the subject study chart. If memory aids are not completed or returned by Day 28 following each vaccination and there are missing data such as site reaction measurements, temperatures, or other adverse event information, then a protocol deviation will be documented.

- - 1. Follow-Up Visits Post-Immunization

Post immunization follow-up visits will occur at 2, 7, and 14 days after each DNA immunization and at 28 and 70 days after the third DNA immunization. Post immunization follow-up visits will occur at 2, 7, and 27 days after the ChAd63 boost immunization.

The following evaluations and procedures will occur during Days 2 and 7 after each immunization visits:

- Medical history will be updated, a directed physical examination will be performed, and vital signs will be collected.
- Solicited AEs that occur through Day 7 after each immunization will be documented.
- Unsolicited AEs that occur after each immunization will be documented.
- Laboratory abnormalities that occur after each immunization will be documented.
- Concomitant medications will be documented.
- Subject memory aid entries will be reviewed at Days 2 and 7 after each immunization and solicited AEs will be documented on the progress notes.
- Blood samples will be collected for safety tests (CBC with differential and/or serum chemistry) and immunogenicity assays at the time points shown in Table 5.
- Method of birth control/contraception used will be reviewed with each subject.

The following evaluations and procedures will occur on Day 14 post-DNA immunization visits:

- Medical history will be updated, a directed physical examination will be performed, and vital signs will be collected.
- Concomitant medications will be documented.
- Method of birth control/contraception used will be reviewed with each subject.
- Unsolicited AEs will be documented.
- Blood samples will be collected for immunogenicity assays at the time points shown in Table 5.

The following evaluations and procedures will occur on Day 28 post-third DNA immunization and Day 27 post-ChAd63 immunization:

- Medical history will be updated, a directed physical examination will be performed, and vital signs will be collected.
- Concomitant medications will be documented.
- Method of birth control/contraception used will be reviewed with each subject.
- Unsolicited AEs will be documented.
- Blood samples will be collected for safety tests (CBC with differential and/or serum chemistry), immunogenicity assays at the time points shown in Table 5.

The following evaluations and procedures will occur on Day 70 post-third DNA immunization:

- Medical history will be updated, a directed physical examination will be performed, and vital signs will be collected.
- Concomitant medications will be documented.
- Method of birth control/contraception used will be reviewed with each subject.
- Blood samples will be collected for safety tests (CBC with differential and/or serum chemistry), and possible immunogenicity assays at the time points shown in Table 5.

Each subject will be contacted by the study team within 24 hours after each immunization for a telephone follow-up, unless the subject already has a coinciding clinic visit on that day.

If the memory aid is not returned at each visit, it will not be considered a protocol deviation. The memory aid should be returned to the NMRC CTC by Day 7 post-immunization for examination by the clinical investigator. The memory aid shall serve as a reminder tool for each subject and will become a part of the source document; incomplete data on the memory aid would not be considered a deviation. If memory aids are not completed or returned by Day 28 following each vaccination and there are missing data such as site reaction measurements, temperatures, or other adverse event information, then a protocol deviation will be documented. The complete characterization of each symptom experienced by the subject post-immunization will be conducted by the clinical investigator who evaluates the subject during the follow-up visits.

Additionally, unsolicited AEs and safety laboratory abnormalities that occur through Day 28 after each immunization will be documented and concomitant medications during the active phase of the study (starting at enrollment through 3 months after CHMI) will be documented.

- - 1. Challenge (CHMI)

Challenge with viable, infectious sporozoites (Pf strain 3D7) will be conducted at WRAIR Insectary, Silver Spring, Maryland. The interval between last immunization and challenge for a given subject is 28 days (± 7 days). For infectivity controls, Day 0 is defined as day of challenge. The immunized and control subjects will be treated in an identical fashion.

In order to challenge subjects, mosquitoes infected via membrane feeds 14 to 21 days prior to challenge and containing sporozoites in their salivary glands will be allowed to feed on the subjects. For each subject, 5 mosquitoes will be allowed to feed. The mosquitoes will be dissected to confirm the presence of a blood meal and to determine the infectivity rate and the salivary gland score. The salivary gland score is a measure of the number of sporozoites observed under a microscope in media after crushing the mosquito salivary glands. The score ranges from 0 to 4. Additional mosquitoes will be allowed to feed until the subject receives a total of 5 infected mosquito bites from mosquitoes with a minimum 2+ salivary gland score (2+ = 11 - 100 sporozoites observed) ([Rickman et al-1990](#Rickman_et_al_1990)).

Subjects will be asked not to wear cologne, perfume, or aftershave on the day of challenge or use scented soaps in the 24 hours prior to challenge as it may discourage mosquito feeding. Subjects will be transported by a designated NMRC staff from the NMRC CTC to the WRAIR Insectary for the challenge.

The multiple feeds need to be accomplished within 1 challenge visit. The WRAIR insectary is prepared to conduct the challenge over several hours, if needed. During a challenge visit, a feeding session (in which a subject receives bites from 5 infected mosquitoes) using *A stephensi* carrying the 3D7 Pf strain may take up to 20 to 45 minutes. There have been rare instances when adequate mosquito feeding took greater than 4 hours, which were possibly related to the subject’s non-compliance with specific pre-challenge instructions or the altered biting habits of *A stephensi* carrying a different Pf strain.

Prior to the challenge, the following evaluations and procedures will be performed:

- Inclusion and exclusion criteria will be reviewed
- Medical history will be updated, a directed physical examination will be performed, and vital signs will be recorded
- Solicited and unsolicited AEs (for immunized subjects only) and concomitant medications will be documented
- Method of birth control/contraception used will be reviewed with each subject
- Blood samples will be collected for safety tests and transcriptome assays (Table 6 and Table 7)
- For infectivity control subjects, PSM data will be downloaded from the subject’s smartphone to a laboratory laptop on Day -7 and Day 0 (Table 7)
- Women of childbearing potential will take a urine pregnancy test

The challenge may not proceed unless a negative pregnancy test has been obtained.

- - 1. Management of Subjects Post-Challenge

Following the challenge, each subject will be closely observed for at least 30 minutes to assure that there are no systemic allergic reactions or other untoward events. Temperature, pulse rate, and blood pressure will also be taken. Subjects with signs or symptoms related to the challenge will be managed appropriately by a clinical investigator. Symptomatic treatment, such as topical anti-inflammatory medication, will be provided as necessary.

Before completing the challenge day, subjects will be informed of common malaria symptoms: fever (oral temperature of > 38.0ºC/100.4°F), chills, rigors (shaking chills), sweats, headache, dizziness, malaise, fatigue, insomnia, joint pain, muscle pain, neck ache, nausea, vomiting, stomach/abdominal cramps, diarrhea, and increased heart rate. Careful and systematic monitoring will be provided because subjects are expected to develop blood stage malaria. The period from the time of exposure (challenge day) to the time of detection of malaria symptoms is generally 9 to 14 days, and symptoms can present at the earliest on Day 7 ([Church et al-1997](#Church_et_al_1997), [Epstein et al-2007](#Epstein_et_al_2007), [Roestenberg et al-2012](#Roestenberg_et_al_2012)). Therefore, for the first 6 days subjects will be allowed to return to their usual routine.

Subjects will be given a "Notification Card" and instructed to keep this card with them at all times until they have either been fully treated for malaria or, in the event that they do not develop malaria, until the completion of the Day 28 post-challenge follow-up period. In the event that a subject requires urgent medical care (for example, following a motor vehicle accident), this card will provide an alert to medical staff, that the subject has been challenged with mosquitoes infected with Pf. It will also include the date of challenge and contact numbers for study staff. Subjects will also be instructed to contact the study team immediately in the event of any signs and symptoms of illness.

In addition, subjects will be counseled to use methods that will reduce risk of exposure to mosquitoes beginning 5 days after challenge until 28 days post-challenge (if remaining without parasitemia) or until 2 consecutive daily negative smears after treatment for parasitemia. This counseling will be provided in order to minimize the risk that research subjects might transmit malaria to local mosquito populations. However, individuals developing Pf malaria do not generate gametocytes until several days after the infection becomes patent, making transmission extremely unlikely even in the absence of precautions against mosquito bites.

Subjects will return to the CTC 1 day after CHMI for review of adverse events and a blood collection for transcriptome.

- - 1. Days 7 through 18 Post Challenge (Overnight Hotel Stays)

Pre-patent periods are generally within 9 to 14 days post challenge although they can be as early as 6 days and as late as 23 days ([Church et al-1997](#Church_et_al_1997); [Epstein et al-2007](#Epstein_et_al_2007), [Roestenberg et al-2012](#Roestenberg_et_al_2012)). To facilitate close daily monitoring of clinical status and blood smears from Days 7 through 18 post-challenge, subjects who are challenged will be required to stay overnight in a pre-determined hotel for 11 days, or until the first detection of parasitemia (and documentation of 2 consecutive negative daily smears after treatment with an anti-malarial drug). The subjects’ overnight stay at a designated hotel will facilitate 24/7 access to the study team during the hotel phase. During this time, subjects will be free to go about their normal daily activities, but must return to the hotel in the evenings. If a subject works at night or has an alternate schedule, he/she would be expected to return to the hotel after work to sleep. Each morning, subjects will be evaluated by the study team. A study physician will be available for consultation 24 hours per day during the hotel phase. Overnight hotel stay and parking expenses will not be paid by study subjects. The costs of the overnight hotel stay and parking, as needed per subject, will be paid by study funding.

The following evaluations and procedures will be performed daily during the hotel phase:

- Medical history will be updated, a directed physical examination will be performed, and temperature, pulse rate, and blood pressure will be recorded.
- Signs and symptoms of malaria will be documented (as expected signs and symptoms of malaria, rather than as AEs).
- Adverse events and concomitant medications will be documented.
- For monitoring of any anti-malarial treatment related AE, each subject will be specifically evaluated for the presence (or absence) of any of the following symptoms:
  - Abdominal pain or discomfort
  - Nausea
  - Vomiting
  - Diarrhea
  - Blurred vision
  - Pruritus
  - Loss of appetite
  - Dizziness
  - Headache
- The severity of malaria signs and symptoms, or anti-malarial treatment related AEs will be graded by using the definitions in Table 19, Table 20, or Table 21.
- Blood samples will be collected for thick smear (for malaria diagnosis) and PCR (for retrospective analysis).
- For infectivity control subjects who participate, PSM data will be downloaded from the subject’s smartphone to a laboratory laptop on Days 7 and 14 (Table 7).
- Blood samples will be collected for transcriptome assays on Days 1 and 7 and on completion of treatment post-CHMI (Table 6).
- For monitoring of any potential cardiac-related AE, each subject will be specifically evaluated for the presence (or absence) of any of the following symptoms:
  - Chest pain or chest pressure
  - Palpitations
  - Lightheadedness
  - Syncope

Any subject who experiences any cardiac-related symptoms will be immediately evaluated by the research physician. The evaluation may include a 12-lead EKG, and immediate referral for higher level care, if indicated.

Malaria diagnosis will be made by microscopic examination of thick smears performed in real time according to a consensus SOP developed by World Health Organization Committee for use in malaria clinical trials ([Moorthy et al-2009](#Moorthy_et_al_2009)).

Subjects who experience malaria symptoms or who are diagnosed with parasitemia based on a positive result on the thick smear microscopic evaluation will be managed according to the following:

- The subject will be called and asked to be seen immediately by the study physician to initiate antimalarial therapy as described in Section 8.2.
- Medical history will be updated, a directed physical examination will be performed if indicated, and temperature, pulse rate, and blood pressure will be collected.
- Signs and symptoms of malaria will be documented as expected signs and symptoms of malaria (or “Malaria Events”) rather than as AEs.
- Adverse events and concomitant medications will be documented, if applicable.
- To monitor for cardiac-related AEs, subjects will be instructed to notify the study team immediately at the onset of any of the following symptoms
  - Chest pain or chest pressure
  - Palpitations
  - Lightheadedness
  - Syncope

Any subject who experiences any cardiac-related symptoms will be immediately evaluated by the research physician. The evaluation may include a 12-lead EKG, and immediate referral for higher level care, if indicated.

- Study subjects symptomatic for malaria will have blood smears collected as frequently as every 6-8 hours and/or at any time post-challenge for a definitive diagnosis of malaria; the frequency of the blood smear collection will be based upon the clinical judgment of the study physician.
- At the time each blood smear is collected, a blood sample will be stored for retrospective PCR analysis.
  - Although PCR will not be used as a diagnostic tool in this trial, PCR is expected to become positive for parasites about 1 to 3 days prior to blood smear.
  - The analysis of PCR results will be useful in terms of comparing the 2 methods (blood smear vs. PCR) and in understanding the kinetics of parasitemia in immunized subjects.
  - PCR will not continue to be collected after malaria diagnosis.
- Blood smear collection will continue daily until the subject is cured of parasitemia, defined as meeting both of these criteria:
  - Two consecutive daily negative blood smears have been recorded and
  - The PI or clinical investigators deem the subject clinically ready for discharge.
- Study subjects who have been cleared for discharge by the study physician can check out of the hotel, and will be asked to return to the CTC for additional visits on Day 28 and Day 35 post-challenge (see Section 8.1.8 and Section 8.1.9).
- Study subjects who have been cleared for discharge and check out of the hotel will be instructed to monitor their symptoms and notify the study team immediately upon the onset of chest pain, chest pressure, palpitations, lightheadedness or syncope.
  - 1. Days 20, 22, 25, and 28 Post Challenge Visits

An immunized individual who does not have complete protection may have a prolongation of the pre-patent period post challenge. Although this has not previously been observed with these vaccine candidates, in theory, the pre-patent period could be longer than 23 days. For this reason, immunized subjects who are still negative 18 days post-challenge will be followed on Days 20, 22, 25, and 28 after challenge. Subjects will be permitted to leave the hotel on Day 18 and will be seen at the CTC on Days 20, 22, 25, and 28 after CHMI. All subjects who are diagnosed, treated, and cleared for discharge from the hotel are to come to the CTC for the Day 28 visit.

During these visits, the following evaluations and procedures will be performed:

- Medical history will be updated, a directed physical examination will be performed, and temperature, pulse rate, and blood pressure will be collected.
- Signs and symptoms of malaria will be documented as expected signs and symptoms of malaria (or “Malaria Events”) rather than as AEs.
- Adverse events and concomitant medications will be documented.
- To monitor for cardiac-related AEs, subjects will be assessed for presence (or absence) of any of the following symptoms:
  - Chest pain or chest pressure
  - Palpitations
  - Lightheadedness
  - Syncope

Any subject who experiences any cardiac-related symptoms will be immediately evaluated by the research physician. The evaluation may include a 12-lead EKG, and immediate referral for higher level care, if indicated.

- Blood samples will be collected (Table 6) for blood smears, safety tests and retrospective PCR analysis.
- For infectivity control subjects, PSM data will be downloaded from the subject’s smartphone to a laboratory laptop on Days 22 and 28 (Table 7)

Subjects who do not develop parasitemia by Day 28 post-challenge will be considered protected and will not receive empiric antimalarial treatment. However, these subjects will be instructed to monitor themselves for signs/symptoms of malaria for the duration of the study and call the clinical investigator for further management should they develop any signs/symptoms suggestive of malaria. The study physician will be accessible 24/7 by telephone to the subjects for consultation.

Infectivity control subjects will be asked to return their devices to the NMRC Clinical Trials Center on Day 28 post-challenge, marking the conclusion of the PSM data collection component of the study.

- - 1. Day 35 Post Challenge Visit

The following evaluations and procedures will be performed at this visit:

- Medical history will be updated, a directed physical examination will be performed, and temperature, pulse rate, and blood pressure will be collected.
- Signs and symptoms of malaria will be documented as expected signs and symptoms of malaria (or “Malaria Events”) rather than as AEs.
- AEs and concomitant medications will be documented.
- Blood samples will be collected for cellular and humoral mediated immunity assays (Table 6).
  - 1. Day 90 Post Challenge (Final Visit)

The following evaluations and procedures will be performed at this visit:

- Medical history will be updated, a directed physical examination will be performed, and temperature, pulse rate, and blood pressure will be collected.
- AEs and concomitant medications will be documented.
- Blood samples will be collected for safety tests and cellular mediated immunity assays (FluoroSpot and ICS) and humoral (ELISA and IFA) immunity assays (Table 6 and Table 7).

All subjects will be instructed to contact the study team any time, within 1 year post-challenge should they experience fever. If subjects leave the study, prior to the passage of 1 year post‑challenge and move outside of the Washington, DC area, they will be instructed to inform their medical care providers that they have been exposed to malaria and the date of exposure.

- - 1. Post-Challenge Telephone Follow-up

Subjects will have a telephone follow up at 6 months and 12 months post‑challenge.

- - 1. Unscheduled Visits

At any unscheduled visits, requested by the subject or the study physician the following evaluations and procedures may occur:

- Medical history will be updated, a physical examination will be performed, and temperature, pulse rate, and blood pressure will be collected.
- Clinically indicated laboratory tests will be performed.
- AEs and concomitant medications will be documented.
- Any other medically-indicated diagnostic or therapeutic procedures will be performed.
  1. Treatment of Malaria for Parasitemic Subjects

Malarone^®^ (250 mg atovaquone/100 mg proguanil tablets) 4 tablets taken orally once per day for 3 days, will be administered as first line therapy. This will be done by directly observed treatment (DOT); that is, a study team physician or nurse will witness the swallowing of the Malarone dose by the subject. In the event that a subject is allergic to, or unable to tolerate Malarone, he or she will be treated with an alternative, but equally effective, antimalarial agent. Malarone-intolerant subjects who have undergone CHMI will receive a standard dose of Coartem as a second line of therapy. Coartem^®^ (artemether/lumefantrine) dosing is 4 tablets taken orally twice daily for 3 days. The third line therapy will be Chloroquine, administered with a total dose of 1500 mg chloroquine base given orally in divided doses: 600 mg initially, followed by 300 mg given approximately at 6 hours, 24 hours, and 48 hours after the initial dose. Subjects will be informed of the potential side effects of the antimalarial treatment. All subjects exposed to malaria will not be eligible to donate blood for 3 years.

On development of symptoms, provided there are no contraindications, subjects may be given acetaminophen (325 to 650 mg every 4 to 6 hours or 1,000 mg every 6 to 8 hours as needed) or ibuprofen (400 mg orally every 6 hours as needed). In case of vomiting, particularly if it interferes with the administration of anti-malarial medication, subjects will be treated with ondansetron (Zofran) 4 to 8 mg by mouth at the onset of nausea or vomiting and the dose may be repeated 8 hours after the initial dose and then 8 mg every 12 hours as needed. Anti-malarial medication administration will be repeated once if vomiting occurs within 30 minutes of initial administration. If vomiting occurs twice, an alternative treatment will be selected (see above).

- 1. Photographs

A study staff member may document local reactions at the vaccination sites by taking digital photographs of the injection site immediately post-vaccination and at the Day 2 and Day 7 follow-up visits. Additional photographs may be taken at other time points if there are local signs and/or symptoms. These photographs are optional and will be taken to allow investigators to better understand the effects of the vaccination. The photograph will only include the subject's arm and no other part of the subject's body. Study staff, study investigators, the ethical review boards, members of a safety committee, and the study sponsor may view these pictures. Each subject may indicate his or her preference regarding photography of the injection sites by initialing the appropriate section in the informed consent document.

- 1. Specimen Collection/Processing/Storage

Blood specimens, for safety testing and research purposes, will be collected by standard venipuncture. Repeated blood collection via venipuncture will be performed at various time points during the screening period, immunization, and challenge period.

In addition to blood collection for screening purposes and post challenge blood smears, blood will also be drawn from the control subjects at the same time points as the immunized subjects during the challenge period and when there is a clinical indication.

The amount of blood drawn will be tracked and will not exceed 5 mL/kg (250 mL for a 50 kg subject) during a 24 hour period and 525 mL over any 8 week period whether drawn at a single time point or at multiple time points.

As part of the informed consent process subjects will be asked to provide permission for the future use of their specimens.

Please see Section 9 for further information on specimen collection/handling/storage.

- 1. Concomitant Medications

This protocol places no restrictions on the use of concomitant medications with the exception of the immunomodulators and immunosuppressants as detailed in Section 7.6, drugs with anti-malarial properties (eg, doxycycline, clindamycin, azithromycin), and drugs that might interfere with anti-malarial treatment in subjects who become parasitemic during the post-challenge phase. Any pre-existing conditions that require routine or intermittent medications should be discussed at the time of screening, and a study physician will determine if participation is safe and will not interfere with any data being collected. If participation is permitted, the concomitant medication(s) will be reviewed with the subject during every study visit and will be recorded on the source document as soon as feasible upon receipt of the information.

Subjects are also instructed to notify the clinical investigator as soon as feasible when they are started on new prescription or over the counter (OTC) medications by their primary care physicians to ensure the safety of the subject and integrity of the data being collected. Information regarding all OTC and/or prescription medications taken will be solicited and recorded at each scheduled study visit.

The investigator may recommend medication(s) for symptomatic relief, if necessary, during post-immunization or post-challenge periods and these medications will also be documented on the Concomitant Medication form. Information from subjects pertaining to their use of concomitant medications will be collected from screening through Day 90 post CHMI.

- 1. Procedures for Monitoring Subject Compliance

If a subject fails to attend a scheduled study visit, the PI or his or her designee will make a reasonable effort to determine the reason for the attendance failure. Telephone calls, registered letters, email correspondence, SMS text messages, and the use of social media tools, such as the private messaging features of social media sites like Facebook, are considered appropriate means of communication. If a study subject is deemed to be unreliable for future study visits, he or she may be withdrawn from the study based upon the judgment of the PI. For subjects being withdrawn from the study, a targeted examination may be performed, if medically indicated and permitted by the subject.

For infectivity control subjects that are participating in PSM data collection, subjects may receive a daily phone call or text reminder to wear their device and charge it appropriately.

Immunogenicity Assays

- 1. Cellular Assays

Vaccine-induced T-cell responses at various time points will be assessed using the cytokine FluoroSpot assay and multiparameter flow cytometry assay. PBMCs will be stimulated with synthetic peptides derived from PfCSP, PfAMA, and Pf-TRAP to assess the frequency of T‑cell responses that produce IFN-γ, IL-2, Granzyme B, and TNF-α. The cellular immunogenicity assays will be conducted at the following time points using the blood volumes specified in Table 9.

Table 9: Blood Volumes Required for Cellular Immunogenicity Assays

| Time point | Blood Volume (mL) |
| --- | --- |
| Infectivity Controls: |  |
| 1. Pre CHMI: (Day -7) | 140 |
| 2. Post CHMI: 5 weeks (Day 35) | 140 |
| 3. Post CHMI: 12 weeks (Day 90) | 140 |
| Groups 1 and 2 |  |
| 1. Pre immunization: Day -14 to -1 | 140 |
| 2. Post DNA prime immunization 3: 4 weeks (Day 84 relative to the first immunization) | 140 |
| 3. Day of ChAd63 boost immunization | 140 |
| 4. Pre CHMI: (Day 27 post ChAd63 boost immunization) | 140 |
| 5. Post CHMI: 5 weeks (Day 35 relative to CHMI) | 140 |
| 6. Post CHMI: 12 weeks (Day 90 relative to CHMI) | 140 |

Cellular immunogenicity assays will be performed using both fresh and frozen PBMCs. Assays using fresh PBMCs were performed for previous clinical trials (AdCA dose-escalation trial with no CHMI [IND 13003], AdC trial with CHMI [IND 13003], DNA/AdCA trial with CHMI [IND 13977], and AdCA-alone trial with CHMI [IND 13003]) to allow for a comparison in the magnitude of responses among past NMRC and University of Oxford studies. The use of fresh PBMCs is a critical feature of the immunologic testing. This is well known in the literature, and the NMRC immunology team has previously shown that the magnitude of responses obtained with frozen PBMCs is often lower when compared to that obtained with fresh samples. This also allows for comparability of data from the clinical trials recently conducted by the University of Oxford.

We have determined that the cellular immunology assays described require approximately 70 mL of whole blood. Any excess blood from subjects who provide consent for use of their samples in future studies will be stored. The remaining PBMCs will be cryopreserved and may be used for exploratory studies such as:

- Evaluation of a T-cell based vaccine targeting important epitopes from multiple antigens.
- Analysis of the amino acid variation in the identified protection-associated sequences in the Pf 7G8 strain.
- If protection associated epitopes are found, analyses to determine if immune PBMCs from protected subjects would recognize variant protective epitopes from the *Plasmodium falciparum* 7G8 strain that differ from the vaccine (3D7) strain.
  1. Antibody Assays

Antibody assays will include ELISA against PfCSP NANP repeat peptide, N- and C-terminal peptides, and full-length recombinant protein, PfAMA1 recombinant protein, and Pf-TRAP recombinant protein. Antibody levels will also be assessed against sporozoite- and erythrocyte‑stage parasites using IFA. Blood will be drawn for sera and antibody analyses at the time points indicated in Table 10.

Table 10: Blood Volume Requirements for Antibody Assays

| Time point | Blood Volume (mL) |
| --- | --- |
| Infectivity Controls |  |
| 1. Pre CHMI: (Day -7) | 10 |
| 1. Post CHMI: 5 weeks (Day 35) | 10 |
| 1. Post CHMI: 12 weeks (Day 90) | 10 |
| Groups 1 and 2 |  |
| 1. Pre immunization (Day -7) | 10 |
| 1. Post DNA prime immunization 1 (Day 14) | 10 |
| 1. Day of DNA prime immunization 2 | 10 |
| 1. Post DNA prime immunization 2 (Day 14) | 10 |
| 1. Day of DNA prime immunization 3 | 10 |
| 1. Post DNA prime immunization 3 (Day 14) | 10 |
| 1. Post DNA prime immunization 3 (Day 28) | 10 |
| 1. Day of ChAd63 boost immunization | 10 |
| 1. Post ChAd63 boost immunization (Day 27) | 10 |
| 1. Post CHMI: 5 weeks (Day 35) | 10 |
| 1. Post CHMI: 12 weeks (Day 90) | 10 |

The immunogenicity assays conducted at the time points indicated in the study event schedules (Table 5, Table 6, and Table 7) are described in Table 11.

Table 11: Immunogenicity Assays

| Assay | Sample (mL) | Description | Laboratory |
| --- | --- | --- | --- |
| Cytokine FluoroSpot | Blood collected in preservative-free heparinized syringes for PBMC separation (140 mL for Immunization Groups 1 and 140 mL for Immunization Group 2) | The FluoroSpot assay is based on ELISpot but utilizes fluorescent-based detection systems, enabling the detection of cells secreting either of 2 different cytokines, or both, in the same cell. Two types of FLUOROSpot kits will be used: The IFNg/IL2 kit will simultaneously measure IFN-γ, IL-2, and IFN-γ + IL-2, while the IFN-γ/Granzyme B kit will simultaneously measure IFN-γ, Granzyme B, and IFN-γ + Granzyme B cytokines in response to stimulation with synthetic peptides derived from PfCSP, PfAMA, and Pf-TRAP. Readout: Magnitude of cytokine responses to vaccine antigens and frequency of responders in a population. | USMMVP  NMRC Malaria Department Clinical Immunology Laboratory |
| Flow Cytometry (ICS) | Included in the 140 mL blood sample (from Immunization Groups 1 and 2) for FluoroSpot | This assay measures the frequency of T-cell subsets producing IFN-γ, TNF-α, and IL2 cytokine secretion by flow cytometry in response to stimulation with synthetic peptides derived from PfCSP, PfAMA, and Pf-TRAP. CD4+ and CD8+ T cells will be characterized for activation status, intracellular content of IFN-γ, TNF-α, and IL2. Readout: Phenotype, magnitude activation status, and frequency of cytokine-producing cells | USMMVP  WRAIR Malaria Vaccine Branch Flow Cytometry Center |
| ELISA | 10 mL of blood | Antibody titers will be assessed against PfCSP NANP repeat peptide, N- and C-terminal peptides, and full-length recombinant protein, PfAMA1 recombinant protein, and Pf-TRAP recombinant protein. Readout: Data will be reported as the serum dilution at which the optical density equals 0.5 (OD 0.5). | ELISAs against CSP and AMA1: USMMVP, WRAIR Malaria Vaccine Branch Serology Laboratory  ELISA against TRAP: University of Oxford |
| IFA | Included in the 10 mL blood sample for ELISA | Antibody levels will be assessed against sporozoite- and erythrocyte-stage parasites. Readout: Serum dilution at which there will be the least immunofluorescence detected. | USMMVP  WRAIR Malaria Vaccine Branch Serology Laboratory |
| Transcriptome Analysis | 2.5 mL | PBMC transcriptome shortly after vaccination reflect the innate immune responses. RNA will be stabilized and purified using PAXGene blood collection tubes and RNA kits. RNA will also be extracted from cryopreserved PBMCs at specific time points. RNA sequences will be detected de novo on an Illumina HiSeq2000, or equivalent, instrument. | TBD |
| HuAd5 Serology | Included in the 10 mL blood sample for ELISA | HuAd5 serology will be assessed by an in vitro assay that evaluates the ability of human sera (that may contain HuAd5 neutralizing antibodies due to a previous HuAd5 infection) to block a recombinant HuAd5-luciferase virus from infecting cells and producing a luminescent signal. | USMMVP  NMRC, Dr. Limbach’s laboratory |

([Duncan et al-2011](#Duncan_et_al_2011) and [M](#Miuraetal2013)[i](#Miuraetal2013)[u](#Miuraetal2013)[r](#Miuraetal2013)[a](#Miuraetal2013)[e](#Miuraetal2013)[t](#Miuraetal2013)[a](#Miuraetal2013)[l](#Miuraetal2013)[-](#Miuraetal2013)[2](#Miuraetal2013)[0](#Miuraetal2013)[1](#Miuraetal2013)[3](#Miuraetal2013).)

Physiological Status Monitoring

- 1. Wearable Devices

Infectivity Control Research subjects will be issued a commercial off-the-shelf (COTS) smartwatch (Samsung Gear S3 or equivalent) along with a smartphone. The smartphone (Samsung Galaxy S8 or equivalent) automatically downloads and stores the data from the wearable devices wirelessly via Bluetooth.

The Samsung Gear S3 is a smartwatch that is worn on the left wrist and measures heart rate, skin temperature, and physical activity in the form of 3-axis accelerometer data. It has a battery life of approximately 30 hours, must be charged daily, and requires approximately 2 to 3 hours to fully charge. The smart watch stores up to 24 hours of vital signs data on its on-board memory.

The Samsung Galaxy Note S8 is a smartphone used to store data collected by the smartwatch. Data is transferred automatically from the smart watch to the smartphone wirelessly via Bluetooth. The smartphone will be locked and will not be useable for any purpose other than data collection. The smartphone will have an application that will allow subjects to view a summary of the vital sign data they collected throughout the course of the study. This will include both the number of hours of data collected each day of the study, as well as a visualization of the summary of the vital sign statistics for the days where data was collected.

- 1. Data Collection

Subjects will be wearing PSM devices that continuously monitor vital signs from approximately two weeks prior to challenge until 4 weeks post-challenge. Subjects will be instructed on the appropriate use of the devices, including battery charging strategies, device placement, and suggested wearing schedules. Subjects will be asked to wear the smartwatch for at least 12 hours a day, every day from up to two weeks prior to challenge to 4 weeks post-challenge (approximately 42 days in total). In order to meet that 12‑hour minimum of daily data collection, the subject is free to wear the device while awake, during sleep, or while carrying out routine activities. The data from the smartwatches will be automatically and wirelessly uploaded to the smartphone whenever the wearable devices are in range. Subjects will be responsible for charging the smartwatch and smartphone daily, using the provided charging kit, which also automatically uploads the data to the smartphone.

During the data-collection phase of the study, data will be uploaded from the smartphone to BHSAI laptop during each weekly visit. The data will be recorded by Screening Identification Numbers (SIDs) and Device ID and thus is coded to a specific individual. Data will be transferred to BHSAI periodically throughout the study and stored in a BHSAI secure server indefinitely. BHSAI will also receive coded data on the parasitemia results, from blood smear, in order to determine how PSM data is predictive of infection status.

- 1. Data Analysis Plan

The total duration for PSM data collected will be determined for all subjects for the smartwatch (in Hours) for each day of the study. Compliance rate will be calculated for each subject based on the percentage of days that subject wore the device for at least 12 hours.

The PSM data collected for this trial will not be analyzed as a part of this trial. Under a separate research agreement, the PSM data and corresponding parasitemia data (blood film and/or qPCR) will be transferred in de-identified form to BHSAI for analysis under a separate non-human subjects protocol. BHSAI will employ multiple approaches involving signal processing and pattern recognition of time-series data, including autoregressive models and machine learning algorithms, to detect vital-sign deviations that are associated with a change in infection status. This will enable us to identify which vital-sign measures are most predictive of infection, allowing us to develop a model for predicting the likelihood of infection directly from continuous vital-sign data.

Safety Assessment

Safety monitoring will be conducted throughout the study; therefore safety concerns will be identified by continuous review of the data by the PI, clinic staff, clinical monitor, research monitor, and United States Army Medical Research and Materiel Command (USAMRMC) Office of Regulated Activities (ORA), Product Safety Surveillance Branch (PSSB).

**S****t****u****d****y** **S****a****f****e****t****y** **M****a****n****a****g****e****m****e****n****t****:** The IRB, research monitor, and principal investigator will review any safety concern. A data safety monitoring board (DSMB) and safety monitoring committee (SMC) are not required for this study because both the DNA vaccine products and the adenovirus vaccine products have been previously evaluated in human studies. These data have been published and safety in humans has been demonstrated ([C](#Chuang_et_al_2013)[h](#Chuang_et_al_2013)[u](#Chuang_et_al_2013)[a](#Chuang_et_al_2013)[n](#Chuang_et_al_2013)[g](#Chuang_et_al_2013)[e](#Chuang_et_al_2013)[t](#Chuang_et_al_2013)[a](#Chuang_et_al_2013)[l](#Chuang_et_al_2013)[-](#Chuang_et_al_2013)[2](#Chuang_et_al_2013)[0](#Chuang_et_al_2013)[1](#Chuang_et_al_2013)[3](#Chuang_et_al_2013), [T](#Tamminga_et_al_2011)[a](#Tamminga_et_al_2011)[m](#Tamminga_et_al_2011)[m](#Tamminga_et_al_2011)[i](#Tamminga_et_al_2011)[n](#Tamminga_et_al_2011)[g](#Tamminga_et_al_2011)[a](#Tamminga_et_al_2011)[e](#Tamminga_et_al_2011)[t](#Tamminga_et_al_2011)[a](#Tamminga_et_al_2011)[l](#Tamminga_et_al_2011)[-](#Tamminga_et_al_2011)[2](#Tamminga_et_al_2011)[0](#Tamminga_et_al_2011)[1](#Tamminga_et_al_2011)[1](#Tamminga_et_al_2011), and [T](#Tamminga_et_al_2013)[a](#Tamminga_et_al_2013)[m](#Tamminga_et_al_2013)[m](#Tamminga_et_al_2013)[i](#Tamminga_et_al_2013)[n](#Tamminga_et_al_2013)[g](#Tamminga_et_al_2013)[a](#Tamminga_et_al_2013)[e](#Tamminga_et_al_2013)[t](#Tamminga_et_al_2013)[a](#Tamminga_et_al_2013)[l](#Tamminga_et_al_2013)[-](#Tamminga_et_al_2013)[2](#Tamminga_et_al_2013)[0](#Tamminga_et_al_2013)[1](#Tamminga_et_al_2013)[3](#Tamminga_et_al_2013) for the DNA and HuAd5 prime boost vaccines, and [O](#OHara_et_al_2012)[’](#OHara_et_al_2012)[H](#OHara_et_al_2012)[a](#OHara_et_al_2012)[r](#OHara_et_al_2012)[a](#OHara_et_al_2012)[e](#OHara_et_al_2012)[t](#OHara_et_al_2012)[a](#OHara_et_al_2012)[l](#OHara_et_al_2012)[-](#OHara_et_al_2012)[2](#OHara_et_al_2012)[0](#OHara_et_al_2012)[1](#OHara_et_al_2012)[2](#OHara_et_al_2012), [E](#Ewer_et_al_2013)[w](#Ewer_et_al_2013)[e](#Ewer_et_al_2013)[r](#Ewer_et_al_2013)[e](#Ewer_et_al_2013)[t](#Ewer_et_al_2013)[a](#Ewer_et_al_2013)[l](#Ewer_et_al_2013)[-](#Ewer_et_al_2013)[2](#Ewer_et_al_2013)[0](#Ewer_et_al_2013)[1](#Ewer_et_al_2013)[3](#Ewer_et_al_2013), [S](#Sedegah_et_al_2011)[h](#Sedegah_et_al_2011)[e](#Sedegah_et_al_2011)[e](#Sedegah_et_al_2011)[h](#Sedegah_et_al_2011)[y](#Sedegah_et_al_2011)[e](#Sedegah_et_al_2011)[t](#Sedegah_et_al_2011)[a](#Sedegah_et_al_2011)[l](#Sedegah_et_al_2011)[-](#Sedegah_et_al_2011)[2](#Sedegah_et_al_2011)[0](#Sedegah_et_al_2011)[1](#Sedegah_et_al_2011)[1](#Sedegah_et_al_2011), [S](#Sheehy_et_al_2012)[h](#Sheehy_et_al_2012)[e](#Sheehy_et_al_2012)[e](#Sheehy_et_al_2012)[h](#Sheehy_et_al_2012)[y](#Sheehy_et_al_2012)[e](#Sheehy_et_al_2012)[t](#Sheehy_et_al_2012)[a](#Sheehy_et_al_2012)[l](#Sheehy_et_al_2012)[-](#Sheehy_et_al_2012)[2](#Sheehy_et_al_2012)[0](#Sheehy_et_al_2012)[1](#Sheehy_et_al_2012)[2](#Sheehy_et_al_2012)[a](#Sheehy_et_al_2012), [S](#Sheehy_et_al_2012b)[h](#Sheehy_et_al_2012b)[e](#Sheehy_et_al_2012b)[e](#Sheehy_et_al_2012b)[h](#Sheehy_et_al_2012b)[y](#Sheehy_et_al_2012b)[e](#Sheehy_et_al_2012b)[t](#Sheehy_et_al_2012b)[a](#Sheehy_et_al_2012b)[l](#Sheehy_et_al_2012b)[-](#Sheehy_et_al_2012b)[2](#Sheehy_et_al_2012b)[0](#Sheehy_et_al_2012b)[1](#Sheehy_et_al_2012b)[2](#Sheehy_et_al_2012b)[b](#Sheehy_et_al_2012b), and [H](#Hodgson_et_al_2014)[o](#Hodgson_et_al_2014)[d](#Hodgson_et_al_2014)[g](#Hodgson_et_al_2014)[s](#Hodgson_et_al_2014)[o](#Hodgson_et_al_2014)[n](#Hodgson_et_al_2014)[e](#Hodgson_et_al_2014)[t](#Hodgson_et_al_2014)[a](#Hodgson_et_al_2014)[l](#Hodgson_et_al_2014)[-](#Hodgson_et_al_2014)[2](#Hodgson_et_al_2014)[0](#Hodgson_et_al_2014)[1](#Hodgson_et_al_2014)[4](#Hodgson_et_al_2014) for the ChAd63 vaccines)

**R****e****s****e****a****r****c****h** **M****o****n****i****t****o****r****:** The Department of Defense research monitor is responsible for overseeing the safety of the research and report observations/findings to the IRB or a designated institutional official. The research monitor will review all unanticipated problems involving risks to subjects or others associated with the protocol and provide an independent report of the event to the IRB. The research monitor may discuss the research protocol with the investigators; shall have authority to stop a research protocol in progress, remove individual human subjects from a research protocol, and take whatever steps are necessary to protect the safety and well-being of human subjects until the IRB can assess the monitor’s report; and shall have the responsibility to promptly report their observations and findings to the IRB or other designated official and the HRPO.”

In addition to the responsibilities above the research monitor is required to review and provide an unbiased written report for all SAEs and subject deaths to the USAMRMC PSSB (Safety Office) within 24 hours of their awareness of the event using the Research Monitor Report form. The report provided must include, at a minimum, a brief summary of the research monitor's review of the event and event outcome, relationship of the event to the investigational product and whether or not the research monitor concurs with the details of the study investigator's report. For this protocol the secondary research monitor will provide input in the event of a discrepancy between the investigator’s report and the primary research monitor’s report.

**Office of Regulated Activities, Product Safety Surveillance Branch****:** The PSSB is responsible for coordinating and integrating the review of safety data regarding The Surgeon General (TSG) Department of the Army-sponsored products. The PSSB reviews each SAE report for medical consistency, accuracy, and completeness and follows each event until it is satisfactorily resolved. The ORA Safety Pharmacovigilance (PVG) physician, as delegated by the sponsor, evaluates all safety cases and provides the final determination on relatedness to the product, and whether expedited reporting is warranted, per current FDA regulation and guidance.

- 1. Specification of Safety Endpoints

Safety endpoints are listed in Section 6.1.

- 1. IND Safety Reporting

The following terms, as defined by 21 CFR 312.32, apply to IND safety reporting. All AEs, regardless of vaccine group or suspected causal relationship to the investigational product will be reported as described in the following sections.

- - 1. Adverse Event or Suspected Adverse Reaction

Adverse event means any untoward medical occurrence associated with the use of a drug, vaccine or other intervention in humans, whether or not considered drug related.

Suspected adverse reaction means any adverse event for which there is a reasonable possibility that the intervention caused the adverse event. For the purposes of IND safety reporting, “reasonable possibility” means there is evidence to suggest a causal relationship between the intervention and the adverse event. Suspected adverse reaction implies a lesser degree of certainty about causality than adverse reaction, which means any adverse event caused by a drug.

The occurrence of solicited adverse events will be determined/captured from the information provided by the memory aid completed daily by each subject for 7 days after each immunization, medical history update, and directed physical examination performed by investigators , and temperature, pulse rate, blood pressure measurements performed during the subject follow up visits as specified in Table 5, Table 6, and Table 7. The occurrence of unsolicited adverse events will be determined from the medical history and focused physical examination performed by the clinical investigators, temperature, pulse rate and blood pressure measured and safety laboratory tests performed during the follow-up visits as specified in Table 5, Table 6, and Table 7.

- - 1. Solicited Adverse Event

A solicited AE is a predetermined event, identified in the Investigator's Brochure, which may reflect safety concerns related to the investigational product. The solicited AEs for this study are shown in Table 12.

Table 12: Local and Systemic Solicited Adverse Events

| Local (injection site) | Systemic | |
| --- | --- | --- |
| Pain  Swelling  Redness  Tenderness  Induration  Ecchymosis/Bruising  Pruritus/Itching  Warmth  Paresthesia  Scaling | Fever (oral, T>100.4°F)  Fever (subjective)  Chills  Rigors  Headache  Vomiting  Nausea  Dizziness  Malaise  Abdominal pain | Fatigue  Myalgia  Arthralgia  Ipsilateral adenopathy  Regional adenopathy  Flu-like syndrome  Cough |

- - 1. Serious Adverse Event or Serious Suspected Adverse Reaction

An adverse event or suspected adverse reaction is considered “serious” if, in the view of either the investigator or sponsor, it results in any of the following outcomes:

- Death
- Life-threatening adverse event: an adverse event or suspected adverse reaction is considered “life-threatening” if, in the view of either the investigator or sponsor, its occurrence places the patient or subject at immediate risk of death. It does not include an adverse event or suspected adverse reaction that, had it occurred in a more severe form, might have caused death.
- Inpatient hospitalization or prolongation of existing hospitalization
- Persistent or significant incapacity or substantial disruption of the ability to conduct normal life functions
- Congenital anomaly/birth defect
- Important medical events that may not result in death, be life-threatening, or require hospitalization may be considered serious when, based upon appropriate medical judgment, they may jeopardize the patient or subject and may require medical or surgical intervention to prevent one of the outcomes listed in this definition. Examples of such medical events include allergic bronchospasm requiring intensive treatment in an emergency room or at home, blood dyscrasias or convulsions that do not result in inpatient hospitalization, or the development of drug dependency or drug abuse.
  - 1. Unexpected Adverse Event or Unexpected Suspected Adverse Reaction

An adverse event or suspected adverse reaction is considered "unexpected" if it is not listed in the investigator brochure or is not listed at the specificity or severity that has been observed; or, if an investigator brochure is not required or available, is not consistent with the risk information described in the general investigational plan or elsewhere in the current application, as amended. For example, under this definition, hepatic necrosis would be unexpected (by virtue of greater severity) if the investigator brochure referred only to elevated hepatic enzymes or hepatitis. Similarly, cerebral thromboembolism and cerebral vasculitis would be unexpected (by virtue of greater specificity) if the investigator brochure listed only cerebral vascular accidents. "Unexpected," as used in this definition, also refers to adverse events or suspected adverse reactions that are mentioned in the investigator brochure as occurring with a class of drugs or as anticipated from the pharmacological properties of the drug, but are not specifically mentioned as occurring with the particular drug under investigation.

- - 1. Unanticipated Problems Involving Risks To Subjects Or Others

Federal regulations require that unanticipated problems involving risks to subjects or others be promptly reported to the IRB. These events encompass a broader category of events than SAEs and may include issues such as problems with loss of control of subject data or the investigational product; adverse psychological reactions; or breach of confidentiality. Risks to others (eg, program personnel) must also be reported.

Unanticipated problems involving risks to subjects or others are any incident, experience, or outcome that meets all of the following criteria:

- Unexpected (in terms of nature, severity, or frequency) given (a) the procedures that are described in the protocol, investigators brochure or informed consent document; and (b) the characteristics of the subject population;
- Related or possibly related to a subject's participation in the study; and
- Suggests that the study places subjects or others at a greater risk of harm than was previously known or recognized.

The IRB and the ORP will evaluate the PI's and research monitor's reports to determine whether a given incident, experience or outcome constitutes an unanticipated problem involving risk to subjects or others to the investigative sites and appropriate regulatory offices.

- - 1. Relationship to Investigational Product

The investigator must assign a relationship of each AE to the receipt of the investigational product using the categories shown in Table 13. The investigator will use clinical judgment in conjunction with the assessment of a plausible biologic mechanism, a temporal relationship between the onset of the event in relation to receipt of the investigational product, and identification of possible alternate etiologies including underlying disease, concurrent illness or concomitant medications. The following guidelines should be used by investigators to assess the relationship of an AE to study product administration. **Only a physician can make this determination.**

Table 13: Categories for Adverse Event Relationship to Investigational Product

| Category | Description |
| --- | --- |
| **Not related** | No relationship to investigational product. Applies to those events for which evidence exists that there is an alternate etiology. |
| **Unlikely** | Likely unrelated to the investigational product. Likely to be related to factors other than investigational product, but cannot be ruled out with certainty. |
| **Possible** | An association between the event and the administration of investigational product cannot be ruled out. There is a reasonable temporal association, but there may also be an alternative etiology such as the subject’s clinical status or underlying factors including other therapy. |
| **Probable** | There is a high degree of certainty that a relationship to the investigational product exists. There is a reasonable temporal association, and the event cannot be explained by known characteristics of the subject’s clinical state or factors including other therapy. |
| **Definite:** | An association exists between the receipt of investigational product and the event. An association to other factors has been ruled out. |

- - 1. Severity Assessment

All AEs will be assessed for severity by the investigator using the grading scales in Appendix E. Inherent in this assessment is the medical and clinical consideration of all information surrounding the event including any medical intervention required. The criteria in Table 14 may be used for any symptom not included in the grading scales.

Table 14: Adverse Event Severity Categories

| Severity Category | AE Grade | Description |
| --- | --- | --- |
| **Mild** | 1 | Does not interfere with routine activities  Minimal level of discomfort |
| **Moderate** | 2 | Some interference with routine activities  Requires no medical intervention  Moderate level of discomfort |
| **Severe** | 3 | Significant interference with routine activities  Requires medical intervention  Unable to perform routine activities  Significant level of discomfort |
| **Potentially life-threatening** | 4 | Hospitalization or ER visit for either a medically important event or a potentially life-threatening event |
| **Fatal** | 5 | Results in death |

Each event will be assigned one of the following categories: mild, moderate, severe, potentially life-threatening, or fatal. Any Grade 4 (potentially life-threatening) or Grade 5 (fatal) AE must be reported as an SAE.

If a subject is evaluated in an emergency room for non-life-threatening illness or symptoms (ie, visits emergency department on weekend for mild problems because the physician's office is closed), the information from that visit will be reviewed and severity of the adverse event will be assessed according to the subject's clinical signs and symptoms.

As defined by the ICH guideline for GCP, the term “severe” is often used to describe intensity (severity) of a specific event (as in mild, moderate, or severe myocardial infarction); the event itself however, may be of relatively minor medical significance (such as severe headache). This is not the same as "serious," which is based on subject/event outcome or action criteria usually associated with events that pose a threat to a subject's life or functioning. Seriousness (not severity) serves as a guide for defining regulatory reporting obligations.

The source records and eCRFs for AEs will reflect only the highest severity for continuous days an event occurred.

- 1. Recording Adverse Events
     1. Methods/Timing for Assessing, Recording, and Analyzing Safety Endpoints

Solicited and unsolicited AEs, and SAEs will be assessed at all study visits, documented in the source records/eCRFs and electronic database using accepted medical terms and/or the diagnoses that accurately characterize the event. It should be noted that the form for collection of SAE information is not the same as the AE CRF. Where the same data are collected, the forms must be completed in a consistent manner. The same AE terms, for example, should be used on both forms. When a diagnosis is known, the AE term recorded on the source records/eCRFs will be the diagnosis rather than a constellation of symptoms. The PI or clinical investigator will assess all AEs for seriousness, relationship to investigational product, severity, and other possible etiologies and record the following on the AE CRF: a description of the event (if the event consists of a cluster of signs and symptoms, a diagnosis should be recorded rather than each sign and symptom); onset date and time; stop date and time; intensity (recorded as mild, moderate, or severe, potentially life threatening, or fatal); seriousness (an SAE Report Form must also be completed for SAEs); causality (relationship to study drug); outcome (eg, not recovered/not resolved (ongoing), recovering/resolving, recovered/resolved, recovered/resolved with sequelae, death, or unknown (if applicable); and action taken with the study agent (eg, no action, study agent discontinued, or other action taken. When an event has not resolved by study closure, it will be documented on the AE CRF as “not recovered/not resolved”.

- - - 1. Post Immunization

Solicited AEs will be assessed starting on the day of immunization (following receipt of vaccine) through Day 7 after immunization; unsolicited AEs will be assessed starting on the day of immunization (following receipt of vaccine) through Day 28 after immunization; occurrence of laboratory abnormalities will be assessed starting on the day of immunization (following receipt of vaccine) through Day 28 after each immunization. The occurrence of serious adverse events, as defined in 21 CFR 312.32, will be assessed starting on the day of immunization (following receipt of vaccine) and continue for up to 12 months after CHMI. All SAEs that occur during the active phase of the study (period starting on the day of the first immunization up to 3 months post CHMI) will be reported as outlined in Section 11.2. All SAEs will be recorded and reported to the sponsor’s safety office.

- - - 1. Following CHMI

Unsolicited signs and symptoms will be collected through Day 28 post challenge. Solicited local signs and symptoms will be collected through Day 7 post challenge and solicited systemic signs and symptoms will be collected through Day 7.

Starting at Day 7 post-challenge, subjects will be monitored for signs and symptoms consistent with malaria infection. These will be documented on a Malaria Events form but will not be categorized as adverse events related to immunization. Safety laboratories will be collected on the day of the challenge, at the time of first detection of parasitemia, approximately 72 hours later, and on Day 28 post-challenge.

Subjects will be closely monitored for the occurrence of any cardiac-related AEs. Each subject who underwent CHMI will be specifically assessed for the presence (or absence) of chest pain or pressure, palpitations, lightheadedness or syncope during all of the post-immunization visits. Subjects will be instructed to promptly report the occurrence of any of these symptoms to the study team, who can be readily accessed during the hotel phase.

- - 1. Duration of Follow-Up of Subjects after Adverse Events

Investigators are required to follow SAEs to resolution, even if this extends beyond the prescribed reporting period. Resolution is the return to baseline status or stabilization of the condition with the probability that it will become chronic. The SAE outcomes will be reported to the USAMRMC Office of Regulated Activities, Product Safety Surveillance Branch, SAE mailbox: usarmy.detrick.medcom-usamrmc.mbx.sae-reporting@mail.mil using the Serious Adverse Event Report Form.

Investigators are not obligated to actively seek SAEs in former subjects; however, if a SAE, considered to be related to the investigational product is brought to the attention of the investigator at any time following completion of the study, the event will be reported to the sponsor's safety office as defined in Section 11.4.1.1.

- 1. Reporting Adverse Events

The PI will report all AEs to the USAMRMC, the local IRB, the USAMRMC ORP, and collaborators, in the appropriate safety, annual, and/or final reports. After appropriate data cleaning and query resolution between the clinical site, sponsor's clinical monitor, and clinical data manager, SAEs from the clinical database will be reconciled with the sponsor's SAE database. SAEs and AEs for inclusion in annual and final reports to the FDA will come from the clinical database.

- - 1. Reporting Serious and Unexpected Adverse Events

Contact information for reporting SAEs is provided in Table 15.

Table 15: Study Contacts for Reporting Serious Adverse Events and Unanticipated Problems Involving Risk to Patients or Others

| **Sponsor’s Safety Office** | US Army Medical Research & Materiel Command ATTN: MCMR-UMR 1430 Veterans Drive Fort Detrick, MD 21702-5009 Telephone: 301-619-1005 Fax: 301-619-7790  Email: usarmy.detrick.medcom-usamrmc.mbx.sae-reporting@mail.mil |
| --- | --- |
| **Institutional Review Boards** | Naval Medical Research Center Office of Research Administration 503 Robert Grant Avenue Silver Spring, MD 20910 Office: 301-319-7276 Fax: 301-319-7277 |
|  | Human Research Protection Office US Army Medical Research and Materiel Command,  ATTN: MCMR-RPH 504 Scott Street Fort Detrick, MD 21702-5012 Telephone: 301-619-2165 Fax: 301-619-7803  Email: usarmy.detrick.medcom-usamrmc.other.hrpo@mail.mil |
| **Research Monitor** | Charmagne G Beckett, MD, MPH, FACP, CAPT, MC, USN Naval Medical Research Center Infectious Diseases Directorate 503 Robert Grant Ave Silver Spring, Maryland 20910 Desk 301-319-7662 Cell 301-919-2350 Email: [charmagne.g.beckett.mil@mail.mil](mailto:charmagne.g.beckett.mil@mail.mil) |
|  | Alexandra Singer, MD, LCDR, MC, USN Defense Health Agency  J3/Public Health 7700 Arlington Blvd, Suite 3M348A Falls Church, VA 22042-5143 Office: (703) 681-6866 Cell: (803) 553-2447 Email: alexandra.l.singer.mil@mail.mil |

- - - 1. Reporting to the Sponsor

All SAEs must be reported promptly (within 24 hours) to the sponsor's representative as per 21 CFR 312.64, whether or not the event is considered related to study product. All notifications must, at a minimum, be reported by emailing or faxing the initial SAE Report Form to the sponsor's safety office the Office of Regulated Activities, Product Safety Surveillance Branch. Further, the investigator should comply with relevant study site SOPs on reporting SAEs.

The minimum information that the investigator will provide to the Office of Regulated Activities Product Safety Surveillance Branch is specified in Table 16. The sponsor's representative may request additional information for purposes of the study.

Table 16: SAE Information to be Reported to the Sponsor's Safety Office

| Notification Method | Information to be Provided |
| --- | --- |
| Email or Telephone (within 24 hours) | IND number, sponsor study number, name of the investigational product, and investigator name and contact number |
|  | Subject identification number |
|  | SAE term, description, onset date, date(s) of investigational product administration, severity, relationship, and subject’s current status |
| AND |  |
| Email or Fax | Cover sheet or letter |
|  | Serious adverse event report form |
|  | Medical record progress notes including pertinent laboratory/diagnostic test results |
| NOTE: When submitting SAE reports via email, the subject line of each email notification will read as follows:  **SAFETY REPORT – IND # _17572_, Sponsor Study #_S-14-07_, Subject# _____, Event term: _____** | |

In order to comply with regulations mandating sponsor notification of specified SAEs to the FDA within 7 calendar days, investigators must submit additional information as soon as it is available. The sponsor's representative will report unexpected SAEs associated with the use of the drug or vaccine to the FDA as specified at 21 CFR 312.32 (c).

Investigators must follow all relevant regulatory requirements as well as specific policy regarding the timely reporting of SAEs to the research monitor and the local IRB and the USAMRMC ORP.

Reporting to the sponsor's safety office does not fulfill the investigator's duty to report all unanticipated problems involving risk to human subjects or others to the IRB. The PI will notify the local IRB and the USAMRMC ORP, and the research monitor.

- - - 1. Reporting to NMRC IRB

Unanticipated problems involving risk to subjects or others, SAEs related to participation in the study, and all subject deaths related to participation in the study should be promptly reported by telephone, email, or fax to the NMRC Institutional Review Board (IRB) and/or the USAMRMC ORP within 24 hours of knowledge of occurrence. A complete written report should follow the initial notification.

Investigators are required to forward safety information provided by the sponsor's representative to the IRB.

- - 1. Reporting Additional Immediately Reportable Events to the Sponsor's Safety Office and Local IRB and the USAMRMC ORP
       1. Pregnancy

Each pregnancy must be reported immediately (within 24 hours of identification) by completing and submitting the Pregnancy Report Form by email or fax to the sponsor's safety office (Office of Regulated Activities, Product Safety Surveillance Branch). Report the incident to NMRC IRB and the USAMRMC ORP in accordance with IRB policy.

Subjects who become pregnant after Day 0 will be followed to term, and the following information will be gathered and documented on the Pregnancy Report for: outcome, type and date of delivery, Apgar scores, and health status of the mother and child including the child's sex, head circumference, gestational age at delivery, length, and weight. Complications and or abnormalities should be reported including any premature terminations. A pregnancy is reported as an AE or SAE only when there is suspicion that the investigational product may have interfered with the effectiveness of contraception or there was a serious complication in the pregnancy including a spontaneous abortion or an elective termination for medical rationale. Otherwise, pregnancy will not be categorized as an adverse event, but it will be reported in a similar method as a study deviation.

- - - 1. AE-related Withdrawal of Consent

Any AE-related withdrawal of consent during the study must be reported immediately (within 24 hours of identification) by email or fax to the sponsor's clinical trial monitor. The report should contain the relevant CRFs. Report the withdrawal to local IRB and the USAMRMC ORP in accordance with IRB policy.

- - - 1. Pending Inspections/Issuance of Reports

The knowledge of any pending compliance inspection/visit by the FDA, Office for Human Research Protections (Department of Health and Human Services), or other government agency concerning clinical investigation or research, the issuance of Inspection Reports, FDA Form 483, warning letters, or actions taken by any regulatory agency including legal or medical actions and any instances of serious or continuing noncompliance with the regulations or requirements will be reported immediately to the local IRB and the USAMRMC ORP and the sponsor's representative.

- - 1. IND Annual Reports and Final Clinical Study Reports
       1. IND Annual Report to the FDA

The PI will be responsible for the preparation of a detailed annual synopsis of clinical activity, including adverse events, for submission to the sponsor's representative (through ORA). Each annual report will summarize IND activity for 1 year. The sponsor's representative will notify the PI of the due date with sufficient time for the PI to assemble the required information.

- - - 1. Final Clinical Study Report

A final study report will be prepared in accordance with "Guidance for Industry: Submission of Abbreviated Reports and Synopses in Support of Marketing Applications", ICH E3 Guideline "Structure and Content of Clinical Study Reports", and electronic Common Technical Document (eCTD) standards and requirements and provided to the sponsor's representative (through ORA) for review and approval. The sponsor's representative will use this report to prepare the final clinical study report for submission to the FDA.

Statistics

This is a Phase 1 study. As such the sample size was not chosen to test a formal null hypothesis concerning safety, immunogenicity, or protective efficacy. However, the study will provide preliminary indications of these outcomes. Comparisons may be made between the heterologous vaccine regimens, but the sample is not designed to have good power for detecting statistically significant differences between vaccine groups.

Detailed statistical procedures, listings, table shells and figures will be provided in a separate statistical analysis plan (SAP) written shortly after protocol approval but before any subject enrollment. The SAP will be finalized before study close-out and database lock.

- 1. Description of Statistical Methods
     1. Statistical Tests

Descriptive statistics (percentage of study subjects, rate/immunization) will be used to characterize the occurrence of local and systemic adverse events in immunized subjects. Measurements with normal distributions expressed as means of continuous data (eg, magnitude of responses) will be assessed using the Student's t test (2-tailed), paired if pre-immunization values are compared with post immunization values, and unpaired if comparisons are made between groups. For discrete variables with normal distributions (eg number of responders, the number of positive assays, the number of individuals protected against challenge), the chi-squared test or Fisher's exact test will be used (two-tailed, uncorrected for chi-squared), except when the cell value is 5 or less, in which case only Fisher's exact test will be used (2-tailed). For days to parasitemia, subjects will be rank-ordered and a non-parametric test suitable for unpaired groups (eg, Mann Whitney) will be employed (1-tailed if comparing a vaccine group to controls, two-tailed if comparing 1 vaccinated group to another vaccinated group). In addition, days to parasitemia will be assessed using a Cox Proportional Hazards model and will be displayed using Kaplan-Meier plots.

Because of the relatively small sample size of this Phase 1 study, statistical power to formally compare the efficacy between the 2 vaccinated groups will be limited. For this reason, the primary efficacy comparisons will be made between each vaccine group and the infectivity controls. Nevertheless, the results of the trial should provide information regarding the relative protective efficacy of the 2 vaccine regimens and guide future development decisions.

- - 1. Safety Analyses

Safety analysis will include data collected from all immunized subjects. Adverse event data will be listed individually (including intensity and relatedness to investigational product), summarized by body system and preferred terms within a body system for each vaccine group, and categorized as local or systemic and as solicited or unsolicited AEs. Serious and/or unexpected AEs will also be discussed on a case-by-case basis. For the tabulation of AEs only the highest intensity of a specific AE will be recorded.

All adverse experiences will be described on the source records/eCRFs using standard medical terminology. The PI or clinical investigator will evaluate all adverse experiences as to their severity and relationship to the immunizations or challenge, and will report outcome and action taken, if any.

To analyze safety and tolerability, the overall percentage of subjects with at least 1 solicited local and/or systemic adverse event over a 7-day follow-up period will be tabulated. Similarly, the overall percentage of subjects with at least 1 unsolicited local and/or systemic adverse event during the 28-day follow-up period after immunization will be tabulated. In addition, the incidence, intensity, and relationship of individual solicited or unsolicited symptoms over the specified follow-up period will be calculated per group. The intensity and relationship to immunization of the unsolicited symptoms reported will also be assessed.

Adverse events already documented in the source records (ie, at a previous assessment) and designated as "ongoing" will be reviewed at subsequent visits. If these ongoing events have resolved, the documentation in the source records/eCRFs will be completed. If an adverse event increases in frequency or intensity during a study period, a new record of the event will be started.

Rates of all adverse events will be analyzed by Pearson's Chi-square test (or Fisher's exact test if assumptions are not met for Pearson's Chi-square) to compare groups. Summary tables will be created which will indicate the number of subjects who experienced events. Adverse events will be tabulated by study group. In addition, tables will be prepared to list each adverse event, the number of subjects in each vaccine group who experienced an event at least once, and the rate of subjects with adverse event(s). Adverse events will be divided into defined severity grades as described in Section 11.2.7. The tables will also divide the adverse events by severity and relationship to the investigational product. All subjects who receive at least 1 dose of the investigational vaccine will be included in the safety analysis.

- - 1. Efficacy Analyses

If immunized subjects are completely protected against malaria infection, protective efficacy will be calculated as the number of malaria positive subjects divided by the number challenged in the experimental group, relative to the control group:

| Efficacy = [1- | (# positive in experimental group X)/(# challenged in experimental group X) | ] x 100 |
| --- | --- | --- |
|  | (# positive in control group)/(# challenged in control group) |  |

If subjects are not completely protected against malaria infection, immunized and control subjects will be ranked according to time of onset of parasitemia and a non-parametric rank-order statistical test performed to look for delays in parasitemia induced by vaccination.

- - 1. Clinical Laboratory Data Analyses

The primary analysis will also include clinical data and laboratory data regarding safety, which includes vital signs and safety laboratory measures. Changes in pulse rate, systolic and diastolic blood pressure, and weight will be compared within each group and among groups using analysis of variance procedures. For hematology and serum chemistry tests, the mean, mean change, median, median change, and range of all values for each test for each group at baseline and for the final values will be tabulated.
Abnormal hematology and serum chemistry laboratory results will be tabulated by subject and by specific laboratory parameters. This will include day of onset, day or resolution and intensity. These tables will be reviewed by the PI to evaluate whether any significant trends in laboratory values occurred.

- - 1. Analysis of Immune Responses

For immune responses, both qualitative and quantitative (log10 transformed values) results will be analyzed. Geometric mean titers will be calculated along with 95% confidence intervals or standard deviation. Between groups comparisons will be examined with nonparametric tests (Kruskal-Wallis for continuous data and Fisher's exact test for categorical data) unless assumptions are fulfilled for Student's t test. Only subjects who receive at least 2 doses of the investigational vaccine will be included in the immunology analysis. All statistical tests will be interpreted in a 2-tailed fashion using an alpha = 0.05.

- - 1. Assessment of PSM Data Collection

For PSM data collection, the total number of hours, per day, for each subject will be calculated for each day in the study. A compliance rate will be determined for each subject based on the percentage of days in the study where the subject collected at least 12 hours of PSM data. Additionally, usage patterns will be assessed such as the average time-window that PSM data was collected.

- 1. Sample Size and Power Calculations

This section provides an overview of the power calculations for the clinical trial. The overall power calculation is based on 20 research subjects in each vaccine group and 12 infectivity controls. The following calculations were made:

1. The primary outcome variable for this study is safety. Therefore, the study design shall provide data to illustrate that the frequency of a serious or severe vaccine-related adverse event falls below a particular threshold, thereby supporting the safety of the candidate vaccine and its advancement in a phase 2 study, which will evaluate the experimental product in a larger number of human subjects. In this study, 40 subjects will be administered a specific investigational prime/boost vaccine regimen in one of two 20‑subject vaccine groups. If none of the subjects experience severe or serious vaccine-related adverse events, there is a 95% level of confidence that the true rate of these events in the general population would be 15% or less. The Upper Bound Calculation for No Events based on The Rule of Threes was used to determine this estimate of the potential risk of a study product related serious adverse event. The Rule of Threes states that given no observed events in “n” trials, the 95% upper bound on the rate of occurrence is 3/n ([van Belle G-2008](#vanBelle_2008)).
2. The power to assess vaccine efficacy (VE) in the immunized group versus the control group can be calculated using Fisher’s exact test: If 80% of the infectivity controls become positive, the study provides 49%, 74%, and > 90% power to detect vaccine efficacies of 50%, 60%, and 70%, respectively, comparing each vaccine group to controls. Power is higher if 100% of infectivity controls become positive. The statistical power of a trial to test the null hypothesis that VE = 0% versus various alternative hypotheses with VE > 0%, assuming a proportional hazards model and 12 evenly spaced visit time-points at which patent parasitemia can be detected.
3. The power to compare vaccine efficacy head-to-head: Fisher’s exact test is used to calculate the power comparing the infection rates between the 2 vaccinated groups. The power calculations are based on a sample size of 20 per group and a 2-sided 0.05 level test. Table 17 illustrates the power calculations for various values of the anticipated vaccine efficacies with the assumption of a 10% drop-out rate among subjects.

Table 17: Power for Comparing Vaccine Efficacies between Groups 1 and 2

| Anticipated Vaccine Efficacies | | Power |
| --- | --- | --- |
| Vaccine Group 1  D/ChAd63-CA | Vaccine Group 2  D/ChAd63-CAT |  |
| 20% | 40% | 25% |
| 20% | 50% | 47% |
| 20% | 60% | 70% |
| 20% | 80% | >95% |

For a 2-fold difference between the lower efficacy group of 0.2 (20%) and the higher efficacy group of 0.4 (40%), the power to assess the difference in protective efficacies between the 2 vaccine regimens is 25%. For a 2.5-fold difference in vaccine efficacies between the 2 groups, the power rises to 47%. The power rises to 70% for a 3-fold difference in vaccine efficacies between the groups. The power exceeds 95% when the difference between the lower vaccine efficacy group and the higher efficacy group is 4-fold.

In summary, these assessments indicate that with a sample size of 12 in the infectivity control group and 20 in each vaccine group, the study is adequately powered to assess efficacy of any vaccine group vs the control group (power calculation 1). The power to distinguish 2 vaccine regimens is less, unless there is at least a 3-fold difference in VE (power calculation 2). Despite the modest power to compare vaccine regimens directly, logistical considerations and cost prohibit larger sample sizes than those planned.

- 1. Statistical Criteria for the Termination of the Trial

There are no statistical criteria for study termination of this trial.

- 1. Accounting for Missing, Unused, and Spurious Data

Safety data from all immunized subjects will be included in analysis. Nonanalyzable data will be documented in the deviations.

- 1. Procedures for Reporting Deviations from the Original Statistical Plan

Any deviation(s) from the original statistical plan as indicated in the protocol will be described in an amendment to the protocol and the SAP. Deviations from the SAP will be documented in accordance with relevant SOPs.

- 1. Selection of Subjects to be Included in Analyses

Data collected from all immunized subjects will be analyzed for safety. Efficacy (protection or lack thereof) will include those subjects completing all immunization sessions and the challenge per protocol (per protocol analysis).

Direct Access to Source Data/Documents

Subjects will be identified by a unique subject identification number. No personal identifier will be used in any publication or communication used to support this research study. The subject identification number will be used if it becomes necessary to identify data specific to a single subject. Representatives of USAMRMC, the sponsor's representative, the local IRB and the USAMRMC ORP, and the FDA are eligible to review medical and research records related to this study as a part of their responsibility to protect human subjects in clinical research. Personal identifiers will be removed from photocopied medical and research records.

- 1. Study Monitoring

Study monitoring will be the responsibility of the USAMRMC Office of Regulated Activities. Upon successful approval of the protocol and establishment of the regulatory file, the clinical monitor will establish a clinical monitoring plan. To ensure that the investigator and the study staff understand and accept their defined responsibilities, the clinical monitor will maintain regular correspondence with the site and may be present during the course of the study to verify the acceptability of the facilities, compliance with the investigational plan and relevant regulations, and the maintenance of complete records. As needed, the clinical monitor may witness the informed consent process or other applicable study procedures to assure the safety of subjects and the investigators' compliance with the protocol and GCPs.

Monitoring visits by a sponsor's representative-designated clinical monitor will be scheduled to take place at the initiation of the study, during the study at appropriate intervals, and after the last subject has completed the study. A report of monitoring observations will be provided to the PI (for corrective actions), USAMRMC Office of Regulated Activities, and the product manager.

- 1. Audits and Inspections

Authorized representatives of the sponsor, the FDA, the independent ethics committee or NMRC IRB may visit the site to perform audits or inspections, including source data verification. The purpose of the audit or inspection is to systematically and independently examine all study-related activities and documents to determine whether these activities were conducted, and data were recorded, analyzed, and accurately reported according to the protocol, GCP guideline of the ICH, and any applicable regulatory requirements.

The investigator should contact the sponsor's representative and ORP HRPO immediately if contacted by a regulatory agency about an inspection.

- 1. Institutional Review Board

The NMRC IRB will serve as the responsible IRB and will review the protocol, informed consent, and progress reports on a continuing basis in accordance with all applicable regulations, including Title 21, Code of Federal Regulations (CFR), Parts 50 and 56. The PI must obtain IRB approval for the study. All documents approved by the NMRC IRB for this study, including the subject consent form and recruitment materials, must be maintained by the investigator and made available for inspection. The USAMRMC ORP HRPO will also provide oversight. The protocol will also be submitted to the WRAIR Human Subjects Protection Branch.

The PI will be responsible for preparing and submitting continuing review reports per institution and NMRC IRB requirements. The PI or a designee will submit the approved continuing review reports and the NMRC IRB approval notifications to HRPO as soon as the documents are available.

The PI or a designee will transmit the approved final study report and the NMRC IRB approval notification to the USAMRMC ORP HRPO as soon as the documents are available.

Quality Management Systems

Quality Management Systems (QMS) is an overall system of oversight utilized at the NMRC CTC to document and track site performance. The QMS activities facilitate planning for effective protocol implementation, ensure compliance with regulation, identify areas in need of corrective action, and promote constant state of readiness for an external audit or clinical monitoring visit. The QMS includes Quality Assurance (QA) and Quality Control (QC). The focus is to provide site staff with the means to proactively identify and resolve problems with protocol implementation and regulatory compliance, in the early stages.

- 1. Policies, Processes, Procedures, and Forms

SOPs for work practices are written as clear step by step instructions to ensure consistency in procedure performance. Clinical trial SOPs are authored by staff who are the subject matter experts for that particular procedure. Staff training requirements on SOPs are job-specific.

The QA department maintains updated and pertinent policies and procedures for the relevant institutional review boards.

- 1. Document Control

Document control is centralized to simplify and ensure version control and helps to ensure that the most current version of each document is used.

- 1. Personnel and Training

Each new CTC employee is required to perform initial SOP training with written examinations. Failure of an examination requires the employee to re-take the written exam until he/she is able to pass. SOP training is completed by each employee annually after that. Training on Good Clinical Practice is mandatory. General training is also required for each employee, which includes CITI (Collaborative Institutional Training Initiative), Henry M Jackson, FEMA and Department of Defense sponsored online and class room training. All medical doctors, nurses, and most staff are Basic Life Support (BLS) certified. All medical doctors are Advanced Cardiac Life Support (ACLS) certified.

- 1. Quality Assurance and Quality Control

As a general rule, a full internal QC audit is performed for each subject. These internal audits include reviewing each subject's informed consent, inclusion and exclusion criteria, demographics, safety screening laboratories, immunizations, CHMI, and scheduled and unscheduled visits. For cause audits are done based on complaints from study coordinators, investigators, sponsors, regulating agencies or subjects, or when subjects in the study experience serious adverse events.

- 1. Equipment and Facility

The CTC maintains a state of the art facility and equipment to accommodate complex and routine procedures for the conduct of a variety of clinical protocols. The CTC is capable of apheresis procedures, blood collection (for safety and immunological studies), vaccine (investigational product) preparation using a biosafety cabinet, and storing samples and investigational product at either refrigerated or ultra-low temperatures. Installation, operation, and performance evaluations of refrigerator and ultra-low temperature freezer were performed as part of the equipment qualifications.

CTC conducts vendor verification of its sample and vaccine storage, shipping, and receiving to ensure consistent delivery of quality services.

Currently, the CTC is supported by 3 emergency outlets (supplied by generator) located in 3 different rooms. These ensure continued function of critical storage equipment in case of electrical power failure.

- 1. Computer System Validation

The Food and Drug Administration (FDA) has indicated that the system user is responsible to assure that a system has been installed properly, is operating as designed, and performs as required in the user environment. Further, the FDA has indicated that there should be plans for these efforts, reflecting pre-determined testing, test data entry, and acceptable test results. The CTC, in compliance with the FDA regulation, conducts installation qualification (IQ), operation qualification (OQ), performance qualification (PQ), and user acceptance testing (UAT).

Ethics

- 1. Ethics Review

The study is based on adequately performed laboratory and animal experimentation; the study will be conducted under a protocol reviewed and approved by the NMRC IRB, WRAIR Division of Human Subjects Protection Branch (HSPB), and other applicable IRBs. The study is to be conducted by scientifically and medically qualified persons; the benefits of the study are in proportion to the risks; the rights and welfare of the subjects will be respected; the physicians conducting the study will ensure that the hazards do not outweigh the potential benefits; the results to be reported will be accurate; subjects will give their informed consent and will be competent to do so and not under duress; and all study staff will comply with the ethical principles in 21 CFR Part 50 and the Belmont Principles.

- - 1. Review/Approval of Study Protocol

The study protocol and other required documents will be submitted to the following for review and/or approval, with the final review by the FDA:

- Sponsor's Representative Team (USAMRMC ORA)
- NMRC ORA and IRB
- WRAIR HSPB
- Commanding Officer, NMRC
- Office of Research Protections, Human Research Protection Office (ORP HRPO)
- Sponsor's Representative, acting for The Surgeon General (TSG), Department of the Army
- USAMRMC Commanding General, if applicable

Enrollment in this protocol may not begin until all approvals have been obtained and the formal authorization letter is received by the PI from the sponsor's representative.

- - 1. Protocol Modifications

Modifications to the protocol and supporting documents must be reviewed and approved prior to implementation. Any protocol amendment will be agreed upon and approved by the sponsor's representative prior to submission to NMRC IRB and the ORP and prior to implementation of said change or modification. Any modification that could potentially increase risk to subjects must be submitted to the FDA prior to implementation. The informed consent document must be revised to concur with any amendment as appropriate and must be reviewed and approved with the amendment.

Any subject already enrolled in the study will be informed about the revision and asked to sign the revised informed consent document if the modification directly affects the individual's participation in the study. A copy of the revised, signed, and dated informed consent document will be given to the subject. All original versions of the informed consent document will be retained in the protocol regulatory file.

Any modification that could potentially increase risk to subjects must be submitted to the ORP HRPO, the FDA, and the NMRC IRB for approval prior to implementation. Documentation that NMRC IRB reviewed and approved the modifications also will be submitted. All other amendments will also be submitted to the ORP HRPO for inclusion in the HRPO study file. Additionally, the NMRC Commander approval authorization will be required for all protocol modifications and protocol amendments.

- - 1. Protocol Deviation Procedures

All subject-specific deviations from the protocol (eg, failure to return for follow-up visits or blood collection within the time indicated in the protocol) are to be documented. The PI or designee will be responsible for identifying and reporting all deviations, which are defined as isolated occurrences involving a procedure that did not follow the study protocol or study-specific procedure. Deviations will be reported annually in the continuing review report to the NMRC IRB and the ORP and, if appropriate, in the final study report. Action taken in response to the deviation, and the impact of the deviation will be assessed by the PI or clinical investigators and recorded as significant or nonsignificant.

Any protocol deviation that adversely affects the safety or rights of a subject or scientific integrity of the study, the deviation will be reported immediately to the sponsor's representative, NMRC IRB and the ORP.

- 1. Ethical Conduct of the Study

This study will be conducted in accordance with all applicable Federal and DoD human research protections requirements and the Belmont Principles of respect for persons, beneficence, and justice.

The procedures set out in this study are designed to ensure that the sponsor's representative and all study personnel abide by the principles of the ICH GCP Guideline and the CFR. The PI confirms this by signing this study protocol and FDA Form 1572.

- - 1. Confidentiality

HIPAA requires that researchers obtain the subject's permission (HIPAA Authorization) to use and disclose health information about the subject that is either created by or used in connection with this research. The information includes the entire research record and supporting information from the subject's medical records, results of laboratory tests, and both clinical and research observations made during the individual's participation in the research.

In this research, the subject's health information will be collected and used to conduct the study; to monitor the subject's health status; to measure effects of the investigational product; to determine research results, and possibly to develop new tests, procedures, and commercial products. Health information is used to report results of research to the sponsor's representative and Federal regulators and may be reviewed during study audits for compliance with study plans, regulations, and research policies. After the study ends, each subject has the right to see and receive a copy of his/her information.

Representatives of TSG as the IND sponsor, USAMRMC ORA as the sponsor's representative, the NMRC IRB, the MRMC ORP HRPO, the DoD, and the FDA are permitted to photocopy and review records related to this protocol and witness applicable study procedures as a part of their responsibility to protect the subjects of this protocol.

No personal identifier will be used in any publication or communication used to support this research study. The subject's identification number will be used in the event it becomes necessary to identify data specific to a single subject.

- - 1. Compensation for Participation

The following is a list of compensation amounts per type of study visit for civilian subjects:

- For completion of the screening visit each subject will receive $50.
- For study visits not involving an immunization or CHMI, civilian subjects will receive $100 per visit.
- For unscheduled visits each subject will receive $50.
- For immunization, each subject will receive $250 per immunization.
- For CHMI, each subject will receive $275.
- For the post-CHMI follow-up (hotel phase), each subject who is compliant with all of the required hotel phase daily visits, including all follow up visits after initiation of treatment for malaria, will receive $100 per day for 11 overnight stays for a total of $1,100. Each subject will receive $1,100 for the post-CHMI period, whether or not they are diagnosed with malaria.
- For PSM data collection, infectivity control subjects who opt-in will receive a $100 initial incentive when they first receive the PSM devices and training. Additionally, each subject will receive $50 for each visit to the Clinical Trial Center to upload the data from their smartphone (up to 6 visits). Subjects will receive $100 completion incentive upon return of the devices.
- For completion of all study visits subjects will be compensated an additional $250.
- A subject who is enrolled in a vaccine group and attends all visits, may receive a maximum total amount of compensation of $4,875.00.
- A subject who is enrolled as an infectivity control subject and undergoes CHMI, participates in PSM data collection, and attends all visits may receive a maximum total compensation amount of $3,010.00. Infectivity Control subjects who do not participate in the PSM data collection and attend all visits may receive a maximum total compensation amount of $2,475.00.

For active duty military subjects, compensation is limited to $50 per visit with a blood draw. However, if the visit with blood draw occurs during off-duty hours or while on leave, the compensation will be the same as that for non-military personnel.

Compensation will be provided for each study visit only when a subject completes all of the required study procedures designated for that specific study visit, as shown in the study event schedule. Subjects who serve as alternates but do not receive an immunization or who do not undergo CHMI will be paid $50 for successful completion of the screening visit and $100 for the day they serve as an alternate.

- - 1. Medical Care for Research-Related Injury

All non-exempt research involving human subjects shall, at a minimum, meet the requirement of 32 CFR 219.116(a)(6).

If a subject is injured because of participation in this research and is a DoD healthcare beneficiary (eg, active duty in the military, military spouse or dependent), the subject is entitled to medical care for that injury within the DoD healthcare system, as long as the subject remains a DoD healthcare beneficiary. This care includes, but is not limited to, free medical care at Army hospitals or clinics.

If a subject is injured because of participation in this research and is not a DoD healthcare beneficiary, the subject entitled to medical care for that injury at an Army hospital or clinic; medical care charges for care at an Army hospital or clinic will be waived. The subject is also entitled to care for that injury, but such care for that injury at other DoD (non-Army) hospitals or clinics may be limited by time, and the subject's insurance may be billed. It cannot be determined in advance which Army or DoD hospital or clinic will provide care. If the subject obtains care for research-related injuries outside of an Army or DoD hospital or clinic, the subject or the subject's insurance will be responsible for medical expenses.

- 1. Written Informed Consent

The informed consent process and document (Appendix A and Appendix E) will be reviewed and approved by the IRB(s), ORP HRPO, and sponsor's representative prior to initiation of the study. The ICD contains a full explanation of the possible risks, advantages, and alternate treatment options, and availability of treatment in the case of injury, in accordance with 21 CFR 50. The consent document indicates that by signature, the subject permits witnessing of applicable study procedures by the sponsor's representative, as well as access to relevant medical records by the sponsor's representative and by representatives of the FDA. The sponsor's representative will submit a copy of the initial IRB- and sponsor's representative-approved consent form to the FDA and will maintain copies of revised consent documents that have been reviewed and approved by the NMRC IRB and the ORP.

An informed consent document (in compliance with 21 CFR Part 50, 32 CFR Part 219, and the Belmont Principles) and HIPAA Authorization will be signed by the subject before any study-related procedures are initiated for that subject. This consent document must be retained by the investigator as part of the study records. Each subject will receive a copy of the signed informed consent document.

As part of the informed consent process, investigators or their designees will present the protocol in lay terms to subjects. Questions on the purpose of the protocol, protocol procedures, and risks to the subjects will then be solicited. Any question that cannot be answered will be referred to the PI. No subject should grant consent until questions have been answered to his/her satisfaction. The subject should understand that the study product is investigational and is not licensed by the FDA for commercial use, but is permitted to be used in this clinical research. Informed consent includes the principle that it is critical the subject be informed about the principal potential risks and benefits. This information will allow the subject to make a personal risk versus benefit decision and understand the following:

- Participation is entirely voluntary
- Subjects may withdraw from participation at any time
- Refusal to participate involves no penalty
- The individual is free to ask any questions that will allow him/her to understand the nature of the protocol
- A description of this clinical trial will be available on http://www.ClinicalTrials.gov, as required by US law

Subjects will also be asked to provide permission for the future use of their specimens. Refusal to allow samples to be stored for future use will not exclude subjects from participation.

Should the protocol be modified, the subject consent document must be revised to reflect the changes to the protocol. If a previously enrolled subject is directly affected by the change, the subject will receive a copy of the revised informed consent document. The approved revision will be read, signed, and dated by the subject.

Data Handling and Recordkeeping

The primary source document for this study will be the subject's medical record. If separate research records are maintained by the investigator(s), the medical record and the research records will be considered the source documents for the purposes of auditing the study. All source documents and subject study files will be retained at the site and stored in a locked records room with key access only given to NMRC CTC key personnel.

For this study, an EDC database system will be used for the collection of the study data in an electronic format. The EDC database system will be designed based on the protocol requirements, the approved eCRF layouts and specifications, and in accordance with 21 CFR Part 11. The eCRF layouts and specifications define and identify the applicable source data that will be collected and captured into the EDC database system. The applicable source data will be electronically entered by the study site designee onto the eCRF (data entry screens) in the EDC database system. The investigator is ultimately responsible for the accuracy of the data transcribed on the eCRF. Data monitoring and management will be performed in the EDC database system by the study clinical monitor and the designated data management group.

A detailed data management plan will be written and approved by the designated data management group and the Sponsor’s oversight data management designee and the PI prior to study start. All updates to the data management plan will be approved before study close-out and database lock.

- 1. Inspection of Records

The sponsor's representative or designee will be allowed to conduct site visits at the investigation facilities for the purpose of monitoring any aspect of the study. The investigator agrees to allow the monitor to inspect the drug storage area, investigational product stocks, drug accountability records, subject charts, study source documents, and other records relative to study conduct.

Subjects' health information is used to report results of research to the sponsor's representative and Federal regulators and may be reviewed during study audits for compliance with study plans, regulations, and research policies. The consent document indicates that by signature, the subject permits access to relevant medical records by the sponsor's representative and by representatives of the FDA as well as by the involved IRBs.

Upon a subject's termination from the trial, completed source records/eCRFs will be ready and available for on-site review by the sponsor's representative or the designated representative within 14 days after receipt of the subject's data.

- 1. Retention of Records

The PI must maintain all documentation relating to the study for a period of 2 years after the last marketing application approval, or if not approved for 2 years following the discontinuance of the investigational product for investigation. If it becomes necessary for the sponsor's representative or designee or the FDA to review any documentation relating to the study, the investigator must permit access to such records.

Completed, monitored source records/eCRFs will be stored in a secure location by the sponsor's representative or designee. A copy of each completed source records/eCRFs will be retained by the investigator.

The PI will be responsible for retaining sufficient information about each subject (ie, name, address, telephone number, Social Security number, and subject identifier in the study), so that the sponsor's representative, the local IRB, the FDA, employees of USAMRMC, or other regulatory authorities, and authorized USAID representatives may have access to this information should the need arise.

It is the policy of the USAMRMC that data sheets are to be completed for all subjects participating in research (Form 60-R, Volunteer Registry Data Sheet). The data sheets will be entered into this Command's Volunteer Registry Database. The information to be entered into this confidential data base includes the subject's name, address, and Social Security Number; study title; and dates of participation. The intent of this data base is two-fold: first, to readily answer questions concerning an individual's participation in research sponsored by USAMRMC; and second, to ensure that USAMRMC can exercise its obligation to ensure research subjects are adequately warned (duty to warn) of risks and to provide new information as it becomes available. The information will be stored at USAMRMC for a minimum of 75 years. The Volunteer Registry Database is a separate entity and is not linked to the study database.

Publication Policy

All data collected during this study will be used to support this IND. All data may be published in the open medical or military literature with the identity of the subjects protected. Anyone desiring to publish or present data obtained during the conduct of the study will conform to NMRC Command policies and publication review clearance procedures. Publication must also be forwarded for review to the Commander, United States Army Medical Research and Materiel Command (USAMRMC) or designee and Office of Regulated Activities at usarmy.detrick.medcom-usamrmc.mbx.regulatory-affairs@mail.mil prior to submission.

1. List of References

Armed Forces Health Surveillance Center. Case Report: Fatal outcome of *falciparum* malaria acquired in Liberia, U.S. Navy member. Medical Surveillance Monthly Report. 2010:17(1).

Beadle C, Hoffman SL. History of malaria in the United States Naval Forces at war: World War I through the Vietnam conflict. Clin Infect Dis. 1993;16(2):320-9.

Breman JG, Alilio MS, Mills A. Conquering the intolerable burden of malaria: what's new, what's needed: a summary. Am J Trop Med Hyg. 2004;71(2 Suppl):1-15.

Buchbinder SP, Mehrotra DV, Duerr A, Fitzgerald DW, Mogg R, Li D, et al. Efficacy assessment of a cell-mediated immunity HIV-1 vaccine (The Step Study): a double-blind, randomized, placebo-controlled, test-of-concept trial. Lancet. 2008;372(9653):1881-93.

Catanzaro AT, Roederer M, Koup RA, et al., Phase 1 clinical evaluation of a six-plasmid multiclade HIV-1 DNA candidate vaccine. Vaccine. 2007; 25(20): 4085-92.

Centers for Disease Control. Malaria acquired in Haiti-2010. MMWR Morb Mortal Wkly Rep. 2010:59(8):217-9.

Chuang I, Sedegah M, Cicatelli, S, Spring M, Polhemus M, Tamminga C, et al. DNA prime/adenovirus boost malaria vaccine encoding *P. falciparum* CSP and AMA1 induces sterile protection associated with cell-mediated immunity. PLoS ONE. 2013;8(2): e55571. doi:10.1371/journal.pone.0055571.

Church LW, Le TP, Bryan JP, Gordon DM, Edelman R, Fries L, et al. Clinical manifestations of *Plasmodium falciparum* malaria experimentally induced by mosquito challenge. J Inf Dis. 1997;175:915-20.

de Barra E, Hodgson SH, Ewer KJ, et al., A Phase 1a Study to Assess the Safety and Immunogenicity of New Malari Vaccine Candidates ChAd63 CS Administered Alone and with MVA CA. PLoS ONE. 2014; 9(12):e115161.

Dunachie SJ, Walther M, Epstein JE, et al. A DNA prime-modified vaccinia virus ankara boost vaccine encoding thromboplastin-related adhesion protein but not circumsporozoite protein partially protects healthy malaria-naïve adults against Plasmodium falciparum sporozoite challenge. Infect Immun. 2006; 74(10):5933-42.

Duncan CJA, Sheehy SH, Ewer KJ, Douglas AD, Collins KA, Halstead FD, et al. Impact on malaria parasite multiplication rates in infected volunteers of the protein-in-adjuvant vaccine AMA1-C1/Alhydrogel+CPG 7909. PLoS One. 2011;6(7):e22271.

Duerr A, Huang Y, Buchbinder S, Coombs RW, Sanchez J, del Rio C, Casapia M, et al. Extended follow-up confirms early vaccine-enhanced risk of HIV acquisition and demonstrates waning effect over time among participants in a randomized trial of recombinant adenovirus HIV vaccine (Step Study). J Infect Dis. 2012:206(2):258-66.

Duerr A, Huang Y, Buchbinder S, Coombs RW, Sanchez J, del Rio C, et al. Reply to Richie and Villasante. J Infect Dis. 2013:207(4):690-2.

Epstein JE, Gorak EJ, Charoenvit Y, et al. Safety, Tolerability and lack of antibody responses after administration of a PfCSP DNA malaria vaccine via needle or needle-free jet injection, and comparison of intramuscular and combination intramuscular/intradermal routes. Hu Gene Ther. 2002 Sep 1; 13 (13): 1551-60.

Epstein JE, Rao S, Williams F, Freilich D, Luke T, Sedegah M, et al. Safety and clinical outcome of experimental challenge of human volunteers with *Plasmodium falciparum*-infected mosquitoes: an update. J Infect Dis. 2007;196(1):145-54.

Ewer, KJ, O’Hara GA, Duncan CJA, Collins KA, Sheehy SH, Reyes-Sandoval A, et al. Protective CD8+ T-cell immunity to human malaria induced by chimpanzee adenovirus-MVA immunization. Nat Commun. 2013; 4:2836.

Fauci AS, Marovich MA, Dieffenbach CW, Hunter E, Buchbinder SP. Immune Activation with HIV Vaccines. Science. 2014; 344; 49 -51.

Gallup JL, Sachs JD. The economic burden of malaria. Am J Trop Med Hyg. 2001;64(1-2 Suppl):85-96.

Gaziano TA, Young CR, Fitzmaurice G, Atwood S, Gaziano JM. Laboratory-based versus non-laboratory-based method for assessment of cardiovascular disease risk: the NHANES I Follow-up Study cohort. Lancet. 2008;371(9616):923-31.

Graham BS, Koup RA, Roederer M, et al. Phase 1 safety and immunogenicity evaluation of a multiclade HIV-1 DNA candidate vaccine. J Infect Dis 2006; 194(12): 1650-60.

Graham BS, Enama ME, Nason MC, Gordon IJ, Peel SA, Ledgerwood JE, Plummer SA, Mascola JR, Bailer RT, Roederer M, Koup RA, Nabel GJ. DNA Vaccine Delivered by a Needle-Free Injection Device Improves Potency of Priming for Antibody and CD8+ T-Cell Responses after rAd5 Boost in a Randomized Clinical Trial. PLoS One 2013;8(4):e59340.

Gray GE, Allen M, Moodie Z, et al. Safety and efficacy of the HVTN 503/Phambili Study of a clade-B-based HIV-1 vaccine in South Africa: a double-blind, randomised, placebo-controlled test-of-concept phase 2b study. Lancet Infect. Dis. 2011;11(7):507.

Gray GE, Moodie Z, Metch B, et al. Recombinant adenovirus type 5 HIV gag/pol/nef vaccine in South Africa: unblinded, long-term follow-up of the phase 2b HVTN 503/Phambili study Lancet Infect. Dis. 2014;14(5):388-97.

Hammer SM, Sobieszczyk ME, Janes H, et al. Efficacy Trial of a DNA/rAd5 HIV-1 Preventive Vaccine. N Engl J Med. 2013;369(22):2083-2092.

Hedstrom RC, Doolan DL, Wang R, Kumar A, Sacci JB Jr, Gardner MJ, et al. In vitro expression and in vivo immunogenicity of *Plasmodium falciparum* pre-erythrocytic stage DNA vaccines. Int J Mol Med. 1998 Jul;2(1):29-38.

Hill AV, Reyes-Sandoval A, O'Hara G, Ewer K, Lawrie A, Goodman A, et al. Prime-boost vectored malaria vaccines: progress and prospects. Hum Vaccin. 2010;6(1):78-83.

Hodgson SH, Ewer KJ, Bliss CM, Edwards NJ, Rampling T, Anagnostou NA, et al. Evaluation of the Efficacy of ChAd63-MVA Vectored Vaccines Expressing Circumsporozoite Protein and ME-TRAP Against Controlled Human Malaria Infection in Malaria-Naïve Individuals. J Infect Dis. 2014;PMID: 25336730.

Hodgson SH, Choudhary P, Elias SC, et al. Combining viral vectored and protein-in-adjuvant vaccines against the blood stage malaria antigen AMA1: report on a phase 1a clinical trial. Mol Ther. 2014; 22(12):2142-54. PMID: 25156127.

Hoffman SL, Goh LM, Luke TC, Schneider I, Le TP, Doolan DL, et al. Protection of humans against malaria by immunization with radiation-attenuated *Plasmodium falciparum* sporozoites. J Infect Dis. 2002;185(8):1155-64.

Laurens MB, Duncan CJ, Epstein JE, Hill AV, Komisar JL, Lyke KE, et al. A consultation on the optimization of controlled human malaria infection by mosquito bite for evaluation of candidate malaria vaccines. Vaccine. 2012;30(36):5302-4.

McElrath MJ, De Rosa SC, Moodie Z, et al. HIV-1 vaccine-induced immunity in the test-of-concept Step Study: a case-cohort analysis. Lancet. 2008;372(9652):1894-1905.

Moorthy VS, Diggs C, Ferro S, Good MF, Herrera S, Hill AV, et al. Report of a consultation on the optimization of clinical challenge trials for evaluation of candidate blood stage malaria vaccines, 18-19 March 2009, Bethesda, MD, USA. Vaccine. 2009 Sep 25;27(42):5719-25.

Miura K, Herrera R, Diouf A, Zhou H, Mu J, Hu Z, et al. Overcoming allelic specificity by immunization with five allelic forms of Plasmodium falciparum apical membrane antigen. Infect Immun. 2013;81(15):1491–501.

National Institute of Allergy and Infectious Diseases Mini-Summit on Adenovirus Platforms for HIV Vaccines, 19 September 2013 (webcast available at www.niaid.nih.gov/topics/HIVAIDS/Research/vaccines/Pages/adenovirusPlatforms.aspx).

Nieman AE, de Mast Q, Roestenberg M, Wiersma J, Pop G, Stalenhoef A, et al. Cardiac complication after experimental human malaria infection: a case report. Malaria J. 2009;8:277.

Ogwang C, Afolabi M, Kimani D, Jagne YJ, Sheehy SH, Bliss CM, et al. Safety and immunogenicity of heterologous prime-boost immunisation with *Plasmodium falciparum* malaria candidate vaccines, ChAd63 ME-TRAP and MVA ME-TRAP, in healthy Gambian and Kenyan adults. PLoS One. 2013;8(3):e57726.

Ogwang C, Kimani D, Edwards N, et al. Prime-boost vaccination with chimpanzee adenovirus and modified vaccinia Ankara encoding TRAP provides partial protection against *Plasmodium falciparum* infection in Kenyan adults. Sci Transl Med. 2015 May 6;7 (286):286re5, DOI: 10.1126/scitranslmed.aaa2373.

O'Hara GA, Duncan CJ, Ewer KJ, Collins KA, Elias SC, Halstead FD, et al. Clinical assessment of a recombinant simian adenovirus ChAd63: a potent new vaccine vector. J Infect Dis. 2012;205(5):772-81.

Patterson N, Ganeshan, H, Abot, EN, et al. Tolerability and immunogenicity of a *P. falciparum* multi-antigen multi-stage adenovirus vectored vaccine, Naval Medical Research Center-M3V-Ad-PfCA, in NZW Rabbits. Abstract #1050. The 55^th^ American Society of Tropical Medicine and Hygiene Meeting. Atlanta, GA, USA. 16 Nov 2006.

Patterson NB, Bruder JT, Limbach K, et al. Monovalent and bivalent adenovectored vaccines expressing the *Plasmodium falciparum* antigens AMA-1 and MSP1-42 (3D7) elicit functional antibodies in NZW rabbits. Abstract #48. The 56^th^ American Society of Tropical Medicine and Hygiene Meeting. Philadelphia, PA, USA. 05 Nov 2007.

Quinn KM, De Costa A, Yamamoto A, et al. Comparative Analysis of the Magnitude, Quality, Phenotype, and Protective Capacity of Simian Immunodeficiency Virus Gag-Specific CD8+T Cells following Human-, Simian-, and Chimpanzee-derived Recombinant Adenoviral Vector Immunization. J Immunology. 2013;190:2720-2735.

Richie TL, Charoenvit Y, Wang R, Epstein JE, Hedstrom RC, Kumar S, et al. Clinical trial In healthy malaria-naïve adults to evaluate the safety, tolerability, immunogenicity and efficacy of MuStDO5, a five-gene, sporozoite/hepatic stage *Plasmodium falciparum* DNA vaccine combined with escalating dose human GM-CSF DNA. Hum Vaccin Immunother. 2012;8(11):1564-84.

Rickman LS, Jones TR, Long GW, Paparello S, Schneider I, Paul CF, et al. Plasmodium falciparum infected *Anopheles stephensi* inconsistently transmit malaria to humans. Am J Trop Med Hyg. 1990;43(5):441-5.

Roestenberg M, O'Hara GA, Duncan CJ, Epstein JE, Edwards NJ, Scholzen A, et al. Comparison of clinical and parasitological data from controlled human malaria infection trials. PLoS One. 2012;7(6):e38434.

Sedegah M, Hollingdale MR, Farooq F, et al. Sterile immunity to malaria after DNA prime/adenovirus boost immunization is associated with effector memory CD8+T cells targeting AMA1 class I epitopes. PLoS One. 2014 Sep 11; 9(9):e106241.

Sedegah M, Tamminga C, McGrath S, House B, Ganeshan H, Lejano J, et al. Adenovirus 5-vectored P. falciparum vaccine expressing CSP and AMA1. Part A: safety and immunogenicity in seronegative adults. PLoS One. 2011;6(10): e24586.

Seder RA, Chang LJ, Enama ME, et al. Protection against malaria by intravenous immunization with a nonreplicating sporozoite vaccine. Science 2013; 341(6152):1359-65. doi: 10.1126/science.1241800.

Sheehy SH, Duncan CJ, Elias SC, Biswas S, Collins KA, O'Hara GA, et al. Phase Ia clinical evaluation of the safety and immunogenicity of the *Plasmodium falciparum* blood-stage antigen AMA1 in ChAd63 and MVA vaccine vectors. PLoS One. 2012a;7(2):e31208.

Sheehy SH, Duncan CJ, Elias SC, Choudhary P, Biswas S, Halstead FD, et al. ChAd63-MVAvectored blood-stage malaria vaccines targeting MSP1 and AMA1: assessment of efficacy against mosquito bite challenge in humans. Mol Ther. 2012b;20(12):2355-68.

Statistical Center for HIV/AIDS Research and Prevention (SCHARP). Statistical Calculator. Accessed: 05 Jan 2015. Available from: http://www.scharp.org/tools/RLDwebCalc/RLD.php.

Tamminga C, Sedegah M, Regis D, Chuang I, Epstein JE, Spring M, et al. Adenovirus-5-vectored *P. falciparum* vaccine expressing CSP and AMA1. Part B: safety, immunogenicity and protective efficacy of the CSP component. PLoS One. 2011;6(10):e25868.

Tamminga C, Sedegah M, Maiolatesi S, Fedders C, Reyes S, Reyes, A, Richie TL. Human adenovirus 5-vectored *Plasmodium falciparum* NMRC-M3V-Ad-PfCA vaccine encoding CSP and AMA1 is safe, well-tolerated and immunogenic but does not protect against controlled human malaria infection. Hum Vaccin Immunother. 2013;9(10), 2165-2177. doi: 10.4161/hv.24941.

Tatsis N, Tesema L, Robinson ER, Giles-Davis W, McCoy K, Gao GP, et al. Chimpanzee-origin adenovirus vectors as vaccine carriers. Gene Ther. 2006;13(5):421–9.

Tatsis N, Fitzgerald JC, Reyes-Sandoval A, Harris-McCoy KC, Hensley SE, Zhou D, et al. Adenoviral vectors persist in vivo and maintain activated CD8+ T cells: implications for their use as vaccines. Blood. 2007;110(6):1916-23.

United States Food and Drug Administration. Center for Biologics Evaluation and Research. Guidance for Industry Toxicity Grading Scale for Healthy Adult and Adolescent Volunteers Enrolled in Preventive Vaccine Clinical Trials. 2007. Accessed: 01 Jun 2015. Available from: http://www.fda.gov/cber/guidelines.htm.

van Belle G. Statistical Rules of Thumb, 2^nd^ edition, 2008, New York: John Wiley & Sons, Inc., p 49-50.

van Meer MPA, Bastiaens GJ, Boulaksil M, de Mast Q, Gunasekera A, Hoffman SL, et al. Idiopathic acute myocarditis during treatment for controlled human malaria infection: a case report. Malar J. 2014;13:38.

Verhage DF, Telgt DS, Bousema JT, Hermsen CC, van Gemert GJ, van der Meer JW, Sauerwein RW. Clinical outcome of experimental human malaria induced by *Plasmodium falciparum*-infected mosquitoes. Neth J Med. 2005;63(2):52-8.

Wang R, Epstein J, Baraceros FM, et al. Induction of CD4(+)T cell-dependent CD8(+) responses in humans by a malaria DNA vaccine. Proc Natl Acad Sci USA. 2001 Sep 11; 98(19):10817-22.

Wang R, Richie TL, Baraceros MF, Rahardjo N, Gay T, Banania JG, et al. Boosting of DNA vaccine-elicited gamma interferon responses in humans by exposure to malaria parasites. Infect Immun. 2005 May;73(5):2863-72.

Weiss WR, Kumar A, Jiang G, Williams J, Bostick A, Conteh S, Richie TL. Protection of rhesus monkeys by a DNA prime/poxvirus boost malaria vaccine depends on optimal DNA priming and inclusion of blood stage antigens. PLoS One.2007;2(10), e1063. doi: 10.1371/journal.pone.000106.

Whitman TJ, Coyne PE, Magill AJ, Blazes DL, Green MD, Milhous WK, et al. An outbreak of *Plasmodium falciparum* malaria in U.S. Marines deployed to Liberia. Am J Trop Med Hyg. 2010:83(2);258-65.

World Health Organization. World Malaria Report 2015 [cited 03 January 2016]. Available from: http://www.who.int/malaria/publications/world-malaria-report-2015/report/en/

1. Informed Consent Document (Immunized and Infectivity Controls)
2. Human Immunodeficiency Virus (HIV) Testing Consent
3. Assessment of Understanding (Immunized and Infectivity Controls)
4. Gaziano Cardiovascular Disease Risk Assessment Chart for Men and Women


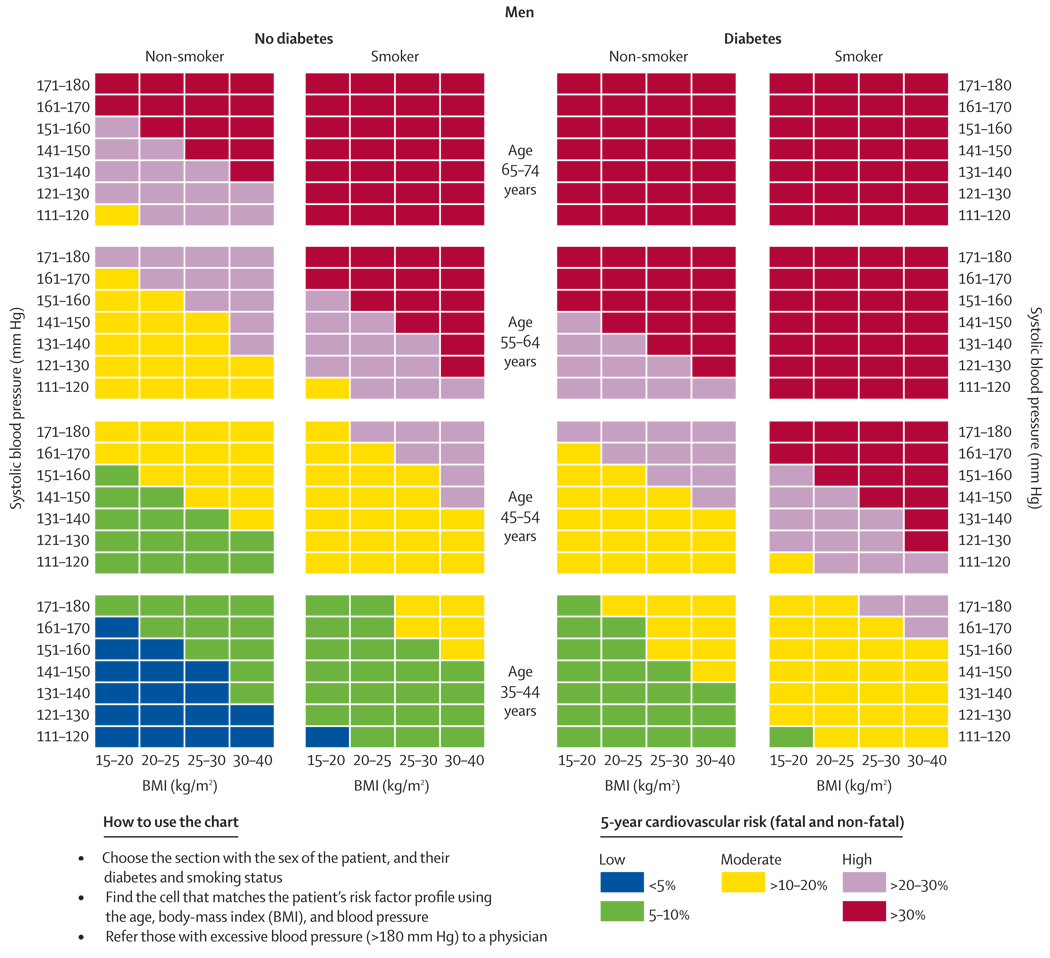


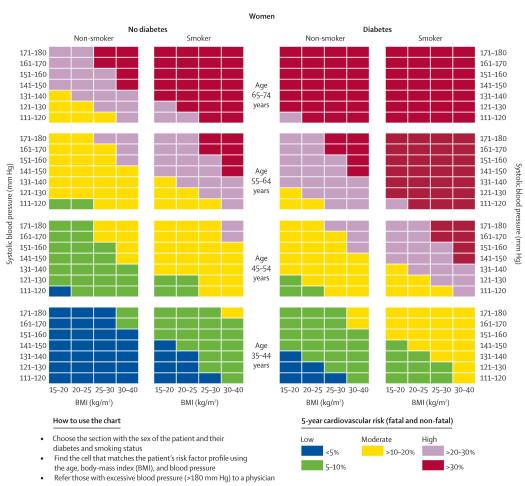


1. Informed Consent Document for Infectivity Controls, Additional Consent for Physiological Status Monitoring
2. Toxicity Grading Scales

Appendix E is based upon the FDA CBER guidelines entitled “Guidance for Industry Toxicity Grading Scale for Healthy Adult and Adolescent Volunteers Enrolled in Preventive Vaccine Clinical Trials” ([2007](#FDA_2007)) and Quest Diagnostics normal reference ranges and contains the following tables:

- Table 18: Grading Scale for Clinical Abnormalities (Local Reaction to Injectable Product)
- Table 19: Grading Scale for Clinical Abnormalities (Vital Signs)
- Table 20: Grading Scale for Clinical Abnormalities (Symptoms)
- Table 21: Grading Scale for Laboratory Abnormalities

Table 18: Grading Scale for Clinical Abnormalities (Local Reaction to Injectable Product)

| Local Reaction to Injectable Product | Mild (Grade 1) | Moderate (Grade 2) | Severe (Grade 3) | Potentially Life Threatening (Grade 4) |
| --- | --- | --- | --- | --- |
| Pain | Does not interfere with activity | Repeated use of non-narcotic pain reliever > 24 hours or interferes with activity | Any use of narcotic pain reliever or prevents daily activity | Emergency room (ER) visit or hospitalization |
| Tenderness | Mild discomfort to touch | Discomfort with movement | Significant discomfort at rest | ER visit or hospitalization |
| Erythema/Redness | 2.5 – 5.0 cm | 5.1 – 10.0 cm | > 10 cm | Necrosis or exfoliative dermatitis |
| Induration / Swelling | 2.5 – 5.0 cm and does not interfere with activity | 5.1 – 10.0 cm or interferes with activity | > 10 cm or prevents daily activity | Necrosis |

a In addition to grading the measured local reaction at the greatest single diameter, the measurement shall be recorded as a continuous variable

b Induration/Swelling shall be evaluated and graded using the functional scale as well as the actual measurement.

Table 19: Grading Scale for Clinical Abnormalities (Vital Signs)

| Vital Signs | Mild (Grade 1) | Moderate (Grade 2) | Severe (Grade 3) | Potentially Life Threatening (Grade 4) |
| --- | --- | --- | --- | --- |
| Fever (^o^C)  (^o^F) b | 38.0 - 38.4  100.4 – 101.1 | 38.5 – 38.9  101.2 – 102.0 | 39.0 – 40.0  102.1 – 104.0 | > 40.0  > 104.0 |
| Tachycardia – beats per minute | 101 – 115 | 116 – 130 | >130 | ER visit or hospitalization for arrhythmia |
| Bradycardia – beats per minute | 50 - 54 | 45 – 49 | < 45 | ER visit or hospitalization for arrhythmia |
| Hypertension (systolic) – mm Hg | 141 – 150 | 151 - 155 | >155 | ER visit or hospitalization for malignant hypertension |
| Hypertension (diastolic) – mm Hg | 91 – 95 | 96 – 100 | >100 | ER visit or hospitalization for malignant hypertension |
| Hypotension (systolic) – mm Hg | 85 – 89 | 80 – 84 | < 80 | ER visit or hospitalization for hypotensive shock |

a Subject should be at rest for all vital signs measurements.

b Oral temperature; no recent hot or cold beverages or smoking.

c When resting heart rate is between 60 – 100 beats per minute. Use clinical judgment when characterizing bradycardia among some healthy subject population, for example, conditioned athletes.

Table 20: Grading Scale for Clinical Abnormalities (Symptoms)

| Systemic Illness (General) | Mild (Grade 1) | Moderate (Grade 2) | Severe (Grade 3) | Potentially Life Threatening (Grade 4) |
| --- | --- | --- | --- | --- |
| Nausea/Vomiting | No interference with activity or 1 – 2 episodes/24 hours | Some interference with activity or > 2 episodes/24 hours | Prevents daily activity, requires outpatient IV hydration | ER visit or hospitalization for hypotensive shock |
| Diarrhea | 2 – 3 loose stools or < 400 g/24 hours | 4 – 5 stools or 400 – 800 g/24 hours | 6 or more watery stools or > 800 g per 24 hours or requires outpatient IV hydration | ER visit or hospitalization |
| Headache | No interference with activity | Repeated use of non-narcotic pain reliever > 24 hours or some interference with activity | Significant; any use of narcotic pain reliever or prevents daily activity | ER visit or hospitalization |
| Fatigue | No interference with activity | Some interference with activity | Significant; prevents daily activity | ER visit or hospitalization |
| Myalgia | No interference with activity | Some interference with activity | Significant; prevents daily activity | ER visit or hospitalization |
| Illness or clinical adverse event (as defined according to applicable regulations) | No interference with activity | Some interference with activity | Prevents daily activity and requires medical intervention | ER visit or hospitalization |

Table 21: Grading Scale for Laboratory Abnormalities

| Laboratory Test | Mild (Grade 1) | Moderate ( Grade 2) | Severe (Grade 3) | Potentially Life Threatening (Grade 4) |
| --- | --- | --- | --- | --- |
| Hemoglobin (female) – gm/dL | 11.0 – 12.0 | 9.5 – 10.9 | 8.0 – 9.4 | < 8.0 |
| Hemoglobin (female) change from baseline value – gm/dL | Any decrease – 1.5 | 1.6 – 2.0 | 2.1 – 5.0 | > 5.0 |
| Hemoglobin (male) – gm/dL | 12.5 – 13.5 | 10.5 – 12.4 | 8.5 – 10.4 | < 8.5 |
| Hemoglobin (male) change from baseline value – gm/dL | Any decrease – 1.5 | 1.6 – 2.0 | 2.1 – 5.0 | > 5.0 |
| Absolute neutrophil count (ANCcells/L)) | 1,500 – 2,000 | 1,000 – 1,499 | 500 - 999 | <500 |
| WBC Increase – cell/ mm^3^ | 10,800 – 15,000 | 15,001 – 20,000 | 20,001 – 25,000 | >25,000 |
| WBC Decrease – cell/mm^3^ | 2,500 – 3,500 | 1,500 – 2,499 | 1,000 – 1,499 | < 1,000 |
| Lymphocyte Decrease (cell/mm^3^) | 750 – 1,000 | 500 – 749 | 250 – 499 | < 250 |
| Neutrophils Decrease (cells/mm^3^) | 1,500 – 2,000 | 1,000 – 1,499 | 500 – 999 | < 500 |
| Eosinophils (cells/mm^3^) | 650 – 1,500 | 1,501 – 5,000 | >5,000 | Hypereosinophilic |
| Platelets decrease (cells/mm^3^) | 125,000 – 139,999 | 100,000 – 124,999 | 25,000 – 99,999 | < 25,000 |
| ALT (ULN as per Quest reference ranges) | 1.1 – 2.5 x ULN | 2.6 – 5.0 x ULN | 5.1 – 10 x ULN | > 10 x ULN |
| AST(ULN as per Quest reference ranges) | 1.1 – 2.5 x ULN | 2.6 – 5.0 x ULN | 5.1 – 10 x ULN | > 10 x ULN |
| Alkaline phosphatase (ULN as per Quest reference ranges) | 1.1 – 2.0 x ULN | 2.1 – 3.0 x ULN | 3.1 – 10 x ULN | > 10 x ULN |
| BUN (mg/dL) | 23 – 26 | 27 - 31 | >31 | Requires dialysis |
| Total bilirubin – when accompanied by any increase in Liver Function Test, increase by factor | 1.1 – 1.25 x ULN | 1.26 – 1.5 x ULN | 1.51 – 1.75 x ULN | > 1.75 x ULN |
| Total bilirubin – when Liver Function Test is normal; increase by factor | 1.10 – 1.50 x ULN | 1.59 – 2.00 x ULN | 2.01 – 3.00 x ULN | > 3.00 x ULN |
| Creatinine – mg/dL | 1.50 – 1.70 | 1.80 – 2.00 | 2.10 – 2.50 | > 2.50 or requires dialysis |
| Glucose (Hypoglycemia) mg/dL | 65 – 69 | 55 – 64 | 45 – 54 | < 45 |
| Glucose (Hyperglycemia) mg/dL (random) | 110 – 125 | 126 – 200 | >200 | Insulin requirement or hyperosmolar coma |
| Calcium – hypocalcemia (mg/dL) | 8.0 – 8.4 | 7.5 – 7.9 | 7.0 – 7.4 | < 7.0 |
| Calcium – hypercalcemia (mg/dL) | 10.5 – 11.0 | 11.1 – 11.5 | 11.6 – 12.0 | > 12.0 |
| Urine protein | Trace | 1 + | 2 + | Hospitalization for dialysis |
| Urine glucose | Trace | 1 + | 2 + | Hospitalization for hyperglycemia |
| Blood (microscopic) – red blood cells per high power field | 1-10 | 11-50 | >50 and/or gross blood | Hospitalization or packed red blood cells (PRBC) transfusion |
