# Supplementary material for: A three-antigen Plasmodium falciparum DNA prime—Adenovirus boost malaria vaccine regimen is superior to a two-antigen regimen and protects against controlled human malaria infection in healthy malaria-naïve adults
Source: PLoS One. 2021 Sep 8;16(9):e0256980. doi: 10.1371/journal.pone.0256980 (PMC8425539; doi:10.1371/journal.pone.0256980)
Supplement: S2 File — (DOCX) [file pone.0256980.s003.docx]

**Supplement**

**A Three-Antigen *Plasmodium falciparum* DNA Prime - Adenovirus Boost Malaria Vaccine Regimen is Superior to a Two-Antigen Regimen and Protects against Controlled Human Malaria Infection in Healthy Malaria-Naïve Adults**

**Sklar M. J., et al.**

**Supplementary Figure S1.** **Study Design**

CA group and CAT group subjects were immunized three times with DNA at weeks 0, 4 and 8, with ChAd63 at week 24, and CHMI was administered at week 28. The final clinic visit was week 41. (*) PBMC samples; (*) Serum samples.

**Supplementary Figure S2. Schematic of the DNA and ChAd63 CSP, AMA1 and TRAP vaccines**

Each panel presents the native protein (top of each panel) and the protein expressed by the DNA or ChAd63 construct (middle and bottom of each panel) for the CSP (Panel A), AMA1 (Panel B) and TRAP (Panel C) vaccine antigens. N = amino terminus; C = carboxy terminus; TPA = human tissue plasminogen activator signal sequence; TM = transmembrane domain.

Identical colors indicate identical sequences. *Represent amino acid substitutions of the original native sequence. The ChAd63 construct included two alleles of AMA1 – 3D7 and FVO – that differ in 34 amino acids.

**Supplementary Figure S3. Pre-existing Nab to HuAd5 do not interfere with protection**

Nab titers to HuAd5 were measured prior to ChAd63 immunization in the CA and CAT groups and compared with days to patency by microscopy after CHMI. Positive Nab >1:500 (horizontal red line). In the CA group, the single protected subject and 8 non-protected subjects were negative (below red line) and 4 non-protected subjects were positive (above red line). In the CAT group, two protected subjects and 8 non-protected subjects were negative (below red line) and three protected subjects, and two non-protected subjects were positive (above red line).

**Supplementary Table S1: Vaccine Products**

| **Name** | **Description** |
| --- | --- |
| **DNA Priming Products** | |
| D-C | DNA plasmid encoding the *Plasmodium falciparum* pre-erythrocytic stage antigen circumsporozoite protein (CSP, PfCSP or “C”). The DNA sequence for the CSP antigen is derived from the 3D7 strain of Pf containing a synthetic, codon optimized gene sequence, using plasmid VCL-2571, manufactured by Aldevron LLC (Fargo, ND), final filtered, and vialed by UIP (Iowa City, IA). |
| D-A | DNA plasmid encoding the *Plasmodium falciparum* pre-erythrocytic/ erythrocytic antigen apical membrane antigen-1 (AMA1, PfAMA1 or “A”). The DNA sequence for the AMA1 antigen is derived from the 3D7 strain of Pf containing a synthetic, codon optimized gene sequence, using plasmid VCL-2577 manufactured by Aldevron LLC (Fargo, ND), final filtered, and vialed by UIP (Iowa City, IA). |
| D-T | DNA plasmid encoding the *Plasmodium falciparum* pre-erythrocytic stage antigen thrombospondin-related adhesion protein/sporozoite surface protein-2 (TRAP/SSP2, PfTRAP/SSP2, TRAP, or “T”). The DNA sequence for the TRAP antigen is derived from the 3D7 strain of Pf containing a synthetic, codon optimized gene sequence^1^. Plasmid VCL-2576 was manufactured by Aldevron LLC (Fargo, ND), final filtered, and vialed by UIP (Iowa City, IA). |
| Phosphate Buffered Saline (DNA diluent) | 0.01M sodium phosphate (NaH_2_PO_4_) [pH 7.2], 0.9% NaCl |
| **Adenovector Boosting Products** | |
| ChAd63-C | Recombinant attenuated simian adenovirus, chimpanzee adenovirus serotype 63, encoding *P falciparum* CSP cloned from the Pf3D7 strain. The CSP insert is codon-optimized encoding a C-terminally truncated PfCSP protein. (ChAd63 CSP) |
| ChAd63-A | Recombinant attenuated simian adenovirus, chimpanzee adenovirus serotype 63, encoding a tandem array of 2 divergent AMA 1 alleles from 3D7 and FVO strains of Pf^2^. The AMA1 insert in this vaccine is codon-optimized. (ChAd63 AMA1) |
| ChAd63-T | Recombinant simian adenovirus, chimpanzee adenovirus serotype 63, encoding a string of codon-optimized malaria multiple epitopes, ME, fused to a complete Pf TRAP of strain T9/96^3^. (ChAd63 ME-TRAP) |
| Formulation Buffer (FB)  for ChAd63 | 10 mM Histidine, 7.5% sucrose, 35mM NaCl, 1mM MgCl_2_, 0.1% Polysorbate 80, 0.1mM EDTA, 0.5% ethanol, pH 6.6 (this is used for formulation only, not vialed separately) |
| **Priming Regimens**  NMRC-M3V-D-PfCA  NMRC-M3V-D-PfCAT | NMRC-MV-D-PfC and NMRC-MV-D-PfA DNA plasmid prime  NMRC-MV-D-PfC, NMRC-MV-D-PfA, and NMRC-MV-D-PfT DNA plasmid prime |
| **Boosting Regimens**  ChAd63 CA  ChAd63 CAT | ChAd63-C and ChAd63-A adenovector boost  ChAd63-C, ChAd63-A, and ChAd63-T adenovector boost |
| **Prime-Boost Regimens**  NMRC-M3V-D/ChAd63-PfCA (Vaccine 1)  NMRC-M3V-D/ChAd63-PfCAT (Vaccine 2) | NMRC-MV-D-PfC and NMRC-MV-D-PfA DNA plasmid prime; ChAd63-C and ChAd63-A adenovector boost  NMRC-MV-D-PfC, NMRC-MV-D-PfA, and NMRC-MV-D-PfT DNA plasmid prime; ChAd63-C, ChAd63-A, and ChAd63-T adenovector boost |

^1^This construct is similar (codon optimized sequence) to the TRAP construct used in a previous study (native sequence) where 5 DNA vaccine constructs including TRAP were tested in a human clinical trial [7] and were designed prior to this trial and therefore lacked the ME epitope string.

^2^This construct was previously designed and tested at the University of Oxford [27], and therefore contains the FVO allele in addition to the 3D7 allele used in the D-A construct.

^3^This construct was designed and tested at the University of Oxford [15] and contains full length T9/96 which differs to the CHMI strain 3D7, but shares >90% homology. FluoroSpot assays used both 3D7 and T9/96 peptide pools.

Supplementary Table S2: Study Event Schedule and Procedures from Day -90 to Day 195 for Immunized Subjects

| Procedure | Screening | Pre-Imm | DNA #1 | Follow-up | | | DNA #2 | Follow-up | | | DNA #3 | Follow-up | | | | | Ad | Follow-up | | |
| --- | --- | --- | --- | --- | --- | --- | --- | --- | --- | --- | --- | --- | --- | --- | --- | --- | --- | --- | --- | --- |
| Visit Number | 1 | 2 | 3 | 4 | 5 | 6 | 7 | 8 | 9 | 10 | 11 | 12 | 13 | 14 | 15 | 16 | 17 | 18 | 19 | 20 |
| Day relative to 1st immunization a | -90 to -8 | -14 to -1 | 0 | 2 | 7 | 14 | 28 | 30 | 35 | 42 | 56 | 58 | 63 | 70 | 84 | 126 | 168 | 170 | 175 | 195 |
| Window |  | ±7 |  | ±1 | ±3 | ±5 | ±7 | ±1 | ±3 | ±5 | ±7 | ±1 | ±3 | ±5 | ±5 | ±5 | ±7 | ±1 | ±3 | ±7 |
| Day relative to each immunization |  |  | 0 | 2 | 7 | 14 | 0 | 2 | 7 | 14 | 0 | 2 | 7 | 14 | 28 | 70 | 0 | 2 | 7 | 27 |
| **General Procedures** | | | | | | | | | | | | | | | | | | | | |
| Study Briefing | X |  |  |  |  |  |  |  |  |  |  |  |  |  |  |  |  |  |  |  |
| Informed Consent | X |  |  |  |  |  |  |  |  |  |  |  |  |  |  |  |  |  |  |  |
| Assessment of Understanding | X |  |  |  |  |  |  |  |  |  |  |  |  |  |  |  |  |  |  |  |
| Provision of Study Information b |  | X |  |  |  |  |  |  |  |  |  |  |  |  |  |  |  |  |  |  |
| Medical History c | X | X | X | X | X | X | X | X | X | X | X | X | X | X | X | X | X | X | X | X |
| Physical Examination d | X | X | X | X | X | X | X | X | X | X | X | X | X | X | X | X | X | X | X | X |
| Vital Signs e | X | X | X | X | X | X | X | X | X | X | X | X | X | X | X | X | X | X | X | X |
| Height and Weight | X |  |  |  |  |  |  |  |  |  |  |  |  |  |  |  |  |  |  |  |
| Memory Aid Review |  |  |  | X | X |  |  | X | X |  |  | X | X |  |  |  |  | X | X |  |
| Concomitant Medications | X |  | X | X | X | X | X | X | X | X | X | X | X | X | X | X | X | X | X | X |
| Inclusion and Exclusion Criteria | X | X | X |  |  |  | X |  |  |  | X |  |  |  |  |  | X |  |  |  |
| Sickle Cell Screen, G6PD (EDTA) | X |  |  |  |  |  |  |  |  |  |  |  |  |  |  |  |  |  |  |  |
| HIV-1/2 antigen/antibodies, 4^th^ generation screen and confirmation (if needed), HBsAg and anti-HCV (SST) | X |  |  |  |  |  |  |  |  |  |  |  |  |  |  |  |  |  |  |  |
| HuAd5 antibody testing (SST) |  |  |  |  |  |  |  |  |  |  |  |  |  |  |  |  | X |  |  | X |
| EKG | X |  |  |  |  |  |  |  |  |  |  |  |  |  |  |  |  |  |  |  |
| Urine Pregnancy Test | X |  | X |  |  |  | X |  |  |  | X |  |  |  |  |  | X |  |  |  |
| HLA Typing (Buccal swab) |  | X |  |  |  |  |  |  |  |  |  |  |  |  |  |  |  |  |  |  |
| **Product Administration** | | | | | | | | | | | | | | | | | | | | |
| Immunization f g |  |  | X |  |  |  | X |  |  |  | X |  |  |  |  |  | X |  |  |  |
| **Safety Assessments** | | | | | | | | | | | | | | | | | | | | |
| Adverse Events |  |  | X | X | X | X | X | X | X | X | X | X | X | X | X | X | X | X | X | X |
| CBC with differential (EDTA) |  |  | X | X | X |  | X | X | X |  | X | X | X |  | X | X | X | X | X |  |
| Serum Chemistry (SST) |  |  | X | X | X |  | X | X | X |  | X | X | X |  | X | X | X | X | X |  |
| Urinalysis | X |  |  |  |  |  |  |  |  |  |  |  |  |  |  |  |  |  |  |  |
| **Immunogenicity Assessments** | | | | | | | | | | | | | | | | | | | | |
| CSP/AMA1 ELISpot Baseline | X |  |  |  |  |  |  |  |  |  |  |  |  |  |  |  |  |  |  |  |
| CSP/AMA1 ELISA Baseline | X |  |  |  |  |  |  |  |  |  |  |  |  |  |  |  |  |  |  |  |
| Cellular Immunity (FluoroSpot, ICS, Heparin tube) |  | X |  |  |  |  |  |  |  |  |  |  |  |  | X |  | X |  |  | X |
| Humoral Immunity (ELISA, IFA, SST) |  | X |  |  |  | X | X |  |  | X | X |  |  | X | X |  | X |  |  | X |
| Transcriptome |  |  | X | X | X | X | X | X | X | X | X | X | X | X |  |  | X | X | X |  |
| **Blood Volumes** | | | | | | | | | | | | | | | | | | | | |
| Daily (mL) | 33^h^ | 150 | 11.5 | 11.5 | 11.5 | 12.5 | 21.5 | 11.5 | 11.5 | 12.5 | 21.5 | 11.5 | 11.5 | 12.5 | 159 k | 9 | 162 ^i^ | 11.5 | 11.5 | 150 h |
| Cumulative (mL) | 33 | 183 | 195 | 206 | 218 | 230 | 252 | 263 | 275 | 287 | 309 | 320 | 332 | 344 | 503 | 512 | 674 | 685 | 697 | 847 |

a The day relative to the first immunization will be documented per the table, even if the full window period is used.

b Study information includes a study schedule and a contact card that contains who to contact and how to contact the research site.

c Complete at screening, then noting interim changes in the medical history at other visits.

d Detailed at screening, then focused or targeted exams at other visits.

e Vital signs include temperature, heart rate, and blood pressure.

f Subjects will remain at the CTC for observation for at least 30 minutes following each immunization. After the 30-minute observation period, temperature, pulse rate, and blood pressure will be measured, and any adverse events will be recorded. Subjects will also have a telephone follow-up within 24 hours of each immunization.

g Subjects will be given a memory aid, ruler, and digital thermometer.

^h^ Screening includes ALT, AST, alkaline phosphatase, BUN, creatinine, glucose, calcium, and total bilirubin. Other visits only include ALT, AST, alkaline phosphatase, total bilirubin, and creatinine.

^i^ Blood volumes of 162 mL include 140 mL for cellular assays, 10 mL for ELISA and HuAd5, 9 mL for safety labs, and 2.5 mL for Transcriptome.

^j^ Blood volume of 150 mL include 140 mL for cellular assays, 10 mL for ELISA and HuAd5.

^k^ Blood volumes of 159 mL include 140 mL for cellular assays and 10 mL for ELISA and 9 mL for safety.

Supplementary Table S3: Study Event Schedule and Procedures from Day 196 to Day 286 for Immunized Subjects

| Procedure | CHMI | Day 1 | Post Challenge Hotel Phase | | | | | | | | | | Post Challenge  Follow-up a | | | | Final Visit b | | | |
| --- | --- | --- | --- | --- | --- | --- | --- | --- | --- | --- | --- | --- | --- | --- | --- | --- | --- | --- | --- | --- |
| Visit Number | 21 | 22 | 23 | 24 | 25 | 26 | 27 | 28 | 29 | 30 | 31 | 32 | 33 | 34 | 35 | 36 | 37 | 38 | 39 | 40 |
| Day relative to 1st immunization c | 196 | 197 | 203 | 204 | 205 | 206 | 207 | 208 | 209 | 210 | 211 | 212 | 213 | 214 | 216 | 218 | 221 | 224 | 231 | 286 |
| Window | ±7 | ±1 |  |  |  |  |  |  |  |  |  |  |  |  | ±1 | ±1 | ±2 | ±3 | ±5 | ±14 |
| Day relative to CHMI | 0 |  | 7 | 8 | 9 | 10 | 11 | 12 | 13 | 14 | 15 | 16 | 17 | 18 | 20 | 22 | 25 | 28 | 35 | 90 |
| **General Procedures** |  |  |  |  |  |  |  |  |  |  |  |  |  |  |  |  |  |  |  |  |
| Medical History d | X |  | X | X | X | X | X | X | X | X | X | X | X | X | X | X | X | X | X | X |
| Physical Examination e | X |  | X | X | X | X | X | X | X | X | X | X | X | X | X | X | X | X | X | X |
| Vital Signs f | X |  | X | X | X | X | X | X | X | X | X | X | X | X | X | X | X | X | X | X |
| Concomitant Medications | X |  | X | X | X | X | X | X | X | X | X | X | X | X | X | X | X | X | X | X |
| Inclusion and Exclusion Criteria | X |  |  |  |  |  |  |  |  |  |  |  |  |  |  |  |  |  |  |  |
| Urine Pregnancy Test | X |  |  |  |  |  |  |  |  |  |  |  |  |  |  |  |  |  |  |  |
| Adverse Events | X | X | X | X | X | X | X | X | X | X | X | X | X | X | X | X | X | X | X | X |
| CBC with differential (EDTA) g | X |  |  |  |  |  |  |  |  |  |  |  |  |  |  |  |  | X |  | X |
| Serum Chemistry (SST) g | X |  |  |  |  |  |  |  |  |  |  |  |  |  |  |  |  | X |  | X |
| Cellular Immunity (FluoroSpot, ICS; Heparine tube) |  |  |  |  |  |  |  |  |  |  |  |  |  |  |  |  |  |  | X | X |
| Humoral Immunity (ELISA, IFA, SST) |  |  |  |  |  |  |  |  |  |  |  |  |  |  |  |  |  |  | X | X |
| Transcriptome | X | X | X |  |  |  |  |  |  | X h |  |  |  |  |  |  |  |  |  |  |
| **CHMI Specific Procedures** |  |  |  |  |  |  |  |  |  |  |  |  |  |  |  |  |  |  |  |  |
| Sporozoite Challenge i | X |  |  |  |  |  |  |  |  |  |  |  |  |  |  |  |  |  |  |  |
| Malaria Smear/PCR (EDTA) |  |  | X | X | X | X | X | X | X | X | X | X | X | X | X | X | X | X |  |  |
| **Blood Volumes** | | | | | | | | | | | | | | | | | | | | |
| Daily (mL) | 11.5 | 2.5 | 6.5 | 4 j | 13 k | 4 | 4 | 13 l | 4 | 6.5 | 4 | 4 | 4 | 4 | 4 | 4 | 4 | 13 | 150 m | 159 n |
| Cumulative (mL) | 858 | 861 | 867 | 871 | 884 | 888 | 892 | 905 | 909 | 916 | 920 | 924 | 928 | 932 | 936 | 940 | 944 | 957 | 1107 | 1266 |

a The Day 20, 22, 25, and 28 visits are for subjects who remain negative for parasitemia. All subjects treated and discharged from the hotel are to come to the CTC for the Day 28 Post CHMI visit.

b There will be a telephone follow-up at 6 months and 12 months after the challenge.

c The day relative to the first immunization will be documented per the table, even if the full window period is used.

d Complete at screening, then noting interim changes in the medical history at other visits.

e Detailed at screening, then focused or targeted exams at other visits.

f Vital signs include temperature, heart rate, and blood pressure.

g Also, at any time point post challenge when deemed clinically indicated by the investigator.

h Screening includes ALT, AST, alkaline phosphatase, BUN, creatinine, glucose, calcium, and total bilirubin. Other visits only include ALT, AST, alkaline phosphatase, total bilirubin, and creatinine.

h To be obtained on completion of treatment before discharge from the hotel phase. Day 14 time point is approximate.

i Subjects will be closely observed for at least 30 minutes following the challenge. After the 30-minute observation period temperature, pulse rate, and blood pressure will be measured. Subjects will be counseled to use methods that will reduce risk of exposure to mosquitoes beginning 5 days after challenge until 20 days after the challenge.

j Blood volume of 4 mL includes 2 mL for malaria smear and 2 mL for RT-PCR for *P falciparum*, the latter will be evaluated on a retrospective basis.

k Blood volume of 13 mL includes 2 mL for malaria smear and 2 mL for RT-PCR for *P falciparum* as part of daily evaluation and 9 mL for safety laboratory tests obtained at the onset of parasitemia. Parasitemia can occur at any time between Day 7 and Day 18 post-challenge.

l Blood volume of 13 mL includes 2 mL for malaria smear and 2 mL for RT-PCR for *P falciparum* as part of daily evaluation and 9 mL for safety laboratory tests obtained at 72 hours after the onset of parasitemia.

m Blood volume of 150 mL include 140 mL for cellular assays, 10 mL for ELISA.

n Blood volume of 159 mL includes 140 mL for cellular assays, 10 mL for ELISA, and 9 mL for safety laboratory tests.

Supplementary Table S4: Study Event Schedule and Procedure for Infectivity Control Subjects

| Procedure | Screening | Pre-CHMI | CHMI | Day 1 | Post Challenge Hotel Phase | | | | | | | | | | | | Post Challenge Follow-up a | | | | | Final Visit b |
| --- | --- | --- | --- | --- | --- | --- | --- | --- | --- | --- | --- | --- | --- | --- | --- | --- | --- | --- | --- | --- | --- | --- |
| Visit Number | 1 | 2 | 3 | 4 | 5 | 6 | 7 | 8 | 9 | 10 | 11 | 12 | 13 | 14 | 15 | 16 | 17 | 18 | 19 | 20 | 21 | 22 |
| Day relative to CHMI c | -90 to -8 | -14 to -1 | 0 | 1 | 7 | 8 | 9 | 10 | 11 | 12 | 13 | 14 | 15 | 16 | 17 | 18 | 20 | 22 | 25 | 28 | 35 | 90 |
| Window |  | ±7 |  | +1 |  |  |  |  |  |  |  |  |  |  |  |  | ±1 | ±1 | ±2 | ±3 | ±5 | ±14 |
| **General Procedures** | | | | | | | | | | | | | | | | | | | | | | |
| Study Briefing | X |  |  |  |  |  |  |  |  |  |  |  |  |  |  |  |  |  |  |  |  |  |
| Informed Consent | X |  |  |  |  |  |  |  |  |  |  |  |  |  |  |  |  |  |  |  |  |  |
| Assessment of Understanding | X |  |  |  |  |  |  |  |  |  |  |  |  |  |  |  |  |  |  |  |  |  |
| Provision of Study Information d |  | X |  |  |  |  |  |  |  |  |  |  |  |  |  |  |  |  |  |  |  |  |
| Medical History e | X | X | X |  | X | X | X | X | X | X | X | X | X | X | X | X | X | X | X | X | X | X |
| Physical Examination f | X | X | X |  | X | X | X | X | X | X | X | X | X | X | X | X | X | X | X | X | X | X |
| Vital Signs g | X | X | X |  | X | X | X | X | X | X | X | X | X | X | X | X | X | X | X | X | X | X |
| Height and Weight | X |  |  |  |  |  |  |  |  |  |  |  |  |  |  |  |  |  |  |  |  |  |
| Concomitant Medications | X |  | X |  | X | X | X | X | X | X | X | X | X | X | X | X | X | X | X | X | X | X |
| Inclusion and Exclusion Criteria | X | X | X |  |  |  |  |  |  |  |  |  |  |  |  |  |  |  |  |  |  |  |
| Sickle Cell Screen, G6PD (EDTA) | X |  |  |  |  |  |  |  |  |  |  |  |  |  |  |  |  |  |  |  |  |  |
| HIV-1/2 antigen/antibodies 4^th^ generation screen and confirmation (if needed), HBsAg and anti-HCV (SST) | X |  |  |  |  |  |  |  |  |  |  |  |  |  |  |  |  |  |  |  |  |  |
| EKG | X |  |  |  |  |  |  |  |  |  |  |  |  |  |  |  |  |  |  |  |  |  |
| Urine Pregnancy Test | X |  | X |  |  |  |  |  |  |  |  |  |  |  |  |  |  |  |  |  |  |  |
| **Safety Assessments** | | | | | | | | | | | | | | | | | | | | | | |
| Adverse Events |  |  | X | X | X | X | X | X | X | X | X | X | X | X | X | X | X | X | X | X | X | X |
| CBC with Differential (EDTA) h | X |  | X |  |  |  |  |  |  |  |  |  |  |  |  |  |  |  |  | X |  | X |
| Serum Chemistry (SST) h i | X |  | X |  |  |  |  |  |  |  |  |  |  |  |  |  |  |  |  | X |  | X |
| Urinalysis | X |  |  |  |  |  |  |  |  |  |  |  |  |  |  |  |  |  |  |  |  |  |
| **Immunogenicity Assessments** | | | | | | | | | | | | | | | | | | | | | | |
| CSP/AMA1 ELISpot Baseline | X |  |  |  |  |  |  |  |  |  |  |  |  |  |  |  |  |  |  |  |  |  |
| CSP/AMA1 ELISA Baseline | X |  |  |  |  |  |  |  |  |  |  |  |  |  |  |  |  |  |  |  |  |  |
| Cellular Immunity (FluoroSpot, ICS; heparine tube) |  | X |  |  |  |  |  |  |  |  |  |  |  |  |  |  |  |  |  |  | X | X |
| Humoral Immunity (ELISA, IFA, SST) |  | X |  |  |  |  |  |  |  |  |  |  |  |  |  |  |  |  |  |  | X | X |
| Transcriptome |  |  | X | X | X |  |  |  |  |  |  | X^p^ |  |  |  |  |  |  |  |  |  |  |
| **CHMI Specific Procedures** | | | | | | | | | | | | | | | | | | | | | | |
| Sporozoite Challenge j |  |  | X |  |  |  |  |  |  |  |  |  |  |  |  |  |  |  |  |  |  |  |
| Malaria Smear/PCR |  |  |  |  | X | X | X | X | X | X | X | X | X | X | X | X | X | X | X | X |  |  |
| **Blood Volumes** | | | | | | | | | | | | | | | | | | | | | | |
| Daily volume (mL) | 33 | 150 | 11.5 | 2.5 | 6.5 | 4 k | 13 l | 4 | 4 | 13 m | 4 | 6.5 | 4 | 4 | 4 | 4 | 4 | 4 | 4 | 13 | 150 n | 159 o |
| Cumulative volume (mL) | 33 | 183 | 195 | 197 | 204 | 208 | 221 | 225 | 229 | 242 | 246 | 252 | 256 | 260 | 264 | 268 | 272 | 276 | 280 | 293 | 452 | 611 |
| **Physiological Status Monitoring** |  |  |  |  |  |  |  |  |  |  |  |  |  |  |  |  |  |  |  |  |  |  |
| Distribution of Devices |  | X |  |  |  |  |  |  |  |  |  |  |  |  |  |  |  |  |  |  |  |  |
| Training on use of devices |  | X |  |  |  |  |  |  |  |  |  |  |  |  |  |  |  |  |  |  |  |  |
| Collect/Download Data |  | X | X |  | X |  |  |  |  |  |  | X^p^ |  |  |  |  | X |  |  | X |  |  |
| Collect Devices |  |  |  |  |  |  |  |  |  |  |  |  |  |  |  |  |  |  |  | X |  |  |

a The Day 20, 22, 25, and 28 visits are for subjects who remain negative for parasitemia. All subjects treated and discharged from the hotel are to come to the CTC for the Day 28 Post CHMI visit.

b There will be a telephone follow-up at 6 months and 12 months after the challenge.

c The day relative to the first immunization will be documented per the table, even if the full window period is used.

d Study information includes a study schedule and a contact card that contains who to contact and how to contact the research site.

e Complete at screening, then noting any interim changes in the medical history at other visits.

f Detailed at screening, then focused or targeted exams at other visits. Screening includes cardiovascular disease risk evaluation [37}.

g Vital signs include temperature, heart rate, and blood pressure.

h Also, at any time point post challenge when deemed clinically indicated by the investigator.

i Screening includes ALT, AST, alkaline phosphatase, BUN, creatinine, glucose, calcium and total bilirubin. Other visits only include ALT, AST, alkaline phosphatase, total bilirubin, and creatinine.

j Subjects will be closely observed for at least 30 minutes following the challenge. After the 30-minute observation period temperature, pulse rate, and blood pressure will be measured. Subjects will be counseled to use methods that will reduce risk of exposure to mosquitoes beginning 5 days after challenge until 28 days post-challenge.

k Blood volumes of 4 mL include 2 mL for malaria smear and 2 mL for RT-PCR for *P falciparum*, the latter will be analyzed on a retrospective basis.

l Blood volume of 13 mL includes 2 mL for malaria smear and 2 mL for RT-PCR for *P falciparum* as part of daily evaluation and safety laboratory tests at the onset of parasitemia.

m Blood volume of 13 mL includes 2 mL for malaria smear and 2 mL for RT-PCR for *P falciparum* as part of daily evaluation and 9 mL for safety laboratory tests at 72 hours after the onset of parasitemia.

n Blood volume of 150 mL includes 140 mL for cellular assays and 10 mL for ELISA.

o Blood volumes of 159 mL include 140 mL for cellular assays, 10 mL for ELISA, and 9 mL for safety laboratory tests.

^p^ To be obtained on completion of treatment before discharge from the hotel phase. Day 14 time point is approximate.

**Supplementary Table S5: Laboratory AEs**

|  | **CA** | | | | | | | | | | |  | **CAT** | | | | | | | | | |
| --- | --- | --- | --- | --- | --- | --- | --- | --- | --- | --- | --- | --- | --- | --- | --- | --- | --- | --- | --- | --- | --- | --- |
| *Immunization*  *Lab Analyte*  *(# Subjects)^2,5^* | |  | **Mild** | | **Moderate** | | | **Severe** | | **Life-Threatening** | | *Immunization*  *Lab Analyte*  *(# Subjects)^2^* | |  | **Mild** | | **Moderate** | | **Severe** | | **Life-Threatening** | |
|  | | Total^3^ | R | NRRRRR | R | | NR | R | NR | R | NR |  | | Total^3^%)^3^ | R | NR | R | NR | R | NR | R | NR |
| *DNA 1 (20)^4^* | | 13 | 1  8 | 7  27 | 1 | | 4 | - | - | - | - | *DNA 1 (20)^4^* | | 10 | - | 8 | - | 2 | - | - | - | - |
| WBC | | 1 | -^6^ | 1 | - | | - | - | - | - | - | WBC | | 1 | - | 1 | - | - | - | - | - | - |
| Hgb | | 7 | - | 4 | - | | 3 | - | - | - | - | Hgb | | 5 | - | 4 | - | 1 | - | - | - | - |
| ANC | | 7 | 1 | 4 | 1 | | 1 | - | - | - | - | ANC | | 2 | - | 2 | - | - | - | - | - | - |
| TBil | | 1 | - | 1 | - | | - | - | - | - | - | TBil | | 1 | - | - | - | 1 | - | - | - | - |
|  | |  |  |  |  | |  |  |  |  |  | ALT | | 1 | - | 1 | - | - | - | - | - | - |
| *DNA 2 (20)^4^* | | 12 | - | 7 | 1 | | 4 | - | - | - | - | *DNA 2 (20)^4^* | | 12 | 1 | 9 | - | 1 | - | 1 | - | - |
| WBC | | 2 | 1 | 1 | - | | - | - | - | - | - | WBC | | 3 | - | 3 | - | - | - | - | - | - |
| Hgb | | 5 | - | 2 | - | | 3 | - | - | - | - | Hgb | | 7 | - | 7 | - | - | - | - | - | - |
| ANC | | 7 | - | 5 | 1 | | 1 | - | - | - | - | ANC | | 3 | - | 2 | - | - | - | 1 | - | - |
| ALT | | 2 | - | 2 | - | | - | - | - | - | - | ALT | | 2 | - | 2 | - | - | - | - | - | - |
| AST | | 2 | - | 2 | - | | - | - | - | - | - | AST | | 2 | - | 1 | - | 1 | - | - | - | - |
|  | |  |  |  |  | |  |  |  |  |  | ALP | | 2 | 1 | 1 | - | - | - | - | - | - |
|  | |  |  |  |  | | - | - | - | - | - |  | |  |  |  |  |  |  |  |  |  |
| *DNA 3 (20)^4^* | | 11 | 1 | 7 | 1 | | 2 | - | - | - | - | *DNA 3 (18)^4^* | | 11 | - | 9 | - | 2 | - | - | - | - |
| WBC | | 1 | - | 1 | - | | - | - | - | - | - | WBC | | 2 | - | 2 | - | - | - | - | - | - |
| Hgb | | 3 | - | 2 | - | | 1 | - | - | - | - | Hgb | | 5 | - | 5 | - | - | - | - | - | - |
|  | |  |  |  |  | |  |  |  |  |  | PLT | | 1 | - | 1 | - | - | - | - | - | - |
| ANC | | 7 | - | 6 | 1 | | - | - | - | - | - | ANC | | 1 | - | - | - | 1 | - | - | - | - |
| ALC | | 1 | - | 1 | - | | - | - | - | - | - |  | |  |  |  |  |  |  |  |  |  |
| AEC | | 1 | - | 1 | - | | - | - | - | - | - | AEC | | 1 | - | - | - | 1 | - | - | - | - |
|  | |  |  |  |  | |  |  |  |  |  | CRN | | 1 | - | 1 | - | - | - | - | - | - |
| TBil | | 1 | - | - | - | | 1 | - | - | - | - | TBiL | | 1 | - | 1 | - | - | - | - | - | - |
|  | |  |  |  |  | |  |  |  |  |  | ALP | | 3 | - | 3 | - | - | - | - | - | - |
| ALT | | 2 | - | 2 | - | | - | - | - | - | - | ALT | | 3 | - | 3 | - | - | - | - | - | - |
| AST | | 2 | - | 2 | - | | -- | - | - | - | - | AST | | 2 | - | 2 | - | - | - | - | - | - |
|  | **Cohort CA** | | | | | | | | | | |  | **Cohort CAT** | | | | | | | | | |
| *ChAd63 (19)^4^* | | 15 | 4 | 3 | | 3 | 3 | 1 | - | 1 | - | *ChAd63 (16)^4^* | | 11 | 4 | 1 | 2 | 1 | 3 | - | - | - |
| WBC | | 3 | 2 | 1 | | - | - | - | - | - | - | WBC | | 3 | 2 | 1 | - | - | - | - | - | - |
| Hgb | | 4 | - | 2 | | - | 2 | - | - | - | - | Hgb | | 2 | - | 2 | - | - | - | - | - | - |
| ANC | | 10 | 1 | 4 | | 3 | - | 1 | - | 1 | - | ANC | | 9 | 5 | - | 1 | 1 | 2 | - | - | - |
| ALC | | 3 | 2 | - | | - | 1 | - | - | - | - | ALC | | 2 | 1 | - | - | - | 1 | - | - | - |
|  | |  |  |  | |  |  |  |  |  |  | TBil | | 1 | - | 1 | - | - | - | - | - | - |
| ALT | | 3 | 1 | 2 | | - | - | - | - | - | - | ALT | | 1 | 1 | - | - | - | - | - | - | - |
| AST | | 2 | 1 | 1 | | - | - | - | - | - | - | AST | | 1 | - | - | 1 | - | - | - | - | - |
|  | |  |  |  | |  |  |  |  |  |  |  | |  |  |  |  |  |  |  |  |  |

^1^ Number of reported lab abnormal events for Cohorts CA and CAT following initial immunization (DNA 1) through 28 days post each immunization

^2^ Number of subjects who completed immunization/boost

^3^ Number of subjects with reported events and relationship to study product (**R**: definite/probable/possible related or **NR**: unlikely/ not related). Individual subjects may report multiple events. A subject experiencing multiple events is counted once at the highest severity for each lab analyte following each immunization.

^4^ Number of subjects with reported events at the highest severity for any lab analyte.

^5^ Total number of subjects with reported events will not equal the sum of subjects by individual lab analytes since a subject may have experienced more than one lab analyte abnormality.

^6^ No abnormality recorded.

Note, following baseline (first immunization), safety laboratory tests were performed 2, 7 and 28 days after each immunization: Day 126 and Day 168.

Analyte abbreviations: WBC White Blood Cells; HgB Hemoglobin, ANC Absolute Neutrophil Count; ALC Absolute Lymphocyte Count; ALT Alanine Aminotransferase; AST Aspartate Aminotransferase; ALP Alkaline phosphatase; TBil Total Bilirubin; PLT Platelet count.

**Supplementary Table S6. Immunized Cohorts CA and CAT: Unsolicited adverse events for 28 days following each immunization**

| **Vaccine** | **Post DNA1 (n=20)** | **Post DNA2 (n=20)** | **Post DNA3 (n=20)** | **ChAd63 (n=19)** | **Total** |
| --- | --- | --- | --- | --- | --- |
| **CA** |  |  |  |  |  |
| Erythema in oropharynx |  |  |  | Possible, Gr1 | **1** |
| Ecchymosis left deltoid (not injection site) |  |  | Possible, Gr1 |  | **1** |
| Cutaneous macular eruption (rash) on trunk, chest and about rib cage |  |  |  | Possible, Gr1 | **1** |
| **Total** | **0** | **0** | **1** | **2** | **3** |
|  | **Post DNA1 (n=20)** | **Post DNA2 (n=20)** | **Post DNA3 (n=18)** | **ChAd (n=16)** | **Total** |
| **CAT** |  |  |  |  |  |
| Lower back/spine pain |  |  |  | Possible, Gr2 | **1** |
| Photophobia |  |  |  | Possible, Gr2 | **1** |
| **Total** | **0** | **0** | **0** | **2** | **2** |
| **Total all AE’s** | **0** | **0** | **1** | **4** | **5** |

Unsolicited adverse events were assessed starting on day of immunization (following receipt of vaccine) through Day 28 after each immunization. Severity classification: Gr1= adverse event does not interfere with daily activities; Gr2= interferes with but does not prevent daily activities; Gr3= prevents daily activities.

**Supplementary Table S7: CA and CAT: ELISA antibody responses to CSPFL**

| **CA** | | | | | | | | | | | |
| --- | --- | --- | --- | --- | --- | --- | --- | --- | --- | --- | --- |
|  | **Visit #2** | **Visit #6** | **Visit #7** | **Visit #10** | **Visit #11** | **Visit #14** | **Visit #15** | **Visit #17** | **Visit #20** | **Visit #39** | **Visit #40** |
| **Subject ID** | **PreVac** | **14 days post DNA #1** | **DNA Imm #2** | **14 d post DNA Imm #2** | **DNA Imm #3** | **14 d post DNA Imm #3** | **28 d post DNA Imm #3** | **Pre ChAd63 boost** | **Pre-CHMI** | **35 d post CHMI** | **90 d post CHMI** |
| **01** | 63 | 67 | 99 | 523 | 348 | 696 | 522 | 202 | 11890 | 4264 | 2490 |
| **78** | 63 | 46 | 54 | 87 | 318 | 730 | 885 | 643 | 30700 | 11740 | 8720 |
| **68** | 10 | 11 | 20 | 89 | 101 | 91 | 102 | 57 | 2663 | 1056 | 564 |
| **88** | 45 | 58 | 53 | 59 | 59 | 81 | 83 | 61 | 726 | 467 | 311 |
| **81** | 17 | 12 | 12 | 12 | 0 | 26 | 67 | 54 | 1595 | 690 | 385 |
| **08** | 13 | 12 | 12 | 12 | 13 | 18 | 16 | 14 | 1308 | 798 | 384 |
| **82** | 60 | 58 | 59 | 70 | 79 | 93 | 95 | 82 | 7670 | 3058 | 1310 |
| **90** | 145 | 129 | 198 | 919 | 1310 | 1542 | 1518 | 1315 | 8700 | 5050 | 4410 |
| **83** | 13 | 9 | 7 | 8 | 9 | 16 | 27 | 15 | 2063 | 1300 | 910 |
| **63** | 14 | 13 | 13 | 15 | 47 | 91 | 176 | 168 | 3009 | 2220 | 1880 |
| **20** | 59 | 55 | 53 | 67 | 95 | 120 | 140 | 194 | 2007 | 672 | 331 |
| **40** | 42 | 33 | 32 | 34 | 36 | 55 | 73 | 57 | 2158 | 1079 | 506 |
| **59** | 51 | 48 | 45 | 49 | 79 | 157 | 146 | 96 | 5250 | 2723 | 1490 |
| **32** | 20 | 15 | 16 | 45 | 75 | 90 | 86 | 56 | 1010 | 655 | 301 |
| **53** | 20 | 19 | 20 | 19 | 21 | 23 | 35 | 35 | 1683 | 950 | 756 |
| **41** | 44 | 48 | 49 | 58 | 79 | 89 | 90 | 71 | 297 | 366 | 362 |
| **GM** | 32.28 | 29.30 | 31.70 | 49.88 | 57.06 | 97.78 | 116.56 | 88.32 | 2678.07 | 1419.72 | 873.28 |
| **CAT** | | | | | | | | | | | |
| **17** | 19 | 17 | 19 | 27 | 40 | 50 | 86 | 68 | 3392 | 1988 | 1480 |
| **57** | 29 | 27 | 30 | 33 | 63 | 105 | 138 | 85 | 7220 | 2547 | 1400 |
| **09** | 23 | 24 | 23 | 26 | 45 | 76 | 82 | 53 | 1138 | 402 | 262 |
| **73** | 371 | 415 | 413 | 583 | 715 | 731 | 700 | 573 | 4447 | 2850 | 2300 |
| **74** | 12 | 13 | 13 | 28 | 84 | 125 | 119 | 101 | 4868 | 2281 | 1590 |
| **15** | 14 | 17 | 17 | 35 | 133 | 200 | 321 | 73 | 3368 | 2110 | 1800 |
| **33** | 21 | 21 | 38 | 296 | 242 | 328 | 224 | 161 | 6280 | 2848 | 1510 |
| **70** | 18 | 18 | 16 | 56 | 55 | 120 | 136 | 94 | 6450 | 2556 | 1450 |
| **12** | 103 | 138 | 233 | 3230 | 7210 | 7210 | 9330 | 5152 | 22200 | 14750 | 11100 |
| **34** | 72 | 75 | 72 | 84 | 98 | 98 | 99 | 79 | 638 | 580 | 218 |
| **76** | 38 | 35 | 40 | 37 | 46 | 87 | 109 | 51 | 1075 | 700 |  |
| **46** | 44 | 42 | 44 | 197 | 301 | 683 | 1012 | 424 | 9280 | 5277 | 1720 |
| **24** | 145 | 167 | 157 | 272 | 229 | 343 | 361 | 230 | 3373 | 1771 | 1270 |
| **75** | 22 | 18 | 17 | 18 | 81 | 361 | 461 | 594 | 9680 | 6090 | 4250 |
| **64** | 1957 | 2659 | 3152 | 3919 | 3749 | 3639 | 3650 | 2774 | 24300 | 11570 | 7430 |
| **35** | 16 | 16 | 16 | 27 | 87 | 122 | 158 | 152 | 4630 | 2157 | 957 |
| **GM** | 45.76 | 47.80 | 52.04 | 105.19 | 174.12 | 266.88 | 311.23 | 205.26 | 4617.60 | 2453.91 | 1608.63 |

Responses were measured pre-vaccination (PreVac), 14 days after the first (DNA1), and second (DNA2), and 14 days and 28 days after the third (DNA3) DNA immunizations, pre-ChAd63 boost and post-ChAd63/pre-CHMI, 35 days and 90 days post-CHMI. GM=geometric mean.

**Supplementary Table S8: CA and CAT: ELISA antibody responses to CSPrp**

| **CA** | | | | | | | | | | | |
| --- | --- | --- | --- | --- | --- | --- | --- | --- | --- | --- | --- |
|  | **Visit #2** | **Visit #6** | **Visit #7** | **Visit #10** | **Visit #11** | **Visit #14** | **Visit #15** | **Visit #17** | **Visit #20** | **Visit #39** | **Visit #40** |
| **Subject ID** | **PreVac** | **14 days post DNA #1** | **DNA Imm #2** | **14 d post DNA Imm #2** | **DNA Imm #3** | **14 d post DNA Imm #3** | **28 d post DNA Imm #3** | **Pre ChAd63 boost** | **Pre-CHMI** | **35 d post CHMI** | **90 d post CHMI** |
| **01** | 19 | 16 | 41 | 457 | 260 | 685 | 459 | 129 | 4446 | 1158 | 575 |
| **78** | 10 | 11 | 12 | 21 | 35 | 70 | 186 | 157 | 25800 | 8330 | 4024 |
| **68** | 9 | 10 | 11 | 71 | 72 | 69 | 69 | 43 | 431 | 434 | 226 |
| **88** | 12 | 13 | 12 | 13 | 14 | 54 | 51 | 32 | 477 | 271 | 173 |
| **81** | 18 | 20 | 20 | 18 | 18 | 20 | 24 | 23 | 214 | 89 | 42 |
| **08** | 8 | 11 | 11 | 12 | 12 | 13 | 9 | 8 | 99 | 48 | 21 |
| **82** | 19 | 17 | 19 | 31 | 34 | 41 | 48 | 38 | 532 | 243 | 130 |
| **90** | 46 | 42 | 105 | 708 | 808 | 1027 | 929 | 694 | 4522 | 3088 | 2600 |
| **83** | 15 | 15 | 13 | 14 | 15 | 19 | 26 | 21 | 1088 | 686 | 527 |
| **63** | 13 | 13 | 13 | 12 | 15 | 21 | 53 | 57 | 198 | 254 | 239 |
| **20** | 61 | 60 | 60 | 71 | 95 | 124 | 142 | 169 | 326 | 254 | 256 |
| **40** | 14 | 12 | 13 | 13 | 14 | 29 | 40 | 28 | 866 | 455 | 273 |
| **59** | 10 | 12 | 13 | 12 | 25 | 56 | 68 | 28 | 1607 | 889 | 394 |
| **32** | 17 | 19 | 18 | 25 | 36 | 40 | 36 | 36 | 129 | 99 | 68 |
| **53** | 26 | 15 | 15 | 14 | 15 | 17 | 18 | 16 | 651 | 224 | 125 |
| **41** | 10 | 11 | 11 | 11 | 14 | 16 | 17 | 18 | 81 | 84 | 52 |
| **GM** | 16.03 | 16.03 | 18.20 | 29.34 | 33.93 | 50.36 | 58.12 | 44.33 | 640.34 | 369.60 | 224.40 |
| **CAT** | | | | | | | | | | | |
| **17** | 13 | 10 | 10 | 13 | 14 | 17 | 23 | 19 | 708 | 424 | 441 |
| **57** | 30 | 36 | 31 | 32 | 44 | 66 | 78 | 70 | 3872 | 1377 | 711 |
| **09** | 12 | 12 | 12 | 14 | 21 | 37 | 40 | 25 | 288 | 158 | 134 |
| **73** | 13 | 14 | 15 | 41 | 66 | 111 | 130 | 70 | 634 | 353 | 161 |
| **74** | 12 | 17 | 17 | 17 | 17 | 18 | 17 | 18 | 1624 | 616 | 490 |
| **15** | 11 | 11 | 11 | 15 | 40 | 62 | 88 | 36 | 643 | 1027 | 957 |
| **33** | 11 | 11 | 11 | 57 | 55 | 168 | 162 | 87 | 2090 | 1115 | 625 |
| **70** | 21 | 16 | 17 | 42 | 79 | 86 | 97 | 74 | 1420 | 706 | 445 |
| **12** | 13 | 13 | 14 | 199 | 218 | 226 | 261 | 124 | 1484 | 961 | 732 |
| **34** | 33 | 25 | 24 | 27 | 35 | 35 | 34 | 27 | 106 | 108 | 43 |
| **76** | 9 | 11 | 11 | 11 | 12 | 17 | 25 | 16 | 187 | 206 |  |
| **46** | 27 | 30 | 34 | 91 | 102 | 332 | 305 | 144 | 2480 | 942 | 366 |
| **24** | 22 | 13 | 12 | 17 | 16 | 40 | 63 | 32 | 731 | 330 | 209 |
| **75** | 10 | 12 | 12 | 12 | 17 | 73 | 99 | 353 | 8000 | 5535 | 2944 |
| **64** | 572 | 1316 | 1517 | 1902 | 1806 | 1639 | 1711 | 1193 | 6690 | 2536 | 1783 |
| **35** | 12 | 10 | 11 | 14 | 25 | 29 | 39 | 31 | 933 | 514 | 338 |
| **GM** | 19.01 | 19.50 | 19.87 | 34.62 | 45.06 | 71.44 | 84.03 | 60.39 | 1092.70 | 639.25 | 434.92 |

Responses were measured pre-vaccination (PreVac), 14 days after the first (DNA1), and second (DNA2), and 14 days and 28 days after the third (DNA3) DNA immunizations, pre-ChAd63 boost and post-ChAd63/pre-CHMI, 35 days and 90 days post-CHMI. GM=geometric mean. Empty boxes indicate when no test was performed.

**Supplementary Table S9: CA and CAT: ELISA antibody responses to Pf16**

| **CA** | | | | | | | | | | | |
| --- | --- | --- | --- | --- | --- | --- | --- | --- | --- | --- | --- |
|  | **Visit #2** | **Visit #6** | **Visit #7** | **Visit #10** | **Visit #11** | **Visit #14** | **Visit #15** | **Visit #17** | **Visit #20** | **Visit #39** | **Visit #40** |
| **Subject**  **ID** | **PreVac** | **14 days post DNA #1** | **DNA Imm #2** | **14 d post DNA Imm #2** | **DNA Imm #3** | **14 d post DNA Imm #3** | **28 d post DNA Imm #3** | **Pre ChAd63 boost** | **Pre-CHMI** | **35 d post CHMI** | **90 d post CHMI** |
| **01** | 417 | 311 | 354 | 442 | 369 | 388 | 500 | 307 | 6890 | 2540 | 988 |
| **78** | 206 | 243 | 289 | 366 | 383 | 330 | 371 | 412 | 5381 | 2124 | 1132 |
| **68** | 726 | 647 | 403 | 540 | 561 | 574 | 532 | 599 | 2301 | 1414 | 659 |
| **88** | 770 | 909 | 847 | 939 | 934 | 968 | 1082 | 1227 | 1527 | 1424 | 1039 |
| **81** | 4965 | 5460 | 5400 | 5410 | 5550 | 4205 | 3179 | 4422 | 4720 | 4480 | 4922 |
| **08** | 392 | 403 | 397 | 341 | 409 | 387 | 333 | 448 | 716 | 568 | 688 |
| **82** | 51 | 44 | 45 | 45 | 49 | 50 | 51 | 51 | 3889 | 1520 | 929 |
| **90** | 44000 | 36600 | 38000 | 34400 | 47200 | 49900 | 44100 | 50800 | 50900 | 49000 | 48900 |
| **83** | 327 | 302 | 262 | 281 | 284 | 287 | 284 | 270 | 708 | 522 | 430 |
| **63** | 2104 | 2243 | 1770 | 1792 | 1795 | 1815 | 1785 | 1604 | 2519 | 2278 | 2291 |
| **20** | 311 | 273 | 279 | 297 | 265 | 261 | 310 | 304 | 1072 | 489 | 352 |
| **40** | 105 | 210 | 199 | 184 | 185 | 117 | 165 | 173 | 585 | 423 | 319 |
| **59** | 457 | 457 | 465 | 401 | 375 | 390 | 456 | 455 | 1668 | 1066 | 852 |
| **32** | 158 | 139 | 73 | 146 | 148 | 152 | 133 | 168 | 606 | 404 | 266 |
| **53** | 83 | 98 | 95 | 97 | 96 | 97 | 89 | 93 | 564 | 352 | 309 |
| **41** | 579 | 547 | 522 | 360 | 518 | 566 | 589 | 624 | 662 | 755 | 859 |
| **GM** | 502.16 | 509.82 | 469.37 | 495.44 | 517.93 | 499.37 | 510.02 | 537.27 | 1929.10 | 1288.83 | 984.90 |
| **CAT** | | | | | | | | | | | |
| **17** | 112 | 97 | 97 | 100 | 98 | 89 | 104 | 90 | 1409 | 849 | 545 |
| **57** | 1346 | 1155 | 1127 | 1121 | 1093 | 1149 | 1153 | 1304 | 3686 | 2085 | 1593 |
| **09** | 420 | 368 | 356 | 374 | 368 | 353 | 361 | 324 | 801 | 484 | 425 |
| **73** | 183 | 247 | 234 | 344 | 434 | 483 | 439 | 429 | 2863 | 1671 | 1323 |
| **74** | 392 | 333 | 348 | 281 | 265 | 277 | 226 | 244 | 2920 | 1253 | 740 |
| **15** | 778 | 827 | 849 | 895 | 846 | 760 | 867 | 718 | 2368 | 1248 | 976 |
| **33** | 267 | 280 | 352 | 338 | 344 | 378 | 385 | 272 | 3545 | 1752 | 819 |
| **70** | 92 | 109 | 94 | 96 | 99 | 89 | 95 | 89 | 3486 | 1149 | 524 |
| **12** | 83 | 67 | 63 | 71 | 72 | 68 | 87 | 56 | 1917 | 809 | 544 |
| **34** | 255 | 211 | 197 | 200 | 198 | 210 | 239 | 205 | 411 | 303 | 284 |
| **76** | 282 | 239 | 219 | 192 | 213 | 224 | 242 | 163 | 591 | 346 |  |
| **46** | 82 | 70 | 70 | 86 | 102 | 165 | 315 | 139 | 4341 | 1766 | 784 |
| **24** | 205 | 168 | 163 | 217 | 190 | 241 | 246 | 201 | 1489 | 846 | 592 |
| **75** | 108 | 101 | 90 | 94 | 107 | 249 | 273 | 235 | 1844 | 697 | 485 |
| **64** | 1079 | 849 | 1016 | 1506 | 1470 | 1346 | 1342 | 1097 | 9480 | 5004 | 3913 |
| **35** | 322 | 232 | 241 | 199 | 201 | 241 | 233 | 190 | 2161 | 807 | 459 |
| **GM** | 255.35 | 231.42 | 229.89 | 245.02 | 250.67 | 277.95 | 300.97 | 245.77 | 2072.34 | 1030.11 | 732.88 |

Responses were measured pre-vaccination (PreVac), 14 days after the first (DNA1), and second (DNA2), and 14 days and 28 days after the third (DNA3) DNA immunizations, pre-ChAd63 boost and post-ChAd63/pre-CHMI, 35 days and 90 days post-CHMI. GM=geometric mean. Empty boxes indicate when no test was performed.

**Supplementary Table S10: CA and CAT: ELISA antibody responses to AMA1**

| **CA** | | | | | | | | | | | |
| --- | --- | --- | --- | --- | --- | --- | --- | --- | --- | --- | --- |
|  | **Visit #2** | **Visit #6** | **Visit #7** | **Visit #10** | **Visit #11** | **Visit #14** | **Visit #15** | **Visit #17** | **Visit #20** | **Visit #39** | **Visit #40** |
| **Subject**  **ID** | **PreVac** | **14 days post DNA #1** | **DNA Imm #2** | **14 d post DNA Imm #2** | **DNA Imm #3** | **14 d post DNA Imm #3** | **28 d post DNA Imm #3** | **Pre ChAd63 boost** | **Pre-CHMI** | **35 d post CHMI** | **90 d post CHMI** |
| **01** | 86 | 77 | 80 | 269 | 255 | 2282 | 1333 | 268 | 10770 | 25800 | 24100 |
| **78** | 12 | 11 | 10 | 87 | 152 | 1006 | 894 | 263 | 13270 | 24100 | 22300 |
| **68** | 12 | 11 | 14 | 74 | 80 | 762 | 1115 | 317 | 42700 | 37200 | 20000 |
| **88** | 20 | 18 | 17 | 52 | 57 | 556 | 429 | 208 | 16270 | 17600 | 11630 |
| **81** | 22 | 16 | 15 | 15 | 15 | 39 | 50 | 33 | 3480 | 17600 | 11150 |
| **08** | 9 | 9 | 8 | 10 | 9 | 19 | 53 | 92 | 3360 | 6550 | 4935 |
| **82** | 11 | 11 | 12 | 38 | 34 | 706 | 636 | 149 | 30840 | 24300 | 15900 |
| **90** | 27 | 31 | 33 | 313 | 295 | 1112 | 770 | 251 | 26970 | 18050 | 17100 |
| **83** | 14 | 16 | 14 | 117 | 90 | 811 | 1079 | 566 | 35270 | 26600 | 26900 |
| **63** | 14 | 13 | 13 | 13 | 13 | 12 | 12 | 11 | 231 | 11530 | 11310 |
| **20** | 16 | 19 | 20 | 27 | 20 | 54 | 54 | 25 | 3730 | 1370 | 699 |
| **40** | 30 | 32 | 33 | 36 | 37 | 118 | 127 | 46 | 11480 | 3990 | 1944 |
| **59** | 14 | 15 | 13 | 46 | 174 | 671 | 793 | 325 | 43400 | 21200 | 11190 |
| **32** | 22 | 19 | 19 | 70 | 62 | 629 | 561 | 254 | 13460 | 195900 | 94200 |
| **53** | 35 | 37 | 37 | 44 | 88 | 560 | 378 | 109 | 8940 | 4250 | 2785 |
| **41** | 16 | 17 | 16 | 24 | 37 | 316 | 287 | 90 | 6080 | 20650 | 18500 |
| **GM** | 18.76 | 18.49 | 18.31 | 48.05 | 55.13 | 290.84 | 294.32 | 123.84 | 10042.20 | 15621.61 | 10938.23 |
| **CAT** | | | | | | | | | | | |
| **17** | 33 | 42 | 44 | 46 | 53 | 163 | 143 | 74 | 5470 | 3140 | 1948 |
| **57** | 20 | 21 | 21 | 27 | 25 | 34 | 36 | 25 | 1880 | 8460 | 2659 |
| **09** | 22 | 24 | 25 | 30 | 33 | 203 | 168 | 56 | 3644 | 22890 | 41600 |
| **73** | 22 | 45 | 31 | 33 | 33 | 37 | 39 | 34 | 377 | 316 | 186 |
| **74** | 18 | 19 | 19 | 20 | 22 | 86 | 70 | 40 | 6570 | 3220 | 1388 |
| **15** | 29 | 37 | 38 | 1016 | 1031 | 2036 | 1771 | 523 | 17120 | 31980 | 26600 |
| **33** | 11 | 13 | 19 | 704 | 459 | 1721 | 1248 | 214 | 15760 | 34900 | 31200 |
| **70** | 22 | 29 | 28 | 55 | 47 | 200 | 111 | 48 | 4090 | 8080 | 5596 |
| **12** | 15 | 18 | 18 | 168 | 109 | 867 | 1058 | 201 | 30250 | 17200 | 11420 |
| **34** | 11 | 15 | 13 | 51 | 45 | 314 | 207 | 41 | 13620 | 14250 | 8860 |
| **76** | 16 | 20 | 21 | 154 | 182 | 619 | 706 | 130 | 21600 | 26110 |  |
| **46** | 14 | 14 | 14 | 67 | 509 | 1327 | 968 | 283 | 8450 | 5170 | 2574 |
| **24** | 49 | 30 | 31 | 72 | 63 | 1839 | 1229 | 165 | 20750 | 11610 | 5196 |
| **75** | 22 | 19 | 19 | 20 | 21 | 132 | 82 | 33 | 5240 | 2080 | 923 |
| **64** | 45 | 42 | 42 | 63 | 59 | 84 | 93 | 136 | 1460 | 578 | 307 |
| **35** | 27 | 23 | 31 | 104 | 74 | 538 | 321 | 102 | 10740 | 7620 | 4896 |
| **GM** | 21.41 | 23.79 | 24.27 | 73.99 | 77.94 | 306.78 | 251.78 | 89.63 | 6605.35 | 6773.51 | 3793.60 |

Responses were measured pre-vaccination (PreVac), 14 days after the first (DNA1), and second (DNA2), and 14 days and 28 days after the third (DNA3) DNA immunizations, pre-ChAd63 boost and post-ChAd63/pre-CHMI, 35 days and 90 days post-CHMI. GM=geometric mean. Empty boxes indicate when no test was performed.

**Supplementary Table S11: CAT: ELISA antibody responses to TRAP**

| **CAT** | | | | | | | | | | | |
| --- | --- | --- | --- | --- | --- | --- | --- | --- | --- | --- | --- |
|  | **Visit #2** | **Visit #6** | **Visit #7** | **Visit #10** | **Visit #11** | **Visit #14** | **Visit #15** | **Visit #17** | **Visit #20** | **Visit #39** | **Visit #40** |
| **Subject**  **ID** | **PreVac** | **14 days post DNA #1** | **DNA Imm #2** | **14 d post DNA Imm #2** | **DNA Imm #3** | **14 d post DNA Imm #3** | **28 d post DNA Imm #3** | **Pre ChAd63 boost** | **Pre-CHMI** | **35 d post CHMI** | **90 d post CHMI** |
| **17** | 4.44 | 6.24 | 6.86 | 7.95 | 5.22 | 10.91 | 6.10 | 6.62 | 29.60 | 20.71 | 9.58 |
| **57** | 53.24 | 8.56 | 12.00 | 8.66 | 9.95 | 29.12 | 38.11 | 42.10 | 251.31 | 100.09 | 48.28 |
| **09** | 5.50 | 10.35 | 10.81 | 10.82 | 11.74 | 16.57 | 17.31 | 14.87 | 55.57 | 42.39 | 27.13 |
| **73** | 4.84 | 8.67 | 8.67 | 8.72 | 6.89 | 7.60 | 7.68 | 6.01 | 22.69 | 19.11 | 8.06 |
| **74** | 4.51 | 9.87 | 11.64 | 11.00 | 7.60 | 22.93 | 24.71 | 29.43 | 60.49 | 49.19 | 17.71 |
| **15** | 30.52 | 14.93 | 16.88 | 16.11 | 18.38 | 22.11 | 20.39 | 14.58 | 24.02 | 37.60 | 11.24 |
| **33** | 148.86 | 73.08 | 76.63 | 112.55 | 138.51 | 167.05 | 161.49 | 145.72 | 587.09 | 455.35 | 468.61 |
| **70** | 7.25 | 9.19 | 6.13 | 14.02 | 13.56 | 14.12 | 17.73 | 20.19 | 366.84 | 205.25 | 123.36 |
| **12** | 9.17 | 14.12 | 27.41 | 56.33 | 76.79 | 106.44 | 132.00 | 62.78 | 589.14 | 267.78 | 143.28 |
| **34** | 9.75 | 11.32 | 10.93 | 44.99 | 40.00 | 45.89 | 45.04 | 33.29 | 122.90 | 110.30 | 54.08 |
| **76** | 7.13 | 81.99 | 65.37 | 57.06 | 60.74 | 65.17 | 100.24 | 52.43 | 90.21 | 101.20 |  |
| **46** | 1.00 | 1.00 | 1.00 | 26.21 | 50.16 | 119.54 | 125.79 | 60.21 | 664.18 | 408.93 | 197.17 |
| **24** | 1.00 | 1.00 | 1.00 | 14.21 | 10.85 | 37.91 | 30.49 | 10.72 | 252.75 | 164.44 | 101.95 |
| **75** | 10.66 | 1.00 | 1.00 | 1.00 | 1.00 | 4.38 | 2.30 | 2.60 | 83.54 | 55.15 | 37.11 |
| **64** | 26.41 | 2.80 | 4.00 | 19.22 | 20.36 | 24.87 | 33.03 | 20.95 | 87.13 | 69.38 | 54.88 |
| **35** | 9.88 | 1.00 | 1.00 | 1.00 | 1.00 | 7.42 | 3.77 | 1.00 | 72.05 | 58.20 | 29.03 |
| **GM** | 8.94 | 6.72 | 7.23 | 13.87 | 14.04 | 26.40 | 25.35 | 18.00 | 119.32 | 88.02 | 46.63 |

**Supplementary Table S12: CA and CAT: IFA antibody responses to Sporozoites**

| **CA** | | | | | | | | | | | |
| --- | --- | --- | --- | --- | --- | --- | --- | --- | --- | --- | --- |
|  | **Visit #2** | **Visit #6** | **Visit #7** | **Visit #10** | **Visit #11** | **Visit #14** | **Visit #15** | **Visit #17** | **Visit #20** | **Visit #39** | **Visit #40** |
| **Subject ID** | **Prevac** | **14 days post DNA #1** | **DNA Imm #2** | **14 d post DNA Imm #2** | **DNA Imm #3** | **14 d post DNA Imm #3** | **28 d post DNA Imm #3** | **Pre ChAd63 boost** | **27 d post ChAd63 boost or Pre-CHMI** | **35 d post CHMI** | **90 d post CHMI** |
| **01** | 1 | 1 | 20 | 80 | 160 | 320 | 320 | 80 | 2560 | 1280 | 320 |
| **78** | 1 | 1 | 0 | 20 | 40 | 160 | 320 | 320 | 10240 | 1280 | 1280 |
| **68** | 1 | 1 | 20 | 40 | 40 | 80 | 80 | 80 | 1280 | 1280 | 320 |
| **88** | 1 | 1 | 10 | 10 | 20 | 20 | 20 | 20 | 80 | 160 | 320 |
| **81** | 1 | 1 | 1 | 1 | 1 | 10 | 40 | 40 | 160 | 320 | 160 |
| **08** | 1 | 1 | 1 | 1 | 1 | 1 | 1 | 1 | 320 | 320 | 80 |
| **82** | 1 | 40 | 40 | 160 | 160 | 160 | 160 | 80 | 2560 | 2560 | 640 |
| **90** | 1 | 40 | 80 | 320 | 640 | 1280 | 1280 | 5120 | 10240 | 10240 | 5120 |
| **83** | 1 | 80 | 80 | 80 | 80 | 160 | 160 | 640 | 2560 | 1280 | 1280 |
| **63** | 1 | 1 | 10 | 10 | 320 | 320 | 320 | 640 | 1280 | 2560 | 1280 |
| **20** | 1 | 1 | 1 | 0 | 10 | 20 | 40 | 160 | 320 | 320 | 320 |
| **40** | 1 | 1 | 1 | 0 | 0 | 10 | 10 | 20 | 640 | 640 | 320 |
| **59** | 1 | 1 | 1 | 0 | 20 | 40 | 40 | 40 | 2560 | 1280 | 640 |
| **32** | 1 | 1 | 1 | 40 | 80 | 160 | 160 | 160 | 160 | 640 | 640 |
| **53** | 1 | 1 | 10 | 10 | 20 | 20 | 40 | 40 | 640 | 640 | 320 |
| **41** | 1 | 1 | 10 | 20 | 40 | 40 | 80 | 80 | 320 | 640 | 320 |
| **GM** | 1 | 2.08 | 5.63 | 12.09 | 28.33 | 55.76 | 72.34 | 89.84 | 905.10 | 905.09 | 493.51 |
| **CAT** | | | | | | | | | | | |
| **17** | 1 | 20 | 20 | 20 | 40 | 80 | 80 | 80 | 640 | 1280 | 640 |
| **57** | 1 | 10 | 20 | 40 | 80 | 80 | 80 | 80 | 2560 | 2560 | 640 |
| **09** | 1 | 1 | 20 | 20 | 40 | 40 | 80 | 80 | 1280 | 640 | 640 |
| **73** | 1 | 10 | 40 | 320 | 640 | 640 | 640 | 640 | 2560 | 2560 | 1280 |
| **74** | 1 | 1 | 20 | 20 | 80 | 160 | 160 | 160 | 5120 | 2560 | 1280 |
| **15** | 1 | 1 | 10 | 10 | 20 | 40 | 40 | 80 | 1280 | 2560 | 1280 |
| **33** | 1 | 1 | 10 | 20 | 320 | 320 | 320 | 320 | 1280 | 1280 | 640 |
| **70** | 1 | 20 | 40 | 160 | 320 | 320 | 320 | 320 | 2560 | 1280 | 1280 |
| **12** | 1 | 20 | 80 | 2560 | 5120 | 5120 | 5120 | 5120 | 20480 | 10240 | 10240 |
| **34** | 1 | 1 | 1 | 20 | 80 | 80 | 160 | 320 | 320 | 320 | 320 |
| **76** | 1 | 1 | 10 | 1 | 10 | 20 | 40 | 40 | 320 | 640 |  |
| **46** | 1 | 20 | 20 | 320 | 640 | 640 | 640 | 320 | 5120 | 5120 | 1280 |
| **24** | 1 | 1 | 1 | 40 | 40 | 80 | 80 | 80 | 1280 | 640 | 320 |
| **75** | 1 | 1 | 1 | 20 | 20 | 160 | 320 | 320 | 5120 | 5120 | 5120 |
| **64** | 320 | 1280 | 2560 | 2560 | 5120 | 5120 | 2560 | 2560 | 10240 | 20480 | 5120 |
| **35** | 1 | 1 | 1 | 40 | 40 | 40 | 80 | 80 | 640 | 640 | 320 |
| **GM** | 1.43 | 4.41 | 11.58 | 53.42 | 128.84 | 182.21 | 216.68 | 226.27 | 1974.03 | 1890.34 | 1114.30 |

Responses were measured pre-vaccination (PreVac), 14 days after the first (DNA1), and second (DNA2), and 14 days and 28 days after the third (DNA3) DNA immunizations, pre-ChAd63 boost and post-ChAd63/pre-CHMI, 35 days and 90 days post-CHMI. GM=geometric mean. Empty boxes indicate when no test was performed.

**Supplementary Table S13: CA and CAT: IFA antibody responses to Blood Stages**

| **CA** | | | | | | | | | | | |
| --- | --- | --- | --- | --- | --- | --- | --- | --- | --- | --- | --- |
|  | **Visit #2** | **Visit #6** | **Visit #7** | **Visit #10** | **Visit #11** | **Visit #14** | **Visit #15** | **Visit #17** | **Visit #20** | **Visit #39** | **Visit #40** |
| **Subject ID** | **Prevac** | **14 days post DNA #1** | **DNA Imm #2** | **14 d post DNA Imm #2** | **DNA Imm #3** | **14 d post DNA Imm #3** | **28 d post DNA Imm #3** | **Pre ChAd63 boost** | **27 d post ChAd63 boost or Pre-CHMI** | **35 d post CHMI** | **90 d post CHMI** |
| **01** | 160 | 160 | 160 | 320 | 320 | 640 | 640 | 640 | 1280 | 2560 | 1280 |
| **78** | 1 | 40 | 40 | 40 | 160 | 320 | 640 | 320 | 1280 | 2560 | 2560 |
| **68** | 1 | 40 | 40 | 80 | 80 | 160 | 160 | 160 | 2560 | 5120 | 1280 |
| **88** | 40 | 40 | 80 | 160 | 80 | 80 | 160 | 80 | 2560 | 5120 | 1280 |
| **81** | 1 | 80 | 80 | 80 | 80 | 80 | 80 | 80 | 320 | 20480 | 5120 |
| **08** | 1 | 0 | 40 | 40 | 40 | 80 | 80 | 80 | 160 | 640 | 640 |
| **82** | 80 | 160 | 160 | 160 | 320 | 320 | 320 | 320 | 1280 | 5120 | 2560 |
| **90** | 1 | 160 | 160 | 160 | 160 | 160 | 320 | 640 | 1280 | 1280 | 640 |
| **83** | 10 | 160 | 80 | 160 | 160 | 160 | 80 | 160 | 640 | 1280 | 1280 |
| **63** | 1 | 320 | 160 | 320 | 320 | 640 | 320 | 320 | 320 | 5120 | 2560 |
| **20** | 1 | 320 | 320 | 320 | 320 | 320 | 320 | 320 | 640 | 320 | 160 |
| **40** | 1 | 320 | 320 | 640 | 640 | 640 | 640 | 640 | 1280 | 1280 | 640 |
| **59** | 40 | 640 | 1280 | 1280 | 1280 | 1280 | 1280 | 1280 | 2560 | 2560 | 1280 |
| **32** | 40 | 320 | 320 | 320 | 640 | 640 | 640 | 640 | 1280 | 5120 | 5120 |
| **53** | 1 | 320 | 320 | 320 | 640 | 640 | 640 | 640 | 1280 | 1280 | 160 |
| **41** | 1 | 320 | 320 | 320 | 320 | 160 | 160 | 160 | 160 | 2560 | 2560 |
| **GM** | 4.16 | 121.67 | 153.22 | 198.70 | 236.29 | 281.00 | 293.44 | 293.44 | 866.72 | 2451.6 | 1225.3 |
| **CAT** | | | | | | | | | | | |
| **17** | 1 | 40 | 80 | 80 | 80 | 320 | 320 | 160 | 320 | 160 | 320 |
| **57** | 40 | 320 | 640 | 640 | 640 | 640 | 640 | 640 | 1280 | 20480 | 10240 |
| **09** | 1 | 80 | 80 | 160 | 80 | 160 | 160 | 160 | 320 | 1280 | 5120 |
| **73** | 40 | 1280 | 1280 | 1280 | 2560 | 1280 | 1280 | 1280 | 1280 | 2560 | 5120 |
| **74** | 10 | 160 | 640 | 320 | 640 | 640 | 640 | 640 | 640 | 640 | 320 |
| **15** | 1 | 80 | 80 | 80 | 80 | 80 | 80 | 80 | 160 | 2560 | 2560 |
| **33** | 10 | 160 | 320 | 320 | 320 | 320 | 320 | 320 | 640 | 5120 | 2560 |
| **70** | 40 | 320 | 1280 | 1280 | 1280 | 640 | 1280 | 1280 | 1280 | 10240 | 10240 |
| **12** | 20 | 160 | 320 | 320 | 320 | 320 | 640 | 640 | 2560 | 2560 | 1280 |
| **34** | 1 | 160 | 80 | 160 | 80 | 160 | 160 | 320 | 2560 | 1280 | 1280 |
| **76** | 40 | 320 | 640 | 640 | 640 | 640 | 640 | 1280 | 2560 | 10240 |  |
| **46** | 20 | 160 | 320 | 320 | 320 | 320 | 640 | 640 | 1280 | 1280 | 640 |
| **24** | 20 | 320 | 320 | 320 | 320 | 640 | 640 | 1280 | 1280 | 2560 | 1280 |
| **75** | 20 | 320 | 640 | 640 | 640 | 640 | 640 | 640 | 1280 | 1280 | 640 |
| **64** | 20 | 640 | 640 | 640 | 640 | 320 | 320 | 640 | 320 | 640 | 640 |
| **35** | 1 | 160 | 160 | 80 | 160 | 160 | 160 | 320 | 640 | 640 | 320 |
| **GM** | 8.55 | 207.49 | 320.00 | 320.00 | 334.17 | 364.41 | 414.99 | 493.51 | 866.72 | 1974.3 | 1470.3 |

Responses were measured pre-vaccination (PreVac), 14 days after the first (DNA1), and second (DNA2), and 14 days and 28 days after the third (DNA3) DNA immunizations, pre-ChAd63 boost and post-ChAd63/pre-CHMI, 35 days and 90 days post-CHMI. GM=geometric mean. Empty boxes indicate when no test was performed.

**Supplementary Table S14: CA and CAT: FluoroSpot IFN-γ responses to CSP**

| **CA** | | | | | | |
| --- | --- | --- | --- | --- | --- | --- |
|  | **Visit #2** | **Visit**  **#15** | **Visit**  **#17** | **Visit**  **#20** | **Visit**  **#39** | **Visit**  **#40** |
| **Subject ID** | **Prevac** | **28 d post DNA Imm #3** | **Pre ChAd63 boost** | **27 d post ChAd63 boost or Pre-CHMI** | **35d post CHMI** | **90d post CHMI** |
| **01** | 21 | 146 | 180 | 284 | 255 | 144 |
| **78** | 26 | 88 | 35 | 119 | 146 | 63 |
| **68** | 16 | 116 | 29 | 96 | 74 | 294 |
| **88** | 27 | 40 | 195 | 233 | 280 | 36 |
| **81** | 10 | 29 | 6 | 25 | 79 | 21 |
| **08** | 5 | 9 | 9 | 16 | 36 | 51 |
| **82** | 14 | 4 | 27 | 88 | 51 | 75 |
| **90** | 20 | 83 | 44 | 78 | 113 | 89 |
| **83** | 1 | 76 | 51 | 124 | 26 | 78 |
| **63** | 0 | 11 | 20 | 148 | 91 | 76 |
| **20** | 20 | 64 | 26 | 54 | 59 | 51 |
| **40** | 3 | 14 | 49 | 56 | 24 | 53 |
| **59** | 12 | 75 | 21 | 234 | 148 | 108 |
| **32** | 8 | 36 | 28 | 62 | 51 | 43 |
| **53** | 38 | 33 | 151 | 61 | 186 | 42 |
| **41** | 29 | 50 | 43 | 105 | 89 | 41 |
| **GM** | 10 | 38 | 37 | 87 | 83 | 64 |
| **No. Pos. (%)** | 0/16 (0%) | 4/16 (25%) | 4/16 (25%) | 10/16 (63%) | 10/16 (63%) | 7/16 (44%) |
| **CAT** | | | | | | |
| **17** | 106 | 66 | 70 | 64 | 68 | 53 |
| **57** | 6 | 111 | 31 | 100 | 60 | 28 |
| **09** | 48 | 69 | 18 | 77 | 139 | 107 |
| **73** | 4 | 9 | 13 | 68 | 75 | 69 |
| **74** | 3 | 27 | 18 | 111 | 20 | 44 |
| **15** | 54 | 58 | 71 | 140 | 60 | 84 |
| **33** | 22 | 73 | 35 | 224 | 159 | 149 |
| **70** | 9 | 6 | 8 | 61 | 53 | 59 |
| **12** | 20 | 408 | 336 | 611 | 457 | 293 |
| **34** | 9 | 28 | 4 | 21 | 48 | 34 |
| **76** | 10 | 44 | 94 | 118 | 128 |  |
| **46** | 111 | 76 | 156 | 106 | 151 | 148 |
| **24** | 12 | 19 | 10 | 24 | 32 | 35 |
| **75** | 31 | 21 | 34 | 56 | 43 | 54 |
| **64** | 0 | 123 | 66 | 263 | 146 | 91 |
| **35** | 6 | 23 | 38 | 56 | 20 | 38 |
| **GM** | 14 | 43 | 34 | 92 | 73 | 68 |
| **No. Pos. %** | 2/16 (13%) | 7/16 (44%) | 5/16 (31%) | 12/16 (75%) | 9/16 (56%) | 6/15 (40%) |

Summed responses were measured pre-vaccination (PreVac), 28 days after the third (DNA3) DNA immunizations, pre-ChAd63 boost and 27 days before post-ChAd63/pre-CHMI, 35 days and 90 days post-CHMI. GM=geometric mean. Subjects with positive responses (see Methods) are shown in red. Empty boxes indicate when no test was performed.

**Supplementary Table S15: CA and CAT: FluoroSpot IFN-γ responses to AMA1**

| **CA** | | | | | | |
| --- | --- | --- | --- | --- | --- | --- |
|  | **Visit #2** | **Visit**  **#15** | **Visit**  **#17** | **Visit**  **#20** | **Visit**  **#39** | **Visit**  **#40** |
| **Subject ID** | **Prevac** | **28 d post DNA Imm #3** | **Pre ChAd63 boost** | **27 d post ChAd63 boost or Pre-CHMI** | **35d post CHMI** | **90d post CHMI** |
| **01** | 77 | 481 | 461 | 686 | 703 | 556 |
| **78** | 48 | 464 | 292 | 418 | 613 | 139 |
| **68** | 15 | 330 | 121 | 403 | 384 | 371 |
| **88** | 127 | 125 | 546 | 578 | 706 | 76 |
| **81** | 111 | 114 | 66 | 156 | 294 | 105 |
| **08** | 10 | 33 | 28 | 188 | 344 | 253 |
| **82** | 59 | 46 | 96 | 609 | 367 | 373 |
| **90** | 83 | 1938 | 577 | 2549 | 1530 | 1110 |
| **83** | 54 | 268 | 51 | 1083 | 546 | 616 |
| **63** | 0 | 0 | 14 | 130 | 78 | 63 |
| **20** | 53 | 86 | 49 | 86 | 128 | 84 |
| **40** | 2 | 55 | 178 | 454 | 151 | 124 |
| **59** | 15 | 113 | 83 | 565 | 376 | 280 |
| **32** | 8 | 1319 | 1856 | 2443 | 2388 | 2283 |
| **53** | 98 | 281 | 224 | 444 | 350 | 192 |
| **41** | 28 | 304 | 218 | 310 | 483 | 258 |
| **GM** | 27 | 147 | 146 | 453 | 417 | 254 |
| **No. Pos. (%)** | 7/16 (44%) | 11/16 (69%) | 10/16 (63%) | 16/16 (100%) | 16/16 (100%) | 14/16 (88%) |
| **CAT** | | | | | | |
| **17** | 104 | 81 | 97 | 116 | 85 | 51 |
| **57** | 6 | 215 | 84 | 311 | 215 | 38 |
| **09** | 205 | 123 | 93 | 168 | 431 | 241 |
| **73** | 4 | 88 | 49 | 597 | 594 | 342 |
| **74** | 11 | 125 | 63 | 317 | 156 | 169 |
| **15** | 58 | 55 | 141 | 55 | 110 | 105 |
| **33** | 28 | 1000 | 462 | 2513 | 1433 | 1901 |
| **70** | 9 | 35 | 43 | 152 | 211 | 264 |
| **12** | 49 | 745 | 758 | 1214 | 631 | 748 |
| **34** | 58 | 261 | 203 | 496 | 651 | 392 |
| **76** | 26 | 68 | 64 | 179 | 203 | 389 |
| **46** | 75 | 432 | 236 | 648 | 403 |  |
| **24** | 12 | 736 | 548 | 4668 | 1595 | 1500 |
| **75** | 43 | 224 | 143 | 418 | 289 | 213 |
| **64** | 25 | 360 | 253 | 701 | 436 | 371 |
| **35** | 21 | 169 | 224 | 779 | 249 | 563 |
| **GM** | 28 | 188 | 150 | 437 | 345 | 294 |
| **No. Pos. %** | 3/16 (19%) | 12/16 (75%) | 12/16 (75%) | 15/16 (94%) | 14/16 (88%) | 12/15 (80%) |

Summed responses were measured pre-vaccination (PreVac), 28 days after the third (DNA3) DNA immunizations, pre-ChAd63 boost and 27 days before post-ChAd63/pre-CHMI, 35 days and 90 days post-CHMI. GM=geometric mean. Subjects with positive responses (see Methods) are shown in red. Empty boxes indicate when no test was performed.

**Supplementary Table S16: CAT: FluoroSpot IFN-γ responses to T9/96 and 3D7 TRAP**

| **T9/96 TRAP** | | | | | | |
| --- | --- | --- | --- | --- | --- | --- |
|  | **Visit #2** | **Visit**  **#15** | **Visit**  **#17** | **Visit**  **#20** | **Visit**  **#39** | **Visit**  **#40** |
| **Subject ID** | **Prevac** | **28 d post DNA Imm #3** | **Pre ChAd63 boost** | **27 d post ChAd63 boost or Pre-CHMI** | **35d post CHMI** | **90d post CHMI** |
| **17** | 19 | 73 | 84 | 19 | 18 | 14 |
| **57** | 16 | 53 | 19 | 39 | 29 | 29 |
| **09** | 200 | 118 | 123 | 94 | 265 | 155 |
| **73** | 11 | 17 | 8 | 126 | 88 | 84 |
| **74** | 25 | 62 | 46 | 139 | 81 | 103 |
| **15** | 64 | 23 | 21 | 14 | 14 | 38 |
| **33** | 49 | 1039 | 479 | 3033 | 2443 | 2586 |
| **70** | 12 | 30 | 48 | 198 | 153 | 191 |
| **12** | 126 | 348 | 319 | 625 | 336 | 403 |
| **34** | 230 | 123 | 108 | 191 | 255 | 158 |
| **76** | 39 | 14 | 33 | 54 | 59 |  |
| **46** | 21 | 90 | 39 | 84 | 71 | 91 |
| **24** | 14 | 121 | 123 | 765 | 384 | 486 |
| **75** | 128 | 369 | 193 | 490 | 493 | 435 |
| **64** | 92 | 114 | 224 | 408 | 266 | 263 |
| **35** | 165 | 326 | 259 | 641 | 268 | 686 |
| **GM** | 46 | 93 | 78 | 171 | 141 | 167 |
| **No. Pos. %** | 7/16 (44%) | 11/16 (69%) | 10/16 (63%) | 12/16 (75%) | 12/16 (75%) | 11/15 (73%) |
| **3D7 TRAP** | | | | | | |
| **17** | 18 | 89 | 96 | 45 | 40 | 26 |
| **57** | 9 | 72 | 29 | 44 | 40 | 14 |
| **09** | 119 | 148 | 144 | 144 | 350 | 218 |
| **73** | 13 | 86 | 13 | 108 | 146 | 93 |
| **74** | 21 | 92 | 59 | 158 | 118 | 106 |
| **15** | 58 | 26 | 24 | 6 | 14 | 14 |
| **33** | 44 | 1265 | 654 | 3431 | 2715 | 2890 |
| **70** | 11 | 70 | 54 | 253 | 233 | 241 |
| **12** | 109 | 416 | 316 | 536 | 288 | 349 |
| **34** | 180 | 178 | 139 | 149 | 313 | 186 |
| **76** | 53 | 19 | 78 | 66 | 101 |  |
| **46** | 16 | 83 | 26 | 68 | 64 | 54 |
| **24** | 20 | 178 | 254 | 718 | 390 | 471 |
| **75** | 123 | 374 | 164 | 556 | 655 | 433 |
| **64** | 86 | 133 | 228 | 386 | 284 | 240 |
| **35** | 103 | 428 | 336 | 691 | 283 | 729 |
| **GM** | 41 | 131 | 98 | 178 | 178 | 158 |
| **No. Pos. %** | 5/16 (31%) | 12/16 (75%) | 10/16 (63%) | 13/16 (81%) | 12/16 (75%) | 11/15 (67%) |

Summed responses were measured pre-vaccination (PreVac), 28 days after the third (DNA3) DNA immunizations, pre-ChAd63 boost and 27 days before post-ChAd63/pre-CHMI, 35 days and 90 days post-CHMI. GM=geometric mean. Subjects with positive responses (see Methods) are shown in red. Empty boxes indicate when no test was performed.

**Supplementary Table S17: Pre-existing neutralizing antibodies (Nab) to HuAd5 before and after ChAd63 boosts in CA and CAT groups**

| **CA** | | | **CAT** | | |
| --- | --- | --- | --- | --- | --- |
| **Subject** | **Pre-ChAd63** | **Post-ChAd63** | **Subject** | **Pre-ChAd63** | **Post-ChAd63** |
| 1 | 1 | 53.2 | 17 | 83.9 | 163.9 |
| 78 | 551.5 | 1328.3 | 57 | 1911.8 | 3725.7 |
| 68 | 32.7 | 278.4 | 9 | 1 | 1 |
| 88 | 1.1 | 1.6 | 73 | 221.5 | 315.1 |
| 81 | 1.5 | 10.2 | 74 | 1062.3 | 650.0 |
| 8 | 6.3 | 21.6 | 15 | 3866.1 | 6137.9 |
| 82 | 22.9 | 402.1 | 33 | 1 | 1.2 |
| 90 | 1 | 1 | 70 | 600.8 | 916.1 |
| 83 | 3.9 | 1 | 12 | 872.5 | 656.2 |
| 63 | 1 | 30.5 | 34 | 80.0 | 108.8 |
| 20 | 1840.8 | 2417.6 | 76 | 883.3 | 806.6 |
| 40 | 974.9 | 2338.5 | 46 | 23.5 | 213.9 |
| 59 | 1 | 1 | 24 | 1355.7 | 1184.0 |
| 32 | 1211.6 | 4102.1 | 75 | 2.3 | 7.5 |
| 53 | 1531.1 | 8599 | 64 | 525.0 | 326.8 |
| 41 | 329.6 | 711.6 | 35 | 3.1 | 1 |
| **GM** | 24.6 | 87.8 | **GM** | 112.2 | 142.7 |

Nab were measured pre-ChAd63 (visit 17) and post-ChAd63 (visit 20). Activities >500 are considered positive (red). Thirteen/32 subjects had positive Nab pre-ChAd63, and 12/13 remained positive post-ChAd63 and one (v64) became negative. One subject with negative Nab responses pre-ChAd63 (v41) became weakly positive post-ChAd63.

**Supplementary Table S18. Rank correlations between pre-existing anti-HuAd5 Nab titers and FluoroSpot responses and ELISA and IFA titers.**

| \| **GROUP** \|  \| **FluoroSpot** \| \| \| \| **ELISA** \| \| \| \| \| **IFA** \| \| \| --- \| --- \| --- \| --- \| --- \| --- \| --- \| --- \| --- \| --- \| --- \| --- \| --- \| \|  \|  \| **CSP** \| **AMA1** \| **TRAP TTm** \| **TRAP TDm** \| **CSP FL** \| **CSP**  **rpt** \| **CSP**  **Pf16** \| **AMA1** \| **TRAP** \| **SPZ** \| **RBC** \| \| CA \| r \| -0.39 \| -0.34 \| NT \| NT \| 0.13 \| -0.14 \| 0.42 \| 0.13 \| NT \| 0.21 \| -0.08 \| \|  \| p \| 0.13 \| 0.19 \| NT \| NT \| 0.62 \| 0.59 \| 0.17 \| 0.62 \| NT \| 0.44 \| 0.77 \| \|  \|  \|  \|  \|  \|  \|  \|  \|  \|  \|  \|  \|  \| \| CAT \| r \| -0.19 \| -0.37 \| -0.34 \| -0.25 \| 0.15 \| 0.009 \| 0.08 \| -0.09 \| -0.35 \| 0.20 \| 0.21 \| \|  \| p \| 0.52 \| 0.16 \| 0.20 \| 0.35 \| 0.58 \| 0.97 \| 0.76 \| 0.73 \| 0.18 \| 0.47 \| 0.45 \| |  |  |  |  |  |  |  |  |  |  |  |  |  |  |  |  |  |  |
| --- | --- | --- | --- | --- | --- | --- | --- | --- | --- | --- | --- | --- | --- | --- | --- | --- | --- | --- | --- | --- | --- | --- | --- | --- | --- | --- | --- | --- | --- | --- | --- | --- | --- | --- | --- | --- | --- | --- | --- | --- | --- | --- | --- | --- | --- | --- | --- | --- | --- | --- | --- | --- | --- | --- | --- | --- | --- | --- | --- | --- | --- | --- | --- | --- | --- | --- | --- | --- | --- | --- | --- | --- | --- | --- | --- | --- | --- | --- | --- | --- | --- | --- | --- | --- | --- | --- | --- | --- | --- | --- | --- | --- | --- | --- | --- | --- | --- | --- | --- | --- | --- | --- | --- | --- | --- | --- | --- | --- | --- |
|  |  |  |  |  |  |  |  |  |  |  |  |  |  |  |  |  |  |  |

Pre-existing HuAd5 Nab titers measured just prior to ChAd63 immunization were tested for negative correlations with IFN-γ FluoroSpot, ELISA and sporozoite IFA for all subjects: r = rank correlation coefficient, and p = p value for the null hypothesis that the correlation is zero (two-tailed). All associations were negative. NT = assay was not performed. See text for abbreviations.
